# Biosynthesis of (+)-palitantin requires homologs of alkyl salicylaldehyde-forming enzymes without involvement of an aromatic intermediate

**DOI:** 10.1039/d6sc04815c

**Published:** 2026-08-07

**Authors:** Rui Ge, Zheng-Xi Zhang, Li Yang, Li-Dan Pang, Shu-Ming Li

**Affiliations:** a Philipps-Universität Marburg, Fachbereich Pharmazie, Institut für Pharmazeutische Biologie und Biotechnologie Robert-Koch-Straße 4 Marburg 35037 Germany shuming.li@staff.uni-marburg.de; b Haikou Key Laboratory for Research and Utilization of Tropical Natural Products, Institute of Tropical Bioscience and Biotechnology, Chinese Academy of Tropical Agricultural Sciences Haikou 571101 P. R. China

## Abstract

(+)-Palitantin, a bioactive fungal polyketide from *Penicillium palitans*, features a densely functionalized cyclohexanone core with multiple stereocenters. Here, we elucidate its biosynthetic pathway by gene deletion experiments as well as *in vivo* and *in vitro* reconstitution of the key steps. The formation of the cyclohexanone scaffold in palitantin is catalyzed by homologs of alkyl salicylaldehyde-forming enzymes without involving an aromatic intermediate. In contrast, the products of known alkyl salicylaldehyde-forming enzymes are aromatic derivatives, including that in the non-aromatic trichoxide pathway. Furthermore, the cupin protein PaltE enhances the formation of an unstable cyclohexenone intermediate for stereospecific ene reduction by PaltF and ensures efficient flux toward (+)-palitantin. Subsequent aldehyde reduction by the SDR PaltG and stereospecific hydroxylation by the P450 enzyme PaltD complete the pathway and establish the stereochemically complex decoration on the cyclohexanone scaffold. Our work expands the functional scope of alkyl salicylaldehyde-forming enzymes and provides a highly programmed strategy for natural product biosynthesis.

## Abstract 2

Alkyl salicylaldehyde-forming enzyme homologs in the biosynthetic pathway of (+)-palitantin in *Penicillium palitans* assemble a non-aromatic skeleton for subsequent stereo controlled modifications by three oxidoreductases.

## Graphical abstract

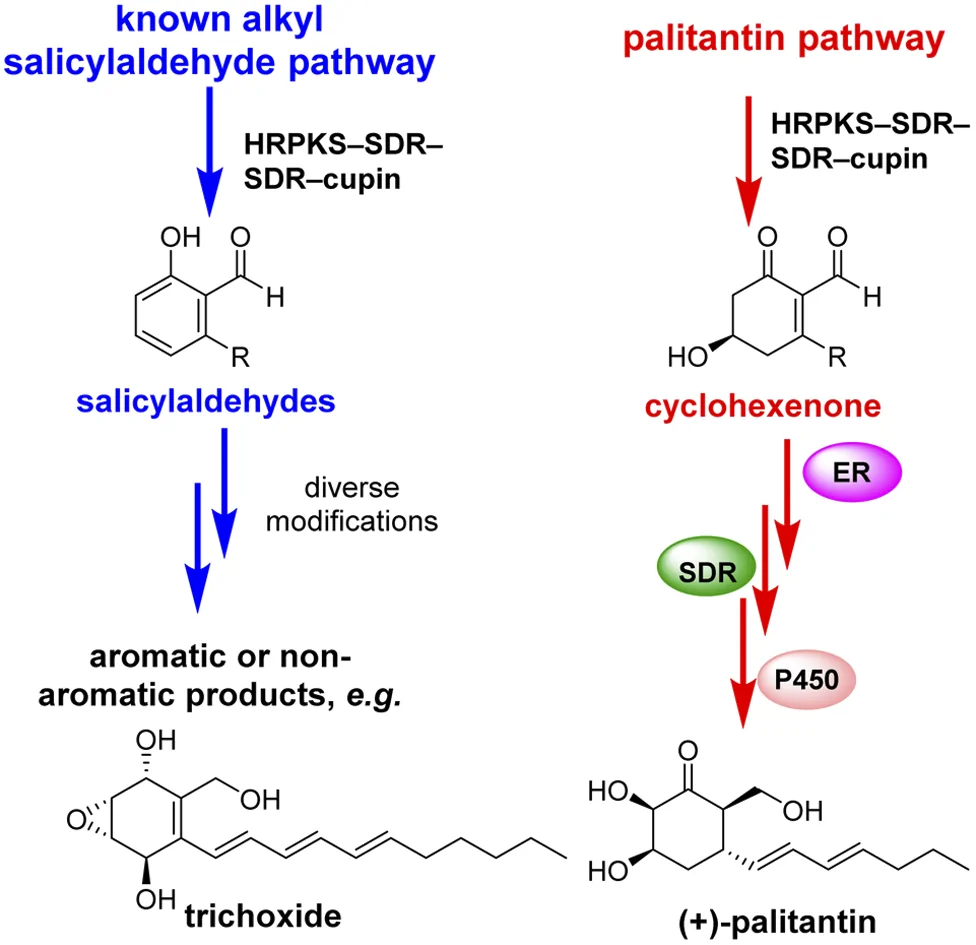

## Introduction

Cyclohexanone-derived scaffolds are privileged frameworks in drug discovery and serve as central motifs in a broad spectrum of bioactive molecules.^1^ The fungal polyketide (+)-palitantin, first isolated from *Penicillium palitans* in 1936, features a densely functionalized cyclohexanone core bearing multiple stereocenters (Fig. 1A).^2^ It exhibits inhibitory activity against acetylcholinesterase and protein tyrosine phosphatase 1B.^3^ Despite decades of efforts in chemical synthesis, the stereo controlled construction of the highly substituted cyclohexanone core of (+)-palitantin remains a formidable challenge.^4^ The enzymatic logic of (+)-palitantin assembly has not been resolved.

**Fig. 1:**
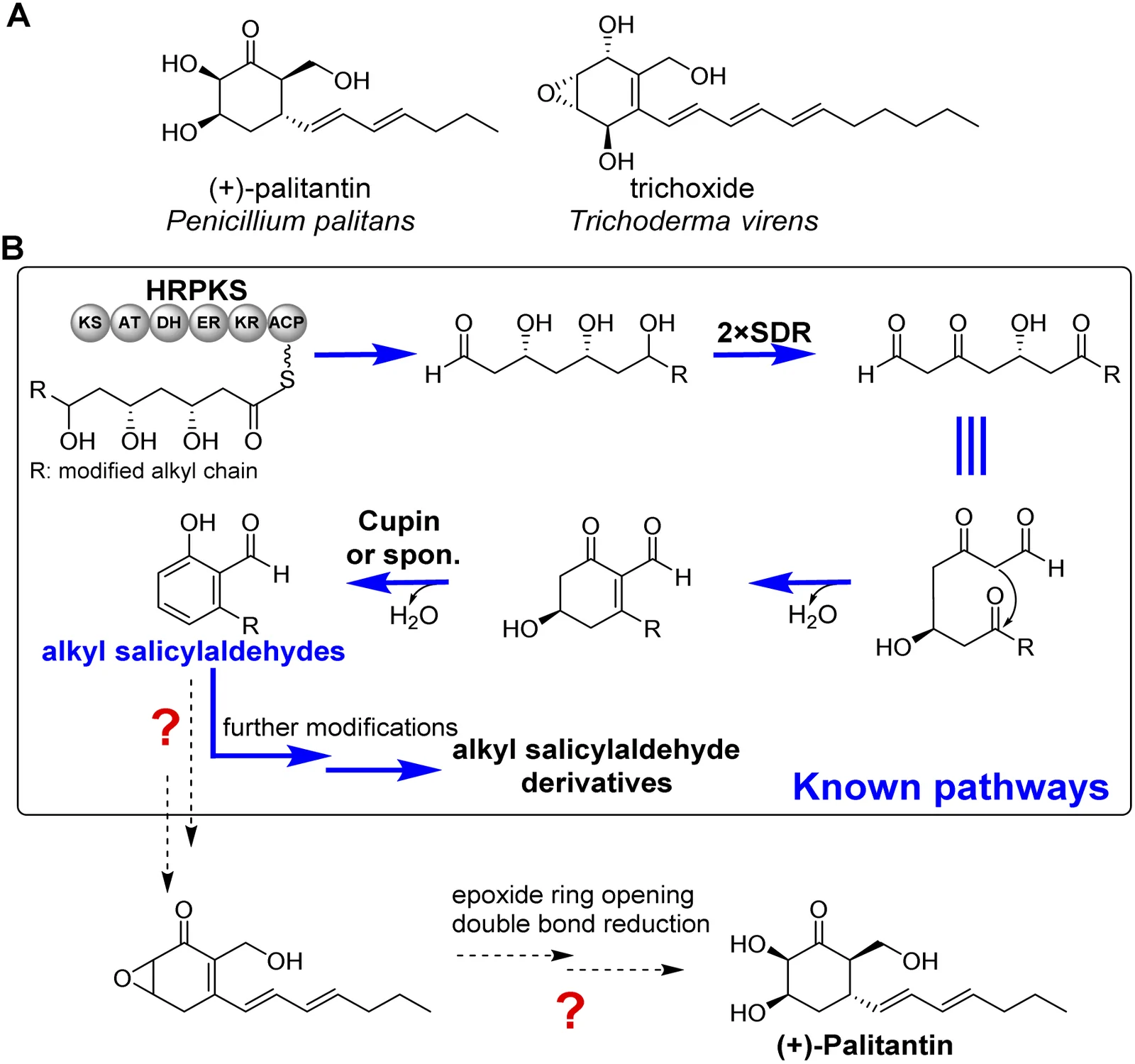
(A) Structural comparison of (+)-palitantin and trichoxide. (B) Simplified known biosynthetic pathways of fungal alkyl salicylaldehydes with a hypothesis for (+)-palitantin formation.

To investigate the biosynthesis of (+)-palitantin, we use compounds with structurally related scaffolds to identify candidate genes/gene clusters. Trichoxide, a fungal metabolite derived from alkyl salicylaldehyde, features a reduced epoxycyclohexenol scaffold with a substitution pattern reminiscent of (+)-palitantin (Fig. 1A). Elucidation of the biosynthesis in 2019 revealed that its scaffold is formed by dearomative epoxidation on the salicylaldehyde ring.^5^ Like most fungal alkyl salicylaldehyde derivatives, the salicylaldehyde precursor in trichoxide is assembled by the combined action of four proteins: a highly reducing polyketide synthase (HR-PKS), two short-chain dehydrogenases/reductases (SDRs), and a cupin domain-containing protein (Fig. 1B and S1). Subsequent modifications by different tailoring enzymes lead to diverse structures (Fig. S1).^5–11^ We therefore hypothesized that (+)-palitantin might be biosynthesized *via* an analogous pathway, *i.e.*, formation of an alkyl salicylaldehyde intermediate followed by dearomative epoxidation and subsequent hydrolytic epoxide ring opening (Fig. 1B). Such epoxide-mediated dearomatization has been reported for different enzyme classes like cupin domain-containing proteins or P450 monooxygenases.^5,12^

However, early feeding experiments with ^2^H-, ^13^C-, and ^18^O-labeled acetate suggested that the cyclohexane core of (+)-palitantin is formed without involvement of an aromatic intermediate.^13^ Based on the isotope-labeling patterns, a cyclohexenone-like intermediate was proposed, with reduction of the enone moiety preventing potential aromatization. On the other hand, neither the postulated intermediate nor the enzyme responsible for the key ene reduction was detected or identified. Nevertheless, the isotope-labeling proposal appears difficult to align with that illustrated in Fig. 1B. This controverse hypotheses raised our interest to elucidate the biosynthetic pathway of (+)-palitantin and to identify the key enzymes.

## Results and discussion

### Isolation and identification of (+)-palitantin and congeners

(+)-Palitantin (1), together with six related metabolites 2–7, was isolated from a 14-day old PDB culture of *P. palitans* NRRL 2033 (WT) (Fig. 2B(ii)). The NMR data of 1 and 4 were consistent with those reported for (+)-palitantin and penihydrone, respectively,^14^ whereas 2, 3, 5, 6, and 7 were identified by extensive spectroscopic analysis to be new metabolites, termed dehydropenihydrone A, dehydropenihydrone B, penihydrone B, palitanol A, and palitanol B, respectively (Tables S7–S13 and Fig. S21–S54, see SI for structural elucidation). Compounds 2–5 are (+)-palitantin congeners with the same carbon number and alkylated hexanone ring and differ from each other by substituents, oxidation states, and stereochemistry at the cyclohexane ring (Fig. 2C and Scheme 1). Compound 4 lacks the 3-OH group in (+)-palitantin, whereas 5 was identified as its C1 epimer. Compounds 2 and 3 are C1–C6 dehydrogenated derivatives of 4 (Scheme 1). Compound 6 exists in DMSO-*d*_6_ (solvent for NMR measurement) as a mixture of two hemiacetalic tetrahydropyrans, with 1β-OH and 1α-OH in a ratio of 7 : 3 (Fig. S43). Its HPLC chromatograms showed two peaks at 15.7 and 17.2 min (Fig. 2B(v) and 3(iii)). Reinjection of the isolated peak at 15.7 min led to the detection of both peaks again. Compound 7 is an aliphatic and alcoholic pathway intermediate.

**Fig. 2:**
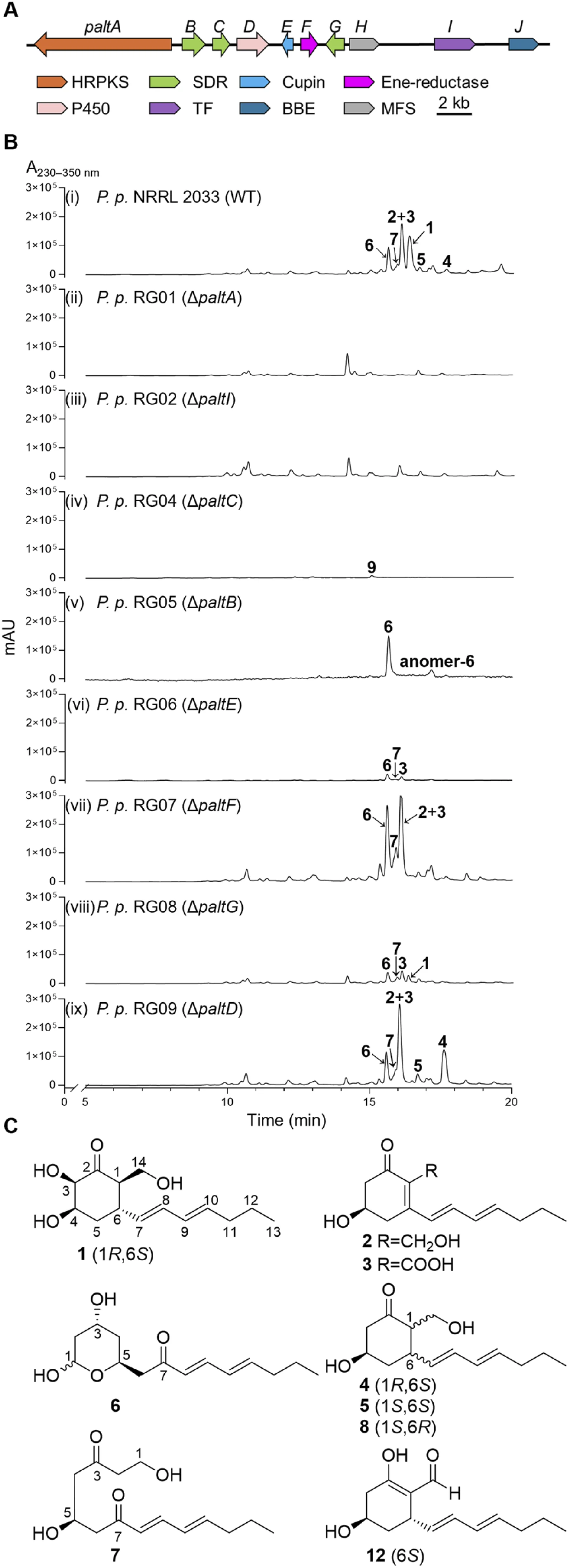
(A) Putative *palt* gene cluster from *P. palitans*. (Bi–ix) Metabolite analysis of *P. palitans* strains by using LC-MS method B with UV-detection at 230–350 nm. The overlapping peaks of 2 and 3 were resolved by EIC detection (Fig. S7A). (C) Structures of the isolated products.

**Scheme 1:**
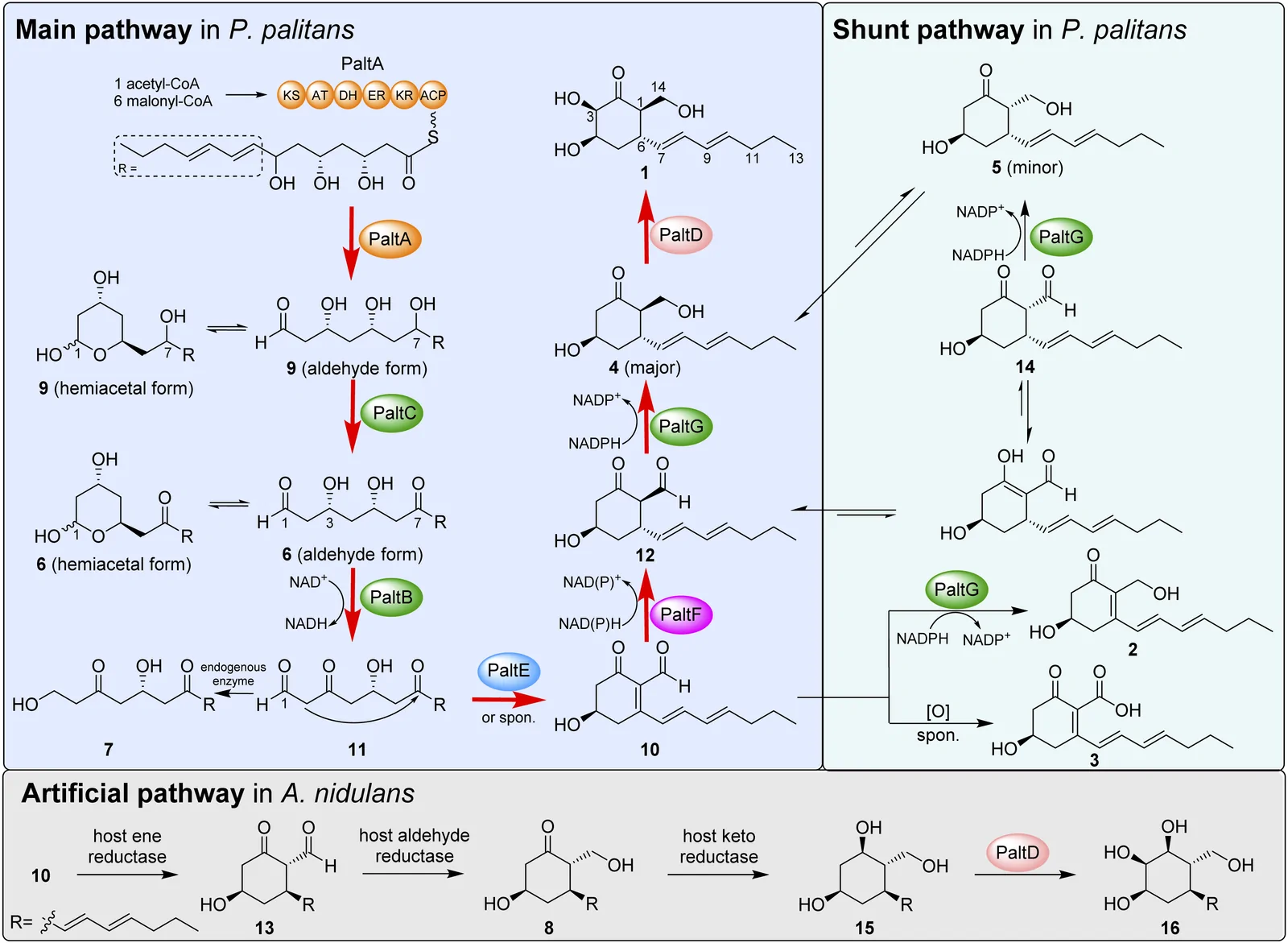
Proposed biosynthetic pathway of (+)-palitantin (1) and congeners.

### Identification of the putative (+)-palitantin biosynthetic gene cluster

To elucidate the biosynthetic pathway of (+)-palitantin, the genome of *P. palitans* NRRL 2033 was sequenced.^15^ Mining the sequence revealed a candidate cluster with ten putative genes, hereafter termed the *palt* cluster (GenBank accession no. PZ404400, Fig. 2A and Table S1). The cluster codes for the four core proteins associated with alkyl salicylaldehyde formation mentioned above: the HR-PKS PaltA, two SDRs PaltB and PaltC, and the cupin domain-containing protein PaltE. Additional genes coding for the third SDR PaltG, a putative NAD(P)H-dependent oxidoreductase PaltF, a cytochrome P450 enzyme PaltD, a MFS-type transporter PaltH, a transcription factor (TF) PaltI, and a berberine bridge enzyme (BBE)-like protein PaltJ are also found in the cluster (Table S1). Orthologous clusters are present in genomes of 14 *Penicillium palitans* strains, with nucleotide sequence identities of up to 99% (Table S2). Comparison of the four core enzymes from the *palt* cluster with their homologs in the pathways of the characterized fungal alkyl salicylaldehyde derivatives, including sordarial,^6^ trichoxide,^5^ flavoglaucin,^7^ annullatin D,^8^ stachysalicyloid C,^9^ 6-propyl salicyl alcohol,^10^ and roquesalin B^11^ (Fig. S1), showed significant sequence identities of at least 43% at the amino acid level (Table S3).

To confirm the involvement of the *palt* cluster in (+)-palitantin biosynthesis, we first knocked out the PKS gene *paltA* and the putative transcription factor gene *paltI* individually in *P. palitans* NRRL 2033 using a split-marker strategy (Fig. S2 and S3).^16^ The resulting mutants RG01 and RG02 showed complete production abolishment of (+)-palitantin as well as compounds 2–7 (Fig. 2B(ii) and (iii)), proving their essential roles in the biosynthesis. Deletion of *paltJ* in the WT strain did not lead to detectable changes in the metabolite profile, suggesting that this BBE-like enzyme, sharing 50.5% sequence identity with AnuG from *Penicillium roqueforti*,^8^ is likely not involved in the (+)-palitantin biosynthesis (Fig. S5).

### Homologs of the alkyl salicylaldehyde-forming enzymes catalyze the formation of a cyclohexenone precursor for further modification

Having associated the *palt* cluster with (+)-palitantin biosynthesis, we next examined the HRPKS–SDR–SDR–cupin enzyme set encoded by *paltA*, *paltC*, *paltB*, and *paltE*, which shares 43.9–59.7% sequence identities with previously characterized alkyl salicylaldehyde-forming enzymes (Table S3). Expression of the four genes in *A. nidulans* LO8030 (ref. 17) resulted in a transformant RG104 with 2, 6, and 8 as the predominant metabolites and 3 as a minor peak, which are absent in the control strain ZL132 (Fig. 3(i), (v) and S7B). The integrity of compounds 2, 3, and 6 was confirmed by comparison of their retention times, UV spectra, and MS data with those from WT (Fig. 2B(i)). After isolation, 8 was identified as a diastereomer of 4 (Fig. 2C). Overall, no detectable aromatic metabolites were observed, suggesting that the homologous alkyl salicylaldehyde-forming enzymes in (+)-palitantin biosynthesis may function through a biosynthetic logic distinct from previously characterized pathways.^5–11^

**Fig. 3:**
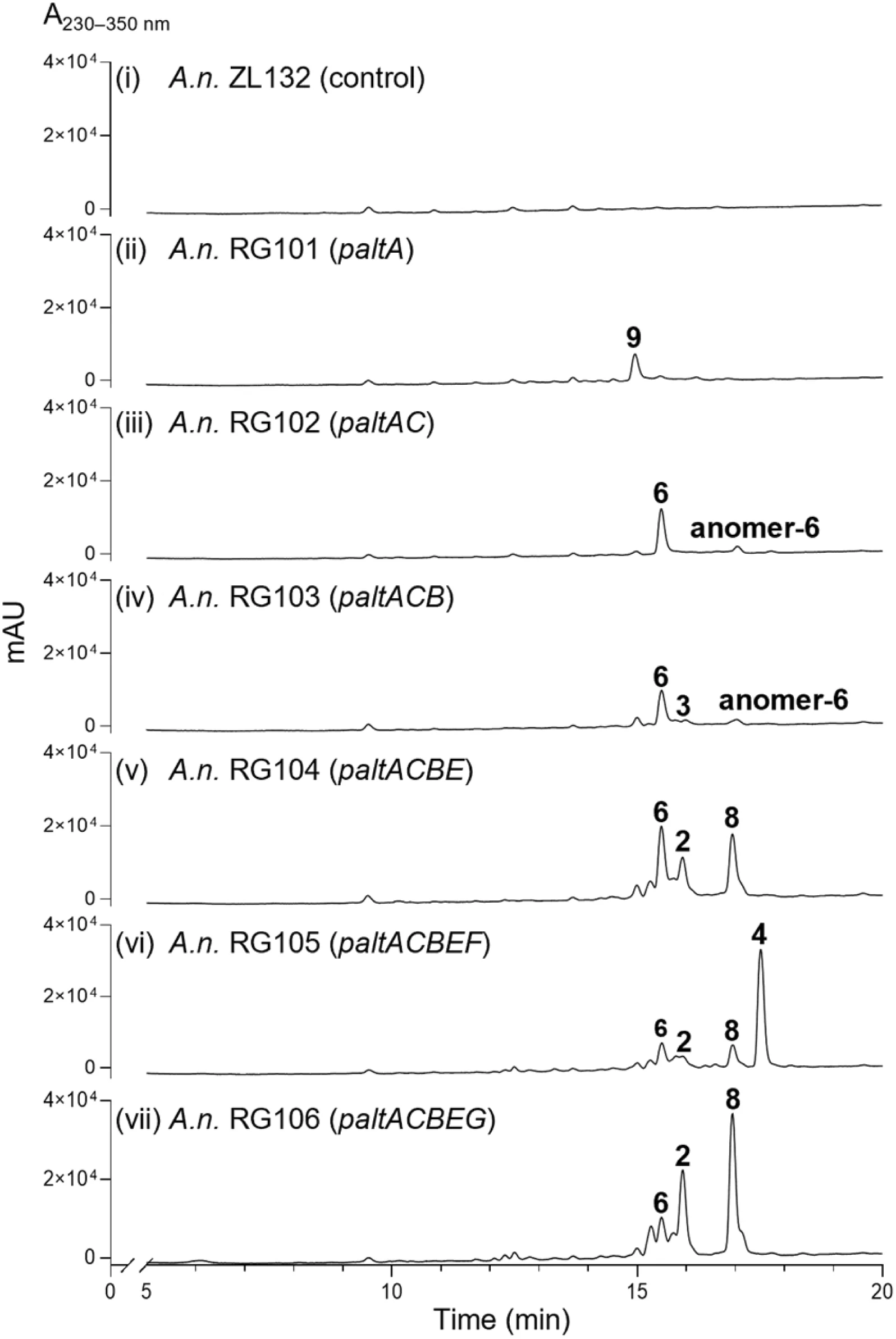
Metabolite analysis of *A. nidulans* transformants by using LC-MS method B with UV-detection at 230–350 nm (i–vii). The overlapping peak corresponding to 2 and 3 was resolved by EIC analysis (Fig. S7B).

We examined afterward the HR-PKS PaltA, which shares about 40% sequence identity with StrA from *Stachybotrys* sp. and VirA from *Trichoderma virens* (Table S3). Overexpression of *paltA* in *A. nidulans* RG101 resulted in the formation of a predominant peak 9 (Fig. 3(ii) and S4). Isolation and structural elucidation revealed that 9, similar to 6, exists as a mixture of two hemiacetalic tetrahydropyrans in the NMR solvent DMSO-*d*_6_ (Scheme 1). The putative SDR PaltC shares 48.0% sequence identity with VirB, which was reported to catalyze the first dehydrogenation of the PKS product (Table S3). Deletion of *paltC* in the WT strain led to the accumulation of trace amounts of 9 (Fig. 2B(iv)), indicating that PaltC is involved in the downstream transformation of the PKS product. To further investigate the role of PaltC, we coexpressed *paltA* and *paltC* in *A. nidulans* RG102. In comparison to the *paltA*-overexpression strain RG101 with 9 as the sole product (Fig. 3(ii)), the coexpression strain RG102 produced 6 as the dominant metabolite, while the amount of 9 was strongly reduced (Fig. 3(iii)). These results confirmed that PaltC catalyzes the conversion of 9 to 6 by oxidation of the C7 hydroxyl group (Scheme 1). We then turned to the second SDR PaltB, which shares 57.4% sequence identity with VirD, a homolog responsible for the second dehydrogenation of the PKS product (Table S3). In the Δ*paltB* mutant, 6 was detected as the predominant peak at 15.7 min and a minor peak at 17.2 min (Fig. 2B(v)), suggesting that PaltB functions downstream of PaltC in the pathway. Consistent with this hypothesis, expression of *paltABC* in *A. nidulans* RG103 led to the decrease of 6, accompanied by the appearance of 3 with the cyclohexenone ring and a carboxyl group at C1 as a minor peak (Fig. 3(iv) and S7B), supporting PaltB in 6 oxidation and probably cyclization.

To test this hypothesis, we overproduced PaltB in *Escherichia coli* and incubated it with 6 in the presence of either NAD^+^ or NADP^+^. As shown in Fig. 4A and S9A, 6 was converted to a new peak 10 with a clear preference for NAD^+^. Due to the instability, attempt to elucidate the structure of 10 by NMR analysis was failed. As shown in Fig. S10, the isolated peak 10 was rapidly converted to 3 as monitored with LC-MS analysis, even in the collected fraction stored on ice. However, HRMS analysis supported its molecular formula to be C_14_H_18_O_3_, with a [M + Na]^+^ ion at *m*/*z* 257.1141 (Fig. S20). Together with its spontaneous conversion to 3, these data suggest that 10 is the corresponding aldehyde of 3 (Scheme 1). Accordingly, we reasoned that 3 in both WT and RG103 (*paltACB*) is an artificial nonenzymatic product of the instable aldehyde 10.

**Fig. 4:**
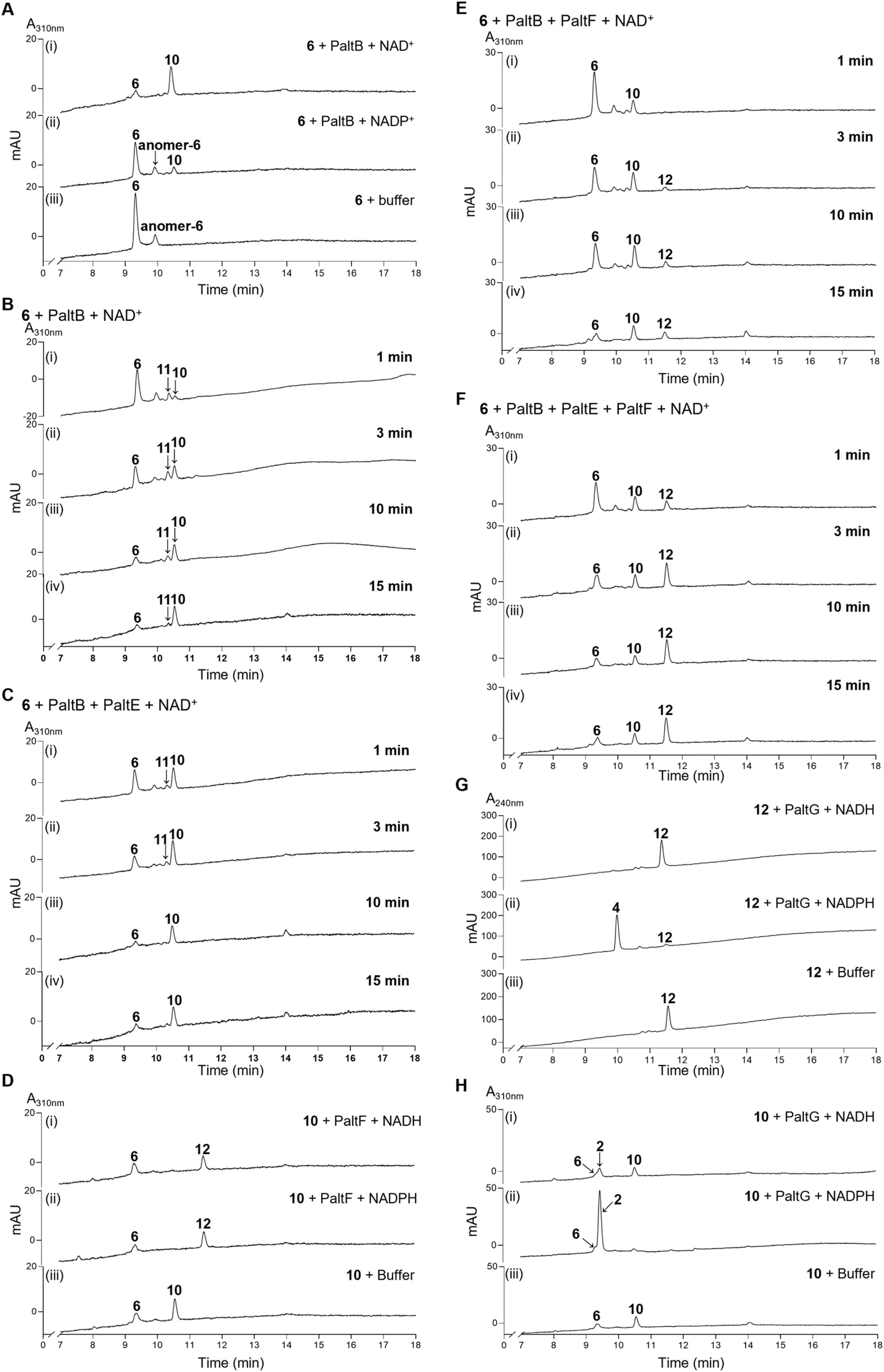
Analysis of *in vitro* enzyme assays of PaltB, PaltE, PaltF, and PaltG by using LC-MS method A. (Ai–iii) PaltB with 6; (Bi–iv) time-dependent analysis of PaltB with 6; (Ci–iv) one-pot time-dependent analysis of PaltB and PaltE with 6; (Di–iii) PaltF with 10 extracted from the quenched reaction shown in (A); (Ei–iv) one-pot time-dependent analysis of PaltB and PaltF with 6; (Fi–iv) one-pot time-dependent analysis of PaltB, PaltF, and PaltE with 6. (Gi–iii) PaltG with 12; (Hi–iii) PaltG with 10.

Based on the previous studies,^5,9^ we proposed that PaltB catalyzes the oxidation of 3-OH in 6 to a postulated diketo aldehyde 11, which likely undergoes spontaneous cyclization to 10 through aldol condensation followed by dehydration (Scheme 1). To probe the existence of 11, we performed a time-dependent PaltB-catalyzed conversion of 6. A peak with the expected mass for 11 was detected after incubation for 1 min, increased slightly at 3 min, and subsequently decreased, while 10 accumulated over time and became the predominant product after 15 min (Fig. 4B and S11). These observations are consistent with the rapid conversion of 11 to 10. In analogy to the previously reported,^5^ phenylhydrazine was used to catch the instable intermediates in the reaction mixture of 6 with PaltB. LC-MS analysis revealed the presence of peaks with masses corresponding to those of phenylhydrazone derivatives of 10 and 11 (Fig. S12).

Cupin domain-containing proteins display diverse functions in known alkyl salicylaldehyde pathways. In trichoxide biosynthesis, VirC had previously been suggested to affect product formation, whereas its direct catalytic role in backbone construction had not been experimentally established.^5^ The aromatase function of the homologous cupin protein StrC was later confirmed by *in vitro* enzymatic characterization in the study of the stachysalicyloid C pathway.^9,18^ In contrast, propyl salicylaldehyde formation proceeds efficiently in the absence of the cupin protein PsaCP.^10^ To clarify the role of the cupin protein PaltE in the (+)-palitantin biosynthesis, we first deleted *paltE* in the WT strain. The resulting Δ*paltE* mutant RG06 completely lost the ability to produce (+)-palitantin (1). Instead, 3, 6, and 7 were accumulated, but with strongly reduced intensities (Fig. 2B(vi) and S7A). It could be speculated that PaltE is involved in the cyclization of the PaltA product after two oxidation steps by PaltC and PaltB. This conclusion is supported by the coexpression of *paltE* with *paltA* in RG107, *paltAC* in RG108, and *paltACB* in RG104. As shown in Fig. S13, metabolite profile changes have been observed neither in RG107 nor in RG108. In contrast, coexpression of *paltE* with *paltACB* in strain RG104 led to the detection of metabolites 2 and 8 (Fig. 3(v) and S7B).

To further investigate the proposed role of PaltE, overproduced PaltE was first incubated with 6. However, no detectable conversion was observed (Fig. S9B(i)). PaltE was then added to the PaltB-catalyzed reaction for a time-dependent analysis. In comparison to the incubation mixture with PaltB alone (Fig. 4B), 11 accumulated to a lower level at the early time points and was not detected after prolonged incubation, which was also confirmed by detection of a phenylhydrazone derivative of 11 only in the early reaction stages (Fig. S12).

Correspondingly, the formation of 10 is more rapid with than without PaltE, already from beginning of the incubation (Fig. 4C). These observations support the proposal that PaltE promotes rapid and efficient conversion of 11 to 10, thereby facilitating the downstream pathway processing. In the Δ*paltE* mutant, inefficient conversion likely diverts part of the intermediate 11 toward formation of 7 through endogenous reduction of the C1 aldehyde group, while the limited amount of instable 10 spontaneously converts to 3 rather than supporting downstream transformations toward (+)-palitantin (Scheme 1). Likewise, coexpression of *paltE* with *paltACB* seems to enable sufficient formation of 10 for further transformation to 2 and 8.

Taken together, our results demonstrate that the early biosynthetic steps of (+)-palitantin are mediated by homologs of alkyl salicylaldehyde-forming enzymes with two sequential SDR-catalyzed dehydrogenation steps of the HRPKS product. However, differing from previously characterized pathways using cyclohexenone intermediates for spontaneous or cupin-assisted dehydration to aromatic metabolites, the cupin-domain containing protein PaltE promotes the formation of the instable cyclohexenone 10. Although 10 was readily converted to 3, its cyclohexenone scaffold remains intact without dehydration toward aromatization. In addition, a metabolite with an aromatic scaffold was detected neither in *A. nidulans* transformants nor in enzyme assays, indicating the non-aromatic feature of the product of the alkyl salicylaldehyde-forming enzyme homologs in the (+)-palitantin biosynthesis. PaltE shares 47.2–53.9% sequence identities with reported alkyl salicylaldehyde pathway-associated cupins (Table S3) with different functions. Phylogenetic analysis revealed no clear clustering of PaltE with these proteins (Fig. S14).

### Coordinated PaltB, PaltE, and PaltF action directs productive flux toward (+)-palitantin

To investigate the metabolism of 10 in the (+)-palitantin pathway, we first introduced the downstream candidate gene *paltF* into *A. nidulans* RG104 (*paltACBE*). The resulting transformant RG105 (*paltACBEF*) predominantly produced 4 (Fig. 3(vi)), while its diastereomer 8 was markedly reduced. The formation of 4, which possesses the C1/C6 stereochemistry required for (+)-palitantin biosynthesis, suggests that PaltF may catalyze the stereospecific reduction of the double-bond between C1 and C6. The minor diastereomer 8 is likely formed by an endogenous reductase from *A. nidulans*. Conversion of 10 to 4 involves two reduction steps: ene reduction catalyzed by PaltF and aldehyde reduction by very likely PaltG or a host enzyme. To address the reaction order, we deleted *paltF* in the WT strain. The resulting mutant produced 2, 3, 6, and 7, whereas 1, 4, and 5 were absent (Fig. 2B(vii)). EIC analysis revealed increased accumulation of 3 (Fig. S7A). This indirectly suggested that 10, the precursor of the shunt pathway product 3, is the substrate of PaltF. We therefore tested PaltF *in vitro* with three structurally related unsaturated intermediates differing in the C14 oxidation state: the alcohol 2, the carboxylic acid 3, and the aldehyde 10, in the presence of NADH or NADPH. Incubation of PaltF with 2 or 3 did not lead to detectable product formation, indicating that the alcohol and acid congeners are not accepted by PaltF as substrates (Fig. S9D and S9E). In contrast, when the instable aldehyde intermediate 10 was generated enzymatically from 6 by PaltB, the reaction mixture extracted with dichloromethane and evaporated to dryness, and immediately incubated with PaltF without isolation, a new product 12 was detected (Fig. 4D). To exclude its direct conversion to 12, 6 was incubated with PaltF alone in the presence of NADH and NADPH. As shown in Fig. S9C(i), (ii) and F, 12 was detected neither in this nor in other incubations with heat-inactivated proteins. Isolation and structural elucidation confirmed that the double bond between C1 and C6 of 10 has disappeared in 12, which exists as an enol form in CDCl_3_ (solvent for NMR measurement, Scheme 1 and Fig. S66–S71). Similar conversion yields were observed with both NADH and NADPH, suggesting that PaltF can utilize both cofactors effectively. The *in vitro* conversion of 10 to 12 under flavin-free conditions, together with bioinformatic analysis of no identifiable FMN/FAD-binding domain, supports PaltF as an apparently flavin-independent ene reductase.

Having established 10 as the direct substrate of PaltF, we next used a one-pot coupled assay to mimic the *in vivo* pathway. Incubation of 6 with PaltB, PaltF, and NAD^+^ led to the formation of 12 (Fig. 4E). In this coupled assay, PaltB reaction-generated NADH serves as reducing agent for the subsequent PaltF-catalyzed ene reduction. To reconstitute a more complete enzymatic cascade, PaltE was further included. Time-course analysis showed that the addition of PaltE markedly enhanced both the early formation and overall accumulation of 12, which was detectable as early as 1 min (Fig. 4F). Taken together, PaltE-enhanced supply of 10 and efficient NADH recycling from the PaltB to the PaltF reaction likely favor the rapid consumption of this instable intermediate, minimizing its diversion into shunt pathways and directing productive flux toward (+)-palitantin.

### PaltG-mediated aldehyde reduction supports main pathway progression

Since PaltF catalyzes the stereospecific ene reduction of 10 to 12, we postulated that the subsequent aldehyde reduction leading to 4 is mediated by the third SDR enzyme PaltG, a VirG homolog known to catalyze a similar transformation in a related pathway (Table S3).^5^ To test this hypothesis, recombinant PaltG was purified and incubated with 12 in the presence of either NADH or NADPH. As shown in Fig. 4G and S9G, PaltG catalyzes the reduction of 12 to 4 and exhibits a strong preference for NADPH, proving its role in aldehyde reduction.

In the WT strain, a small amount of 5, the C1 epimer of 4, was detected. We propose that 5 may arise from PaltG-catalyzed reduction of 14, a tautomerism of 12 (Scheme 1). The predominant formation of the main pathway intermediate 4 in the PaltG reaction may be attributed to the tautomeric equilibrium favoring 12 over 14. Alternatively, 5 may be formed by epimerization of 4*via* keto–enol tautomerism. During storage, even at −20 °C, 4 and 5 changed to each other (Scheme 1). To further examine the role of PaltG in the native producer, we deleted *paltG* in the WT strain. In the resulting Δ*paltG* mutant *P. palitans* RG08, its productivity for (+)-palitantin congeners is strongly reduced. Detection of a minor peak corresponding to (+)-palitantin (1) (Fig. 2B(viii)) implies that the WT strain could contain a host-derived PaltG-like enzyme capable of catalyzing the same aldehyde reduction, as reported for other pathways.^11^ Differing from *P. palitans* WT, compound 2 was not detected in the Δ*paltG* mutant. This suggests that the formation of 2 requires PaltG for the reduction of the aldehyde function in 10. Indeed, 10 was converted by the recombinant PaltG to 2 in the presence of NADH or NADPH (Fig. 4H), whereas no formation of 2 was observed in the reaction mixture of 6 with PaltG and in other negative controls with boiled proteins (Fig. S9C(iii), (iv) and H). Again, NADPH acted as a much better cofactor than NADH.

In *A. nidulans* RG104 (*paltACBE*), compounds 2 and 8 were produced (Fig. 3(v)), suggesting ene reduction and aldehyde reduction by endogenous enzymes. Coexpression of *paltG* with *paltACBE* in RG106 (*paltACBEG*) markedly increased the production of both compounds (Fig. 3(vii)), further supporting the role of PaltG as an aldehyde reductase that promotes the formation of 2 from 10 and 8 from 13, the host-mediated ene reduction product of 10 (Scheme 1). Bioinformatic analysis identified a putative short-chain dehydrogenase XP_681854.1 in the genome of *A. nidulans*, sharing a sequence identity of 65.7% with PaltG and representing a potential candidate for the endogenous aldehyde-reduction activity. Taken together, these data support that PaltG functions as a flexible aldehyde reductase. It accepts 12 as a substrate to produce 4 in the main pathway and also reduces other C-14 aldehyde-containing analogues arising from shunt pathways or artificial pathways reconstructed in *A. nidulans*.

### The P450 enzyme PaltD catalyzes a stereospecific C3 hydroxylation and completes the (+)-palitantin biosynthesis

To complete the conversion of 4 to (+)-palitantin (1), a stereoselective C3 hydroxylation is required. Deletion of *paltD* in *P. palitans* WT strain completely abolished 1 production, with marked accumulation of 4 and a slight increase in 5 (Fig. 2B(ix)). All the detected products 2–5 in Δ*paltD* mutant lack the hydroxyl group at C3, proving PaltD for the C3 hydroxylation unequivocally. Feeding 4 and 5 to the *paltD* overexpression strain *A. nidulans* RG109 resulted in conversion of 4 to 1 (Fig. 5A(ii)), while 5 was not consumed (Fig. 5A(iii)). Consistently, assays with microsomal fraction from *A. nidulans* RG109 revealed the conversion of 4 to 1, as evidenced by extracted ion chromatograms. No product was detected with 5 as substrate under the same conditions (Fig. S17).

**Fig. 5:**
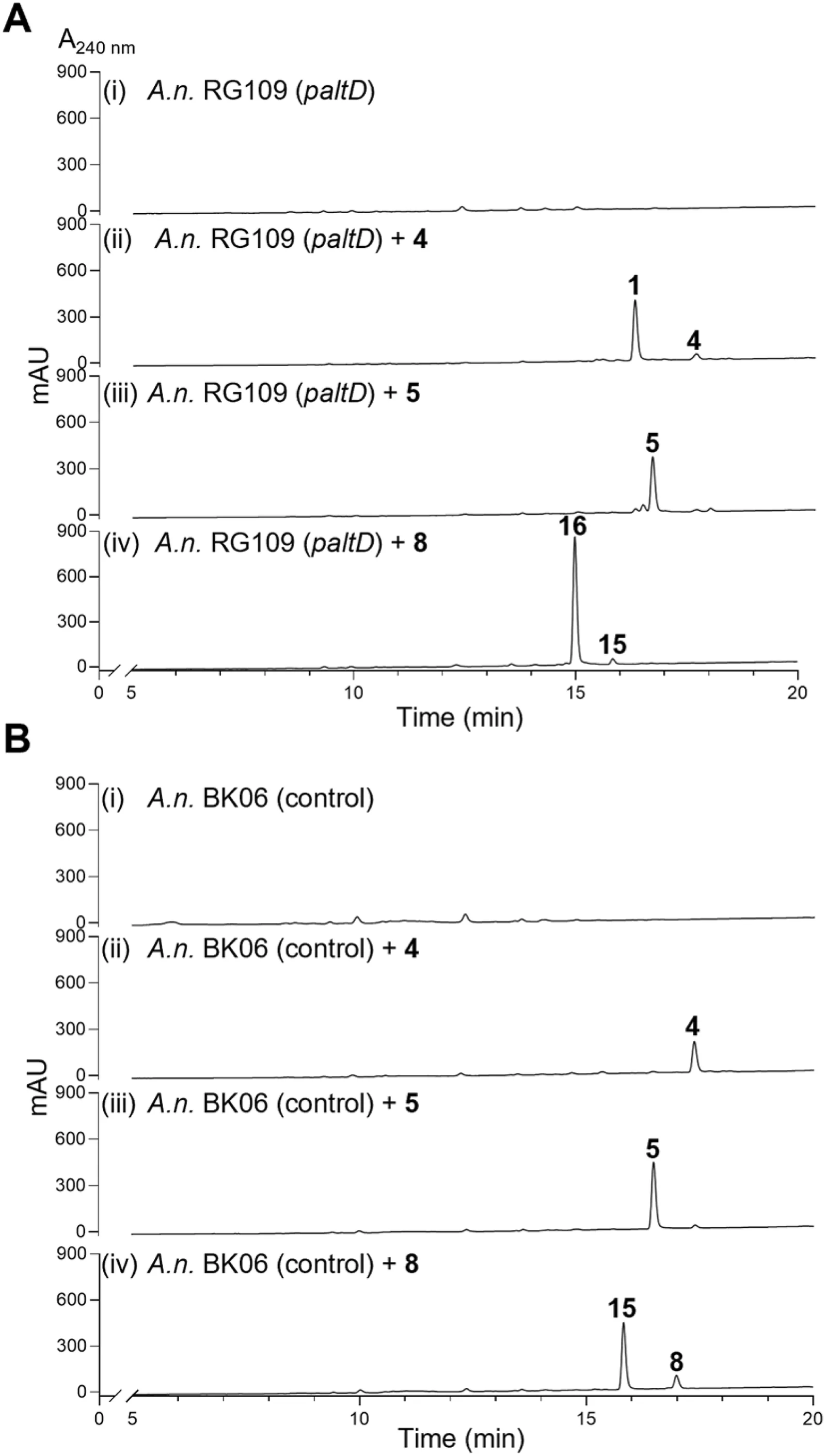
Analysis of culture extracts from *A. nidulans* RG109 harboring *paltD* (Ai–iv) and the control strain (Bi–iv), with or without feeding of 4, 5 and 8 by using method B.

To further examine the substrate selectivity of PaltD, another stereoisomer 8 of 4, was fed to the control strain *A. nidulans* BK06 and the *paltD*-expressing strain RG109. In BK06, 8 was converted to a new product 15 (Fig. 5B(iv)), whose HRMS data indicated the incorporation of two additional hydrogen atoms (Fig. S20). In contrast, RG109 almost completely converted 8 to 16, with 15 as a minor product (Fig. 5A(iv)). Structural elucidation of 16 revealed C3 hydroxylation accompanied by reduction of the C2 ketone to a hydroxy group, suggesting that 15 is generated by host-mediated C2 ketone reduction of 8 and then further hydroxylated by PaltD to afford 16 (Scheme 1). These results proved that PaltD accepts 4 and 15, but not 5, suggesting a preference for substrates with a *trans* C1/C6 relationship.

## Conclusions

In summary, we elucidated the complete biosynthetic pathway of (+)-palitantin (Scheme 1) and demonstrated that homologs of alkyl salicylaldehyde-forming enzymes can be employed in a non-aromatic biosynthetic route. Unlike previously characterized alkyl salicylaldehyde pathways, in which related cyclohexenone intermediates undergo spontaneous or cupin-assisted dehydration to form aromatic products, the cupin protein PaltE enhances the formation of the instable cyclohexenone 10 for further enzymatic modification. This intermediate undergoes a defined sequence of stereo controlled transformations to assemble the stereochemically complex cyclohexanone scaffold of (+)-palitantin. *In vitro* reconstitution demonstrated that the coordinated action of PaltB, PaltE, and PaltF enables efficient generation and stereospecific ene reduction of 10, thereby minimizing shunt product formation and channeling pathway flux toward (+)-palitantin. Subsequent aldehyde reduction by the SDR PaltG and stereospecific hydroxylation by the cytochrome P450 enzyme PaltD complete the late-stage scaffold modifications. This work expands the functional landscape of alkyl salicylaldehyde-forming enzymes, highlighting a programmed strategy for non-aromatic scaffold construction and stereochemical control in fungal polyketide biosynthesis.

## Author contributions

S. M. L., R. G., and Z. X. Z. conceived this project. All authors contributed to the design of the study methods and analysis of results. R. G., Z. X. Z, L. Y. and L. D. P. generated the experimental data. The manuscript was written with all author's contributions.

## Conflicts of interest

The authors declare no competing interests.

## Supplementary Material

SC-017-D6SC04815C-s001

## Data Availability

The supporting data are available within the article and its supplementary information (SI) file. Supplementary information: experimental details, gene and gene cluster information, DNA and protein sequences, primers and plasmids, SDS-PAGE analysis, LC-MS data, NMR, and UV spectra. See DOI: https://doi.org/10.1039/d6sc04815c.

## References

[cit1] Abdullah M. N., Osw P., Hassan S. A., Othman S. (2024). J. Mol. Struct..

[cit2] Birkinshaw J. H., Raistrick H. (1936). Biochem. J..

[cit3] Huang X.-S., Yang B., Sun X.-F., Xia G.-P., Liu Y.-Y., Ma L., She Z.-G. (2014). Helv. Chim. Acta.

[cit4] Sivakrishna B., Sahoo S., Kumar A., Pal S. (2022). ChemistrySelect.

[cit5] Liu L., Tang M. C., Tang Y. (2019). J. Am. Chem. Soc..

[cit6] Zhao Z., Ying Y., Hung Y. S., Tang Y. (2019). J. Nat. Prod..

[cit7] Nies J., Ran H., Wohlgemuth V., Yin W. B., Li S.-M. (2020). Org. Lett..

[cit8] Xiang P., Kemmerich B., Yang L., Li S.-M. (2022). Org. Lett..

[cit9] Yang R., Feng J., Xiang H., Cheng B., Shao L.-D., Li Y.-P., Wang H., Hu Q.-F., Xiao W.-L., Matsuda Y., Wang W.-G. (2023). J. Am. Chem. Soc..

[cit10] Peter M., Li S.-M. (2024). Chem. Commun..

[cit11] Li Z.-H., Dai Y., Zhou J., Yang L., Li S.-M. (2025). Org. Lett..

[cit12] Wang S.-Y., Chiu K.-W., Lin K.-L., Wei H.-Y., Chen Y.-R., Tu Z., Lin Y.-T., Lin C.-H., Chein R.-J., Lin H.-C. (2025). J. Am. Chem. Soc..

[cit13] Chaplen P., Thomas R. (1960). Biochem. J..

[cit14] Hareau G. P.-J., Koiwa M., Hikichi S., Sato F. (1999). J. Am. Chem. Soc..

[cit15] Zhang Z.-X., Li Z.-H., Yin W.-B., Li S.-M. (2022). Org. Lett..

[cit16] Goswami R. S. (2012). Methods Mol. Biol..

[cit17] Chiang Y. M., Ahuja M., Oakley C. E., Entwistle R., Asokan A., Zutz C., Wang C. C., Oakley B. R. (2016). Angew. Chem., Int. Ed..

[cit18] Wang H., Peng C., Chen X.-X., Wang H.-Y., Yang R., Xiang H., Hu Q.-F., Liu L., Chung L. W., Matsuda Y., Wang W.-G. (2024). ACS Catal..

