# Supplementary material for: Biosynthesis of (+)-palitantin requires homologs of alkyl salicylaldehyde-forming enzymes without involvement of an aromatic intermediate
Source: Chem Sci. 2026 Aug 7;17(38):18370–8. doi: 10.1039/d6sc04815c (PMC13463297; doi:10.1039/d6sc04815c)
Supplement: Supplementary file 1 [file SC-017-D6SC04815C-s001.pdf]

Supplementary Information (SI) for Chemical Science.
This journal is © The Royal Society of Chemistry 2026

Supporting Information

Biosynthesis of (+)-Palitantin Requires Homologs of Alkyl Salicylaldehyde-Forming Enzymes without Involvement of an Aromatic Intermediate

Rui Ge, [†a] Zheng-Xi Zhang, [†a] Li Yang,[b] Li-Dan Pang,[a] and Shu-Ming Li[*a]

[a] Philipps-Universität Marburg, Fachbereich Pharmazie, Institut für Pharmazeutische Biologie und Biotechnologie, Robert-Koch-Straße 4, 35037 Marburg, Germany

[b] Haikou Key Laboratory for Research and Utilization of Tropical Natural Products, Institute of Tropical Bioscience and Biotechnology, Chinese Academy of Tropical Agricultural Sciences, Haikou 571101, P. R. China

[†]These authors contributed equally to this work

*Corresponding author. Email: shuming.li@staff.uni-marburg.de

Table of Contents

Experimental Procedures **7**

1. Media, strains, and growth conditions. 7

2. Genomic DNA and RNA isolation and cDNA synthesis. 7

3. Genome sequencing and sequence analysis. 7

4. PCR amplification, gene cloning, and plasmid construction. 7

5. Genetic manipulation of *P. palitans* NRRL 2033. 7

6. Heterologous expression in *A. nidulans*. 7

7. Precursor feeding in *A. nidulans* RG109 (*paltD*) and the control strain BK06. 8

8. Overproduction and purification of PaltB, PaltE, PaltF, and PaltG. 8

9. *In vitro* assays of PaltB and PaltE with 6. 8

10. *In vitro* assays of PaltF and PaltG with 10. 8

11. *In vitro* assays of PaltB, PaltE, and PaltF with 6. 8

12. *In vitro* assays of PaltG with 12. 9

13. Microsome preparation and *in vitro* assays of 4 and 5 with PaltD. 9

14. HPLC and LC-MS analysis of secondary metabolites. 9

15. NMR analysis. 9

16. Measurement of optical rotations. 9

17. The experimental and calculated electronic circular dichroism (ECD) spectra. 9

18. Large-scale fermentation, extraction, and isolation of secondary metabolites. 10

19. Structural elucidation. 10

20. Physicochemical properties of the compounds described in this study. 12

Supplementary Tables **14**

Table S1. Proteins encoded by the *palt* cluster in *Penicillium palitans* NRRL 2033. 14

Table S2. Sequence identities of the *palt* cluster from *Penicillium palitans* NRRL 2033 with those of different *P. palitans* strains. 15

Table S3. Sequence identities of proteins encoded by the *palt* cluster from *Penicillium palitans* NRRL 2033 with those of known clusters. 16

Table S4. Strains used and created in this study. 17

Table S5. Plasmids used and constructed in this study. 18

Table S6. Primers used in this study. 20

Table S7. NMR data of (+)-palitantin (**1**) in $CDCl_3$. 25

Table S8. NMR data of dehydropenihydrone A (**2**) in DMSO-d_6. 26

Table S9. NMR data of dehydropenihydrone B (**3**) in DMSO-d_6. 27

Table S10. NMR data of penihydrone (**4**) in acetone-d_6. 28

Table S11. NMR data of penihydrone B (**5**) in acetone-d_6. 29

Table S12. NMR data of palitanol A (**6**) in DMSO-d_6. 30

Table S13. NMR data of palitanol B (**7**) in DMSO-d_6. 31

Table S14. NMR data of penihydrone C (**8**) in acetone-d_6. 32

Table S15. NMR data of palitanol C (**9**) in DMSO-d_6. 33

Table S16. NMR data of penihydronal (**12**) in $CDCl_3$. 34

Table S17. NMR data of dia-palitantol (**16**) in DMSO-d_6. 35

Supplementary Figures **36**

Figure S1. Reported biosynthetic gene clusters for fungal alkyl salicylaldehyde derivatives and the aromatic salicylaldehyde cores formed by a single HR-PKS, two SDRs, with or without a cupin protein. 36

Figure S2. Schematic illustration of target gene deletion and PCR verification of Δ*paltA*–Δ*paltE* mutant strains in *P. palitans*. 37

Figure S3. Schematic illustration of target gene deletion and PCR verification of Δ*paltF*, Δ*paltG*, Δ*paltI*, and Δ*paltJ* mutant strains in *P. palitans*. 38

Figure S4. Heterologous expression and PCR verification for *A. nidulans* strains. 39

Figure S5. HPLC analysis of metabolites in WT strain and Δ*paltJ* mutant RG03. 40

Figure S6. SDS-PAGE (12%) analysis of purified recombinant N-terminally His_6-tagged PaltB, PaltE, PaltF, and PaltG. 41

Figure S7. Extracted ion chromatograms of the overlapping peaks assigned to **2** and **3** in *P. palitans* strains (A) and *A. nidulans* transformants (B). 42

Figure S8. HPLC analysis of **9** before (i) and after evaporation (ii). 43

Figure S9. HPLC analysis of control assays with different enzymes. 44

Figure S10. LC-MS analysis of the conversion of the isolated **10** to **3** over time. 45

Figure S11. Extracted ion chromatograms of **10** and **11** in the incubation mixture of **6** with PaltB (A) and with PaltB and PaltE (B). 46

Figure S12. (A) Extracted ion chromatograms of the phenylhydrazone derivatives. The reactions were quenched at different time points and followed by incubation with phenylhydrazine for 10 min: **6** with PaltB alone (Ai) and with PaltB and PaltE together (Aii).

(B) The proposed mechanism of PaltB and PaltE-catalyzed reactions and the plausible phenylhydrazone derivatives. 47

Figure S13. HPLC analysis of metabolites in *A. nidulans* transformants. 48

Figure S14. Phylogenetic analysis of PaltE and alkyl salicylaldehyde pathway-associated cupins. 49

Figure S15. LC-MS analysis of the spontaneous aromatization of **3**. 50

Figure S16. LC-MS analysis of **12**. 51

Figure S17. Extracted ion chromatograms of *in vitro* assays of **4** and **5** with microsomes from *A. nidulans* with or without PaltD. 52

Figure S18. Experimental and calculated ECD spectra for **2**, **5**, and **8**. 53

Figure S19. UV spectra of compounds **1–10**, **15**, and **16**. 54

Figure S20. Mass spectra of compounds **1–10**, **12**, **15**, and **16**. 55

Figure S21. ^{1}H NMR spectrum of (+)-palitantin (**1**) in $CDCl_3$ (400 MHz). 56

Figure S22. ^{13}C NMR spectrum of (+)-palitantin (**1**) in $CDCl_3$ (100 MHz). 57

Figure S23. ^{1}H NMR spectrum of dehydropenihydrone A (**2**) in DMSO-d_6 (400 MHz). 58

Figure S24. ^{13}C NMR spectrum of dehydropenihydrone A (**2**) in DMSO-d_6 (100 MHz). 59

Figure S25. HSQC spectrum of dehydropenihydrone A (**2**) in DMSO-d_6. 60

Figure S26. ^{1}H-^{1}H COSY spectrum of dehydropenihydrone A (**2**) in DMSO-d_6. 61

Figure S27. HMBC spectrum of dehydropenihydrone A (**2**) in DMSO-d_6. 62

Figure S28. NOESY spectrum of dehydropenihydrone A (**2**) in DMSO-d_6. 63

Figure S29. ^{1}H NMR spectrum of dehydropenihydrone B (**3**) in DMSO-d_6 (500 MHz) immediately after isolation. 64

Figure S30. ^{1}H NMR spectrum of dehydropenihydrone B (**3**) after 7 days in DMSO-d_6 (500 MHz). 65

Figure S31. ^{13}C NMR spectrum of dehydropenihydrone B (**3**) in DMSO-d_6 (125 MHz). 66

Figure S32. HSQC spectrum of dehydropenihydrone B (**3**) in DMSO-d_6. 67

Figure S33. ^{1}H-^{1}H COSY spectrum of dehydropenihydrone B (**3**) in DMSO-d_6. 68

Figure S34. HMBC spectrum of dehydropenihydrone B (**3**) in DMSO-d_6. 69

Figure S35. ^{1}H NMR spectrum of penihydrone (**4**) in acetone-d_6 (500 MHz). 70

Figure S36. ^{13}C NMR spectrum of penihydrone (**4**) in acetone-d_6 (125 MHz). 71

Figure S37. ^{1}H NMR spectrum of penihydrone B (**5**) in acetone-d_6 (400 MHz). 72

Figure S38. ^{13}C NMR spectrum of penihydrone B (**5**) in acetone-d_6 (125 MHz). 73

Figure S39. HSQC spectrum of penihydrone B (**5**) in acetone-d_6. 74

Figure S40. ^{1}H-^{1}H COSY spectrum of penihydrone B (**5**) in acetone-d_6. 75
Figure S41. HMBC spectrum of penihydrone B (**5**) in acetone-d_6. 76
Figure S42. NOESY spectrum of penihydrone B (**5**) in acetone-d_6. 77
Figure S43. ^{1}H NMR spectrum of palitanol A (**6**) in DMSO-d_6 (500 MHz). Labelling without a or b means signals for both structures. 78
Figure S44. ^{13}C NMR spectrum of palitanol A (**6**) in DMSO-d_6 (125 MHz). 79
Figure S45. HSQC spectrum of palitanol A (**6**) in DMSO-d_6. 80
Figure S46. ^{1}H-^{1}H COSY spectrum of palitanol A (**6**) in DMSO-d_6. 81
Figure S47. HMBC spectrum of palitanol A (**6**) in DMSO-d_6. 82
Figure S48. NOESY spectrum of palitanol A (**6**) in DMSO-d_6. 83
Figure S49. ^{1}H NMR spectrum of palitanol B (**7**) in DMSO-d_6 (500 MHz). 84
Figure S50. ^{13}C NMR spectrum of palitanol B (**7**) in DMSO-d_6 (125 MHz). 85
Figure S51. HSQC spectrum of palitanol B (**7**) in DMSO-d_6. 86
Figure S52. ^{1}H-^{1}H COSY spectrum of palitanol B (**7**) in DMSO-d_6. 87
Figure S53. HMBC spectrum of palitanol B (**7**) in DMSO-d_6. 88
Figure S54. NOESY spectrum of palitanol B (**7**) in DMSO-d_6. 89
Figure S55. ^{1}H NMR spectrum of penihydrone C (**8**) in acetone-d_6 (400 MHz). 90
Figure S56. ^{13}C NMR spectrum of penihydrone C (**8**) in acetone-d_6 (125 MHz). 91
Figure S57. HSQC spectrum of penihydrone C (**8**) in acetone-d_6. 92
Figure S58. ^{1}H-^{1}H COSY spectrum of penihydrone C (**8**) in acetone-d_6. 93
Figure S59. HMBC spectrum of penihydrone C (**8**) in acetone-d_6. 94
Figure S60. NOESY spectrum of penihydrone C (**8**) in acetone-d_6. 95
Figure S61. ^{1}H NMR spectrum of palitanol C (**9**) in DMSO-d_6 (500 MHz). Labelling without a or b means signals for both structures. 96
Figure S62. ^{13}C NMR spectrum of palitanol C (**9**) in DMSO-d_6 (125 MHz). Labelling without a or b means signals for both structures. 97
Figure S63. HSQC spectrum of palitanol C (**9**) in DMSO-d_6. 98
Figure S64. ^{1}H-^{1}H COSY spectrum of palitanol C (**9**) in DMSO-d_6. 99
Figure S65. HMBC spectrum of palitanol C (**9**) in DMSO-d_6. 100
Figure S66. ^{1}H NMR spectrum of penihydronal (**12**) in $CDCl_3$ (500 MHz). 101
Figure S67. ^{13}C NMR spectrum of penihydronal (**12**) in $CDCl_3$ (125 MHz). 102
Figure S68. HSQC spectrum of penihydronal (**12**) in $CDCl_3$. 103

Figure S69. ^{1}H-^{1}H COSY spectrum of penihydronal (**12**) in $CDCl_3$. 104
Figure S70. HMBC spectrum of penihydronal (**12**) in $CDCl_3$. 105
Figure S71. NOESY spectrum of penihydronal (**12**) in $CDCl_3$. 106
Figure S72. ^{1}H NMR spectrum of dia-palitantol (**16**) in DMSO-d_6 (400 MHz). 107
Figure S73. ^{13}C NMR spectrum of dia-palitantol (**16**) in DMSO-d_6 (100 MHz). 108
Figure S74. HSQC spectrum of dia-palitantol (**16**) in DMSO-d_6. 109
Figure S75. ^{1}H-^{1}H COSY spectrum of dia-palitantol (**16**) in DMSO-d_6. 110
Figure S76. HMBC spectrum of dia-palitantol (**16**) in DMSO-d_6. 111
Figure S77. NOESY spectrum of dia-palitantol (**16**) in DMSO-d_6. 112

Supplementary References 113

Experimental Procedures

1. Media, strains, and growth conditions.

The fungal strains used and created in this study are summarized in Table S4. *Penicillium palitans* and *Aspergillus nidulans* strains were cultivated for sporulation according to previously described methods.[1] For secondary metabolite production and compound analysis, all strains were cultivated in PD liquid medium with appropriate supplements. *Escherichia coli* DH5α and BL21(DE3) were grown in Lysogeny Broth (LB) medium or on LB agar plates at 37 °C.[2,3] Recombinant *E. coli* strains were cultivated in the presence of ampicillin (100 μg/mL) or kanamycin (50 μg/mL).

2. Genomic DNA and RNA isolation and cDNA synthesis.

A. nidulans and *P. palitans* strains were cultivated in liquid PD medium for 2–3 days for genomic DNA isolation, whereas *P. palitans* NRRL 2033 was cultivated at 25 °C for 7 days for RNA isolation and cDNA synthesis. Mycelia were collected and transferred to 2 mL screw-cap vials, and subsequent genomic DNA and RNA isolation and cDNA synthesis were performed as previously described.[4]

3. Genome sequencing and sequence analysis.

The genome of *P. palitans* NRRL 2033 was sequenced by BioMarker (Beijing, China).[5] Initial identification and analysis of the (+)-palitantin biosynthetic gene cluster were performed using the online tools antiSMASH, 2ndFind (https://biosyn.nih.go.jp/2ndfind/), and the NCBI BLAST tool.[6] The genes in the (+)-palitantin cluster were named as *paltA* – *J*. The genomic DNA sequence of the (+)-palitantin cluster from *P. palitans* NRRL 2033 reported in this study is available in GenBank under the accession number PZ404400 (Table S1, Figure 2A).

4. PCR amplification, gene cloning, and plasmid construction.

Plasmids generated and used in this study are listed in Table S5, and primers are provided in Table S6. PCR amplification was performed using Phusion High-Fidelity DNA polymerase (NEB) on a T100 Thermal Cycler (Bio-Rad). All plasmids were constructed *via* homologous recombination in *E. coli* DH5α.[7]

5. Genetic manipulation of *P. palitans* NRRL 2033.

Protoplast preparation and PEG-mediated transformation of *P. palitans* NRRL 2033 were performed according to the protocol described before,[4] with the following modifications. Fresh spores were inoculated into 50 mL PD medium in a 250 mL flask and incubated at 25 °C with shaking at 150 rpm for 9 h to obtain germlings. For cell wall digestion, germlings were treated in 10 mL osmotic buffer supplemented with 20 mg yatalase (*Corynebacterium* sp. OZ-21), 25 mg lysing enzyme (*Aspergillus* sp.), 25 mg driselase (*Basidiomycetes* sp.), and 40 μL chitinase (*Trichoderma harzianum*), and incubated at 30 °C with shaking at 100 rpm for 6 h. Diagnostic PCR amplification was used to verify the resulting deletion mutants (Figures S2 and S3).

6. Heterologous expression in *A. nidulans*.

A. nidulans LO8030 was used as the heterologous host. Protoplast preparation and transformation were carried out as previously described.[8] Target genes were amplified from *P.*

palitans NRRL 2033 genomic DNA and cloned into pJN017 or pZL132 for chromosomal integration, or into the replicative vectors pZL69 or pZL70 for episomal expression.[9–11] Plasmids were introduced individually or in combination to generate the desired recombinant strains (Table S4). Transformants were selected using auxotrophic markers and verified by PCR (Figure S4).

7. Precursor feeding in *A. nidulans* RG109 (*paltD*) and the control strain BK06.

Precursors **4**, **5**, and **8** were dissolved in DMSO to prepare 50 mM stock solutions. The mutant strain RG109 and the control strain BK06 were first cultivated in 5 mL of PD liquid medium for 2 days. Subsequently, 2 µL of the stock solutions were added to the cultures to achieve final concentrations of about 20 µM. After further incubation at 25 °C for 7 days, secondary metabolites were extracted twice with EtOAc, dissolved in MeOH, and analyzed by LC-MS using method B.

8. Overproduction and purification of PaltB, PaltE, PaltF, and PaltG.

Intron-free coding sequences of *paltB*, *paltE*, *paltF*, and *paltG* were amplified from *P. palitans* NRRL 2033 cDNA and cloned into pET28a(+) to generate constructs for production of N-terminal His_6-tagged proteins.[4] The resulting plasmids pRG25–pRG28 were individually transformed into *E. coli* BL21(DE3). Protein overproduction was carried out in Terrific Broth (2.4% yeast extract, 2.0% tryptone, 0.4% glycerol, 0.1 M phosphate buffer, pH 7.4) at 37 °C to an absorption of 0.6–0.8 at 600 nm, followed by induction with 0.1 mM IPTG and incubation at 16 °C for 20 h. For PaltB production, 10 mM lactose was used instead of IPTG. Recombinant proteins were purified by Ni-NTA affinity chromatography (Qiagen, Hilden). PaltB was further purified using cobalt affinity resin. The purified proteins were analyzed on a sodium dodecyl sulfate-polyacrylamide gel (12%, w/v) (Figure S6).

9. *In vitro* assays of PaltB and PaltE with 6.

Enzyme assays (50 µL) were performed in 20 mM phosphate buffer (pH 7.4) containing 2 mM NAD^+ or $NADP^+$, 0.5 mM **6**, and either PaltB (1.5 µM) alone or PaltB (1.5 µM) with PaltE (1.6 µM). Reactions were incubated at 30 °C for 15 min and terminated with 100 µL dichloromethane. The organic phase was collected, evaporated under reduced pressure, and the residues dissolved in 100 µL MeOH for LC-MS analysis using method A.

10. *In vitro* assays of PaltF and PaltG with 10.

Due to the chemical instability of **10**, it was produced by using PaltB (1.5 µM) in a 50 µL reaction mixture containing 20 mM phosphate buffer (pH 7.4), 0.5 mM **6**, and 2 mM NAD^+ at 30 °C for 30 min. The reaction was quenched with 100 µL dichloromethane, centrifuged, and the organic phase evaporated. Residues were dissolved in 50 µL reaction mixture containing phosphate buffer (20 mM, pH 7.4), NADH or NADPH (2 mM), and PaltF (1.7 µM) or PaltG (1.7 µM), incubated at 30 °C for 30 min, terminated with one volume of MeOH, centrifuged, and analyzed by LC-MS using method A.

11. *In vitro* assays of PaltB, PaltE, and PaltF with 6.

Enzyme assays (50 µL) were performed in 20 mM phosphate buffer (pH 7.4) containing 2 mM NAD+, 0.5 mM **6**, and either PaltB (1.5 µM) with PaltF (1.7 µM) or PaltB (1.5 µM) with PaltF

(1.7 µM) and PaltE (1.6 µM). Reactions were incubated at 30 °C for 30 min and terminated with one volume of MeOH, centrifuged, and analyzed by LC-MS using method A.

12. *In vitro* assays of PaltG with 12.

Enzyme assays (50 µL) contained 20 mM phosphate buffer (pH 7.4), 2 mM NADH or NADPH, 0.5 mM **12**, and PaltG (1.7 µM). Reaction mixtures were incubated at 30 °C for 30 min, terminated with 50 µL MeOH, centrifuged, and analyzed by LC-MS using method A.

13. Microsome preparation and *in vitro* assays of 4 and 5 with PaltD.

The *A. nidulans* transformant containing *paltD* was cultivated in 50 mL PD medium supplemented with uridine, uracil, and pyridoxine at 25 °C for 3 days. Microsomal fractions were prepared as described previously.[12] Compounds **4** and **5** (0.5 mM) were incubated with 100 µL of the PaltD microsomal fraction in the presence of NADPH (6 mM) at 25 °C for 2 h.The reaction mixtures were extracted with EtOAc, dried, redissolved in MeOH, and analyzed by LC-MS using method A.

14. HPLC and LC-MS analysis of secondary metabolites.

Semi-preparative HPLC was performed on an Agilent system with a VDSpher PUR 100 C18-M-SE column (250 × 10 mm, 5 µm) using H_2O and ACN or MeOH at 2.0 mL/min. Preparative HPLC was conducted on an Agilent system with a VDSpher PUR 100 C18-M-SE column (150 × 20 mm, 5 µm) using ACN/H_2O at 15.0 mL/min. LC-MS analysis was carried out on an Agilent system equipped with a microTOF-Q III spectrometer using an Agilent InfinityLab Poroshell 120 EC-C18 column (100 × 3 mm, 2.7 µm). For LC-MS analysis, a linear gradient of 5–100% ACN in H_2O containing 0.1% (v/v) HCOOH over 10 (method A) or 30 min (method B) was applied, followed by washing with 100% ACN for 5 min and re-equilibration with 5% ACN in H_2O for 5 min at 0.3 mL/min. Exact masses were determined in positive electrospray ionization mode. The capillary voltage and collision energy were set to 4.5 kV and 8.0 eV, respectively. Sodium formate was used for mass calibration in each run, and mass spectra were acquired over an m/z range of 100–1500.

15. NMR analysis.

NMR spectra of the isolated compounds were recorded on a JEOL ECA-500 or 400 MHz spectrometer (JEOL, Akishima, Tokyo, Japan). Samples were dissolved in $CDCl_3$, acetone-d_6, or DMSO-d_6. The δ_H and δ_C values are given in ppm.

16. Measurement of optical rotations.

Optical rotations were measured on a Jasco DIP-370 polarimeter at 20 °C using the sodium D-line (λ = 589.3 nm).

17. The experimental and calculated electronic circular dichroism (ECD) spectra.

Experimental CD spectra were recorded on a J-1500 CD spectrometer. Samples were dissolved in MeOH and measured in the range of 190–400 nm by using a 1 mm path-length quartz cuvette. Conformational search, geometry optimization, ECD calculations, and Boltzmann-weighted spectrum generation were performed according to previously described

methods.[10] Experimental and calculated ECD spectra are shown in Figure S18.

18. Large-scale fermentation, extraction, and isolation of secondary metabolites.

Strains were cultivated in 10 × 2.5 L flasks with 500 mL PD liquid medium at 25 °C for 14 days. Culture supernatants were extracted with EtOAc, and mycelia were extracted with acetone. The combined extracts were concentrated to yield crude extracts, which were first fractionated by preparative HPLC using an ACN/H_2O gradient (15–100%, 20 min). Target compounds were purified by semi-preparative HPLC under the conditions summarized below: (+)-Palitantin (**1**, 20.0 mg, t_R = 65 min), dehydropenihydrone A (**2**, 15.0 mg, t_R = 48 min), penihydrone (**4**, 2.5 mg, t_R = 70 min), and palitanol B (**7**, 2.0 mg, t_R = 32 min) were isolated from the EtOAc extract of WT strain using MeOH/H_2O (50:50, v/v). Dehydropenihydrone B (**3**, 6.0 mg, t_R = 32 min) was obtained under the same conditions supplemented with 0.1% HCOOH. Penihydrone B (**5**, 1.0 mg, t_R = 60 min) and palitanol A (**6**, 2.0 mg, t_R = 13 min) were purified using ACN/H_2O (48:52, v/v). Palitanol C (**9**, 4.0 mg, t_R = 25 min) was purified using ACN/H_2O (36:64, v/v) from *A. nidulans* strain RG101. Penihydronal (**12**, 0.8 mg, t_R = 13 min) was obtained from *in vitro* enzyme assays of PaltB, PaltE, and PaltF with **6** (20 ml), extracted with EtOAc, and purified using ACN/H_2O (80:20, v/v) containing 0.1% HCOOH. Penihydrone C (**8**, 5.0 mg, t_R = 25 min) was isolated from *A. nidulans* RG104 (*paltACBE*) with ACN/H_2O (40:60, v/v). Dia-palitantol (**16**, 1.5 mg, t_R = 30 min) was obtained from the biotransformation of **8** by *A. nidulans* RG109 (*paltD*) and purified with ACN/H_2O (36:64, v/v).

19. Structural elucidation.

The structures of the known compounds, (+)-palitantin (**1**)[13,14] and penihydrone (**4**),[15,16] were confirmed by comparison of their MS, NMR, and optical rotation data with those reported in the literature. The structures of the newly identified compounds were elucidated through comprehensive analysis of their UV, MS, CD, and NMR data (Tables S7–S17 and Figures S18–S77).

Dehydropenihydrone A (**2**), with a $[M + Na]^+$ ion at m/z 259.1314 and molecular formula $C_{14}H_{20}O_3$, is 2 daltons smaller than **4**, suggesting the absence of two hydrogen atoms. In the ^{1}H NMR spectrum, the two methine resonances observed in **4** at δ_H 2.30 (H-1) and 2.82 (H-6) were absent in **2** (Figure S23). Correspondingly, the ^{13}C NMR spectrum exhibited two additional olefinic carbon signals at δ_C 133.9 and 149.9, indicating the presence of a new double bond between C-1 and C-6 (Figure S24). This structural feature was further supported by HMBC correlations from H-7 (δ_H 6.82) and H_2-5 (δ_H 2.43, 2.80) to C-1 (δ_C 133.9), and from H-8 (δ_H 6.82) and H_2-14 (δ_H 4.18, 4.24) to C-6 (δ_C 149.9) (Table S8 and Figure S27). NOE correlations observed for H-7 to H-9 and H-8 to H-10, together with the large coupling constant $J_{H\text{-}9/H\text{-}10}$ (15.1 Hz) established the diene system as both *trans*-(7*E*, 9*E*) geometry (Figure S28). The absolute configuration at C-4 was determined as *R* by comparison of the experimental and calculated ECD spectra (Figure S18).

Dehydropenihydrone B (**3**) was assigned based on the molecular formula $C_{14}H_{18}O_4$, possessing one additional degree of unsaturation relative to **2**. Comparison of the NMR data (Table S9 and Figures S29–S34) revealed that the hydroxymethyl resonance observed in **2** (δ_C 52.1) disappeared in **3**, while a new carboxyl signal appeared (δ_C 167.7), indicating that C-14 is a carboxylic acid in **3**. Compound **3** was found to be instable during evaporation,

undergoing gradual dehydration and subsequent aromatization of the cyclohexenone unit. In the initial ^{1}H NMR spectrum (Figure S29), weak aromatic resonances assignable to H-3′ (δ_H 6.72, d, *J* = 8.7 Hz), H-4′ (δ_H 7.16, dd, *J* = 7.8, 8.7 Hz), and H-5′ (δ_H 7.05, d, *J* = 7.8 Hz) of a trisubstituted benzene ring were detected. Upon standing for seven days at 25 °C, the methylene resonances of H_2-3 (δ_H 2.40, 2.59) and H_2-5 (δ_H 2.54, 2.80) nearly disappeared, accompanied by an increase in the intensity of aromatic signals, confirming the progressive aromatization of **3** (Figures S15 and S30).

Penihydrone B (**5**) and Penihydrone C (**8**) were assigned with identical planar structures as that of **4** based on their NMR data (Tables S11 and S14 and Figures S37–S42, and S55–S60). The large coupling constants of $J_{H\text{-}7/H\text{-}8}$ and $J_{H\text{-}9/H\text{-}10}$ (14.7 and 14.8 Hz for **5**, 14.5 and 14.5 Hz for **8**) indicate *trans*-(7*E*,9*E*) configurations of the diene system in both compounds. The coupling constants of $J_{H\text{-}1/H\text{-}6}$ (5.2 Hz in **5**, and 12.0 Hz in **8**) suggest a *cis* orientation in **5**, and a *trans-diaxial* arrangement in **8**. The relationship between H-4 and H-6 was assigned based on NOE correlations. In **5**, H-4/H-7 correlation indicates a *trans* relationship, while in **8**, H-4/H-6 correlation establishes a *cis* arrangement between H-4 and H-6. The absolute configurations were then determined by ECD calculations as 1*S*,4*R*,6*S* for **5**, and 1*S*,4*R*,6*R* for **8** (Figure S18).

Comparison of the NMR data of palitanol A (**6**) with those of the known compound virensol D revealed considerable similarity, except for the alkyl chain, which lacks one double bond and two methylene groups (Table S12 and Figures S43–S48).[17] Similar to virensol D, **6** exists as hemiacetals in solution through ring-chain tautomerism. NOE correlations between H-8 and H-10, as well as H-9 and H-11, together with the large coupling constant ($J_{H\text{-}8/H\text{-}9}$ = 15.6 Hz), support the assignment of the *trans*-(8*E*, 10*E*) configuration for the diene system. NOE correlations of the major anomer revealed that 3-OH correlates with H-5 and H-1, and H-5 correlates with H-1, establishing its relative configuration.

Palitanol B (**7**) has a $[M+Na]^+$ ion at m/z 277.1423 and a deduced molecular formula of $C_{14}H_{22}O_4$, requiring four degrees of unsaturation. Comparison of the NMR data with (+)-palitantin (**1**) revealed that **7** shares the same alkyl chain, but features an additional carbonyl resonance (δ_C 198.6) (Table S13 and Figures S49–S54). Considering the degree of unsaturation, **7** was therefore inferred to adopt an acyclic structure lacking the cyclohexanone moiety present in **1**. HMBC correlations from H-9 (δ_H 7.18) to C-7 (δ_C 198.6) and from H-8 (δ_H 6.11) to C-6 (δ_C 47.2) indicate that the carbonyl C-7 is connected to C-8 of the alkyl chain and to methylene C-6. ^{1}H-^{1}H COSY correlations from H-5 (δ_H 4.35) to H-4 (δ_H 2.53) and H_2-6 (δ_H 2.60, 2.69), together with the HMBC correlation from H-4 to C-6, confirm that the hydroxyl-bearing methine C-5 is located between C-4 and C-6. HMBC correlations from H-1 (δ_H 3.63), H-2 (δ_H 2.56), H-4, and H-5 to C-3 (δ_C 208.5), along with ^{1}H-^{1}H COSY correlations from H-1 to H-2, establish the connectivity of the carbonyl C-3 to C-2 and C-4, and the hydroxymethyl C-1 to C-2. Collectively, these data allowed the unambiguous assignment of the planar structure of **7**.

Comparison of the NMR data of palitanol C (**9**) with those of the known compound virensol C[17] revealed close similarity, except for the alkyl chain, which lacks one double bond and two methylene groups (Table S15 and Figures S61–S65). Semipreparative HPLC afforded **9** in high purity; however, solvent removal resulted in the formation of a mixture (Figure S8). This

observation indicates that, similar to **6**, **9** exists as interconverting hemiacetal forms in solution through ring-chain tautomerism.

Penihydronal (**12**) showed a $[M+H]^+$ ion at *m/z* 237.1469, consistent with the molecular formula $C_{14}H_{20}O_3$, identical to that of **2**. Comparison of their NMR data revealed that the hydroxymethyl resonance (δ_H 4.18, 4.24, δ_C 52.1) observed in **2** was absent in **12** with an aldehyde signal (δ_H 8.71, δ_C 189.5) instead, suggesting that C-14 is an aldehyde in **12** (Tables S16 and Figures S66–S71). This assignment was further supported by the HMBC correlation from H-14 (δ_H 8.71) to C-2 (δ_C 182.1). In addition, the C-1/C-6 olefinic carbon signals in **2** were absent in **12**, while a methine (δ_C 34.7) and an olefinic carbon (δ_C 110.2) appeared, consistent with enolization of the C-2 ketone, resulting in a C-1/C-2 double bond and saturation at C-6 (δ_C 34.7). This is consistent with the upfield shift of C-2 (δ_C 182.1) and the presence of an exchangeable proton (δ_H 14.61). HMBC correlations from H-6 (δ_H 3.42) to C-4 (δ_C 63.4) and C-8 (δ_C 132.2) further support the assignment. The large coupling constants $J_{H-7/H-8}$ (14.0 Hz) and $J_{H-9/H-10}$ (14.0 Hz) indicate the *trans*-(7*E*, 9*E*) configurations, and NOE correlations between H-4 and H-7 suggest a *trans* relationship between H-4 and H-6. The absolute configurations were assigned as 4*R*,6*S*, based on its enzymatic conversion to **4** by recombinant PaltG in the presence of NADPH (Figure 4Gii).

Dia-palitantol (**16**) showed a $[M+Na]^+$ ion at *m/z* 279.1573, consistent with the molecular formula $C_{14}H_{24}O_4$, which contains two more hydrogen atoms than (+)-palitantin (**1**). Comparison of their NMR data revealed the absence of the C-2 ketone carbonyl resonance at δ_C 211.7 in **16** and the presence of signals for a new oxygenated methine residue (δ_H 3.88, δ_C 69.6), indicating reduction of the C-2 carbonyl to a hydroxy-bearing methine (Tables S16 and Figures S72–S77). This assignment was supported by the HMBC correlation from H-2 (δ_H 3.88) to C-6 (δ_C 35.9), establishing **16** as the C-2 reduced analogue of **1**. The relative configuration of **16** was deduced from the vicinal coupling constants. The large coupling constant ($J_{H-1/H-6}$ = 12.8 Hz) indicated a *trans-diaxial* relationship between H-1 and H-6, whereas the small coupling constant ($J_{H-2/H-3}$ = $J_{H-3/H-4}$ = 3.3 Hz) supported *cis* relationships among H-2, H-3, and H-4. Since **16** was obtained as a biotransformation product of **8** (Figure 5), the pre-existing stereocenters were considered to be retained, allowing the absolute configuration of **16** to be assigned as 1*S*, 2*S*, 3*R*, 4*R*, 6*R*. A stereoisomer (palitantol) with the configuration (1*R*, 2*S*, 3*R*, 4*R*, 6*S*) was obtained by chemical reduction of palitantin.[14] Therefore, we named **16** dia-palitanol.

20. Physicochemical properties of the compounds described in this study.

(+)-Palitantin (**1**): Crystalline white solid; $[\alpha]_D^{20}$ = +92 (*c* 0.1, MeOH); HRMS (ESI) *m/z*: $[M + Na]^+$ calcd. for $C_{14}H_{22}NaO_4^+$ 277.1410, found 277.1413. NMR data are given in Table S7, and NMR spectra in Figures S21–S22.

Dehydropenihydrone A (**2**): Pale yellow oil; $[\alpha]_D^{20}$ = +106 (*c* 0.2, MeOH); HRMS (ESI) *m/z*: $[M + Na]^+$ calcd. for $C_{14}H_{20}NaO_3^+$ 259.1305, found 259.1314. NMR data are given in Table S8 and NMR spectra in Figures S23–S28.

Dehydropenihydrone B (**3**): Yellow oil; HRMS (ESI) *m/z*: $[M + Na]^+$ calcd. for $C_{14}H_{18}NaO_4^+$ 273.1097, found 273.1106. NMR data are given in Table S9 and NMR spectra in Figures S29–S34.

Penihydrone (**4**): White needles; $[\alpha]_D^{20}$ = +30 (*c* 0.4, MeOH); HRMS (ESI) *m/z*: $[M + Na]^+$ calcd. for $C_{14}H_{22}NaO_3^+$ 261.1461, found 261.1474. NMR data are given in Table S10 and NMR spectra in Figures S35–S36.

Penihydrone B (**5**): White solid; $[\alpha]_D^{20}$ = +45 (*c* 0.4, MeOH); HRMS (ESI) *m/z*: $[M + Na]^+$ calcd. for $C_{14}H_{22}NaO_3^+$ 261.1461, found 261.1471. NMR data are given in Table S11 and NMR spectra in Figures S37–S42.

Palitanol A (**6**): Orange-red oil; HRMS (ESI) *m/z*: $[M + Na]^+$ calcd. for $C_{14}H_{22}NaO_4^+$ 277.1410, found 277.1419. NMR data are given in Table S12 and NMR spectra in Figures S43–S48.

Palitanol B (**7**): Yellow oil; $[\alpha]_D^{20}$ = +31 (*c* 0.3, MeOH); HRMS (ESI) *m/z*: $[M + Na]^+$ calcd. for $C_{14}H_{22}NaO_4^+$ 277.1410, found 277.1423. NMR data are given in Table S13 and NMR spectra in Figures S49–S54.

Penihydrone C (**8**): Brown solid; $[\alpha]_D^{20}$ = +70 (*c* 0.3, MeOH); HRMS (ESI) *m/z*: $[M + Na]^+$ calcd. for $C_{14}H_{22}NaO_3^+$ 261.1461, found 261.1470. NMR data are given in Table S14 and NMR spectra in Figures S55–S60.

Palitanol C (**9**): Brown oil; HRMS (ESI) *m/z*: $[M + Na]^+$ calcd. for $C_{14}H_{24}NaO_4^+$ 279.1572, found 279.1574. NMR data are given in Table S15 and NMR spectra in Figures S61–S65.

Penihydronal (**12**): Brown oil; $[\alpha]_D^{20}$ = +50 (*c* 0.1, MeOH); HRMS (ESI) *m/z*: $[M + H]^+$ calcd. for $C_{14}H_{21}O_3^+$ 237.1485, found 237.1469. NMR data are given in Table S16 and NMR spectra in Figures S66–S71.

Dia-palitantol (**16**): Amorphous white solid; $[\alpha]_D^{20}$ = +42 (*c* 0.1, MeOH); HRMS (ESI) *m/z*: $[M + Na]^+$ calcd. for $C_{14}H_{24}NaO_4^+$ 279.1572, found 279.1573. NMR data are given in Table S17 and NMR spectra in Figures S72–S77.

Supplementary Tables

Table S1. Proteins encoded by the *palt* cluster in *Penicillium palitans* NRRL 2033.

Protein	Location at PZ404400	Length	Homologous know protein	Identity/(%) (cover/%)	Putative function
PaltA	1–7849	2382 aa	SrdA (Q7SHI6.1) from *Neurospora crassa* OR74A	52.4 (98)	Highly reducing polyketide synthase
PaltB	8397–9707	285 aa	SrdE (Q7SHI2.1) from *Neurospora crassa* OR74A	59.7 (99)	Short-chain dehydrogenases/reductases
PaltC	10061–11055	274 aa	RusB (W6QKQ9.1) from *Penicillium roqueforti* FM164	53.9 (98)	Short-chain dehydrogenases/reductases
PaltD	11392–13195	497 aa	ABA1 (G4N2Y3.1) from *Pyricularia oryzae* 70-15	44.3 (97)	Cytochrome P450
PaltE	13868–14523	198 aa	SrdB (Q7SHI5.1) from *Neurospora crassa* OR74A	50.0 (94)	Cupin domain-containing protein
PaltF	14878–15867	329 aa	UcsJ (QBC88154.1) from *Acremonium* sp. KY4917	28.0 (93)	Ene-reductase
PaltG	16202–17271	338 aa	DmxR8 (A0A4P8DJW5.1) from *Cryptosporiopsis* sp.	38.1 (100)	Short-chain dehydrogenases/reductases
PaltH	17692–19555	559aa	PgmG (A0A1W5SP56.1) from *Aspergillus terreus*	50.1 (98)	MFS-type transporter
PaltI	22354–24790	679aa	TSF1 (B0ZT45.1) from *Elsinoe fawcettii*	32.2 (68)	Transcription factor
PaltJ	26574–28300	507aa	AnuG (W6QEK0.1) from *Penicillium roqueforti* FM164	50.5 (93)	Berberine bridge enzyme-like protein

Table S2. Sequence identities of the *palt* cluster from *Penicillium palitans* NRRL 2033 with those of different *P. palitans* strains.

Strain	*palt* cluster	Annotation	Sequences location in GenBank	Length	Identities
P. palitans SP3	√	√	CACTIN020000688.1 6293–34583	28291 bp	99%
P. palitans F8_6S-4F	√	-	JAHAOX010000099.1 25937–54227	28291 bp	99%
P. palitans F8_6S-1F	√	-	JAHAOZ010000125.1 25937–54227	28291 bp	99%
P. palitans F8_6S-3F	√	-	JAHAOY010000099.1 25937–54227	28291 bp	99%
P. palitans F8_6S_5F	√	-	JAHAPC010000101.1 25937–54227	28291 bp	99%
P. palitans F8_6S_2F	√	-	JAHAPD010000101.1 41136–69426	28291 bp	99%
P. palitans F6_8S_1C_F	√	-	JAHAQH010000103.1 41136–69426	28291 bp	99%
P. palitans F6_8S_1A_F	√	-	JAHAQI010000100.1 41136–69426	28291 bp	99%
P. palitans F6_7S_1C_F	√	-	JAHAQQ010000098.1 25937–54227	28291 bp	99%
P. palitans F6_7S_1A_F	√	-	JAHAQR010000096.1 28911–57201	28291 bp	99%
P. palitans F6_6S_1_F	√	-	JAHARA010000105.1 41136–69426	28291 bp	99%
P. palitans F6_4S_1B_F	√	-	JAHARL010000104.1 41136–69426	28291 bp	99%
P. palitans F6_4S_1C_F	√	-	JAHARK010000105.1 25937–54227	28291 bp	99%
P. palitans F6_4S_1A_F	√	-	JAHARM010000097.1 28913–57203	28291 bp	99%

Table S3. Sequence identities of proteins encoded by the *palt* cluster from *Penicillium palitans* NRRL 2033 with those of known clusters.

Penicillium palitans NRRL 2033	*Neurospora crassa*	*Trichoderma virens*	*Aspergillus ruber*	*Penicillium roqueforti* FM164		*Stachybotrys sp.* g12	*Aspergillus ustus* 3.3904	Putative Function
PaltA	SrdA (98/52.4)	VirA (98/43.9)	FogA (99/45.3)	AnuA (89/46.4)	RusA (99/46.1)	StrA (98/45.1)	PsaPS (98/47.9)	Highly reducing polyketide synthase
PaltB	SrdE (99/59.7)	VirD (98/57.4)	FogD (98/58.4)	AnuB (88/57.6)	RusD (95/56.2)	StrD (95/56.3)	PsaOX1 (94/58.4)	Short-chain dehydrogenase /reductases
PaltC	SrdC (98/50.2)	VirB (98/48.0)	FogB (98/49.1)	AnuF (99/51.1)	RusB (98/53.9)	StrB (98/44.6)	PsaOX2 (99/52.9)	Short-chain dehydrogenase /reductases
PaltD								Cytochrome P450
PaltE	SrdB (94/50.0)	VirC (84/53.9)	FogC (94/52.7)	AnuC (83/47.2)	RusC (89/52.8)	StrC (89/51.7)	PsaCP (91/49.2)	Cupin domain-containing protein
PaltF								Ene-reductase
PaltG		VirG (91/34.8)	FogG (90/30.9)	AnuD (99/33.7)				Short-chain dehydrogenase /reductases
		VirL (96/32.5)		AnuI (81/25.4)				
PaltJ				AnuG (93/50.5)				Berberine bridge enzyme-like protein

The identity of each homolog with *Penicillium palitans* NRRL 2033 is shown as coverage/identity (%).

Table S4. Strains used and created in this study.

Strain	Genotype	Source
E. coli		
DH5α	F-*endA1 glnV44 thi-1 recA1 relA1 gyrA96 deoR nupG purB20* φ80dl*acZ*ΔM15 Δ(*lacZYA-argF*)U169, hsdR17($r_K^- m_K^+$), λ⁻	[2]
BL21(DE3)	*fhuA2 [lon] ompT gal (λ DE3) [dcm] ∆hsdS* *λ DE3 = λ sBamHIo ∆EcoRI-B int::(lacI::PlacUV5::T7 gene1) i21 ∆nin5*	[3]
Penicillium palitans		
NRRL 2033	wild type	NRRL*
RG01	Δ*paltA* in NRRL 2033	This study
RG02	Δ*paltI* in NRRL 2033	This study
RG03	Δ*paltJ* in NRRL 2033	This study
RG04	Δ*paltC* in NRRL 2033	This study
RG05	Δ*paltB* in NRRL 2033	This study
RG06	Δ*paltE* in NRRL 2033	This study
RG07	Δ*paltF* in NRRL 2033	This study
RG08	Δ*paltG* in NRRL 2033	This study
RG09	Δ*paltD* in NRRL 2033	This study
Aspergillus nidulans		
LO8030	*pyroA4*, *riboB2*, *AfpyrG89*, *nkuA*::*argB*, deletion of seven secondary metabolite biosynthetic gene clusters (sterigmatocystin, emericellamide, asperfuranone, monodictyphenone, terrequinone, austinol, asperthecin)	[18]
BK06	Δ*wA*-PKS::*gpdA*(p)-*Afribo* in LO8030	[19]
ZL132	Δ*wA*-PKS::*gpdA*(p)-*Afribo-hlyA*(p)-*gpdA*#(p) in LO8030	[10]
RG101	Δ*wA*-PKS::*gpdA*(p):*paltA-Afribo-hlyA*(p)-*gpdA*(p) in LO8030	This study
RG102	Δ*wA*-PKS::*gpdA*(p):*paltA-Afribo-hlyA*(p):*paltC-gpdA*(p) in LO8030	This study
RG103	Δ*wA*-PKS::*gpdA*(p):*paltA-Afribo-hlyA*(p):*paltC-gpdA*(p):*paltB* in LO8030	This study
RG104	*paltACBE*, pRG21 (*paltE*) in *A. nidulans* RG103 (*paltACB*)	This study
RG105	*paltACBEF*, pRG22 (*paltF*) in *A. nidulans* RG104 (*paltACBE*)	This study
RG106	*paltACBEG*, pRG23 (*paltG*) in *A. nidulans* RG104 (*paltACBE*)	This study
RG107	*paltAE*, pRG21 (*paltE*) in *A. nidulans* RG101 (*paltA*)	This study
RG108	*paltACE*, pRG21 (*paltE*) in *A. nidulans* RG102 (*paltAC*)	This study
RG109	Δ*wA*-PKS::*gpdA*(p):*paltD-Afribo* in LO8030	This study

*NRRL: ARS Culture Collection

Table S5. Plasmids used and constructed in this study.

Plasmid	Description	Source
pJN017	*URA3*, *wA flanking*, *gpdA*(p), *afriboB*, *ampR*	[19]
pZL132	*URA3*, *wA flanking*, *gpdA*(p), *afriboB*, *hlyA*(p), *gpdA*(p) from pYTR, *ampR*	[10]
pZL69	*AMA1*, *URA3*, *A. fumigatus pyrG* (*AfpyrG*), *ampR*, P_{gpdA}	[11]
pZL70	*AMA1, URA3, A. fumigatus pyroA (AfpyroA), ampR,* P_{gpdA}	This study
p5HY	Two-thirds of the hph resistance gene at the 5'-end, originated from the pUChph and inserted into pESC-URA for gene replacement using hph as a selection marker	[8]
P3YG	Two-thirds of the hph resistance gene at the 3'-end, originated from the pUChph and inserted into pESC-URA for gene replacement using hph as a selection marker	[8]
pET28a(+)	vector with T7 promoter, 6xHis tag, T7 terminator, kanamycin resistance	Merck, KGaA (Darmstadt, Germany)
pZZ21 (p5HY-*paltA*)	a 1378 bp upstream PCR fragment of *paltA* from genomic DNA of *P. palitans* NRRL 2033 inserted in p5HY	This study
pZZ22 (p3YG-*paltA*)	a 1452 bp downstream PCR fragment of *paltA* from genomic DNA of *P. palitans* NRRL 2033 inserted in p3YG	This study
pRG01 (p5HY-*paltB*)	a 1543 bp upstream PCR fragment of *paltB* from genomic DNA of *P. palitans* NRRL 2033 inserted in p5HY	This study
pRG02 (p3YG-*paltB*)	a 1448 bp downstream PCR fragment of *paltB* from genomic DNA of *P. palitans* NRRL 2033 inserted in p3YG	This study
pRG03 (p5HY-*paltC*)	a 1480 bp upstream PCR fragment of *paltC* from genomic DNA of *P. palitans* NRRL 2033 inserted in p5HY	This study
pRG04 (p3YG-*paltC*)	a 1492 bp downstream PCR fragment of *paltC* from genomic DNA of *P. palitans* NRRL 2033 inserted in p3YG	This study
pRG05 (p5HY-*paltD*)	a 1543 bp upstream PCR fragment of *paltD* from genomic DNA of *P. palitans* NRRL 2033 inserted in p5HY	This study
pRG06 (p3YG-*paltD*)	a 1408 bp downstream PCR fragment of *paltD* from genomic DNA of *P. palitans* NRRL 2033 inserted in p3YG	This study
pRG07 (p5HY-*paltE*)	a 1612 bp upstream PCR fragment of *paltE* from genomic DNA of *P. palitans* NRRL 2033 inserted in p5HY	This study
pRG08 (p3YG-*paltE*)	a 1422 bp downstream PCR fragment of *paltE* from genomic DNA of *P. palitans* NRRL 2033 inserted in p3YG	This study
pRG09 (p5HY-*paltF*)	a 1408 bp upstream PCR fragment of *paltF* from gDNA of *P. palitans* NRRL 2033 inserted in p5HY	This study
pRG10 (p3YG-*paltF*)	a 1443 bp downstream PCR fragment of *paltF* from genomic DNA of *P. palitans* NRRL 2033 inserted in p3YG	This study
pRG11 (p5HY-*paltG*)	a 1477 bp upstream PCR fragment of *paltG* from genomic DNA of *P. palitans* NRRL 2033 inserted in p5HY	This study
pRG12 (p3YG-*paltG*)	a 1385 bp downstream PCR fragment of *paltG* from genomic DNA of *P. palitans* NRRL 2033 inserted in p3YG	This study

Table S5. Plasmids used and constructed in this study (continued).

Plasmid	Description	Source
pRG13 (p5HY-*paltI*)	a 1388 bp upstream PCR fragment of *paltI* from genomic DNA of *P. palitans* NRRL 2033 inserted in p5HY	This study
pRG14 (p3YG-*paltI*)	a 1478 bp downstream PCR fragment of *paltI* from genomic DNA of *P. palitans* NRRL 2033 inserted in p3YG	This study
pRG15 (p5HY-*paltJ*)	a 1490 bp upstream PCR fragment of *paltJ* from genomic DNA of *P. palitans* NRRL 2033 inserted in p5HY	This study
pRG16 (p3YG-*paltJ*)	a 1442 bp downstream PCR fragment of *paltJ* from genomic DNA of *P. palitans* NRRL 2033 inserted in p3YG	This study
pRG18	pZL132-*gpdA*(p)-*paltA-afriboB*; a 8425 bp fragment of *paltA* with its terminator from genomic DNA of *P. palitans* NRRL 2033 inserted in pZL132	This study
pRG19	pZL132-*gpdA*(p)-*paltA-afriboB-hlyA*(p)-*paltC-gpdA*(p) from pYTR; a 8425 bp fragment of *paltA* with its terminator and a 1611 bp fragment of *paltC* with its terminator from genomic DNA of *P. palitans* NRRL 2033 inserted in pZL132	This study
pRG20	pZL132-*gpdA*(p)-*paltA-afriboB-hlyA*(p)-*paltC-paltB-gpdA*(p) from pYTR; a 8425 bp fragment of *paltA* with its terminator, a 1611 bp fragment of *paltC* with its terminator, and a 1664 bp fragment of *paltB* with its terminator from genomic DNA of *P. palitans* NRRL 2033 inserted in pZL132	This study
pRG21	pZL70-*gpdA*(p)-*paltE-AfpyroA*; a 1242 bp fragment of *paltE* with its terminator from genomic DNA of *P. palitans* NRRL 2033 inserted in pZL70	This study
pRG22	pZL69-*gpdA*(p)-*paltF-AfpyrG*; a 1491 bp fragment of *paltF* with its terminator from genomic DNA of *P. palitans* NRRL 2033 inserted in pZL69	This study
pRG23	pZL69-*gpdA*(p)-*paltG-AfpyrG*; a 1572 bp fragment of *paltG* with its terminator from genomic DNA of *P. palitans* NRRL 2033 inserted in pZL69	This study
pRG24	pJN017-*gpdA*(p)-*paltD-afriboB*; a 2385 bp fragment of *paltD* with its terminator from genomic DNA of *P. palitans* NRRL 2033 inserted in pJN017	This study
pRG25	pET28a(+)-*paltB*; an 858 bp fragment of *paltB* from cDNA of *P. palitans* NRRL 2033 inserted in pET28a(+) at the restriction sites BamHI and EcoRI	This study
pRG26	pET28a(+)-*paltE*; a 597 bp fragment of *paltE* from cDNA of *P. palitans* NRRL 2033 inserted in pET28a(+) at the restriction sites BamHI and EcoRI.	This study
pRG27	pET28a(+)-*paltF*; a 990 bp fragment of *paltF* from cDNA of *P. palitans* NRRL 2033 inserted in pET28a(+) at the restriction sites BamHI and EcoRI.	This study
pRG28	pET28a(+)-*paltG*; a 1017 bp fragment of *paltG* from cDNA of *P. palitans* NRRL 2033 inserted in pET28a(+) at the restriction sites BamHI and EcoRI.	This study

Table S6. Primers used in this study.

Primer	Oligonucleotide sequence 5'-3'	Function
RG_5-Hygsplit-R	TCGAAGTAGCGCGTCTGC	Amplification of 2/3 from the *hph* gene (5'-end) for split marker cloning
RG_3-Hygsplit-F	TTGGGGAATTCAGCGAGAGC	Amplification of 2/3 from the *hph* gene (3'-end) for split marker cloning
ZZ_pZZ21-F	AAGAATTGTTAATTAAGAGCTCAGATCctgataatgttcacaacgcgaac	Amplification of the upstream region of *paltA* from *P. palitans* NRRL 2033 to construct pZZ21
ZZ_pZZ21-R	CCTCACTAAAGGGCGGCCcaccttgaatatagggcgacat	
ZZ_pZZ22-F	ACTCACTATAGGGCCCGGGCGgaattatcgtccactggagcg	Amplification of the downstream region of *paltA* from *P. palitans* NRRL 2033 to construct pZZ22
ZZ_pZZ22-R	TAGCCGCGGTACCAAGCTTACTCGAcacattcatcagatgcacaacc	
RG_pRG01-F	AAGAATTGTTAATTAAGAGCTCAGATCgccatatggacagcatacagag	Amplification of the upstream region of *paltB* from *P. palitans* NRRL 2033 to construct pRG01
RG_pRG01-R	CCTCACTAAAGGGCGGCCgaatatcagtgctcgtgtgtcag	
RG_pRG02-F	ACTCACTATAGGGCCCGGGCGgagtggaaccagctgcatatg	Amplification of the downstream region of *paltB* from *P. palitans* NRRL 2033 to construct pRG02
RG_pRG02-R	TAGCCGCGGTACCAAGCTTACTCGActacatgtcgctaggtaagcg	
RG_pRG03-F	AAGAATTGTTAATTAAGAGCTCAGATCctgacacacgagcactgatattc	Amplification of the upstream region of *paltC* from *P. palitans* NRRL 2033 to construct pRG03
RG_pRG03-R	CCTCACTAAAGGGCGGCCcttgctcgcggaactagtac	
RG_pRG04-F	ACTCACTATAGGGCCCGGGCGcttgccttccgcttacctag	Amplification of the downstream region of *paltC* from *P. palitans* NRRL 2033 to construct pRG04
RG_pRG04-R	TAGCCGCGGTACCAAGCTTACTCGActgaatctgttgaacgagagtg	
RG_pRG05-F	AAGAATTGTTAATTAAGAGCTCAGATCgagtggaaccagctgcatatg	Amplification of the upstream region of *paltD* from *P. palitans* NRRL 2033 to construct pRG05
RG_pRG05-R	CCTCACTAAAGGGCGGCCctccattacggtgattggtcg	
RG_pRG06-F	ACTCACTATAGGGCCCGGGCGgagtctgtcatggaatgcatg	Amplification of the downstream region of *paltD* from *P. palitans* NRRL 2033 to construct pRG06
RG_pRG06-R	TAGCCGCGGTACCAAGCTTACTCGAccggaggtccgatataatgc	
RG_pRG07-F	AAGAATTGTTAATTAAGAGCTCAGATCcatggacagtgtggtatgaagg	Amplification of the upstream region of *paltE* from *P. palitans* NRRL 2033 to construct pRG07
RG_pRG07-R	CCTCACTAAAGGGCGGCCctggataacacacgaacttgc	
RG_pRG08-F	ACTCACTATAGGGCCCGGGCGcgtgacacatatgacacatacgtgccg	Amplification of the downstream region of *paltE* from *P. palitans* NRRL 2033 to construct pRG08

Table S6. Primers used in this study (continued).

Primer	Oligonucleotide sequence 5'-3'	Function
RG_pRG08-R	TAGCCGCGGTACCAAGCTTACTCGAgaccaacgtcaagctgaatcc	Amplification of the downstream region of *paltE* from *P. palitans* NRRL 2033 to construct pRG08
RG_pRG09-F	AAGAATTGTTAATTAAGAGCTCAGATCgagtctgtcatggaatgcatg	Amplification of the upstream region of *paltF* from *P. palitans* NRRL 2033 to construct pRG09
RG_pRG09-R	CCTCACTAAAGGGCGGCCccggaggtccgatataatgc	
RG_pRG10-F	ACTCACTATAGGGCCCGGGCGgcttgagaggtgcagttatc	Amplification of the downstream region of *paltF* from *P. palitans* NRRL 2033 to construct pRG10
RG_pRG10-R	TAGCCGCGGTACCAAGCTTACTCGAcgagaatgacctcatgaagc	
RG_pRG11-F	AAGAATTGTTAATTAAGAGCTCAGATCgagaatcatctccttgttccg	Amplification of the upstream region of *paltG* from *P. palitans* NRRL 2033 to construct pRG11
RG_pRG11-R	CCTCACTAAAGGGCGGCCgcttcatgaggtcattctcg	
RG_pRG12-F	ACTCACTATAGGGCCCGGGCGcgtaaggtggattgcgatacc	Amplification of the downstream region of *paltG* from *P. palitans* NRRL 2033 to construct pRG12
RG_pRG12-R	TAGCCGCGGTACCAAGCTTACTCGAcgaagcaggactcaagcaac	
RG_pRG13-F	AAGAATTGTTAATTAAGAGCTCAGATCGCCGTCATGTTATACTCTGC	Amplification of the upstream region of *paltI* from *P. palitans* NRRL 2033 to construct pRG13
RG_pRG13-R	ACCCTCACTAAAGGGCGGCCGTGGATGGCTGAATGAGACC	
RG_pRG14-F	ACTCACTATAGGGCCCGGGCGCGGTTGCGAATGGACTAATAG	Amplification of the downstream region of *paltI* from *P. palitans* NRRL 2033 to construct pRG14
RG_pRG14-R	TAGCCGCGGTACCAAGCTTACTCGACGTATAATTGAACGAGAACGCC	
RG_pRG15-F	AAGAATTGTTAATTAAGAGCTCAGATCCGCACTCCAGATATGTTCGC	Amplification of the upstream region of *paltJ* from *P. palitans* NRRL 2033 to construct pRG15
RG_pRG15-R	ACCCTCACTAAAGGGCGGCCGGCACAGTTGCTAATGCTAAG	
RG_pRG16-F	ACTCACTATAGGGCCCGGGCGCAATGCCTTCGGCTTCTATAAC	Amplification of the downstream region of *paltJ* from *P. palitans* NRRL 2033 to construct pRG16
RG_pRG16-R	TAGCCGCGGTACCAAGCTTACTCGACCACTATTATCGCGTATCTAGGC	
RG_paltA-F1	gattgacagcttggacctgtc	Screening of Δ*paltA* transformant *P. palitans* RG01
RG_paltA-R1	cgcatcgttgaatgaatcttgc	
RG_paltA-F2	catcaatgcaccggatcatgc	
RG_paltA-R2	gattggtcttgctggctatgtc	
RG_paltI-F1	GGTCTCATTCAGCCATCCAC	Screening of Δ*paltI* transformant *P. palitans* RG02

Table S6. Primers used in this study (continued).

RG_paltI-R1	GTGGAGAAGTTCTTGAACGG	Screening of Δ*paltI* transformant *P. palitans* RG02
RG_paltI-F2	CTGGTCTGCCTGAACGTATTC	
RG_paltI-R2	GCAGGCAGTTCAACTTACTG	
RG_paltJ-F1	CTTAGCATTAGCAACTGTGCC	Screening of Δ*paltJ* transformant *P. palitans* RG03
RG_paltJ-R1	GTTATAGAAGCCGAAGGCATTG	
RG_paltJ-F2	CCAATTCGCAGGACTAGTAGAC	
RG_paltJ-R2	CTGATACTGAGTAGGCGCTAAG	
RG_paltC-F1	gaaggaagcggaataggacg	Screening of Δ*paltC* transformant *P. palitans* RG04
RG_paltC-R1	catctgtaaggtggttccgc	
RG_paltC-F2	cttggcagatacgacgaggtc	
RG_paltC-R2	gagactctgcggatgtgatgg	
RG_paltB-F1	gctgcaggcattattgaactg	Screening of Δ*paltB* transformant *P. palitans* RG05
RG_paltB-R1	catcaatgcaccggatcatgc	
RG_paltB-F2	gtatgacaagttgacgtgcc	
RG_paltB-R2	catccggagcgaatatccgac	
RG_paltE-F1	ctatgccgatatctatgccacc	Screening of Δ*paltE* transformant *P. palitans* RG06
RG_paltE-R1	ggttggcaatccaacagcac	
RG_paltE-F2	cacgtaaggtggattgcgatac	
RG_paltE-R2	gtcgtacgagacgacatgctg	
RG_paltF-F1	caccggtacaaccggatatc	Screening of Δ*paltF* transformant *P. palitans* RG07
RG_paltF-R1	cgacagtctcatccataagagc	
RG_paltF-F2	gtcctgagcgatggatcgaag	
RG_paltF-R2	gcgtcacgtgatgatatgctgg	

Table S6. Primers used in this study (continued).

Primer	Oligonucleotide sequence 5'-3'	Function
RG_paltG-F1	ggaagacaatcctcatcaccg	Screening of Δ*paltG* transformant *P. palitans* RG08
RG_paltG-R1	gtactcggttcgaacctcagg	
RG_paltG-F2	cagttccgataccgccgatg	
RG_paltG-R2	ggcaacaagacacgaagctcag	
RG_paltD-F1	gcgctgatagtgtgaaggtg	Screening of Δ*paltD* transformant *P. palitans* RG09
RG_paltD-R1	cttcggagaagcttcaggttc	
RG_paltD-F2	gcatgatccggtgcattgatg	
RG_paltD-R2	gagtcctgcttcgcacttgac	
RG_pRG18-F	GCTACCCCGCTTGAGCAGACATCACCatgtcgccctatattcaaggtg	Amplification of the *paltA* gene and its terminator from *P. palitans* NRRL 2033 to construct pRG18, pRG19, and pRG20
RG_pRG18-R	CTCAACACCATATTTTAATCCCATGTGgtcttaccttaaccactcggc	
RG_pRG19-F	CATCAATCACCCTTCACACCACAACACCatgtccgttgatgggtttgc	Amplification of the *paltC* gene and its terminator from *P. palitans* NRRL 2033 to construct pRG19 and pRG20
RG_pRG19-R	tagacacatctaaacaTAAAgcggccgcccttcacactatcagcgctg	
RG_pRG20-F	taccccgccacatagacacatctaaacaatgcagctcacaaagtttgc	Amplification of the *paltB* gene and its terminator from *P. palitans* NRRL 2033 to construct pRG20
RG_pRG20-R	ttttcagcgctgatagtgtgaaggtttgtcttatctgttcttgaac	
RG_pRG21-F	AGCTACCCCGCTTGAGCAGACATCACCatgtcgtccaaagtctatgcc	Amplification of the *paltE* gene and its terminator from *P. palitans* NRRL 2033 to construct pRG21
RG_pRG21-R	gaggtcgacggtatcgataagctgatccgttccaagcaaggttacacg	
RG_pRG22-F	AGCTACCCCGCTTGAGCAGACATCACCatgaaagcacaaaagattttc	Amplification of the *paltF* gene and its terminator from *P. palitans* NRRL 2033 to construct pRG22
RG_pRG22-R	cttgatatcgaattcctgcagcccggggagtgagtccgttctgttcaag	
RG_pRG23-F	AGCTACCCCGCTTGAGCAGACATCACCatgagtgctttattcggcc	Amplification of the *paltG* gene and its terminator from *P. palitans* NRRL 2033 to construct pRG23
RG_pRG23-R	cttgatatcgaattcctgcagcccggggagttgagtgttcctcaggc	
RG_pRG24-F	CAGCTACCCCGCTTGAGCAGACATCACCatggaagccaacagcccgaa	Amplification of the *paltD* gene and its terminator from *P. palitans* NRRL 2033 to construct pRG24
RG_pRG24-R	GCCTCAACACCATATTTTAATCCCATGTGgtgtcggactccagaattacc	
PaltB-28-F	GGTGGACAGCAAATGGGTCGCGGATCCatgcagctcacaaagtttgc	858 bp fragment of *paltB* from *P. palitans* NRRL 2033 cDNA was fused into pET28a(+) to construct pRG25
PaltB-28-R	GCAAGCTTGTCGACGGAGCTCGAATTCtcaagttagcttcactccac	
PaltE-28-F	GGTGGACAGCAAATGGGTCGCGGATCCatgtcgtccaaagtctatgcc	597 bp fragment of *paltE* from *P. palitans* NRRL 2033 cDNA was fused into pET28a(+) to construct pRG26
PaltE-28-R	GCAAGCTTGTCGACGGAGCTCGAATTCctagtgattgtcttcaaaatcag	

Table S6. Primers used in this study (continued).

**Primer**	**Oligonucleotide sequence 5'-3'**	**Function**
PaltF-28-F	GGTGGACAGCAAATGGGTCGCGGATCCatgaaagcacaaaagattttc	990 bp fragment of *paltF* from *P. palitans* NRRL 2033 cDNA was fused into pET28a(+) to construct pRG27
PaltF-28-R	GCAAGCTTGTCGACGGAGCTCGAATTCtcaaacagcggtagcggcaac	
PaltG-28-F	GGTGGACAGCAAATGGGTCGCGGATCCatgagtgctttattcggcc	1017 bp fragment of *paltG* from *P. palitans* NRRL 2033 cDNA was fused into pET28a(+) to construct pRG28
PaltG-28-R	GCAAGCTTGTCGACGGAGCTCGAATTCttagtactcggttcgaacctcag	

Note: Overlapping regions within the primers are shown in capital letters.

Table S7. NMR data of (+)-palitantin (**1**) in $CDCl_3$.

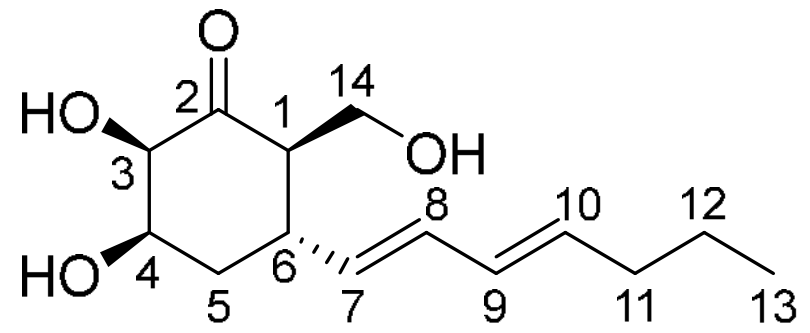

Position	δ_H, multi (*J* in Hz)	δ_C, type
1	2.39, dt (11.9, 5.0)	54.8, CH
2		211.7, C
3	4.22, d (3.6)	77.4, CH
4	4.38, m	71.9, CH
5	1.86, ddd (14.6, 12.4, 2.1)	35.5, CH_2
	2.15, dt (14.6, 3.9)	
6	2.84, m	39.3, CH
7	5.39, dd (14.9, 9.1)	131.1, CH
8	6.11, dd (14.9, 10.3)	132.9, CH
9	5.99, dd (15.0, 10.3)	129.5, CH
10	5.66, dt (15.0, 7.0)	135.4, CH
11	2.05, q (7.4)	34.9, CH_2
12	1.41, sext. (7.4)	22.5, CH_2
13	0.90, t (7.4)	13.9, CH_3
14	3.79, d (5.0)	59.9, CH_2
3-OH	3.85, s	
4-OH	2.55, s	
14-OH	2.35, brs	

The NMR data of **1** correspond well to those of (+)-palitantin published previously.[13,14]

Table S8. NMR data of dehydropenihydrone A (**2**) in DMSO-d_6.

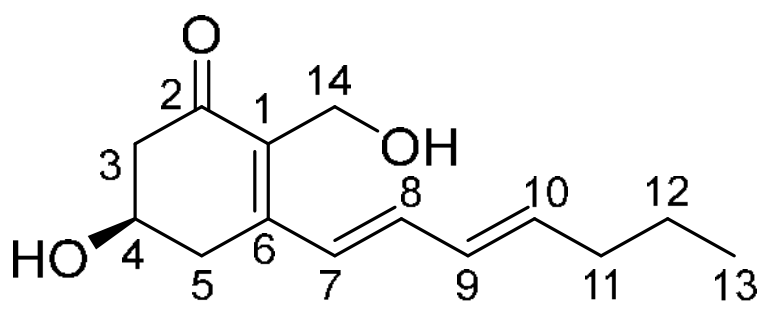

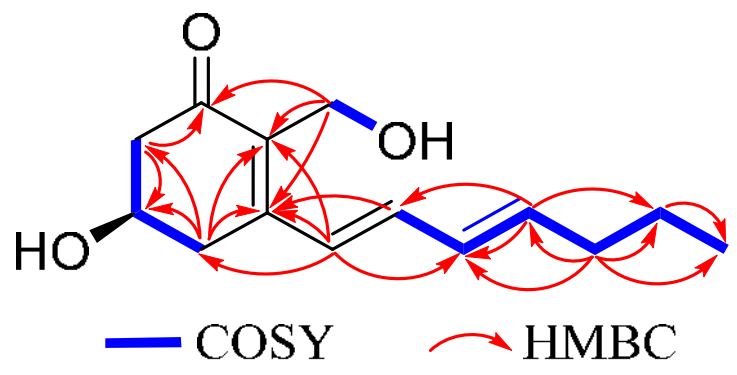

Position	δ_H, multi (*J* in Hz)	δ_C, type	Key HMBC correlations
1		133.9, C	
2		197.0, C	
3	2.33, dd (15.8, 8.6)	46.5, CH$_2$	C-2, C-4, C-5
	2.56, dd (15.8, 4.0)		C-2, C-4, C-5
4	4.03, m	64.5, CH	
5	2.43, dd (17.2, 7.2)	34.4, CH$_2$	C-1, C-3, C-4, C-6, C-7
	2.80, dd (17.2, 4.3)		C-1, C-3, C-4, C-6, C-7
6		149.9, C	
7	6.82[a], m	128.1, CH	C-1, C-5, C-6, C-8, C-9
8	6.82[a], m	136.7, CH	C-9, C-6, C-10
9	6.28, dd (15.1, 9.5)	131.0, CH	C-7, C-8, C-10, C-11
10	6.07, dt (15.1, 6.9)	140.1, CH	C-8, C-9, C-11, C-12,
11	2.12, q (7.4)	34.3, CH$_2$	C-9, C-10, C-12, C-13
12	1.41, sext. (7.4)	21.6, CH$_2$	C-10, C-11, C-13
13	0.88, t (7.4)	13.5, CH$_3$	C-11, C-12
14	4.18, dd (11.5, 3.4)	52.1, CH$_2$	C-1, C-2, C-6
	4.24, dd (11.5, 2.8)		
4-OH	5.04, s		
14-OH	4.48, s		

[a] Signals overlapping with each other.

Table S9. NMR data of dehydropenihydrone B (**3**) in DMSO-d_6.

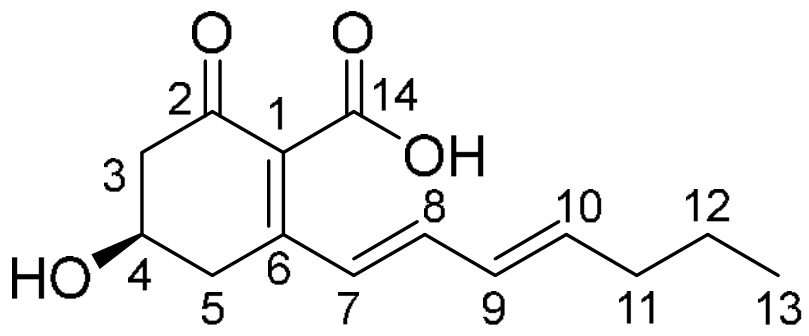

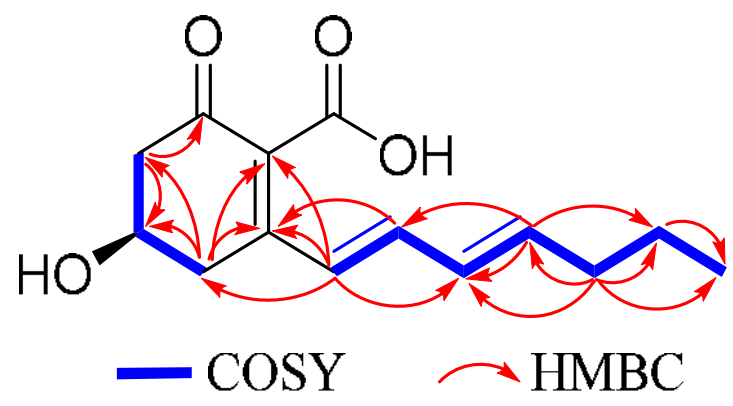

Position	δ_H, multi (*J* in Hz)	δ_C, type	Key HMBC correlations
1		132.2, C	
2		194.6, C	
3	2.40, dd (15.9, 7.4)	45.9, CH_2	C-2, C-4, C-5
	2.59, dd (15.9, 3.7)		
4	4.13, m	64.2, CH	
5	2.54, m	33.0, CH_2	C-1, C-3, C-4, C-6
	2.80, dd (17.3, 4.1)		
6		148.7, C	
7	6.34, d (15.4)	126.9, CH	C-1, C-5, C-6, C-9
8	6.92, dd (15.4, 10.4)	138.9, CH	C-6, C-10
9	6.28, dd (15.0, 10.4)	130.4, CH	C-10
10	6.15, dt (15.0, 7.1)	142.1, CH	C-8, C-9, C-12
11	2.12, q (7.4)	34.2, CH_2	C-9, C-10, C-12, C-13
12	1.41, sext. (7.4)	21.8, CH_2	C-10, C-11, C-13
13	0.88, t (7.4)	13.5, CH_3	C-11, C-12
14		167.7, C	
4-OH	5.12, s		
14-OH	n. d.		

n. d.: Signals not detected.

Table S10. NMR data of penihydrone (**4**) in acetone-d_6.

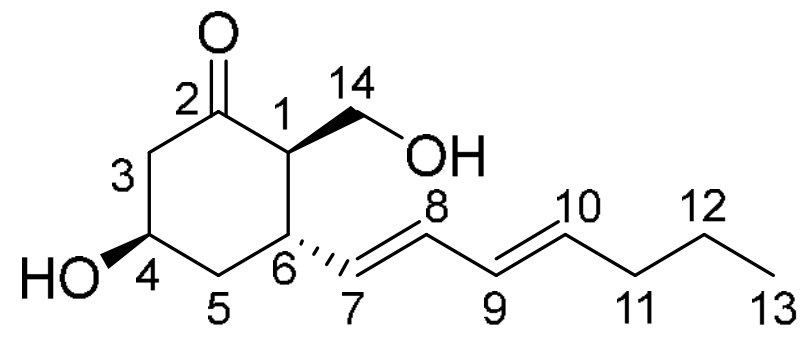

Position	δ_H, multi (*J* in Hz)	δ_C, type
1	2.30, ddd (11.3, 6.0, 3.5)	57.5, CH
2		212.1, C
3	2.34, dd (14.0, 3.9)	39.9, CH_2
	2.63, dd (14.0, 3.4)	
4	4.36, m	69.1, CH
5	1.88, dd (9.6, 3.6)	39.7, CH_2
6	2.82, m	50.1, CH
7	5.49, dd (13.9, 9.3)	134.4, CH
8	6.02[a], m	132.4, CH
9	6.02[a], m	131.6, CH
10	5.59, dt (14.2, 6.8)	135.1, CH
11	2.00, q (7.4)	35.8, CH_2
12	1.36, sext. (7.4)	23.6, CH_2
13	0.85, t (7.4)	14.4, CH_3
14	3.57, dd (11.2, 6.0)	60.6, CH_2
	3.62, dd (11.2, 3.5)	
4-OH	4.07, brs	
14-OH	3.09, brs	

The NMR data of **4** correspond well to those of penihydrone published previously.[15,16]

[a] Signals overlapping with each other.

Table S11. NMR data of penihydrone B (**5**) in acetone-d_6.

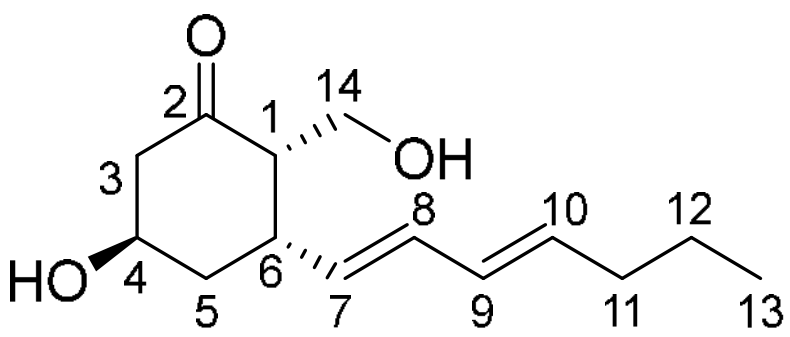

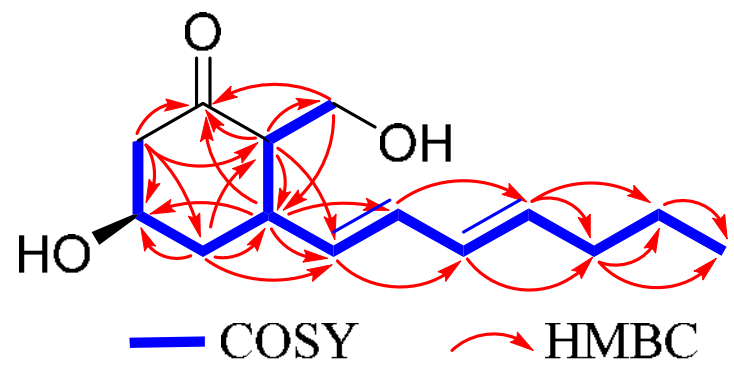

Position	δ_H, multi (*J* in Hz)	δ_C, type	Key HMBC correlations
1	2.70, dd (6.7, 5.2)	56.2, CH	C-2, C-6, C-7, C-14
2		209.8, C	
3	2.43, dd (13.1, 9.0)	51.2, CH_2	C-2, C-4, C-5
	2.63, dd (13.1, 4.6)		C-1, C-2, C-4, C-5
4	4.17, m	67.6, CH	
5	1.93, ddd (13.2, 9.2, 4.3)	40.0, CH_2	C-1, C-3, C-4, C-6, C-7
	2.10, dddd (13.2, 5.5, 3.8, 1.6)		C-1, C-3, C-4, C-6
6	3.07, m	39.3, CH	C-1, C-2, C-4, C-7, C-8
7	5.36, dd (14.7, 9.2)	130.9, CH	C-1, C-6, C-9
8	6.11, dd (14.7, 10.4)	133.1, CH	C-6, C-10
9	6.00, ddt (14.8, 10.4, 1.6)	131.1, CH	C-7, C-11
10	5.62, dt (14.8, 7.0)	134.2, CH	C-8, C-11, C-12
11	2.02, q (7.4)	35.3, CH_2	C-9, C-10, C-12, C-13
12	1.38, sext. (7.4)	23.1, CH_2	C-10, C-11, C-13
13	0.88, t (7.4)	13.9, CH_3	C-11, C-12
14	3.44[a], m	60.1, CH_2	C-1, C-2, C-6
	3.83, m		C-1, C-2, C-6
4-OH	4.14, s		
14-OH	3.41[a], s		

[a] Signals overlapping with each other.

Table S12. NMR data of palitanol A (**6**) in DMSO-d_6.

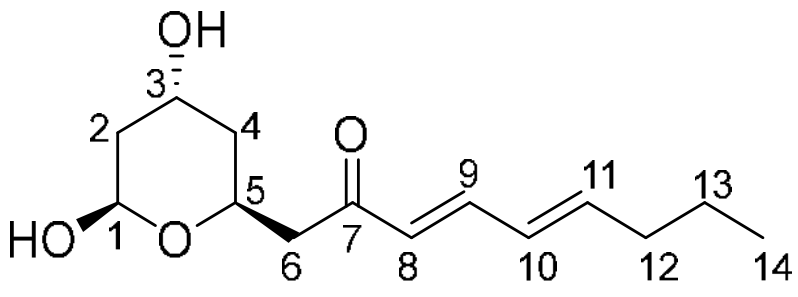

6 (a)

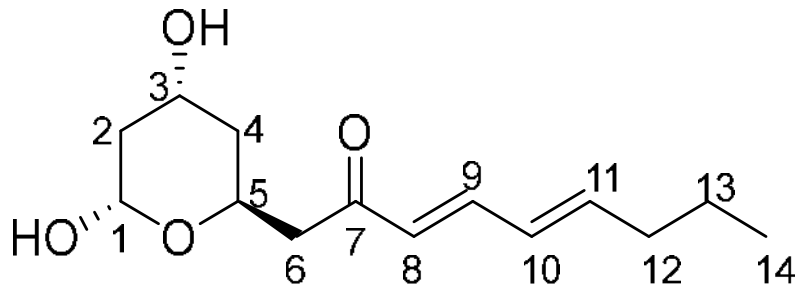

anomer-6 (b)

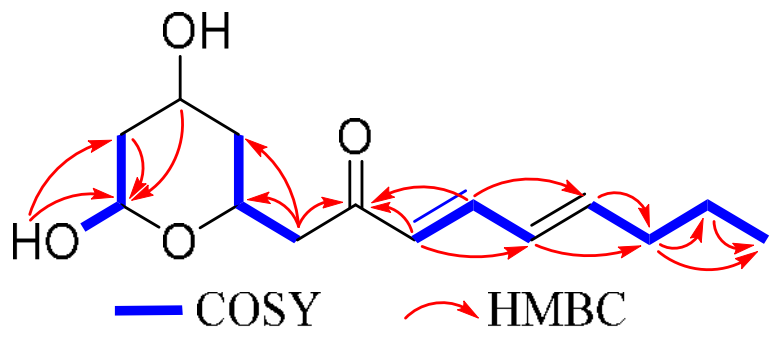

Position	δ_H, multi (*J* in Hz) **6** (a, major)	δ_H, multi (*J* in Hz) **anomer-6** (b, minor)	δ_C, type	Key HMBC correlations
1	4.84, m	4.98, m	91.5, CH	
2	1.28, m	1.28, m	39.8, CH_2	C-1
	1.65, m	1.65, m		
3	4.03, m	3.97, m	63.5, CH	C-1
4,	1.26, m	1.54, m	37.6, CH_2	
	1.48, dd (13.4, 2.7)	1.69, m		
5	4.22, m	4.55, m	66.6, CH	
6	2.51, dd (15.6, 5.1)	2.60	45.9, CH_2	C-5, C-7
	2.77, dd (15.6, 8.0)	2.77, dd (15.3, 8.0)		C-4, C-5, C-7
7			198.1, C	
8	6.13, d (15.6)	6.13, d (15.6)	128.5, CH	C-7, C-10
9	7.17, dd (15.6, 9.6)	7.17, dd (15.6, 9.6)	143.1, CH	C-7, C-11
10	6.28, m	6.28, m	129.1, CH	C-12
11	6.28, m	6.28, m	145.3, CH	C-9, C-12
12	2.13, m	2.13, m	34.4, CH_2	C-13, C-14
13	1.41, sext. (7.4)	1.41, sext. (7.4)	21.3, CH_2	C-12, C-14
14	0.88, t (7.4)	0.88, t (7.4)	13.5, CH_3	C-12, C-13
1-OH	6.28	6.28		C-2, C-1
3-OH	4.61, s	4.92, s		

Table S13. NMR data of palitanol B (**7**) in DMSO-*d*$_6$.

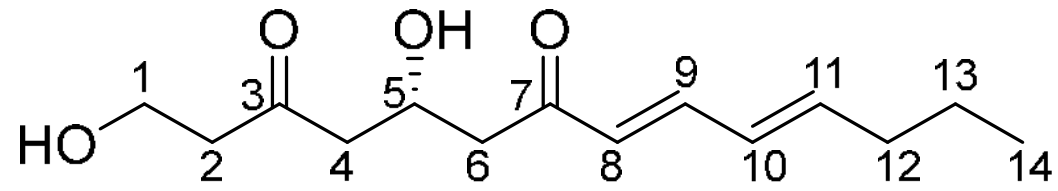

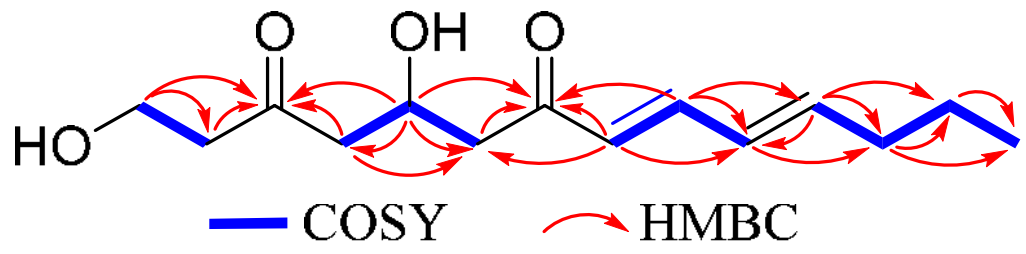

Position	δ_H, multi (*J* in Hz)	δ_C, type	Key HMBC correlations
1	3.63, t (6.3)	56.3, CH_2	C-2, C-3
2	2.56[a], m	46.1, CH_2	C-1, C-3
3		208.5, C	
4	2.53[a], m	50.2, CH_2	C-3, C-5, C-6
5	4.35, m	63.8, CH	C-3, C-4, C-6, C-7
6	2.60[a], dd (15.2, 5.0)	47.2, CH_2	C-4, C-5, C-7
	2.69[a], dd (15.2, 7.6)		C-4, C-5, C-7
7		198.6, CH	
8	6.11, d (15.6)	128.5, CH	C-6, C-7, C-10
9	7.18, dd (15.6, 9.9)	143.1, CH	C-7, C-10, C-11
10	6.27[b], m	129.1, CH	C-12
11	6.27[b], m	145.2, CH	C-9, C-10, C-12, C-13
12	2.14, q (7.4)	34.4, CH_2	C-13, C-14
13	1.42, sext. (7.4)	21.3, CH_2	C-12, C-14
14	0.88, t (7.4)	13.4, CH_3	C-12, C-13
1-OH	4.52, s		
5-OH	4.75, s		

[a, b] Signals with the same letter overlapping with each other.

Table S14. NMR data of penihydrone C (**8**) in acetone-d_6.

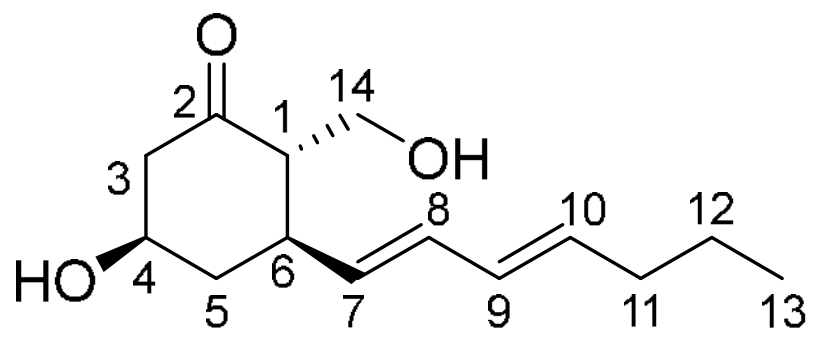

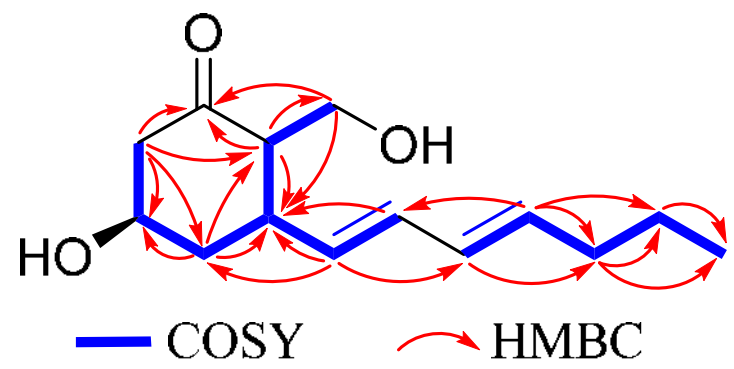

Position	δ_H, multi (*J* in Hz)	δ_C, type	Key HMBC correlations
1	2.25, ddd (12.0, 5.8, 2.4)	56.1, CH	C-2, C-6, C-14
2		210.0, C	
3	2.44, ddd (12.9, 11.4, 1.1)	52.1, CH_2	C-2, C-4, C-5
	2.59, ddd (12.9, 4.8, 2.4)		C-1, C-2, C-4, C-5
4	3.84, m	68.7, CH	
5	1.72, m	42.4, CH_2	C-1, C-3, C-4, C-6
	2.10, m		
6	2.33, ddd (12.0, 8.5, 3.5)	39.3, CH	C-1
7	5.53, dd (14.5, 8.5)	134.3, CH	C-5, C-6, C-9
8	6.07[a], m	132.1, CH	C-6
9	6.07[a], m	131.1, CH	C-11
10	5.64, dt (14.5, 7.0)	134.1, CH	C-8, C-11, C-12
11	2.03[b], m	35.3, CH_2	C-9, C-10, C-12, C-13
12	1.40, sext. (7.4)	23.1, CH_2	C-10, C-11, C-13
13	0.89, t (7.4)	14.0, CH_3	C-11, C-12
14	3.55, dd (11.3, 5.8)	59.3, CH_2	C-1, C-2
	3.73, d (11.3)		C-1, C-2, C-6
4-OH	4.28, s		
14-OH	3.06, s		

[a] Signals overlapping with each other. [b] Overlapping with the solvent signal.

Table S15. NMR data of palitanol C (**9**) in DMSO-d_6.

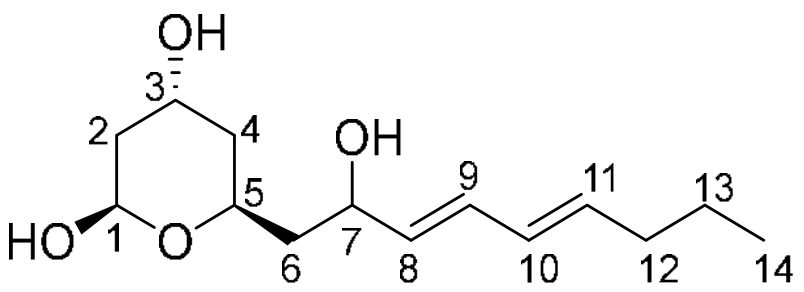

9 (a)

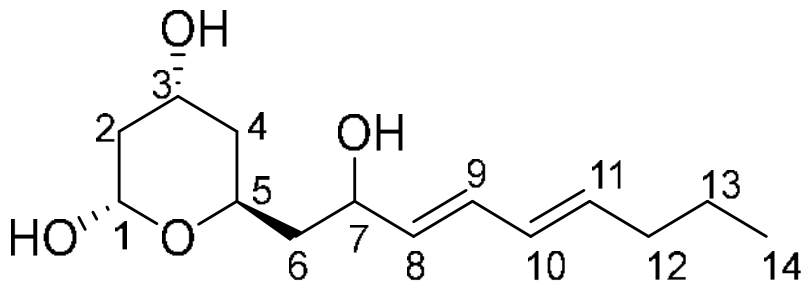

anomer-9 (b)

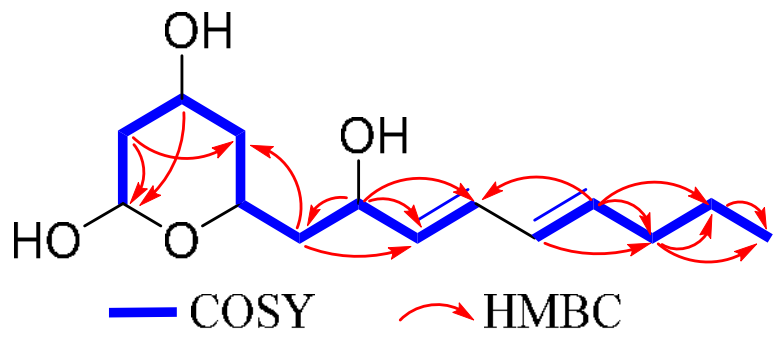

Position	**9** (a, major)		**anomer-9** (b, minor)		Key HMBC correlations
	δ_H, multi (*J* in Hz)	δ_C, type	δ_H, multi (*J* in Hz)	δ_C, type	
1	4.83, dd (9.8, 2.1)	91.6, CH	4.83, dd (9.8, 2.1)	91.7, CH	
2	1.31, m	39.9, CH_2	1.31, m	39.9, CH_2	C-1, C-4
	1.66, m		1.66, m		
3	4.02, m	63.6, CH	4.02, m	63.6, CH	C-1
4	1.23, m	37.9, CH_2	1.23, m	37.9, CH_2	C-2
	1.48, m		1.48, m		
5	3.76, m	69.4, CH	3.80, m	67.2, CH	
6	1.36, m	43.7, CH_2	1.36, m	43.7, CH_2	C-4, C-8
	1.65, m		1.65, m		
7	4.12, m	67.9, CH	3.94, m	70.1, CH	C-6, C-8, C-9
8	5.55, dd (15.1, 6.7)	135.3, CH	5.55, dd (15.1, 6.7)	136.2, CH	C-6
9	6.08, m	129.0, CH	6.08, m	128.6, CH	
10	6.01, m	130.1, CH	6.01, m	131.7, CH	C-12
11	5.63, dd (15.0, 6.7)	133.3, CH	5.63, dd (15.0, 6.7)	129.7, CH	C-9, C-12, C-13
12	2.02, m	34.0, CH_2	2.02, m	34.0, CH_2	C-10, C-11, C-13, C-14
13	1.36, m	21.9, CH_2	1.36, m	21.9, CH_2	C-11, C-14
14	0.87, m	13.9, CH_3	0.87, m	13.5, CH_3	C-13

Note: Most ^{1}H and ^{13}C NMR signals of the two anomers overlapped with each other.

Table S16. NMR data of penihydronal (**12**) in $CDCl_3$.

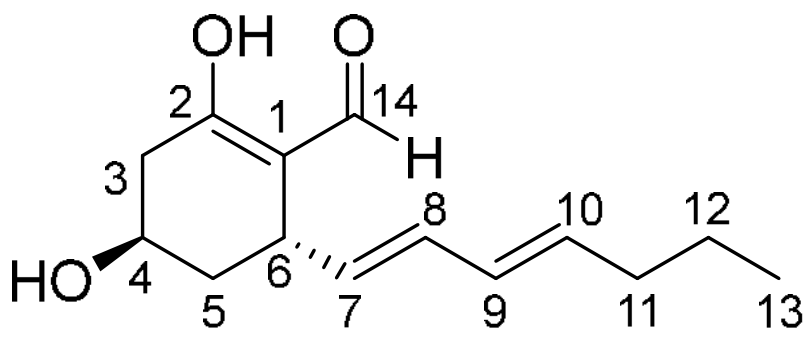

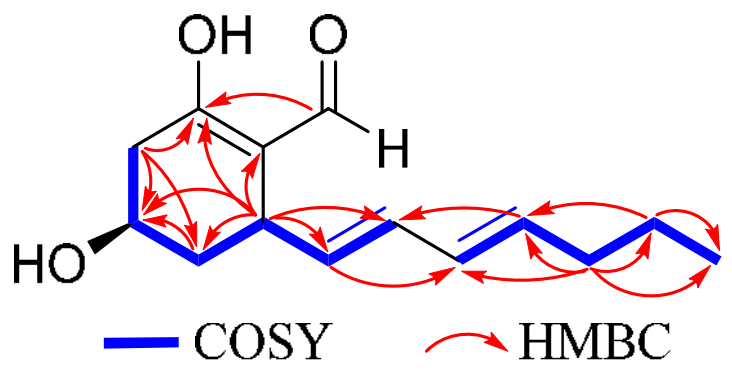

Position	δ_H, multi (*J* in Hz)	δ_C, type	Key HMBC correlations
1		110.2, C	
2		182.1, C	
3	2.39, dd (18.7, 6.6)	40.1, CH_2	C-2, C-4, C-5
	2.71, dd (18.7, 5.1)		C-2, C-5
4	4.16, m	63.4, CH	
5	1.80, ddd (13.0, 6.4, 2.5)	37.4, CH_2	
	1.86, ddd (13.0, 8.6, 5.8)		C-4
6	3.42, m	34.7, CH	C-1, C-2, C-4, C-5, C-7, C-8
7	5.52, dd (14.0, 7.4)	133.9, CH	C-9
8	6.01[a], m	132.2, CH	
9	6.01[a], m	129.6, CH	
10	5.65, dt (14.0, 7.0)	134.9, CH	C-8
11	2.05, q (7.3)	34.9, CH_2	C-9, C-10, C-12, C-13
12	1.41, sext. (7.3)	22.6, CH_2	C-10, C-11, C-13
13	0.90, t (7.3)	13.9, CH_3	C-11, C-12
14	8.71, s	189.5, CHO	C-2
2-OH	14.61, s		
4-OH	n. d.		

[a] Signals overlapping with each other. n. d.: Signals not detected.

Table S17. NMR data of dia-palitantol (**16**) in DMSO-d_6.

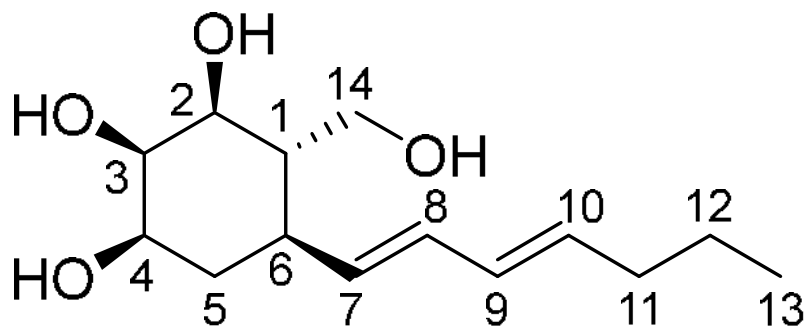

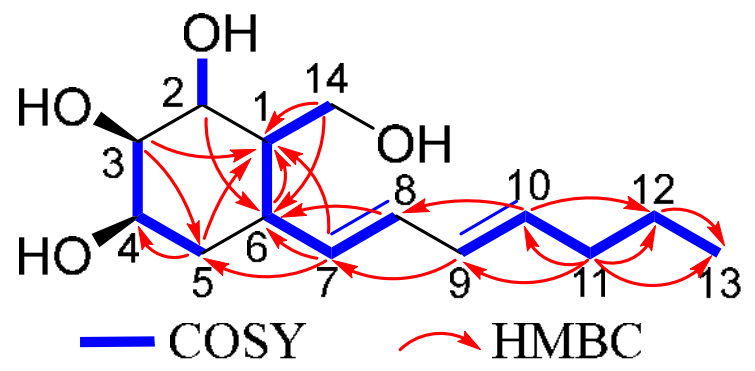

Position	δ_H, multi (*J* in Hz)	δ_C, type	Key HMBC correlations
1	1.50, ddd (12.8, 6.0, 3.3)	41.2, CH	C-14
2	3.88, dd (3.3)	69.6, CH	C-6
3	3.60, dd (3.3)	71.8, CH	C-1, C-5
4	3.70, m	66.2, CH	
5	1.31, m	35.3, CH_2	C-1, C-3, C-4
	1.44, d (11.9)		C-4, C-1
6	2.08, m	35.9, CH	C-1
7	5.36, dd (14.4, 8.9)	136.1, CH	C-1, C-5, C-6, C-9
8	5.93[a], m	129.2, CH	C-6, C-10
9	5.93[a], m	130.5, CH	C-7, C-11
10	5.54, dt (14.4, 7.0)	131.7, CH	C-8, C-11, C-12
11	1.99, m	34.0, CH_2	C-9, C-10, C-12, C-13
12	1.36, sext. (7.4)	22.0, CH_2	C-10, C-11, C-13
13	0.86, t (7.4)	13.5, CH_3	C-11, C-12
14	3.35, d (6.0)	60.4, CH_2	C-1, C-6
2-OH	4.53, s		
3-OH	4.33, s		
4-OH	4.14[b], s		
14-OH	4.14[b], s		

[a, b] Signals with the same letter overlapping with each other.

Supplementary Figures

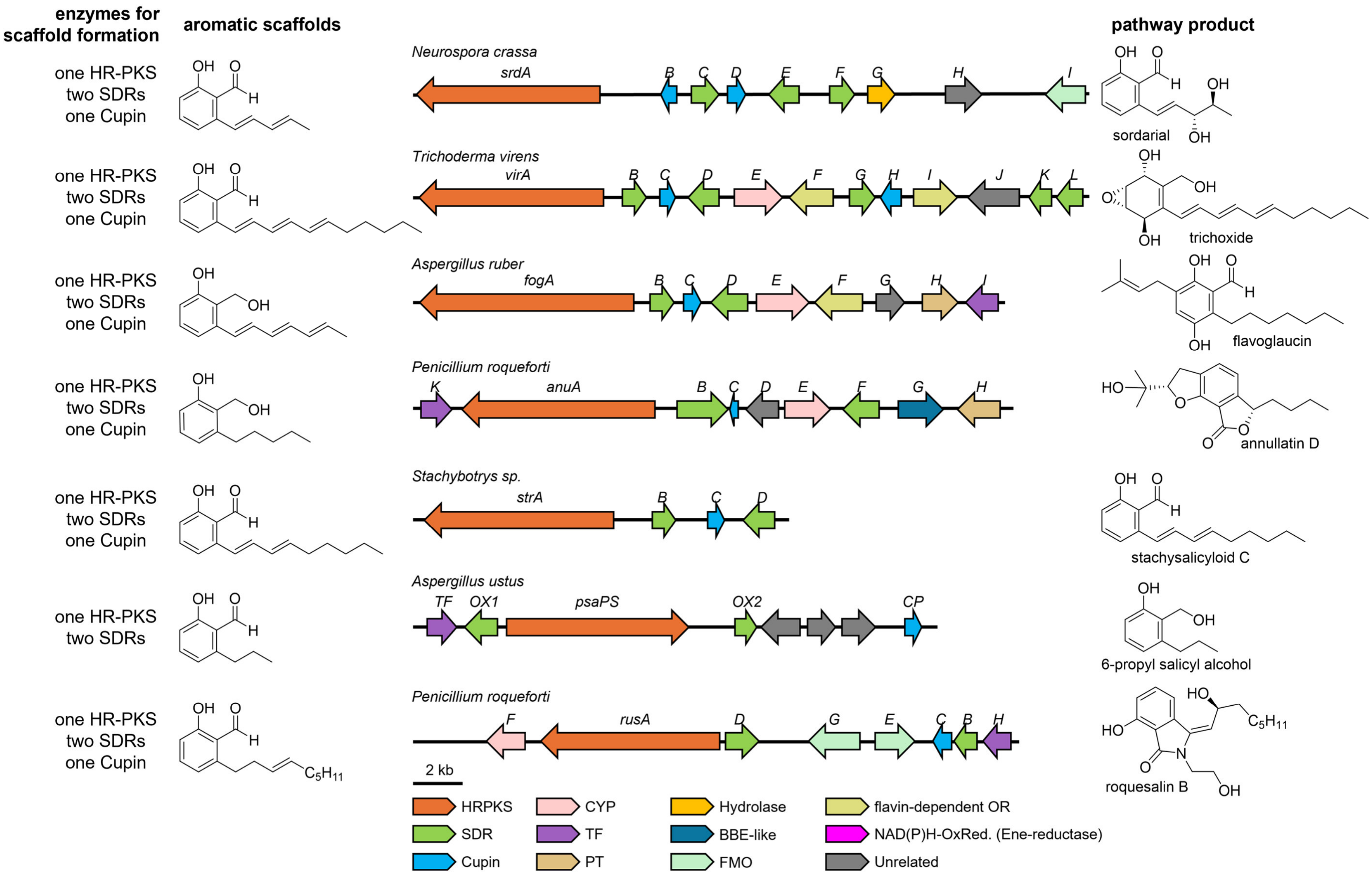

Figure S1. Reported biosynthetic gene clusters for fungal alkyl salicylaldehyde derivatives and the aromatic salicylaldehyde cores formed by a single HR-PKS, two SDRs, with or without a cupin protein.

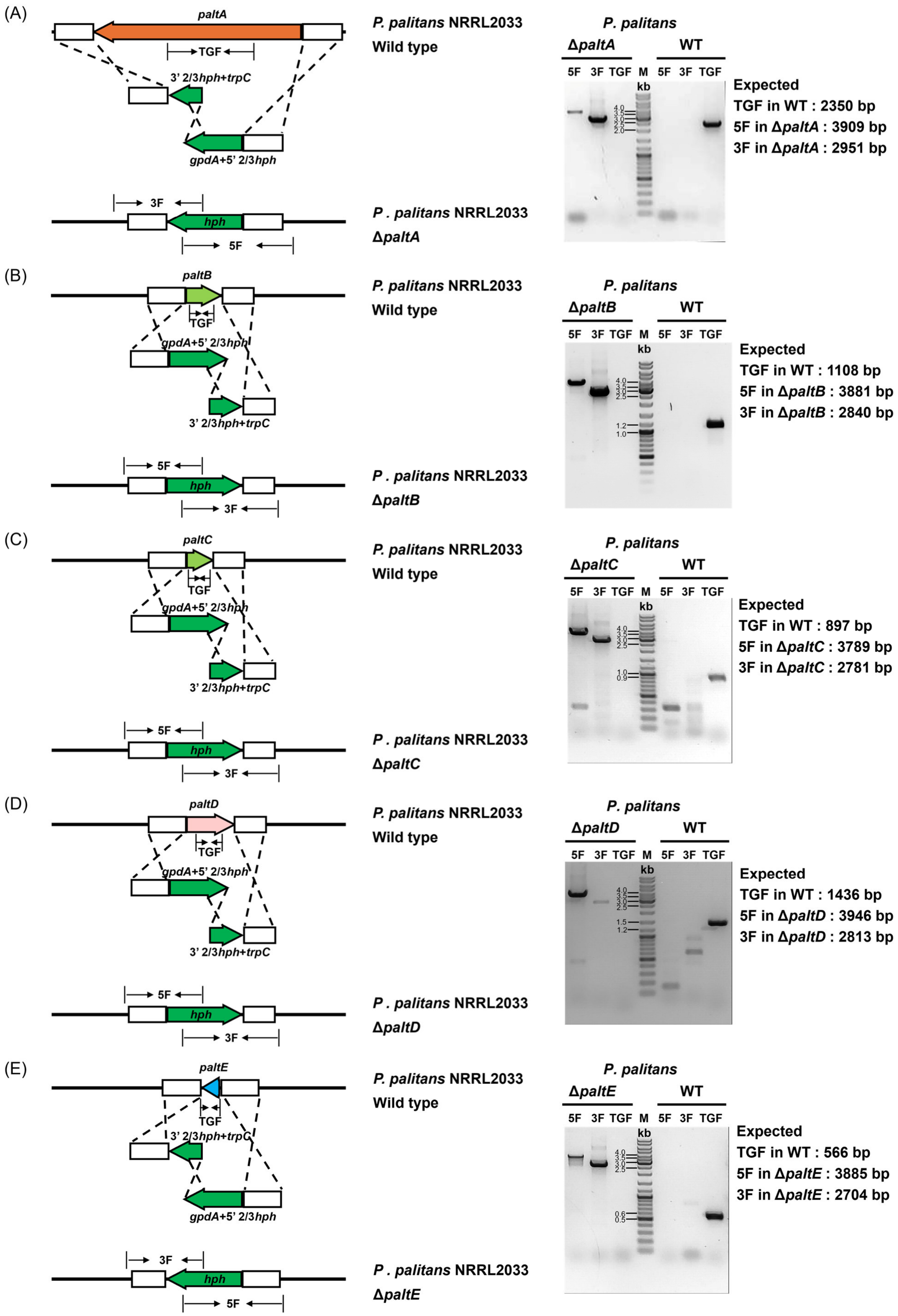

Figure S2. Schematic illustration of target gene deletion and PCR verification of *ΔpaltA*–*ΔpaltE* mutant strains in *P. palitans*.

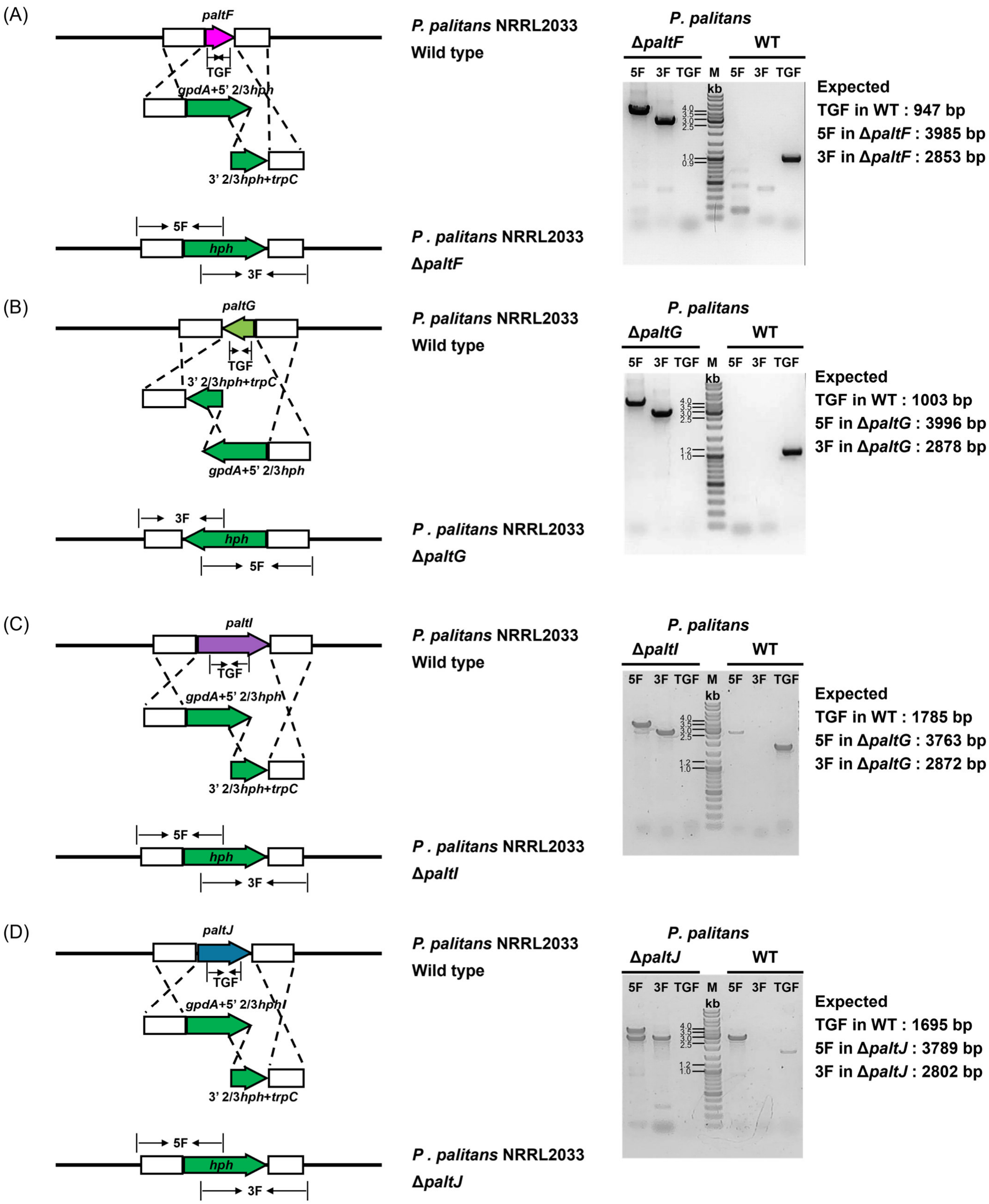

Figure S3. Schematic illustration of target gene deletion and PCR verification of △*paltF*, △*paltG*, △*paltI*, and △*paltJ* mutant strains in *P. palitans*.

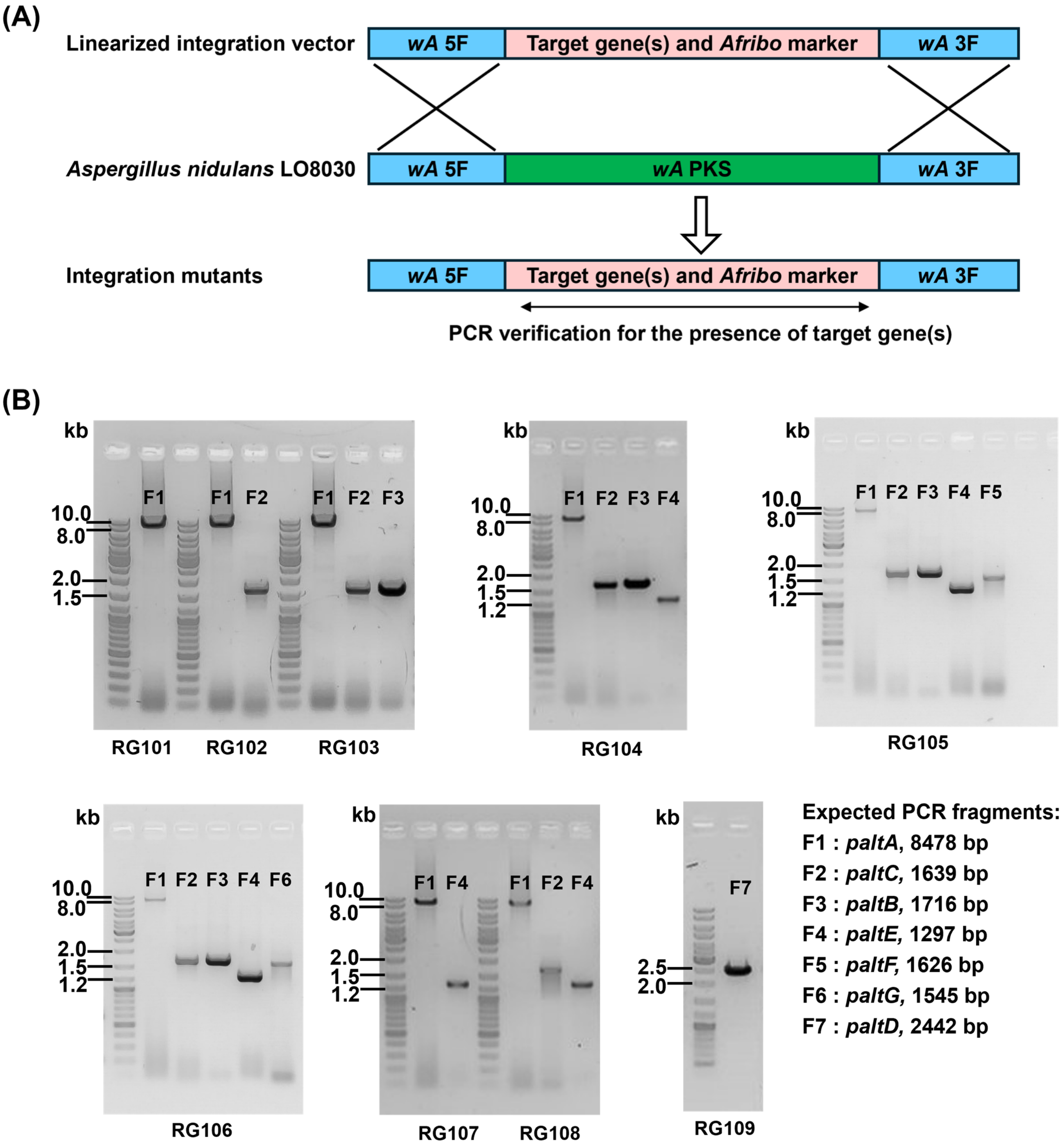

Figure S4. Heterologous expression and PCR verification for *A. nidulans* strains.

Schematic representation of gene integration into the *wA*-PKS locus of *A. nidulans* LO8030. (B) PCR verifications for the presence of the target gene(s) were performed with genomic DNA of the *A. nidulans* transformants using primers listed in Table S6.

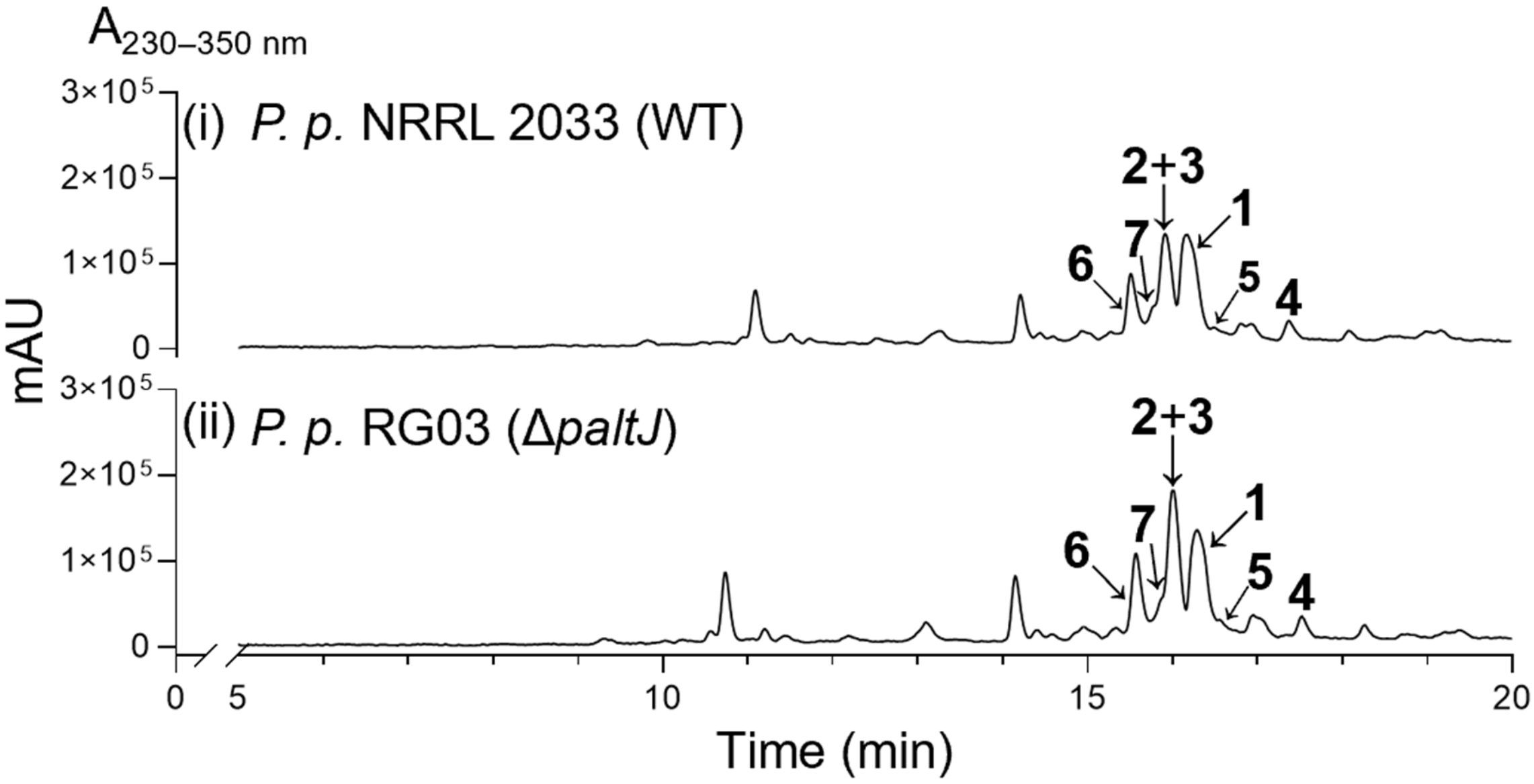

Figure S5. HPLC analysis of metabolites in WT strain and Δ*paltJ* mutant RG03.

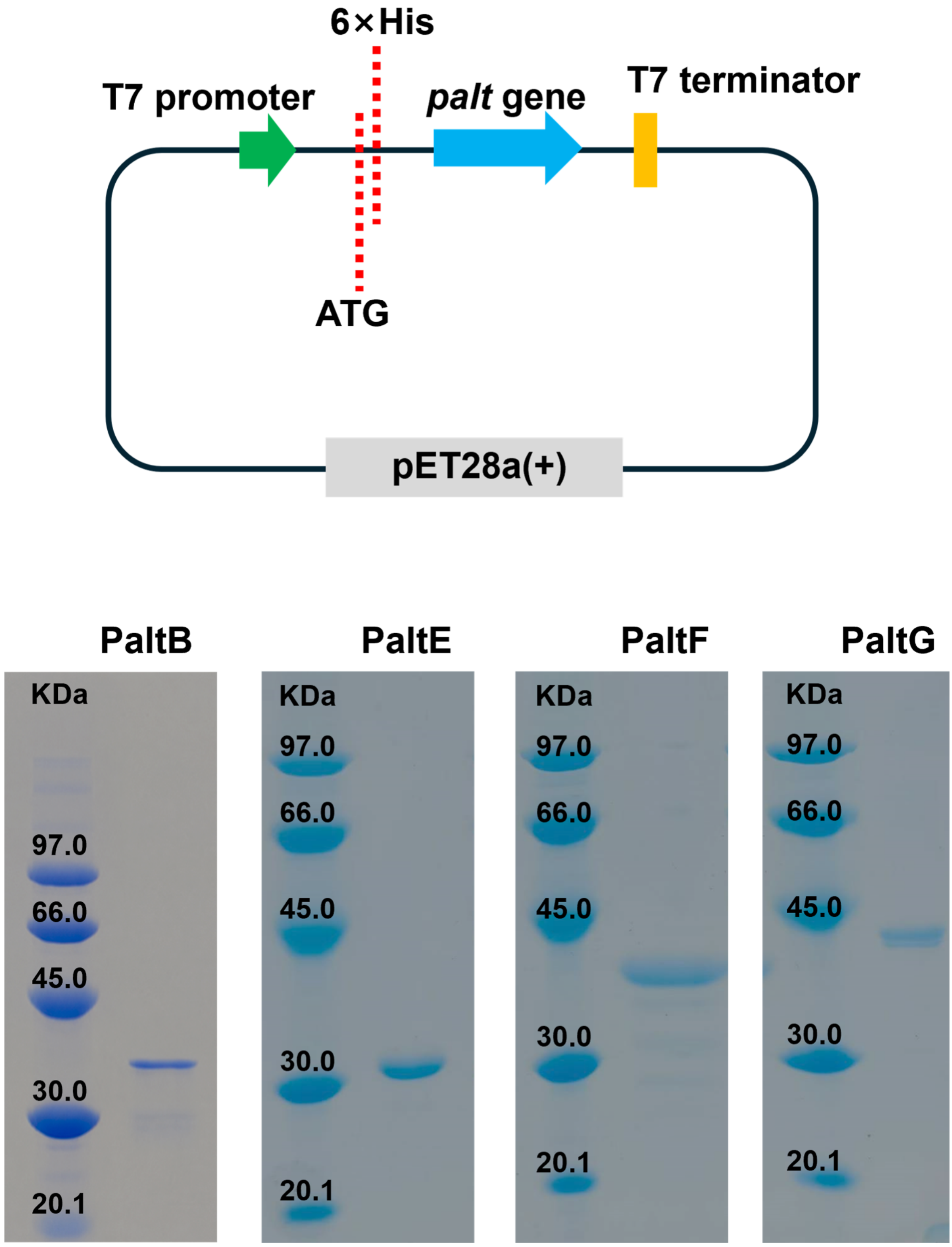

Figure S6. SDS-PAGE (12%) analysis of purified recombinant N-terminally His_6-tagged PaltB, PaltE, PaltF, and PaltG.

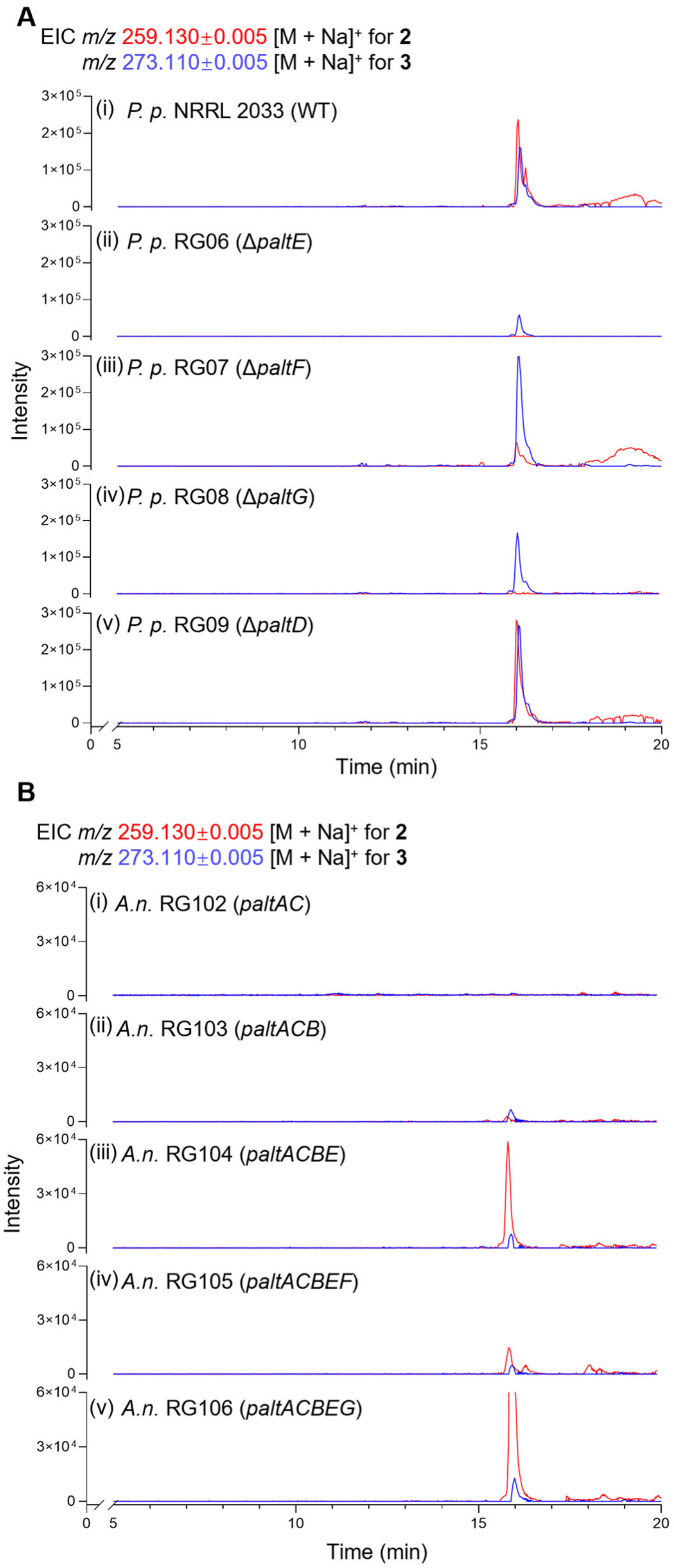

Figure S7. Extracted ion chromatograms of the overlapping peaks assigned to **2** and **3** in *P. palitans* strains (A) and *A. nidulans* transformants (B).

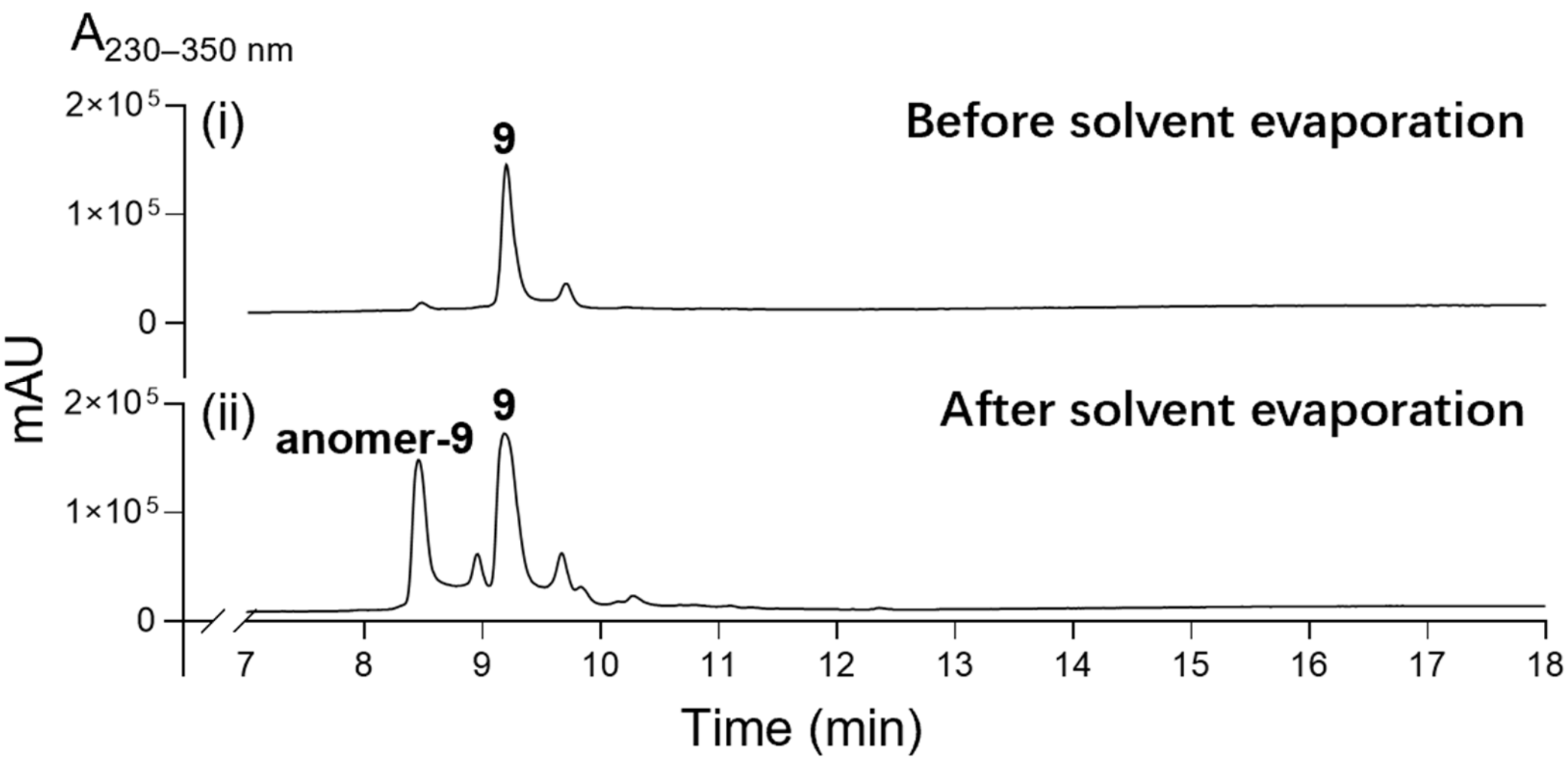

Figure S8. HPLC analysis of **9** before (i) and after evaporation (ii).

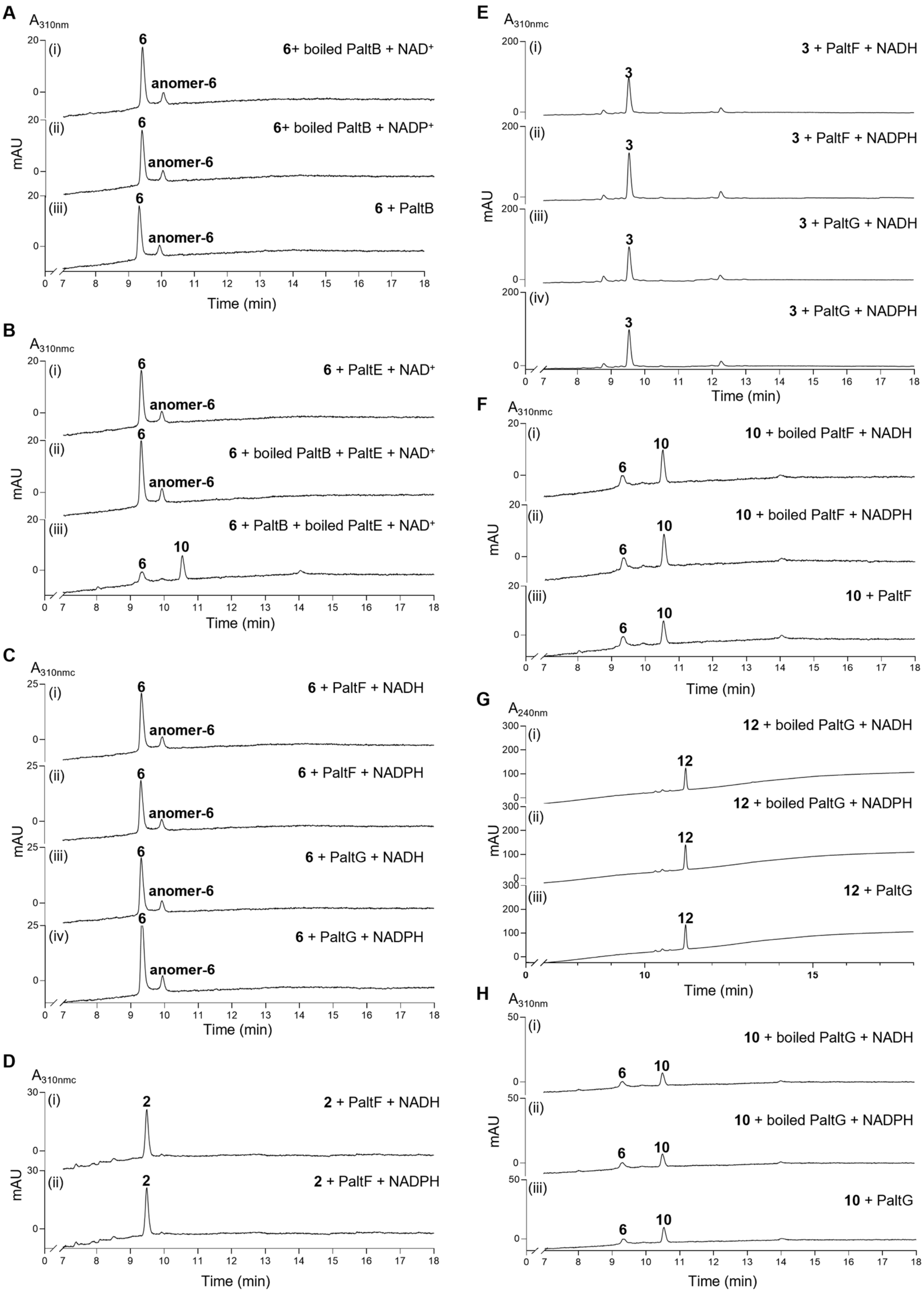

Figure S9. HPLC analysis of control assays with different enzymes.

Chromatograms with UV detection for assays of **6** with PaltB (A); **6** with PaltB and PaltE (B); **6** with PaltF or PaltG (C); **2** with PaltF (D); **3** with PaltF or PaltG (E); **10** with PaltF (F); **12** with PaltG (G); **10** with PaltG (H). Enzyme inactivation was carried out by boiling the recombinant proteins for 10 min.

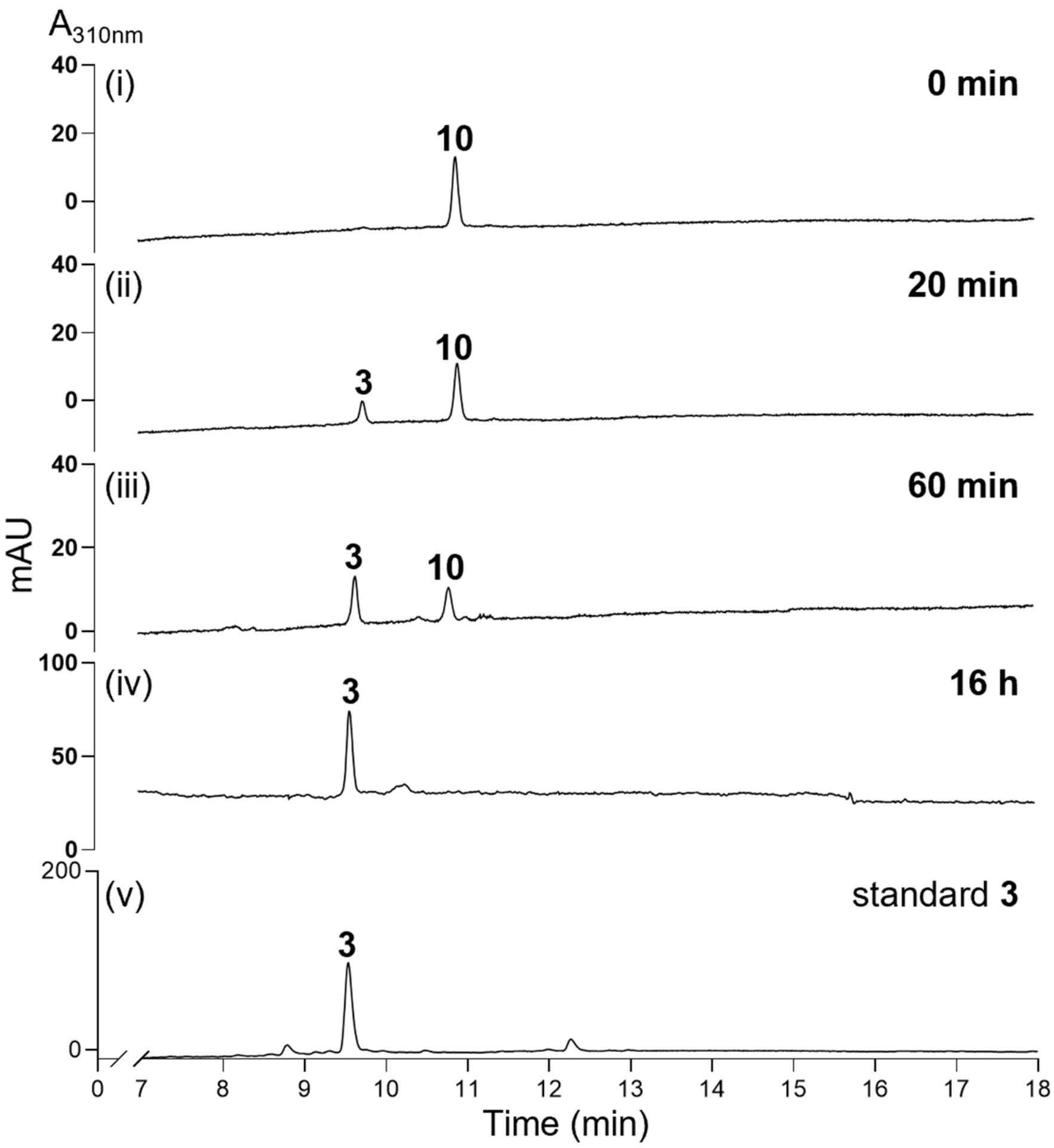

Figure S10. LC-MS analysis of the conversion of the isolated **10** to **3** over time.

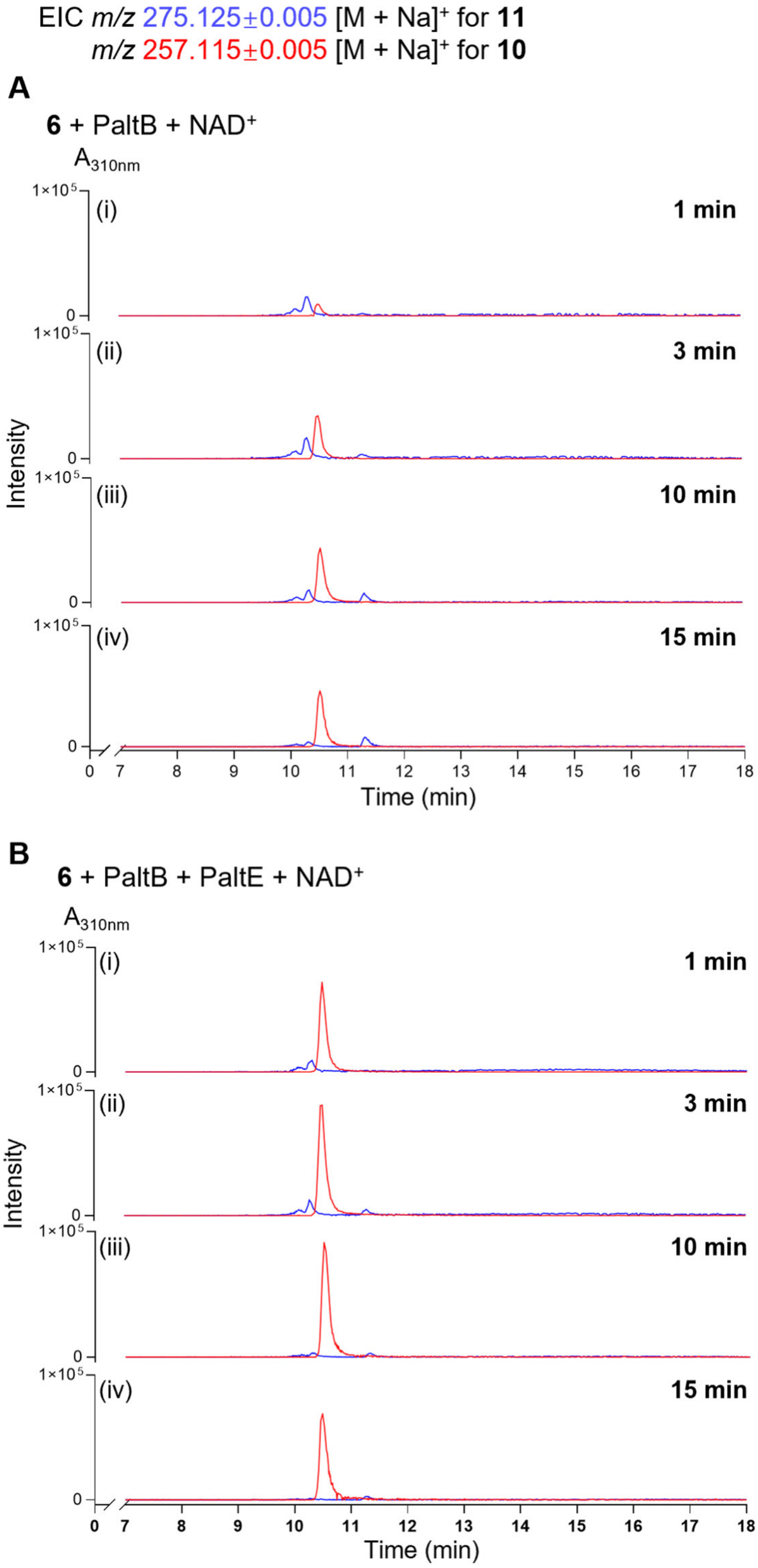

Figure S11. Extracted ion chromatograms of **10** and **11** in the incubation mixture of **6** with PaltB (A) and with PaltB and PaltE (B).

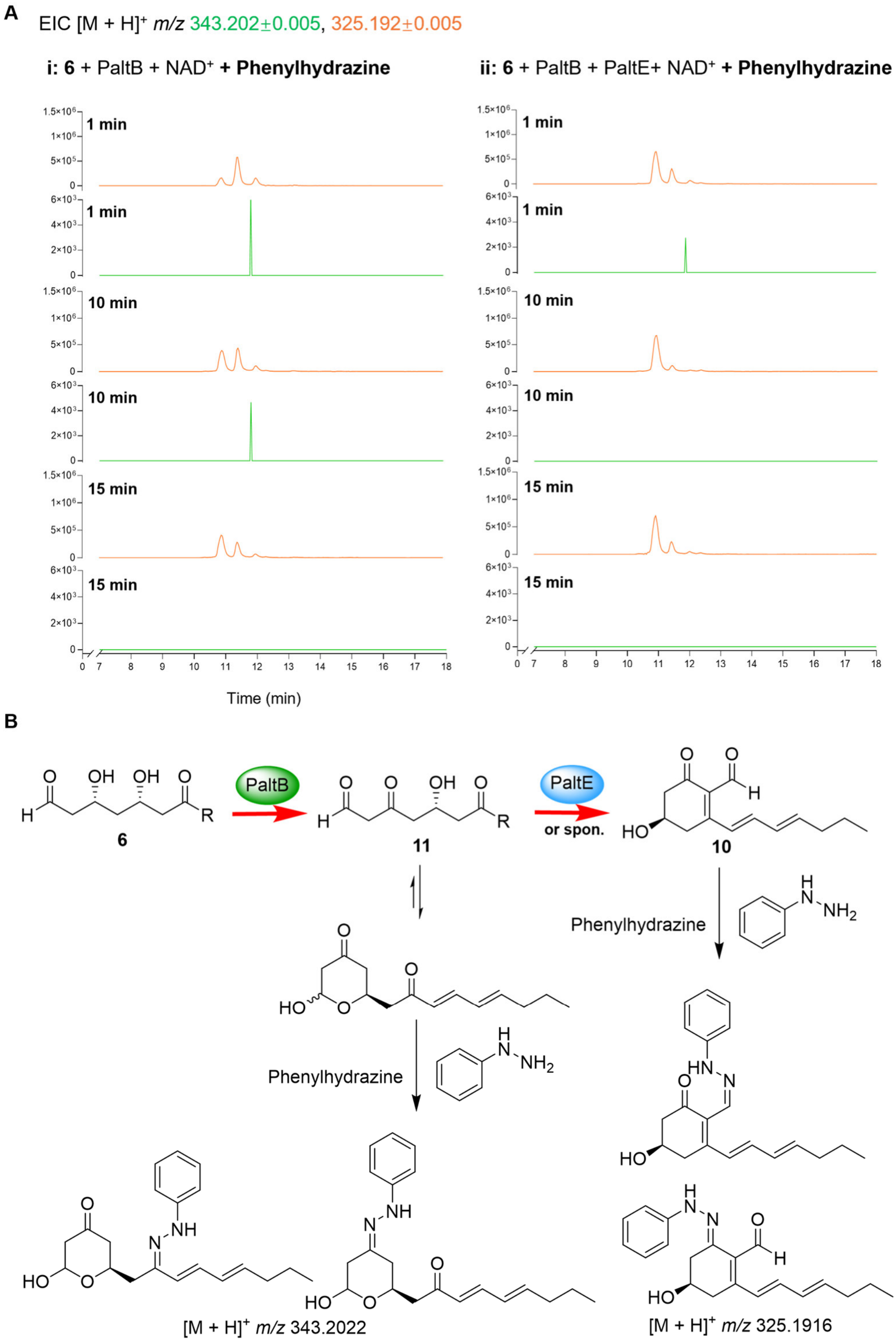

Figure S12. (A) Extracted ion chromatograms of the phenylhydrazone derivatives. The reactions were quenched at different time points and followed by incubation with phenylhydrazine for 10 min: **6** with PaltB alone (Ai) and with PaltB and PaltE together (Aii). (B) The proposed mechanism of PaltB and PaltE-catalyzed reactions and the plausible phenylhydrazone derivatives.

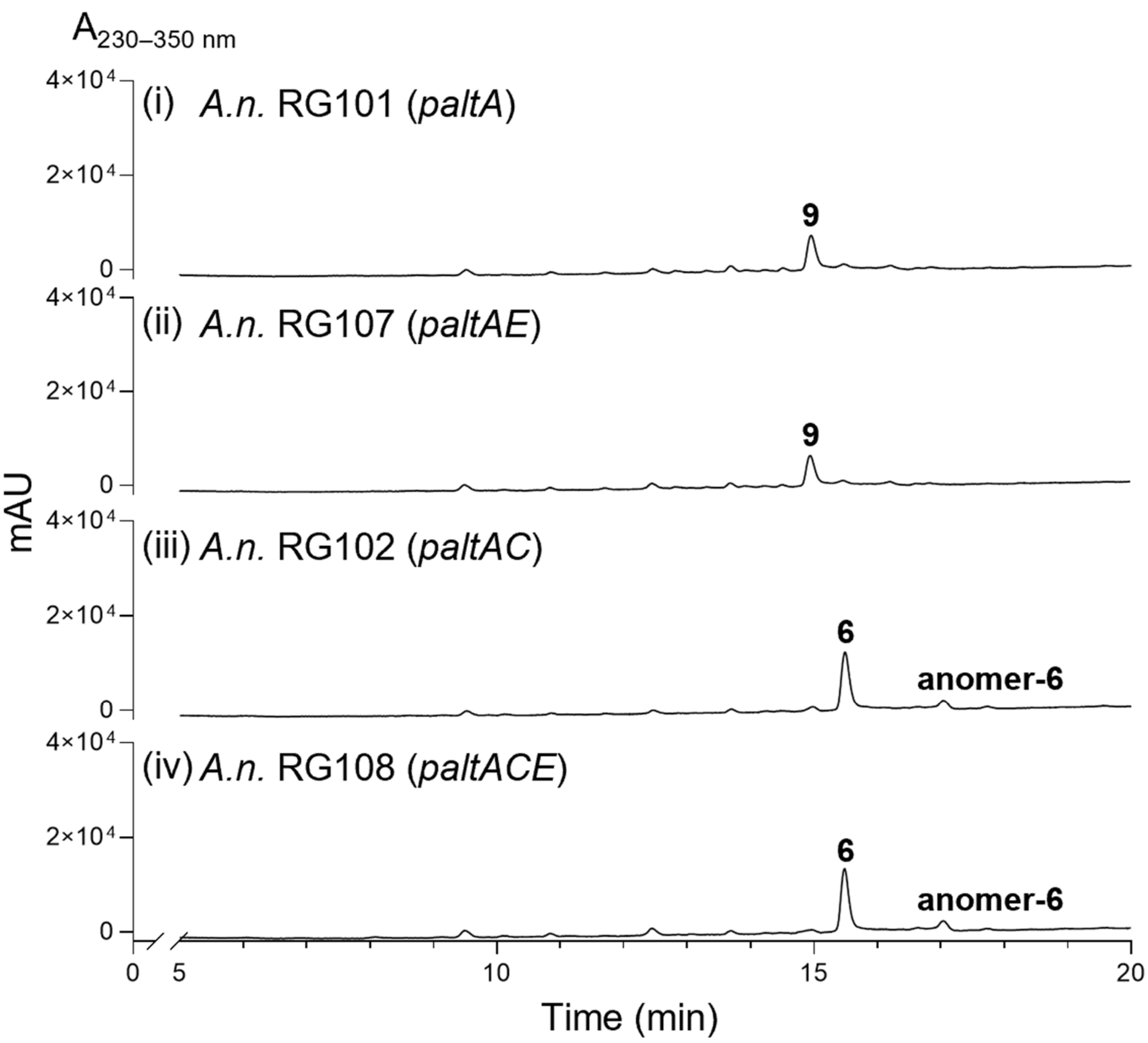

Figure S13. HPLC analysis of metabolites in *A. nidulans* transformants.

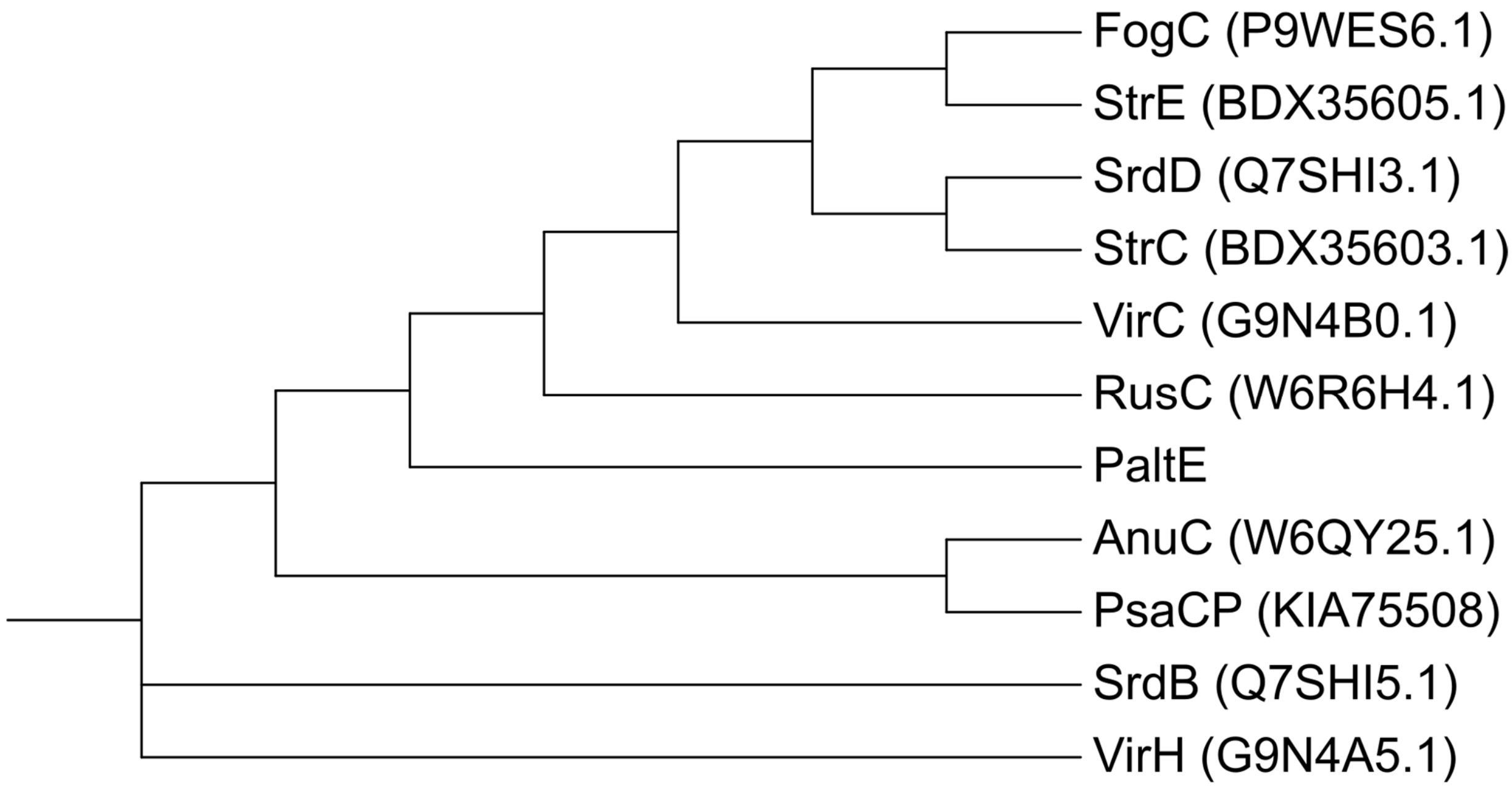

Figure S14. Phylogenetic analysis of PaltE and alkyl salicylaldehyde pathway-associated cupins. The phylogenetic tree was constructed in MEGA 12 using the maximum-likelihood method under the LG+G+I model with 1,000 bootstrap replicates and subsequently visualized using iTOL. Bootstrap values ≥70% are shown at the nodes.

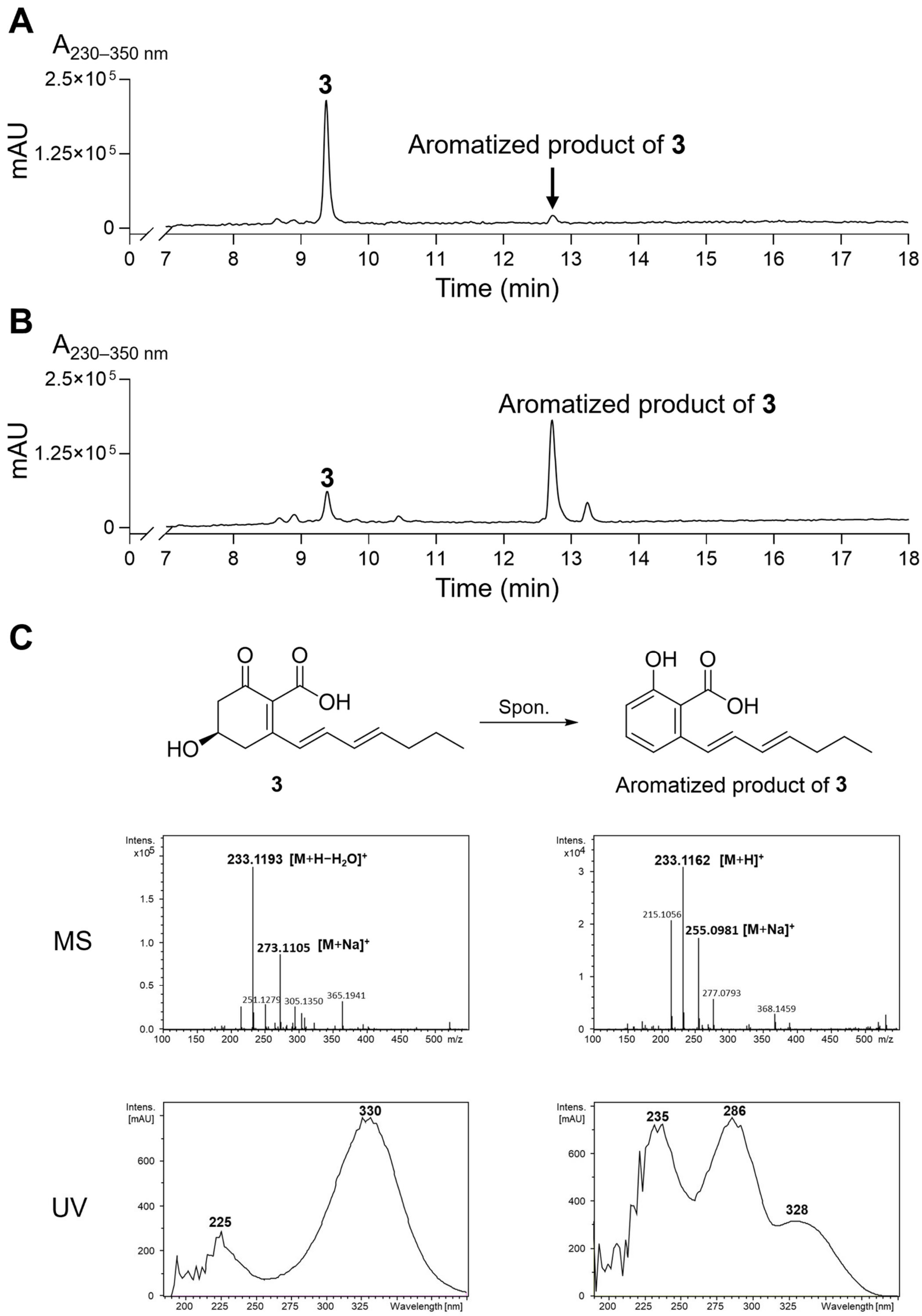

Figure S15. LC-MS analysis of the spontaneous aromatization of **3**.

(A) HPLC chromatogram of **3** before NMR measurement. (B) HPLC chromatogram of **3** recorded 7 days after NMR measurement, showing the formation of an aromatized product. (C) Structures, mass, and UV spectra of **3** and its aromatized product.

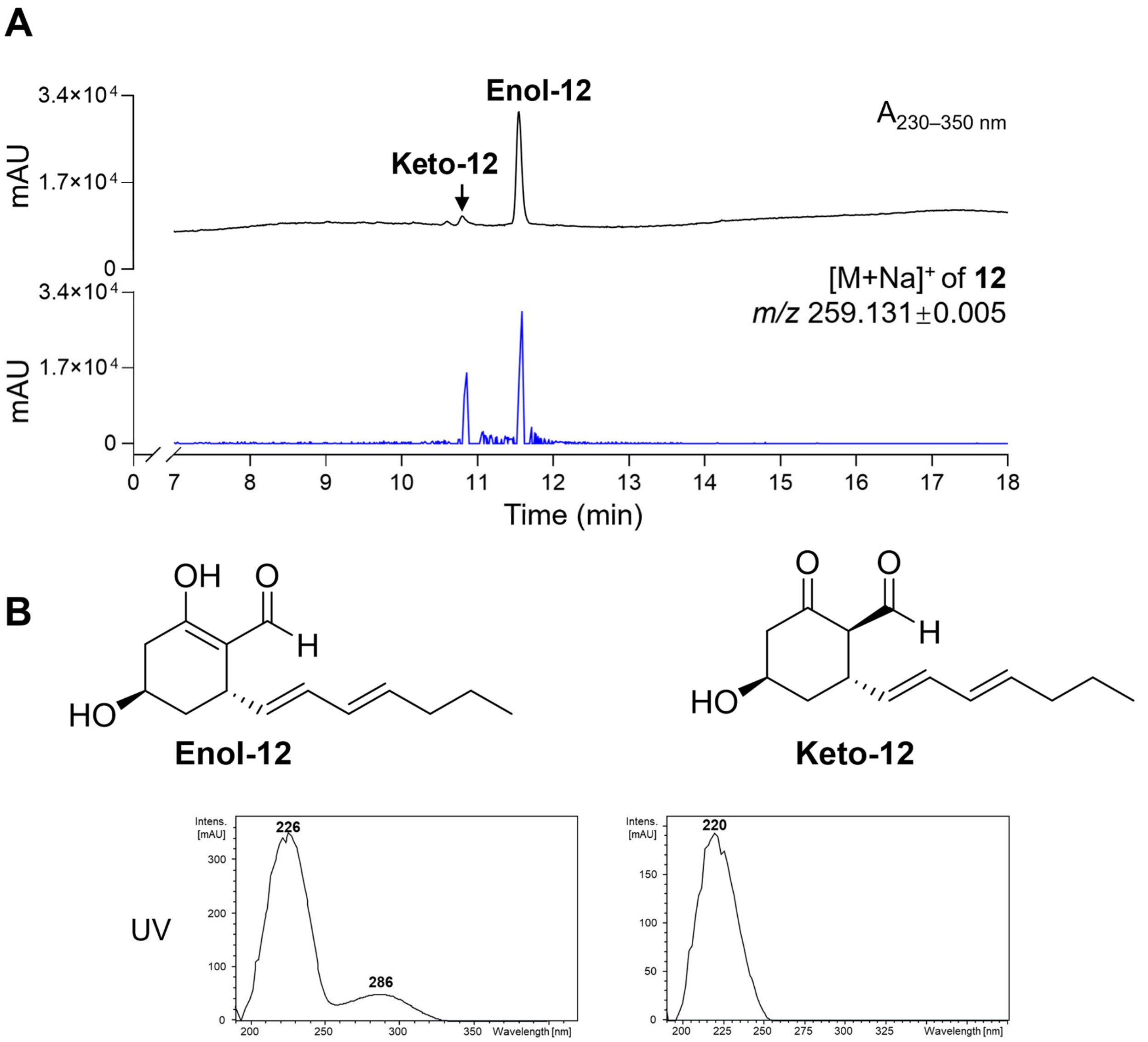

Figure S16. LC-MS analysis of **12**.

(A) UV absorption recorded from 230 to 350 nm. EICs refer $[M+Na]^+$ ions of **12** with a tolerance range of ± 0.005.

(B) Structures and UV spectra of **enol-12** and **keto-12**.

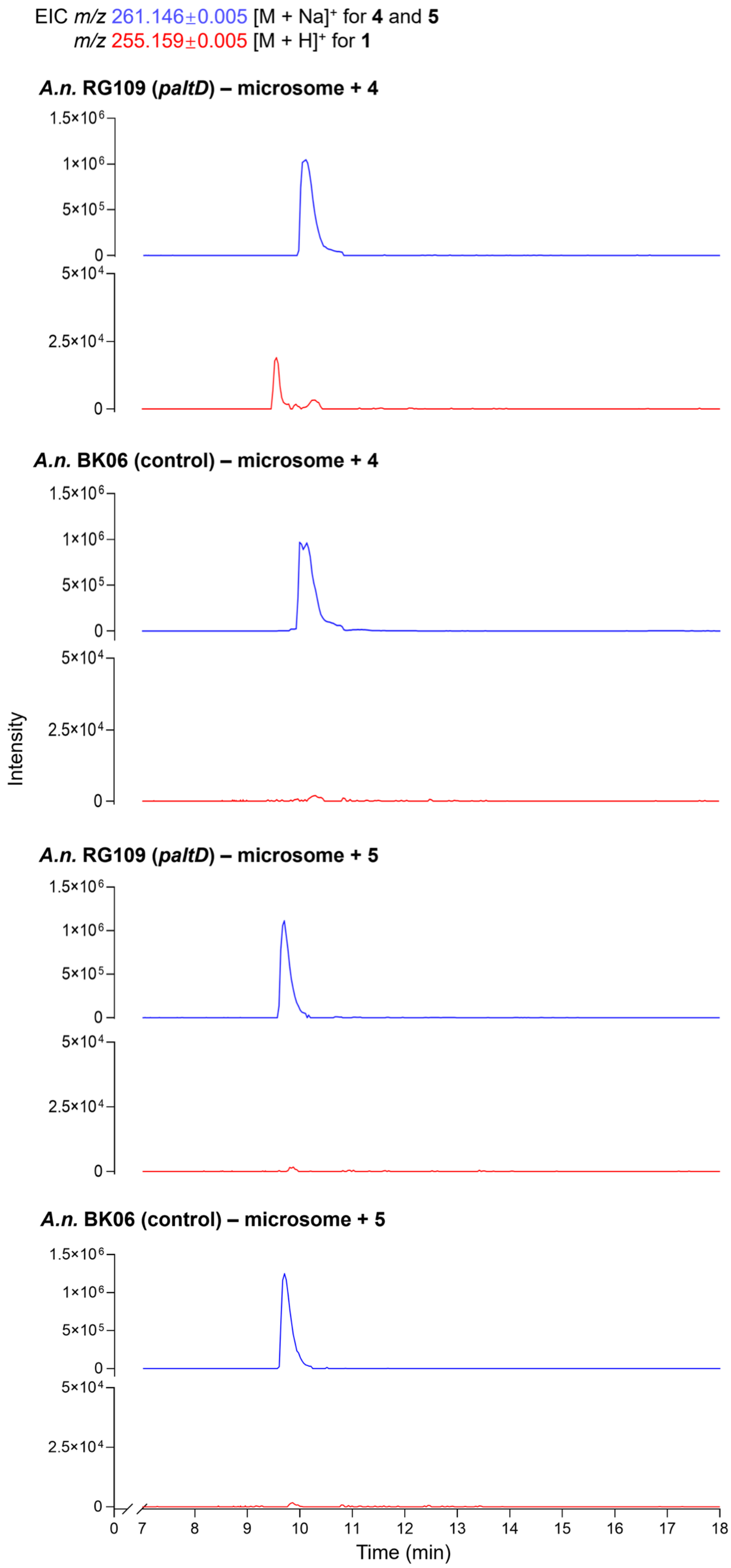

Figure S17. Extracted ion chromatograms of *in vitro* assays of **4** and **5** with microsomes from *A. nidulans* with or without PaltD.

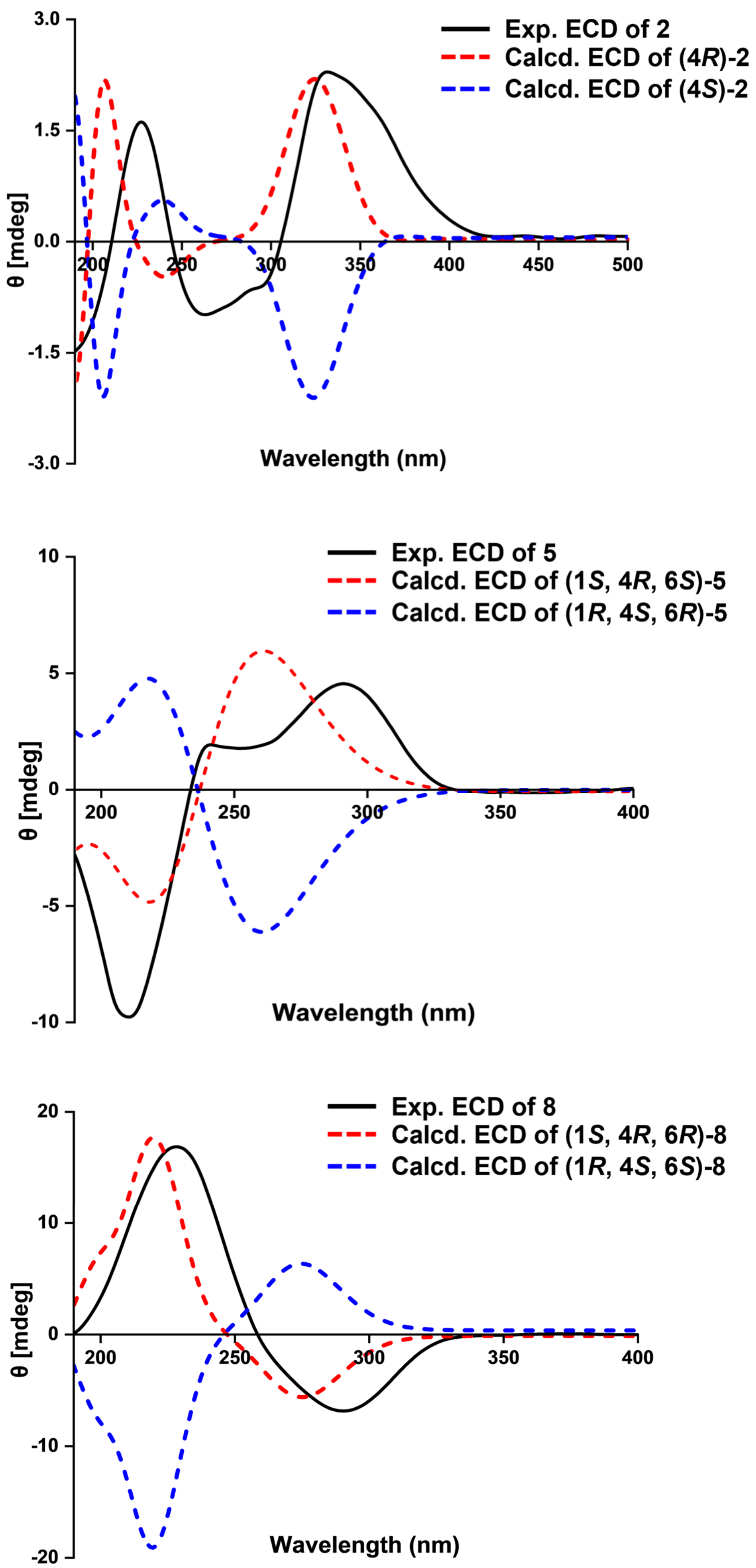

Figure S18. Experimental and calculated ECD spectra for **2**, **5**, and **8**.

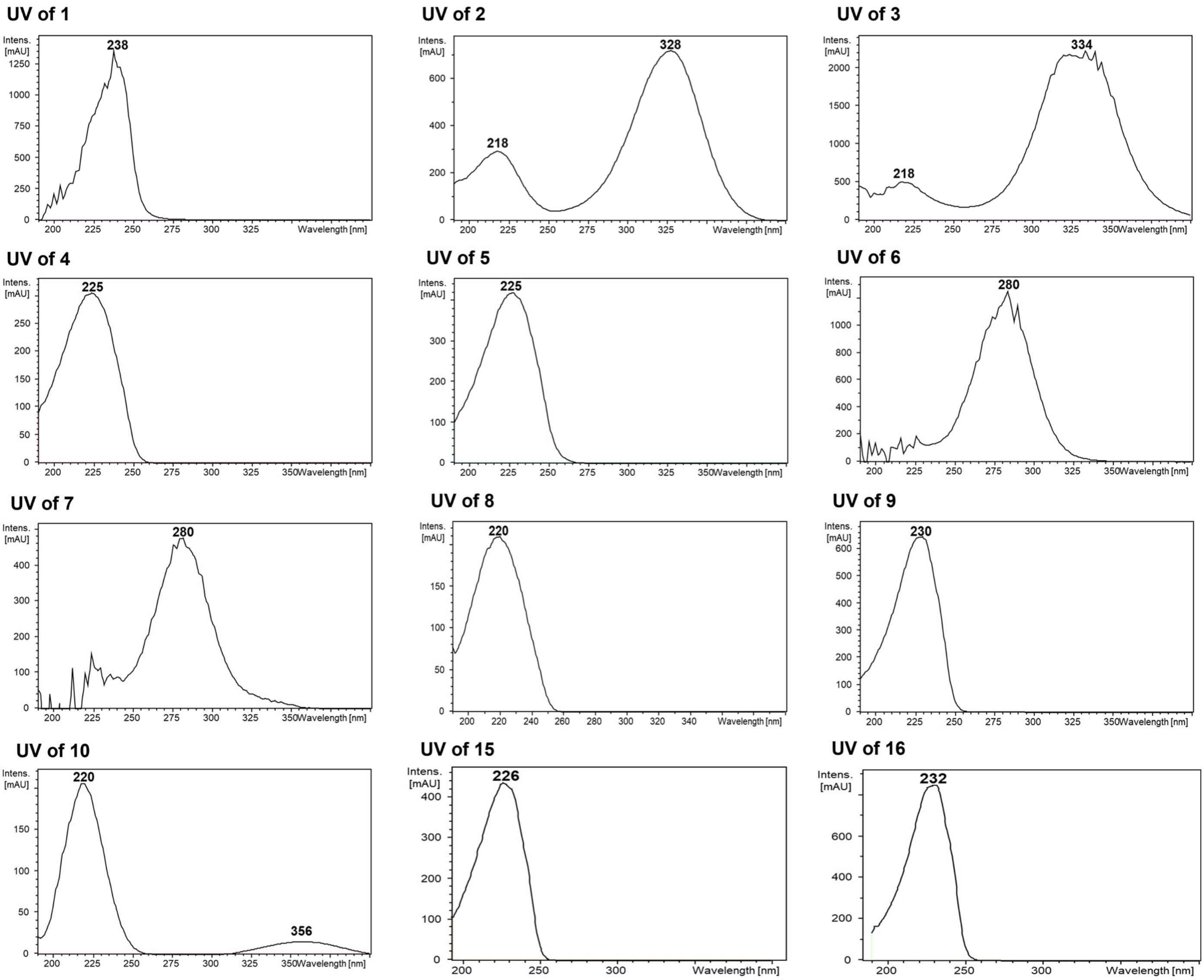

Figure S19. UV spectra of compounds **1**–**10**, **15**, and **16**.

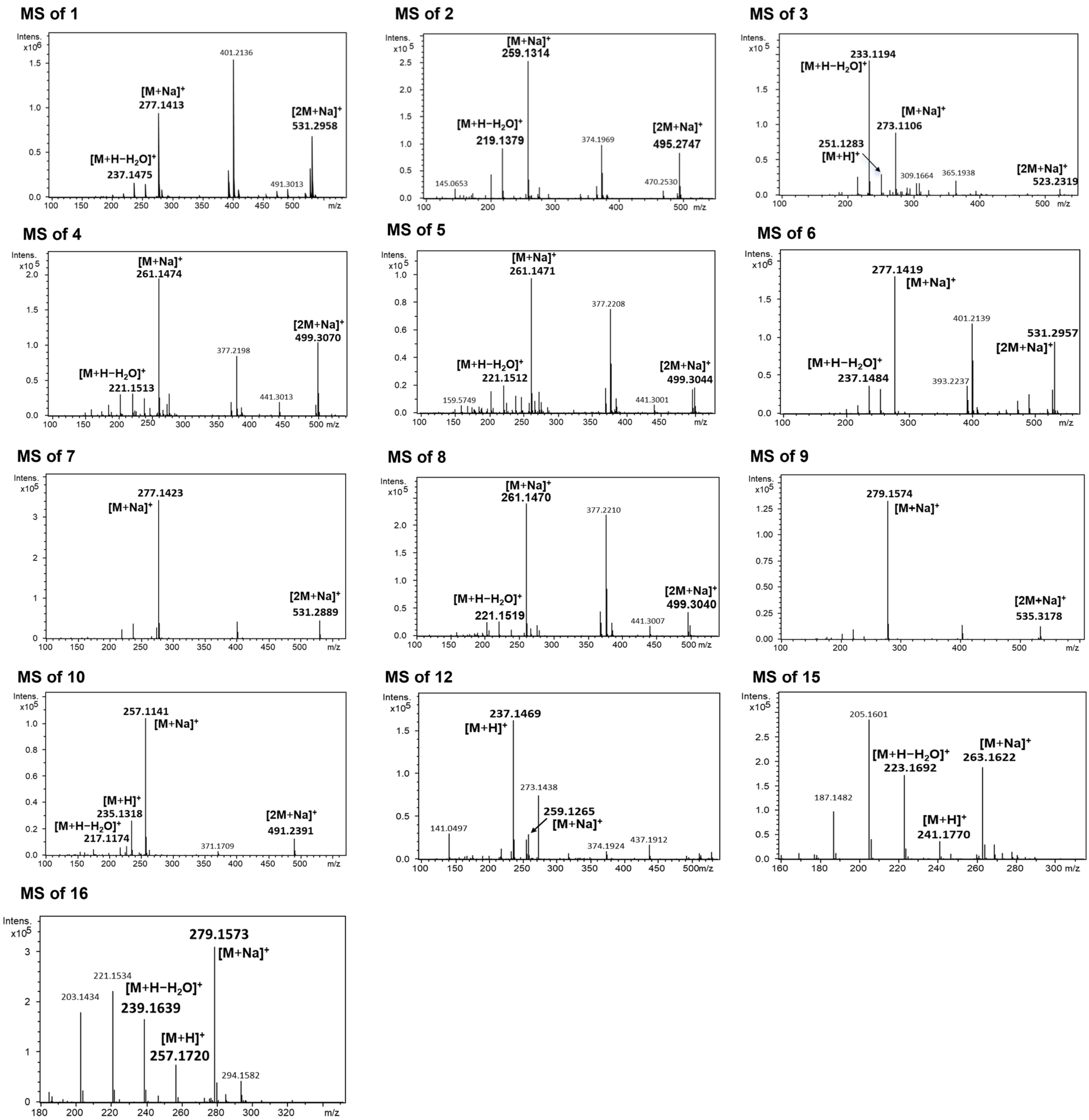

Figure S20. Mass spectra of compounds **1**–**10**, **12**, **15**, and **16**.

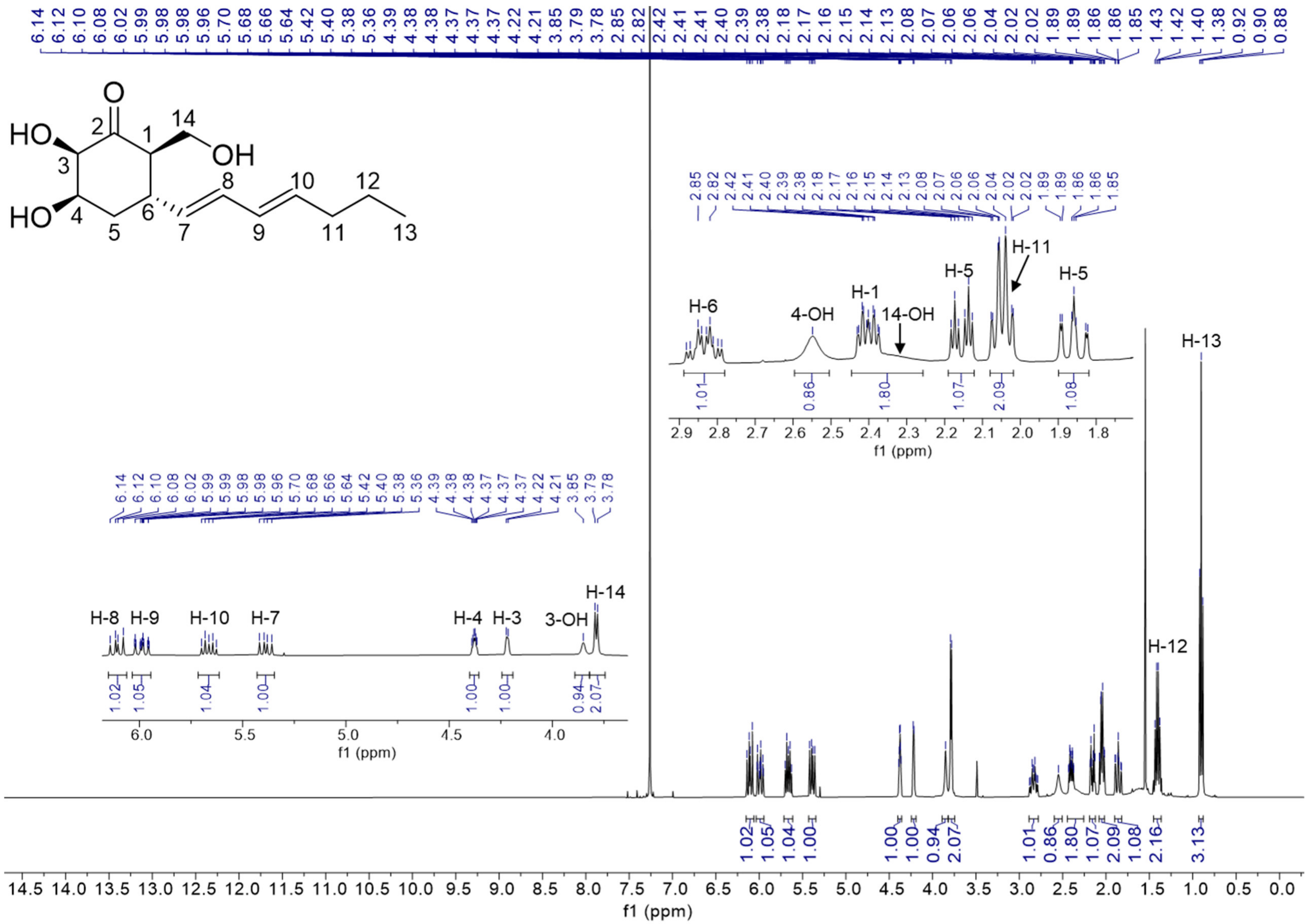

Figure S21. ^{1}H NMR spectrum of (+)-palitantin (**1**) in $CDCl_3$ (400 MHz).

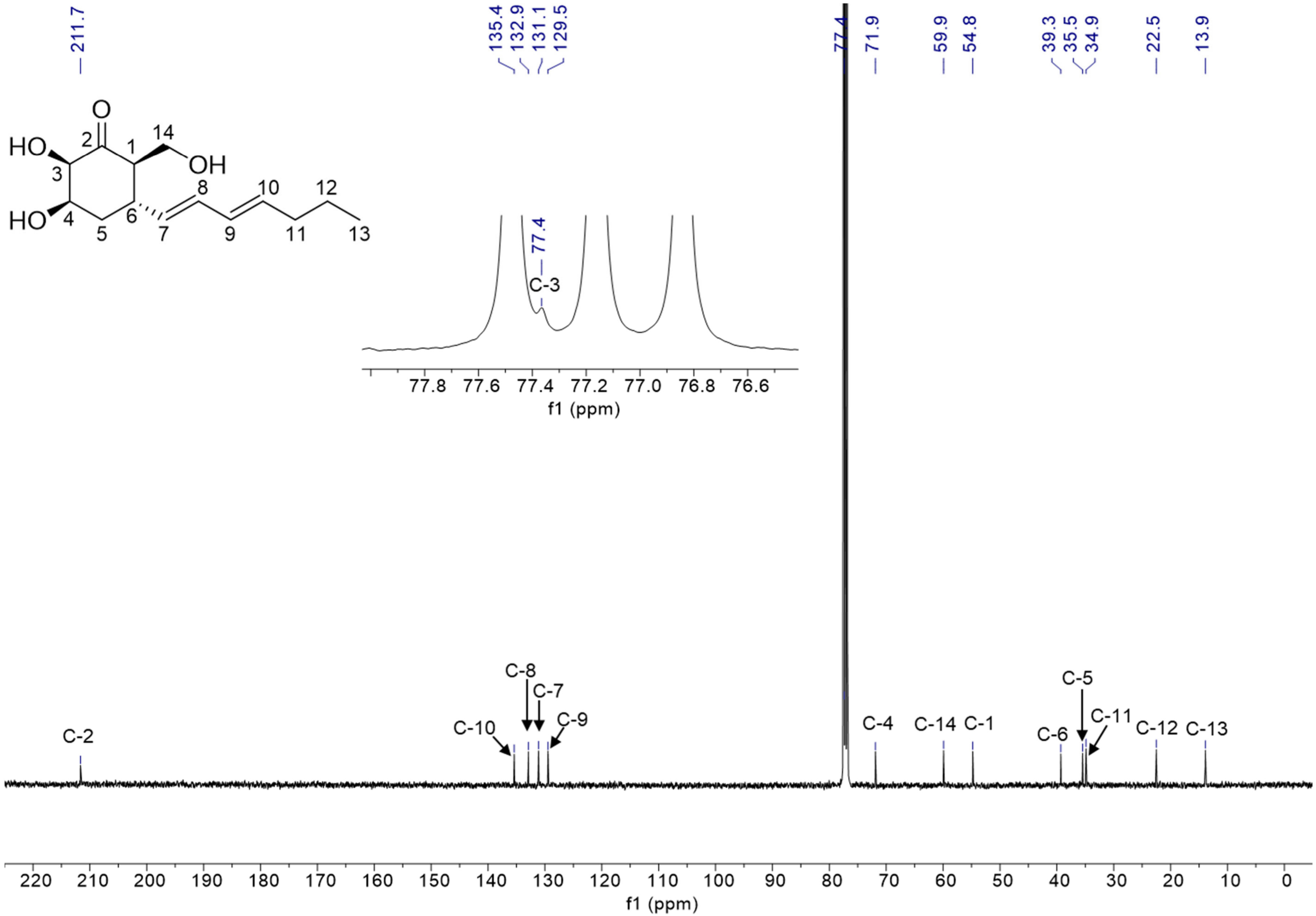

Figure S22. ^{13}C NMR spectrum of (+)-palitantin (**1**) in $CDCl_3$ (100 MHz).

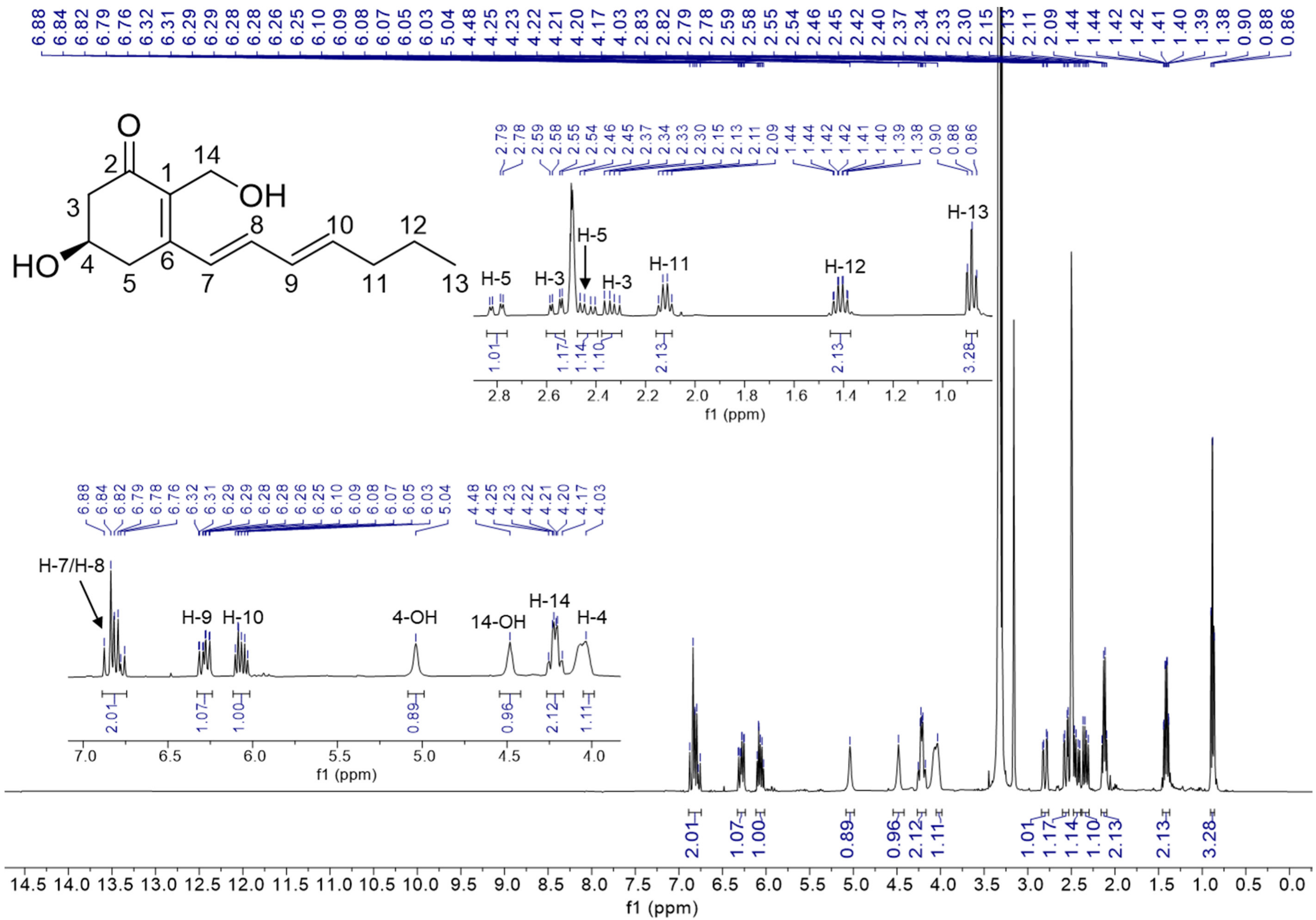

Figure S23. ^{1}H NMR spectrum of dehydropenihydrone A (**2**) in DMSO-d_6 (400 MHz).

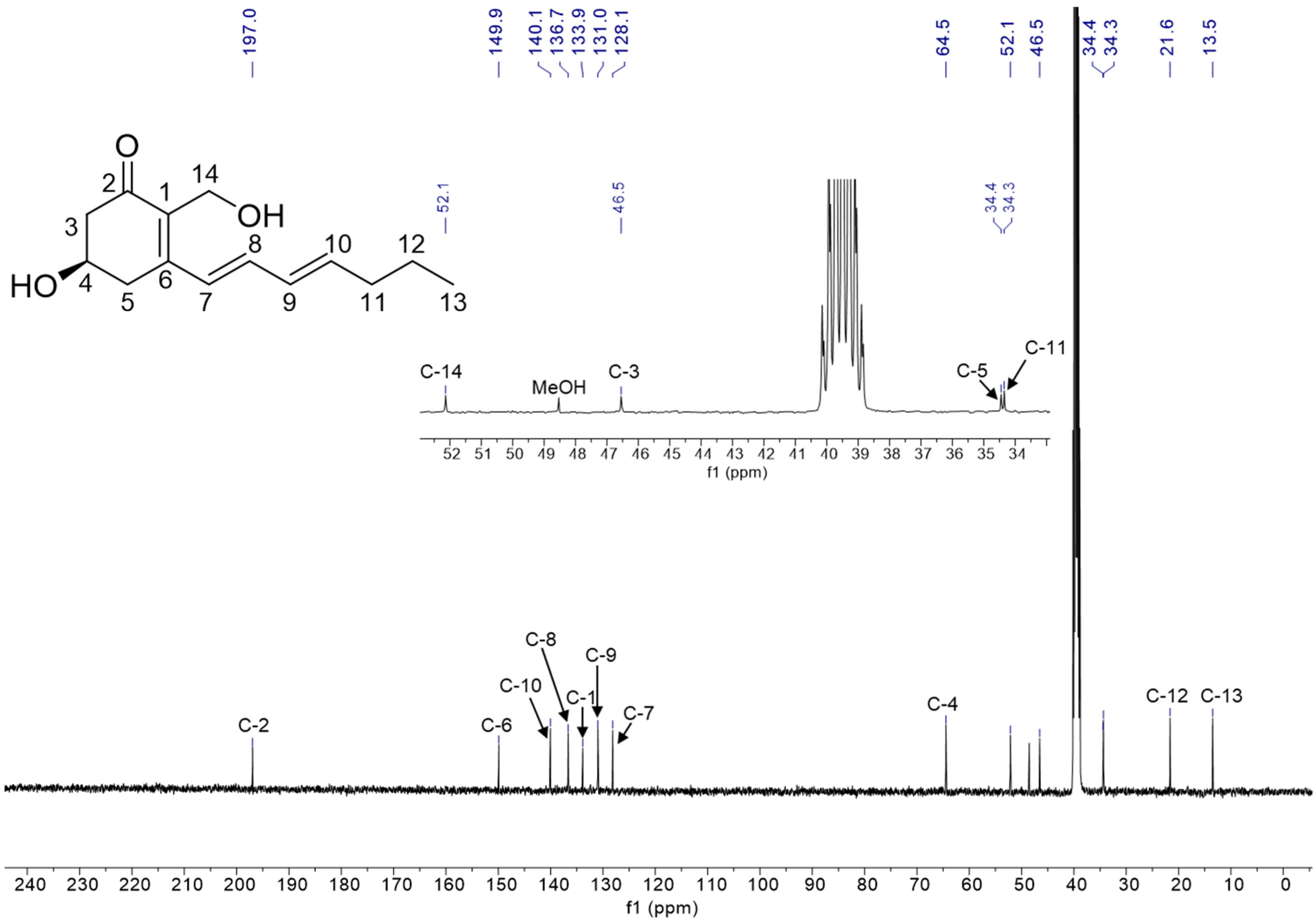

Figure S24. ^{13}C NMR spectrum of dehydropenihydrone A (**2**) in DMSO-d_6 (100 MHz).

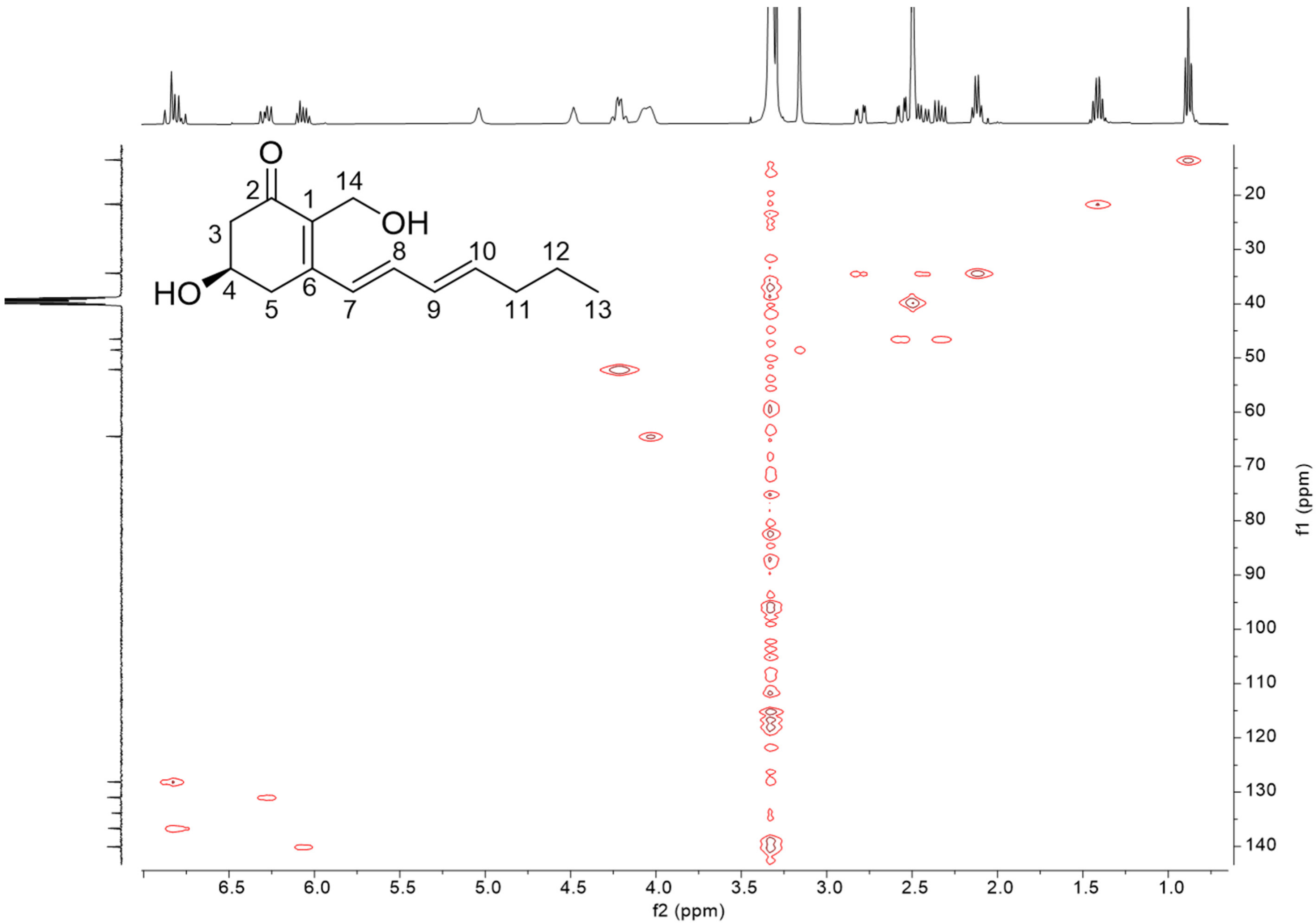

Figure S25. HSQC spectrum of dehydropenihydrone A (**2**) in DMSO-d_6.

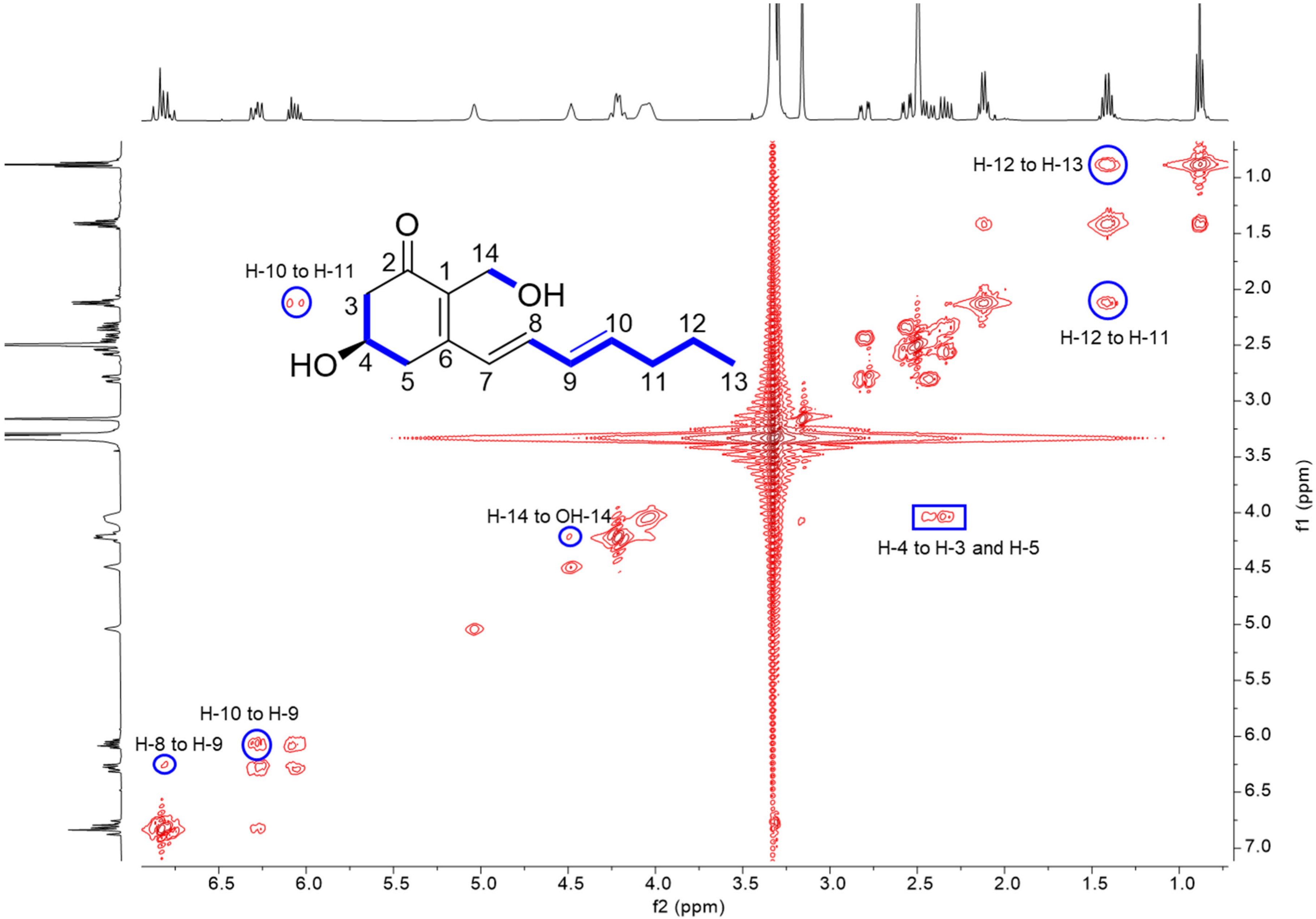

Figure S26. ^{1}H-^{1}H COSY spectrum of dehydropenihydrone A (**2**) in DMSO-d_6.

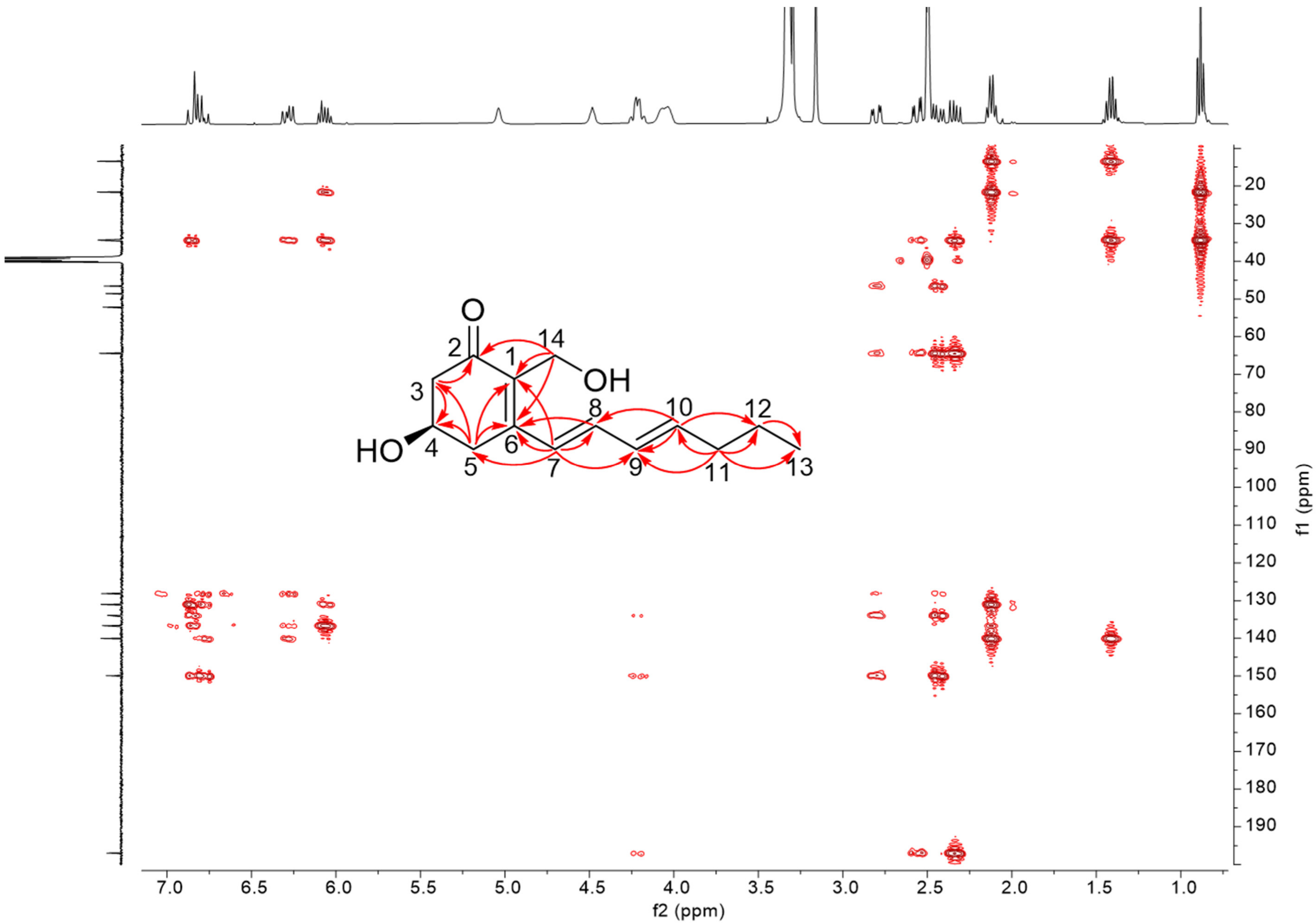

Figure S27. HMBC spectrum of dehydropenihydrone A (**2**) in DMSO-d_6.

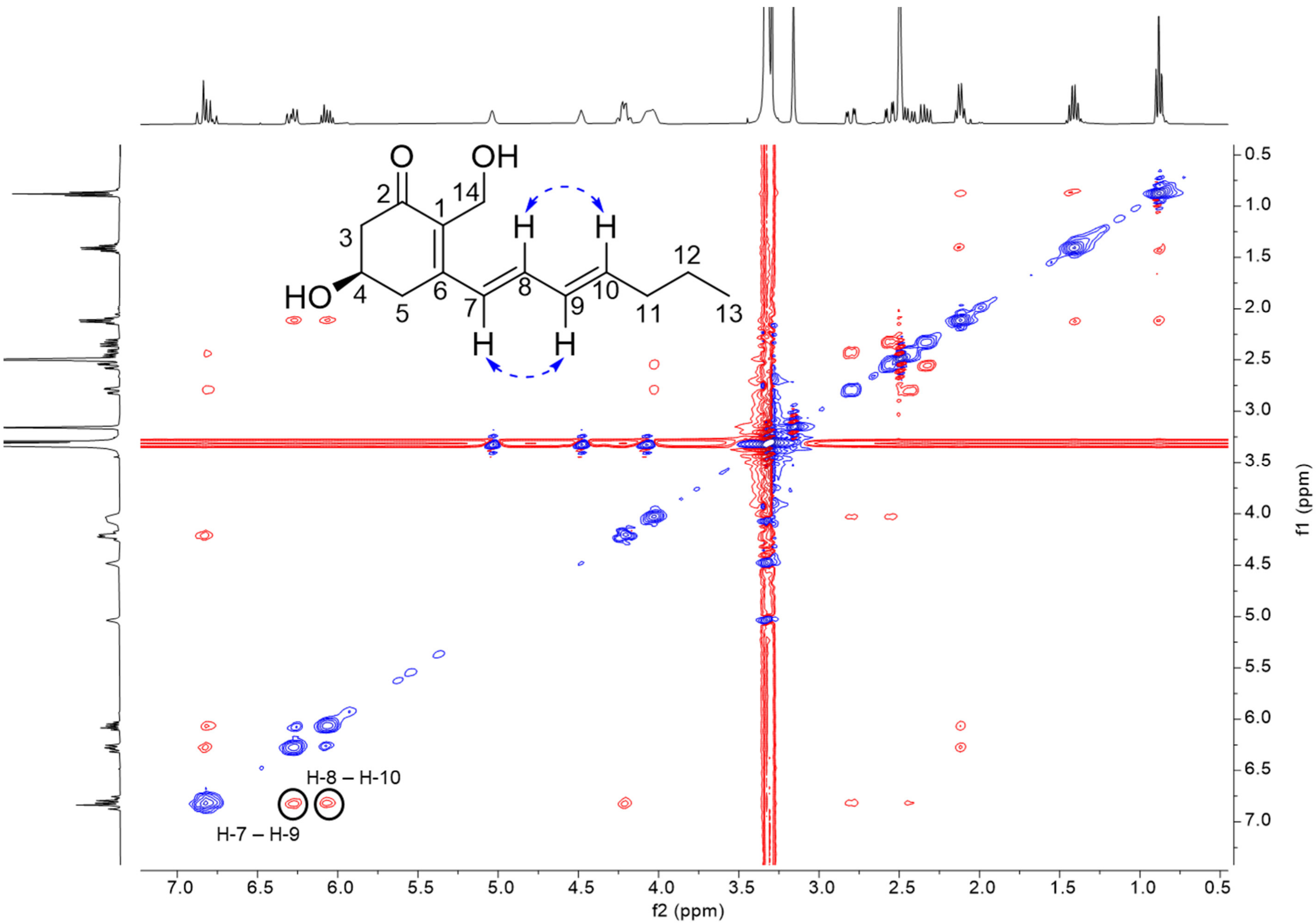

Figure S28. NOESY spectrum of dehydropenihydrone A (**2**) in DMSO-d_6.

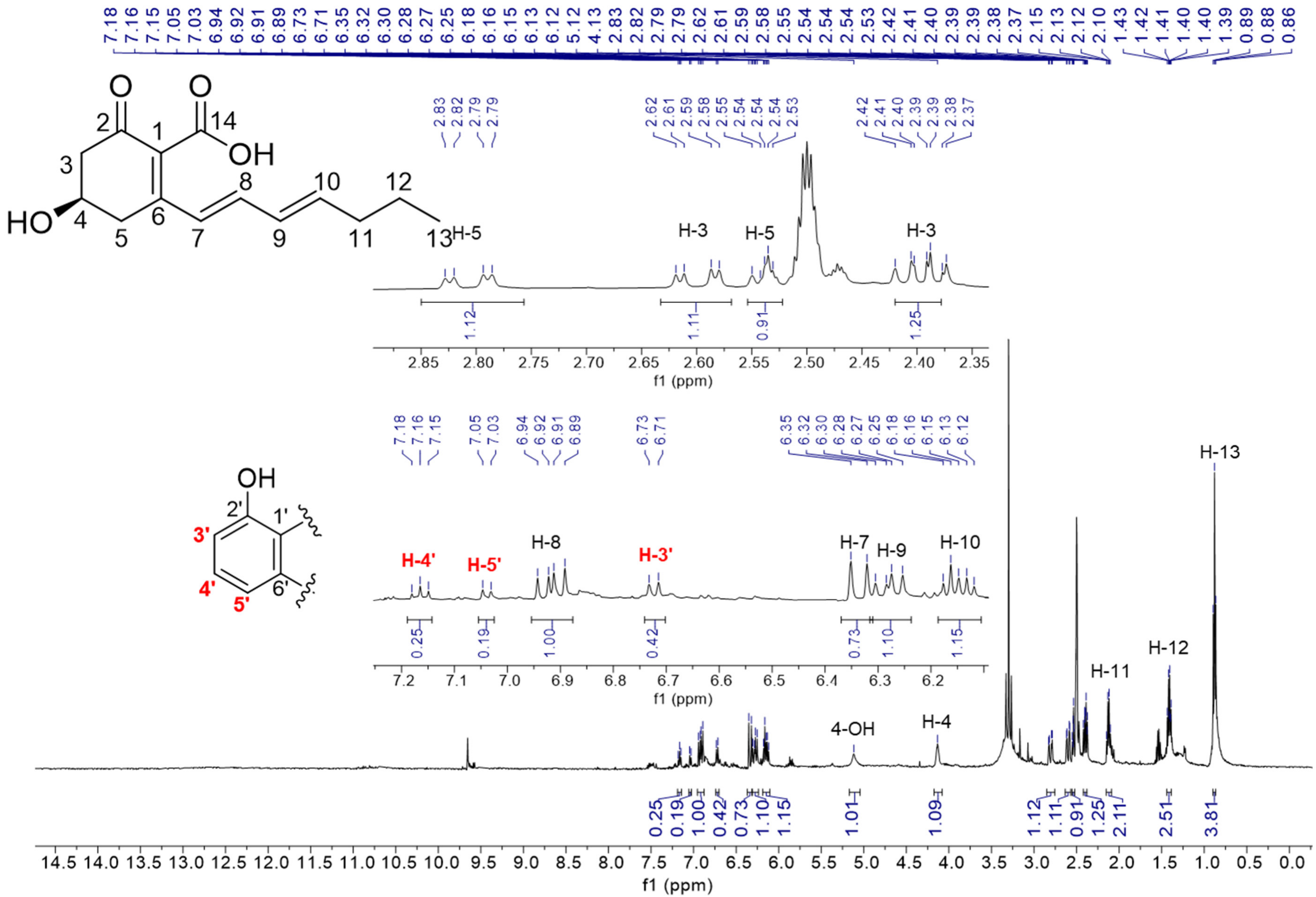

Figure S29. ^{1}H NMR spectrum of dehydropenihydrone B (**3**) in DMSO-d_6 (500 MHz) immediately after isolation. It contains about 20% of the aromatic product.

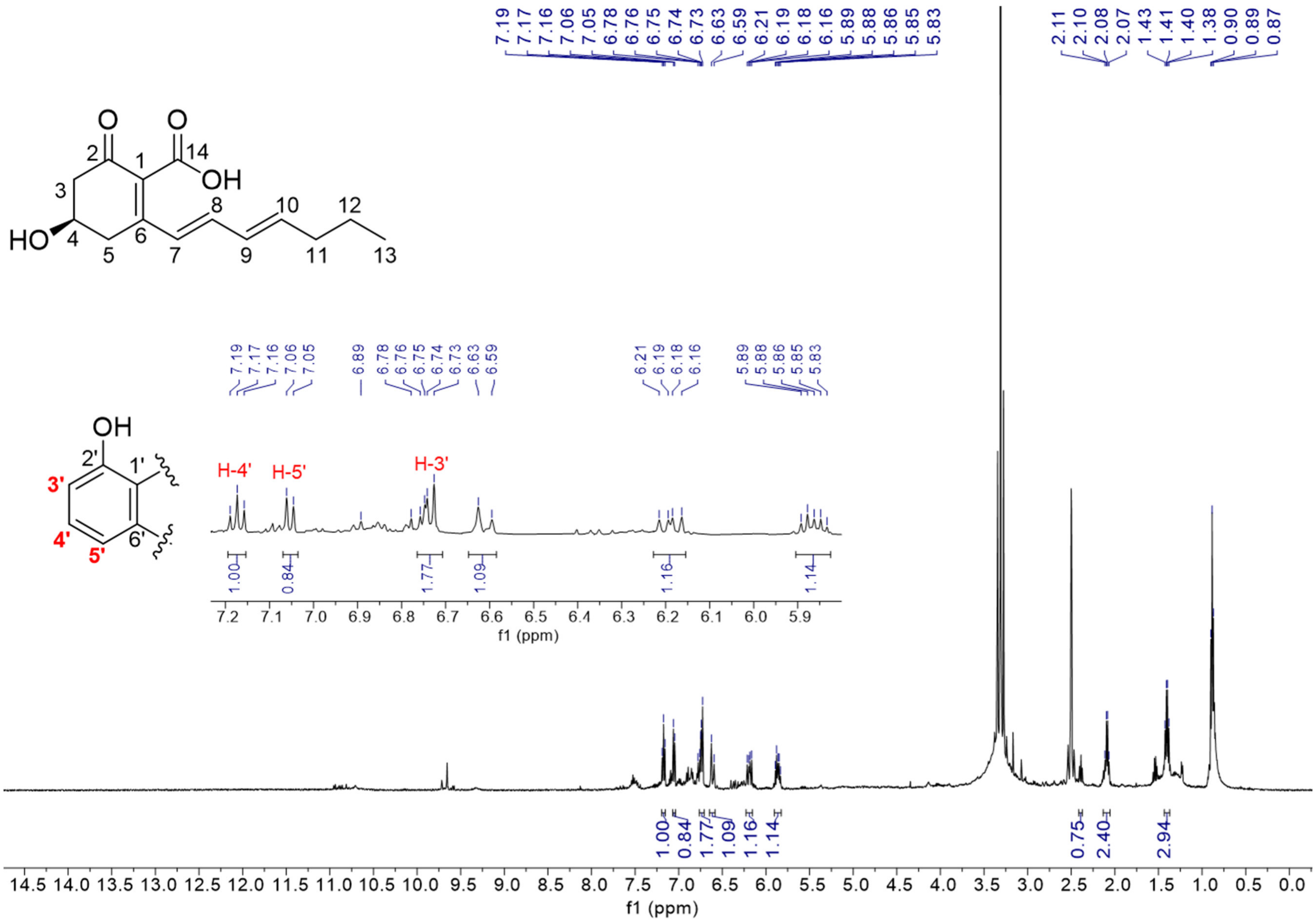

Figure S30. ^{1}H NMR spectrum of dehydropenihydrone B (**3**) after 7 days in DMSO-d_6 (500 MHz).
It shows that 80% of **3** was converted to its aromatic derivative.

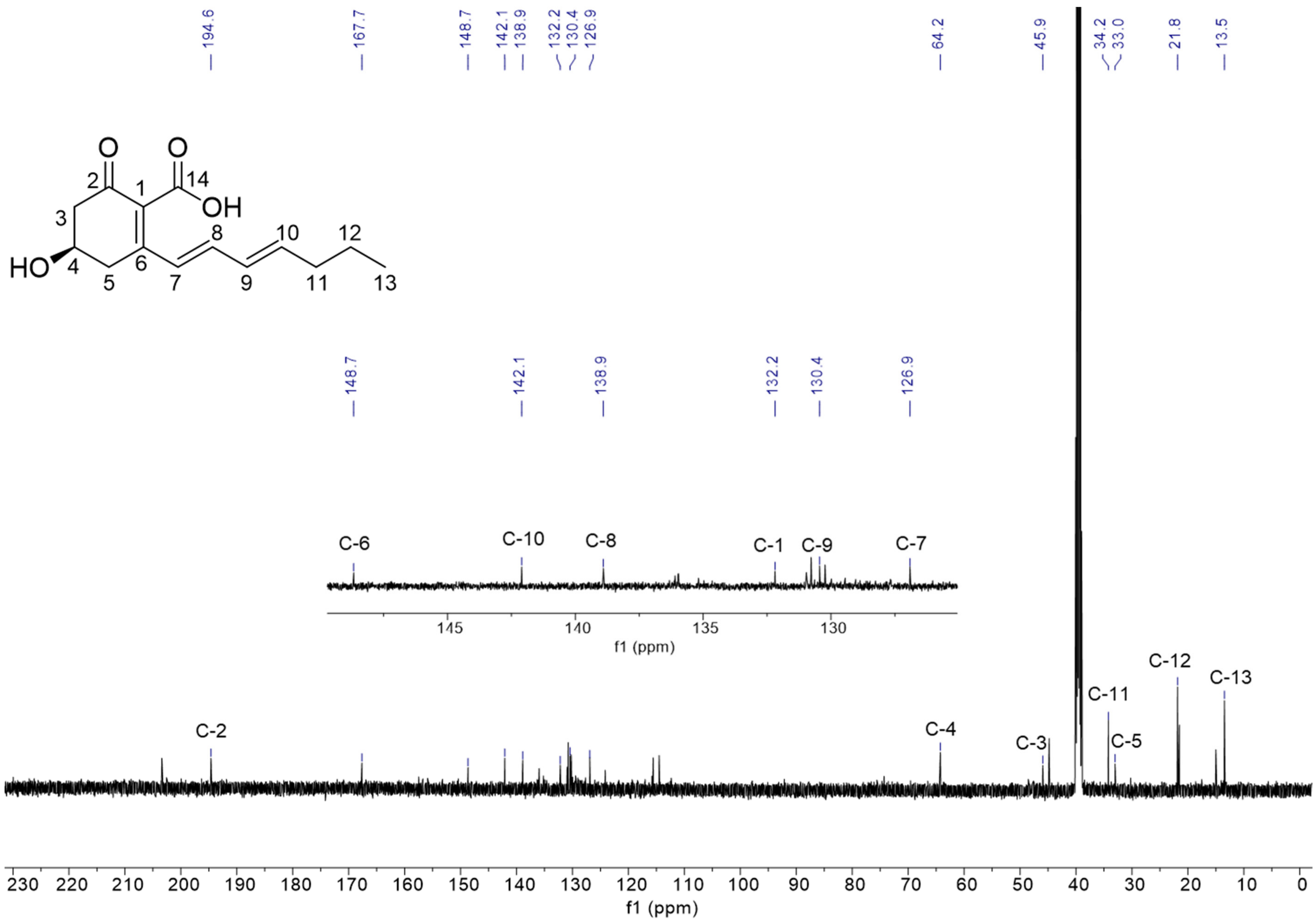

Figure S31. ^{13}C NMR spectrum of dehydropenihydrone B (**3**) in DMSO-d_6 (125 MHz).

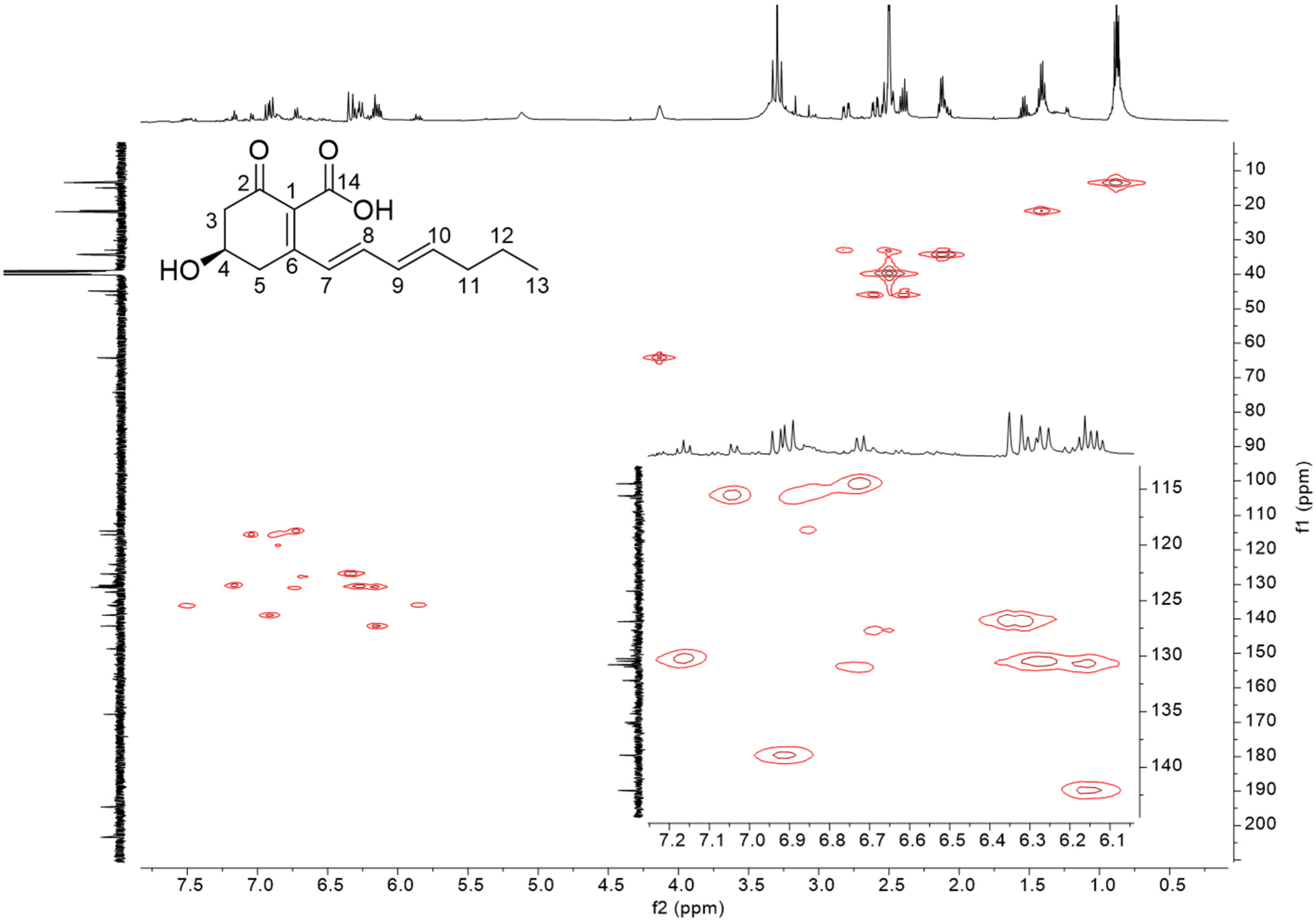

Figure S32. HSQC spectrum of dehydropenihydrone B (**3**) in DMSO-*d*6.

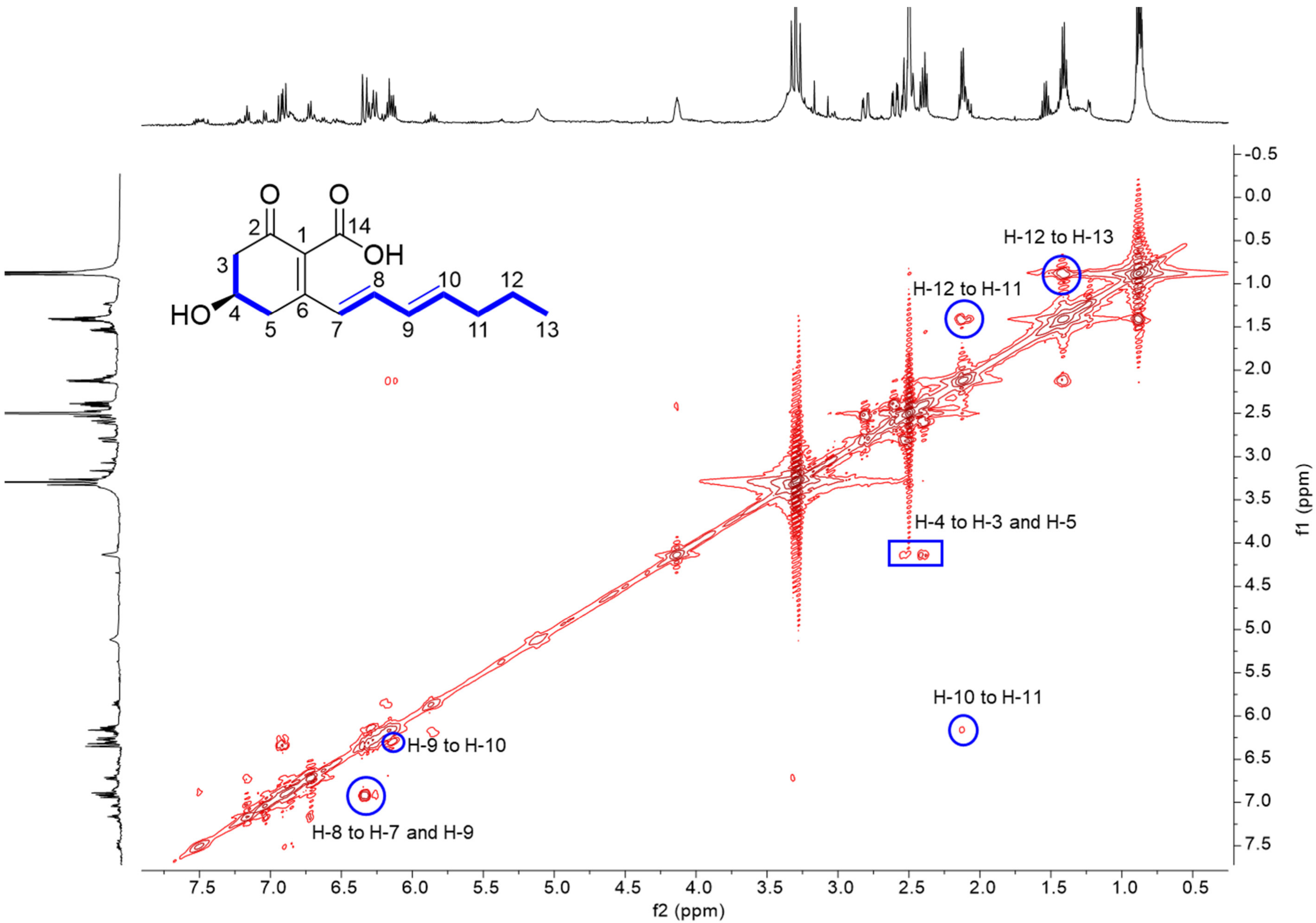

Figure S33. ^{1}H-^{1}H COSY spectrum of dehydropenihydrone B (**3**) in DMSO-d_6.

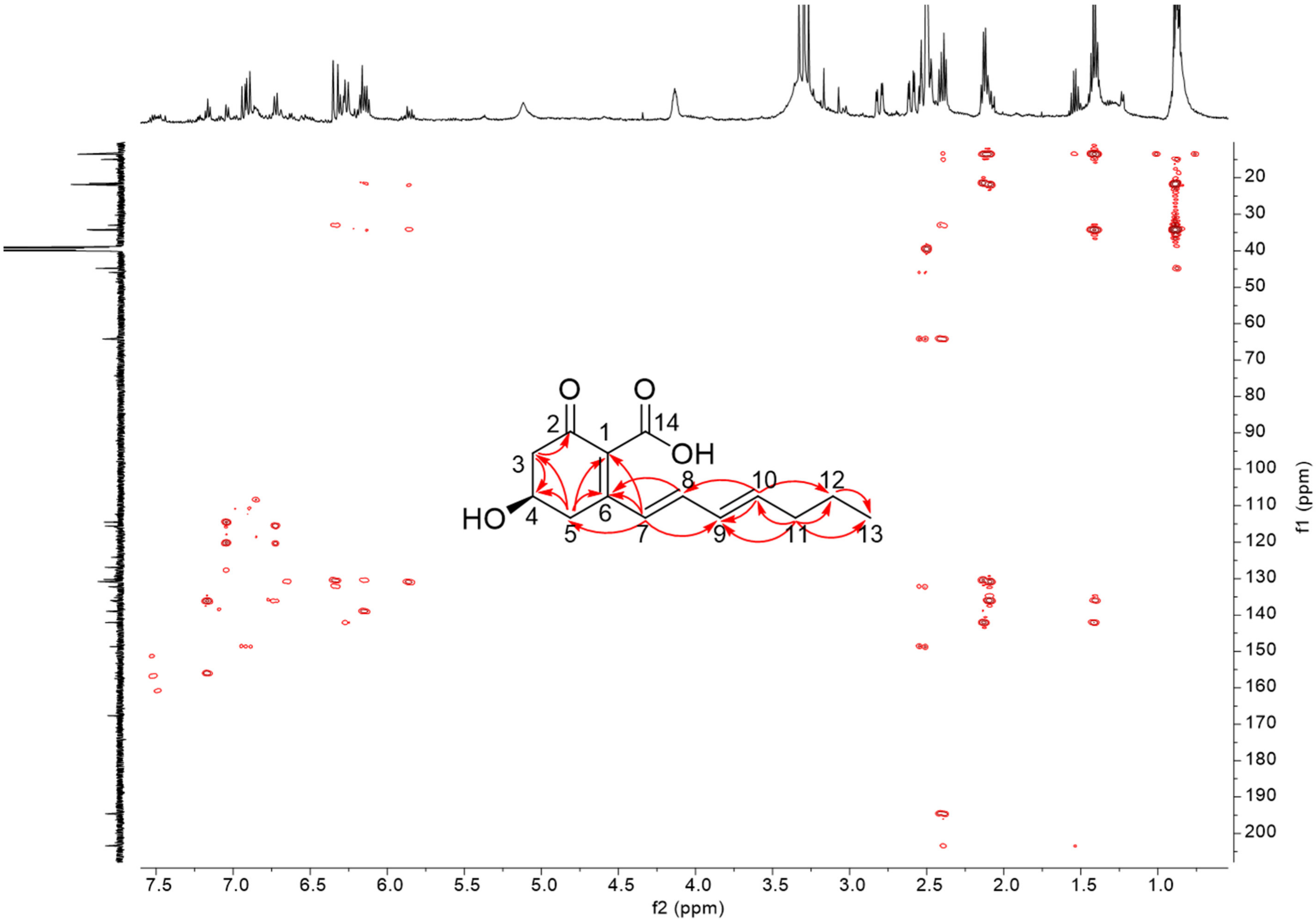

Figure S34. HMBC spectrum of dehydropenihydrone B (**3**) in DMSO-d_6.

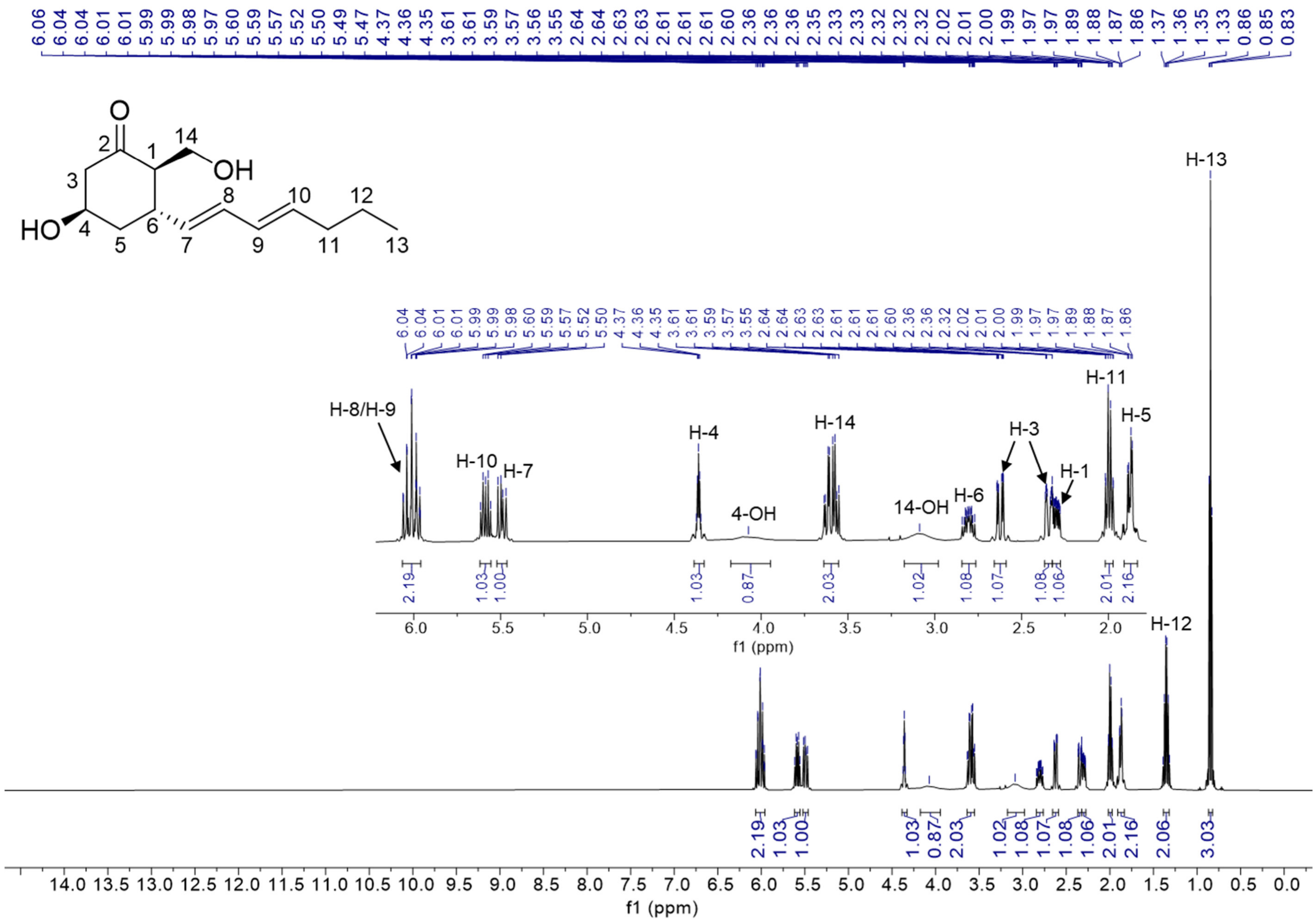

Figure S35. ^{1}H NMR spectrum of penihydrone (**4**) in acetone-d_6 (500 MHz).

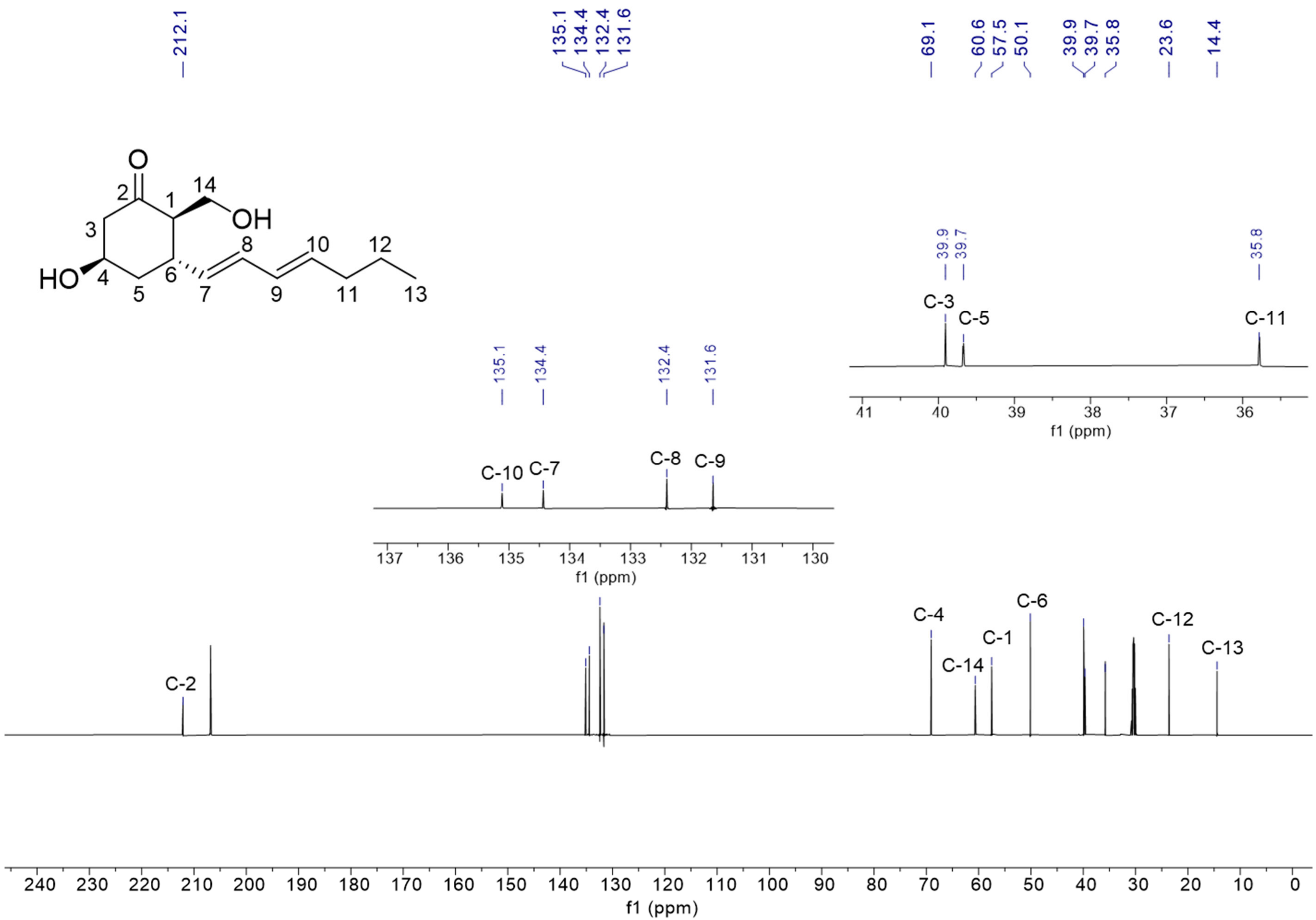

Figure S36. ^{13}C NMR spectrum of penihydrone (**4**) in acetone-d_6 (125 MHz).

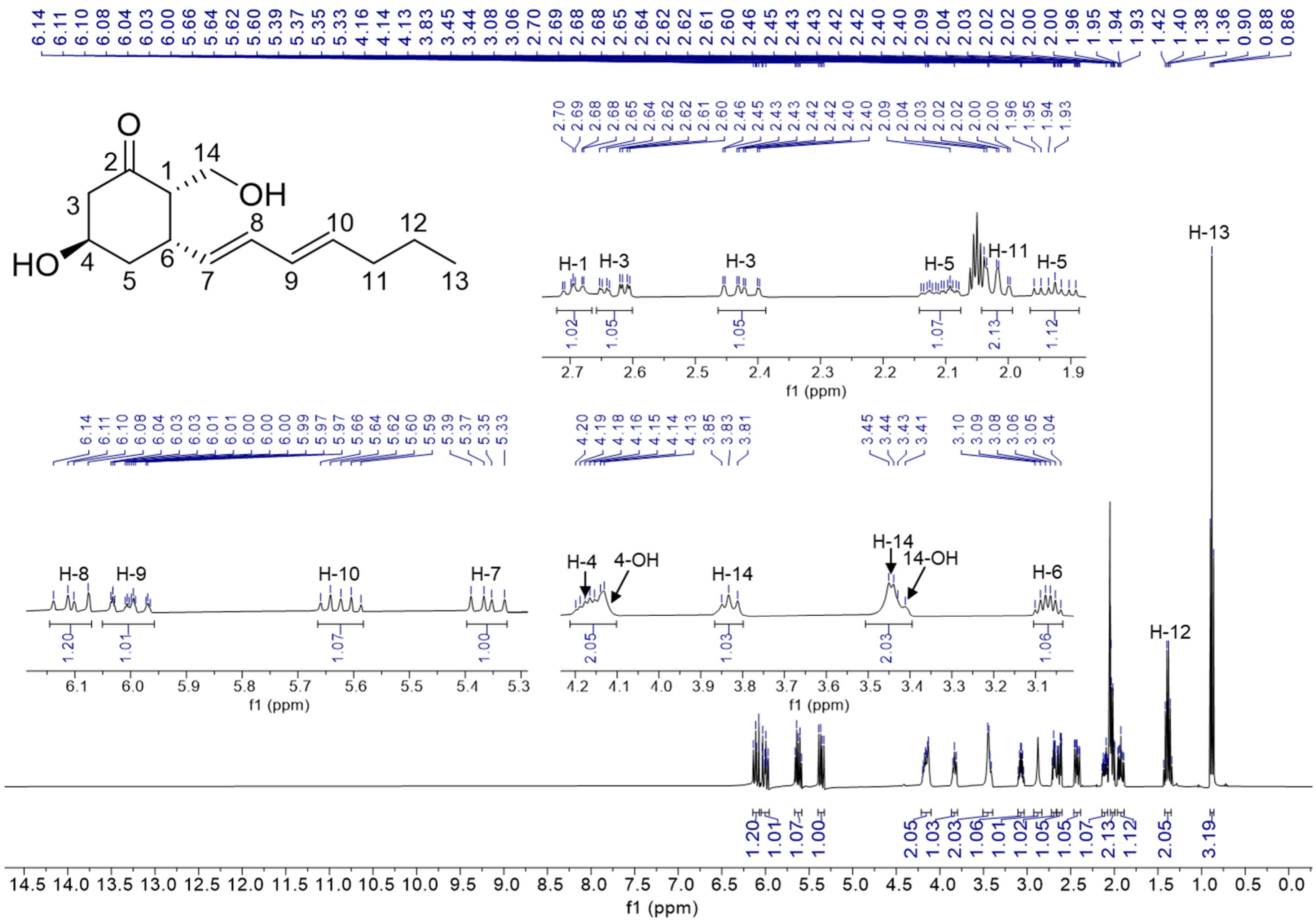

Figure S37. ^{1}H NMR spectrum of penihydrone B (**5**) in acetone-*d*₆ (400 MHz).

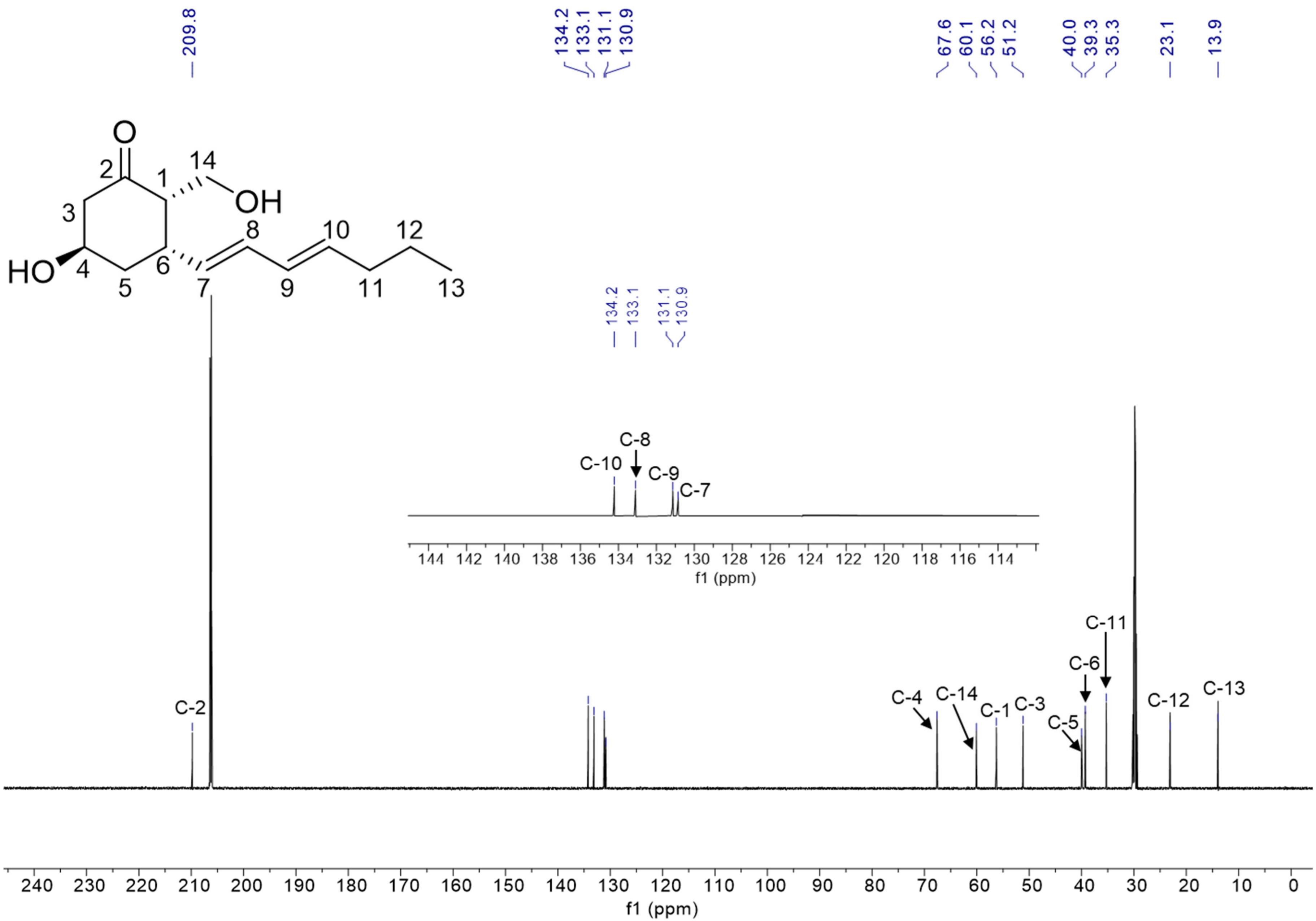

Figure S38. ^{13}C NMR spectrum of penihydrone B (**5**) in acetone-d_6 (125 MHz).

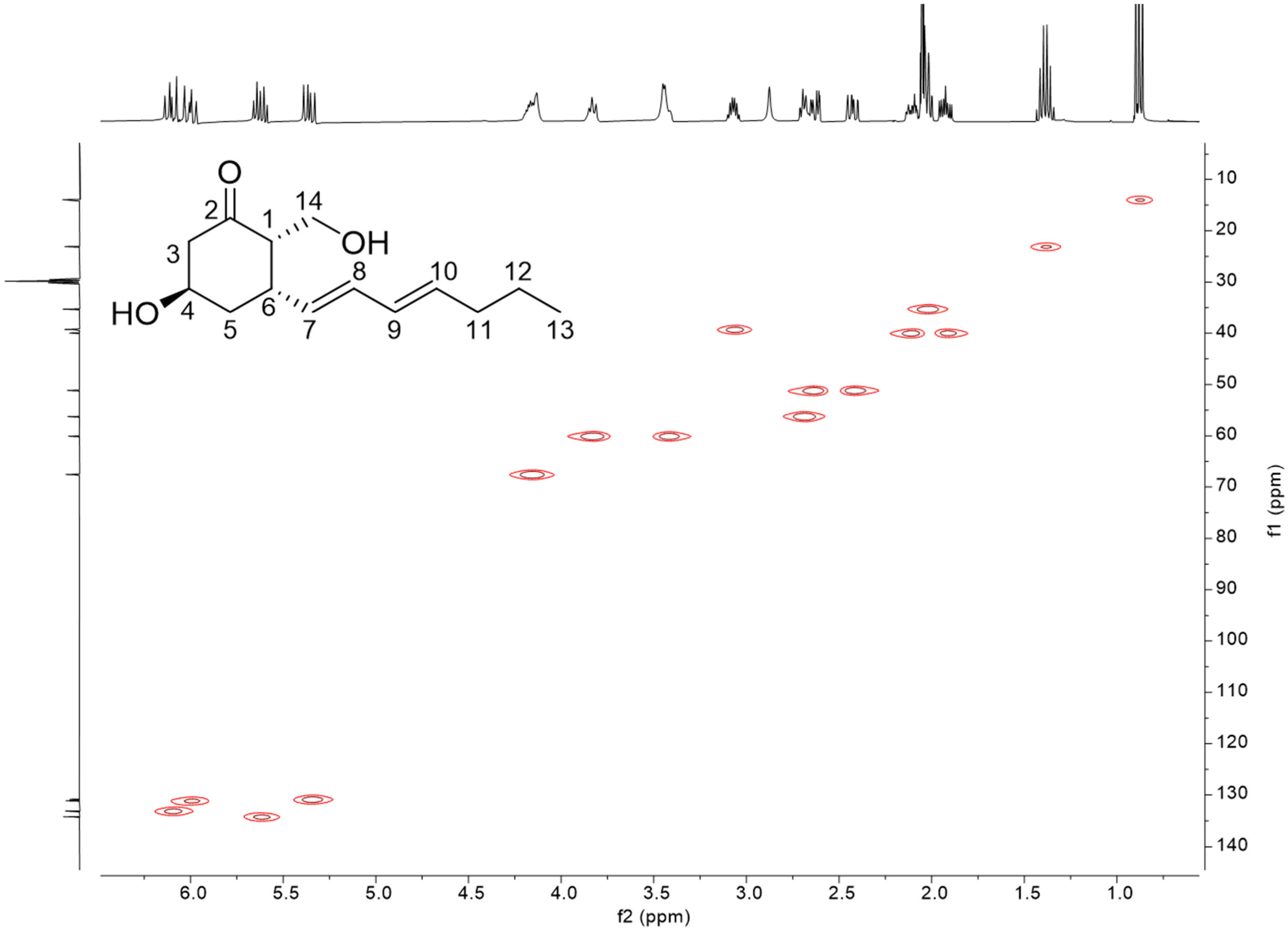

Figure S39. HSQC spectrum of penihydrone B (**5**) in acetone-d_6.

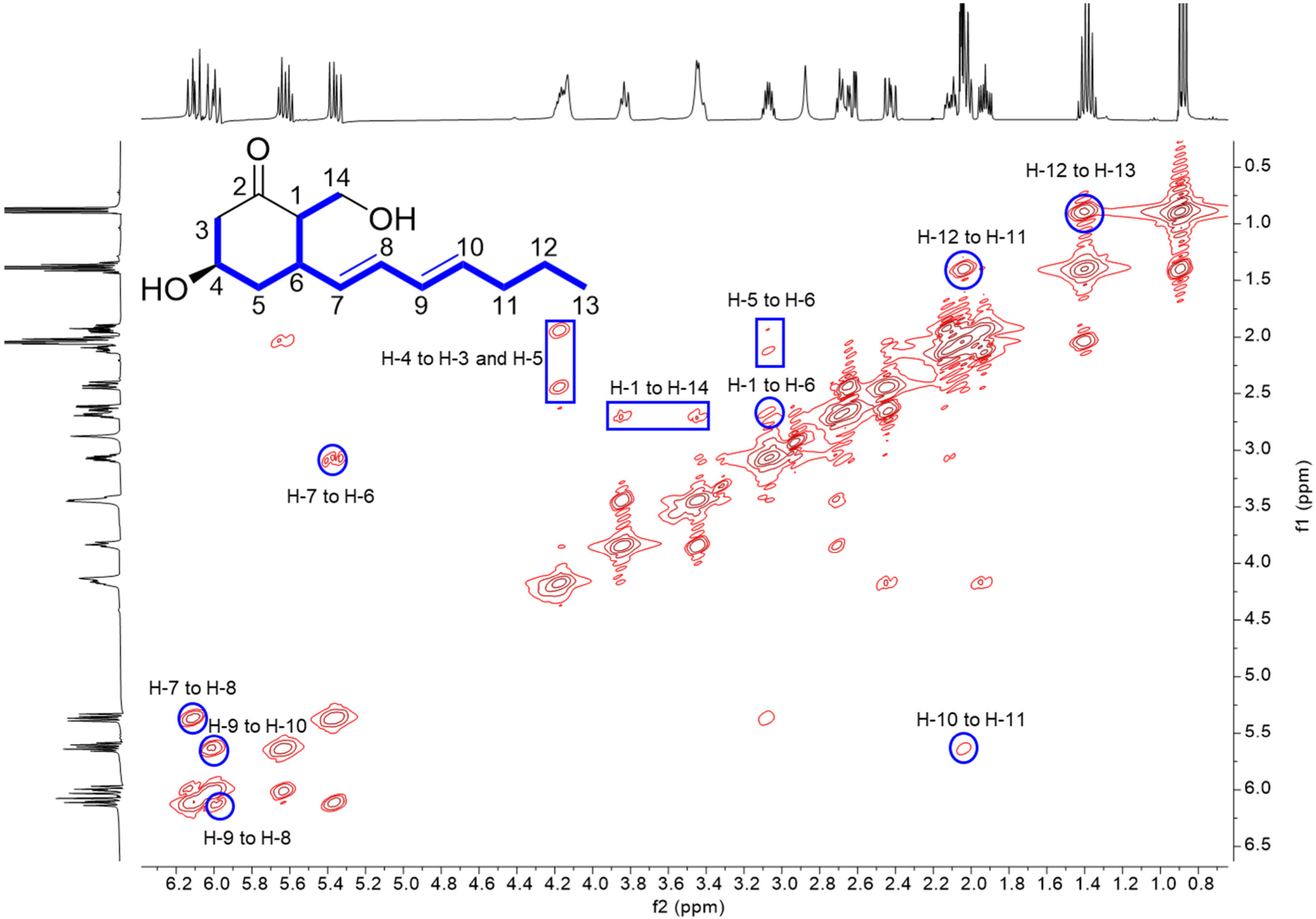

Figure S40. ¹H-¹H COSY spectrum of penihydrone B (**5**) in acetone-d_6.

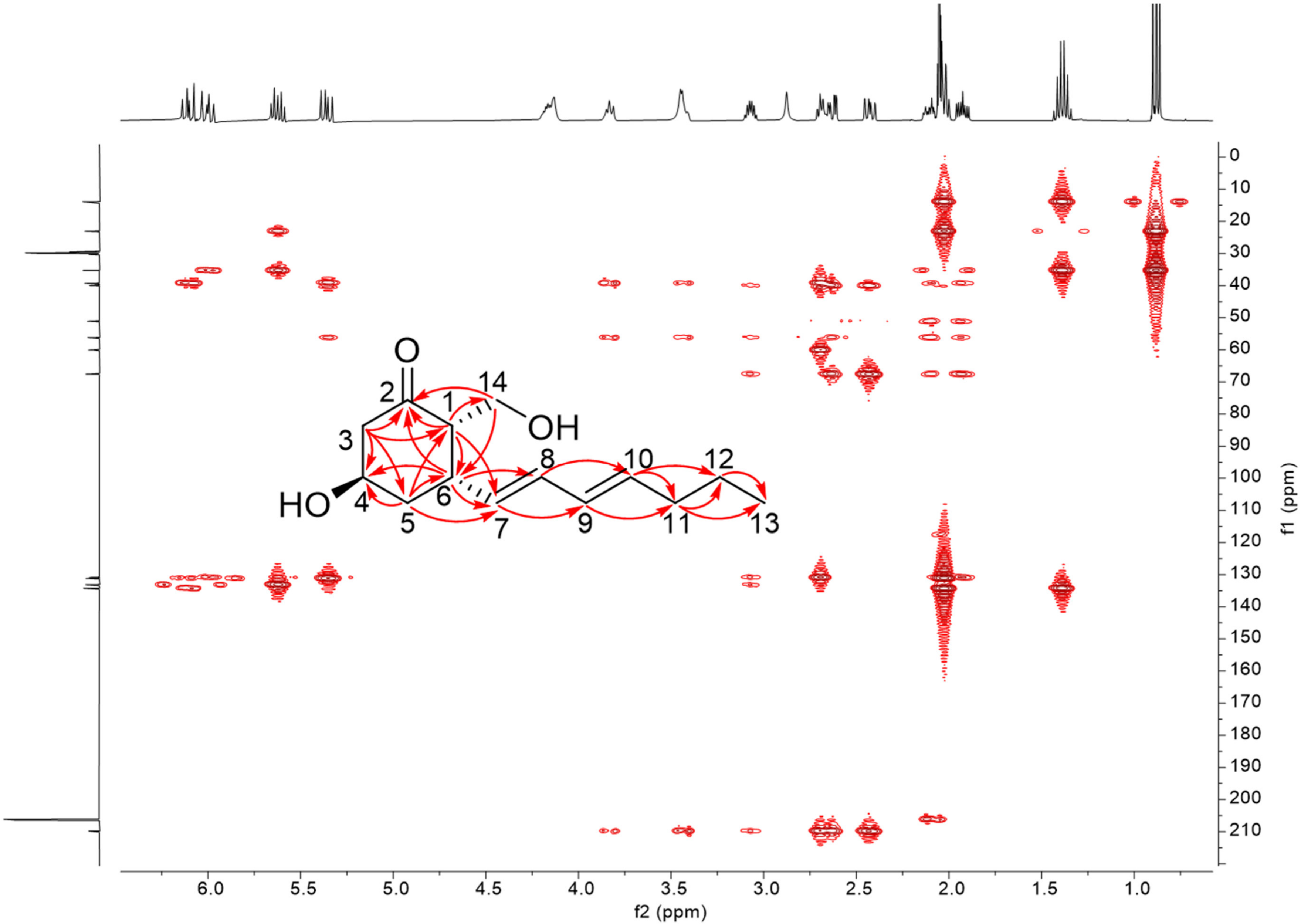

Figure S41. HMBC spectrum of penihydrone B (**5**) in acetone-d_6.

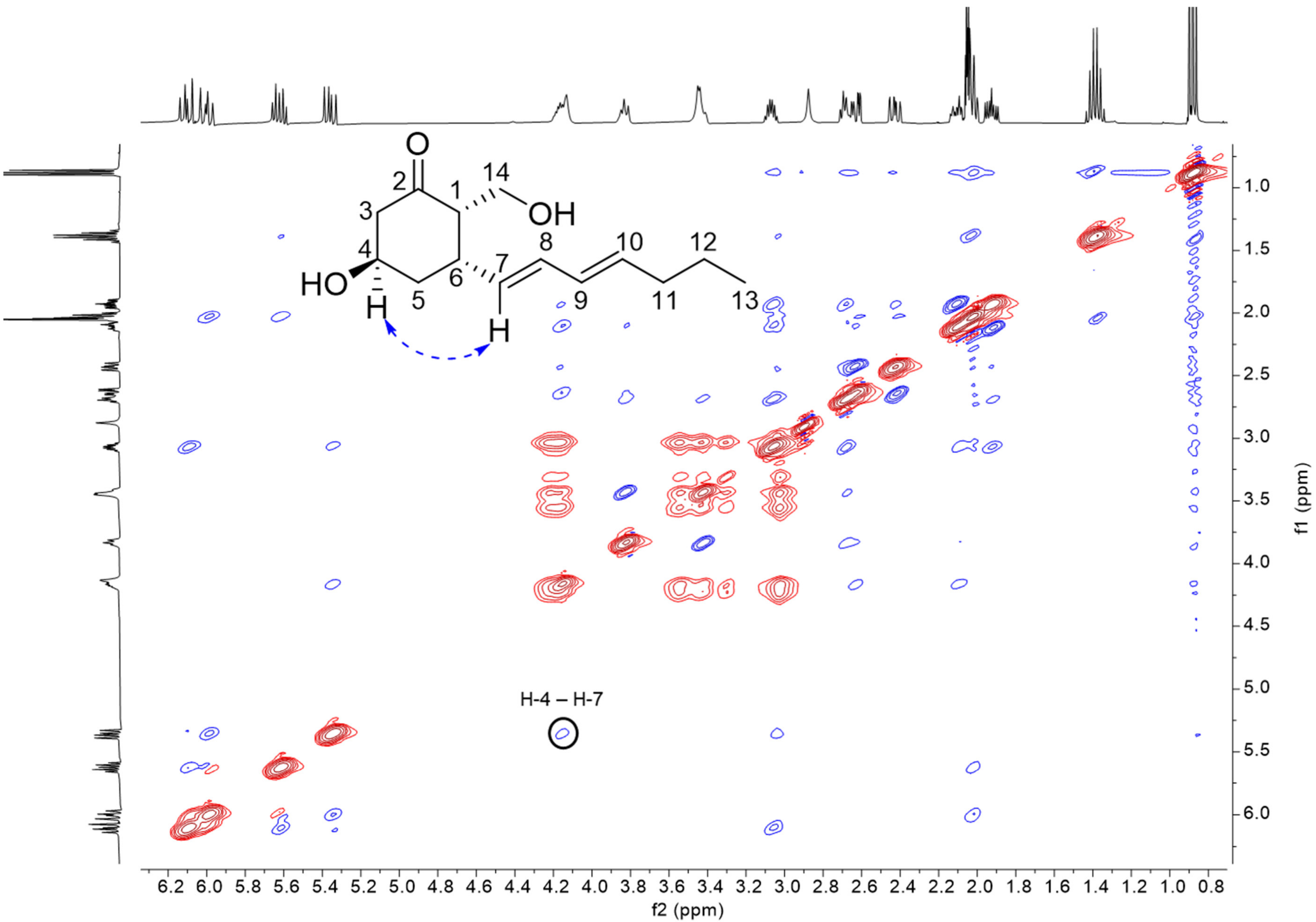

Figure S42. NOESY spectrum of penihydrone B (**5**) in acetone-*d*6.

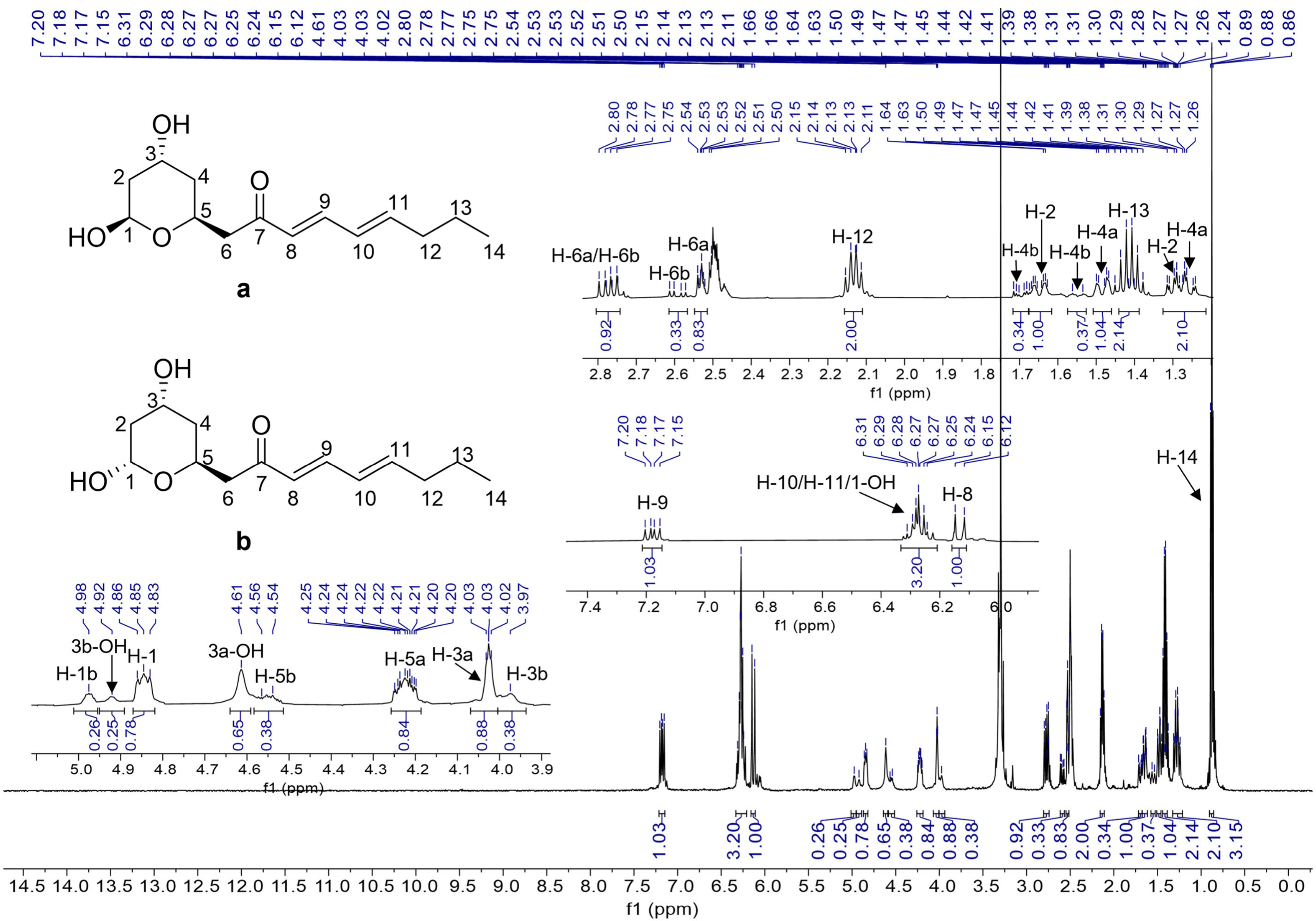

Figure S43. ^{1}H NMR spectrum of palitanol A (**6**) in DMSO-d_6 (500 MHz). Labelling without a or b means signals for both structures.

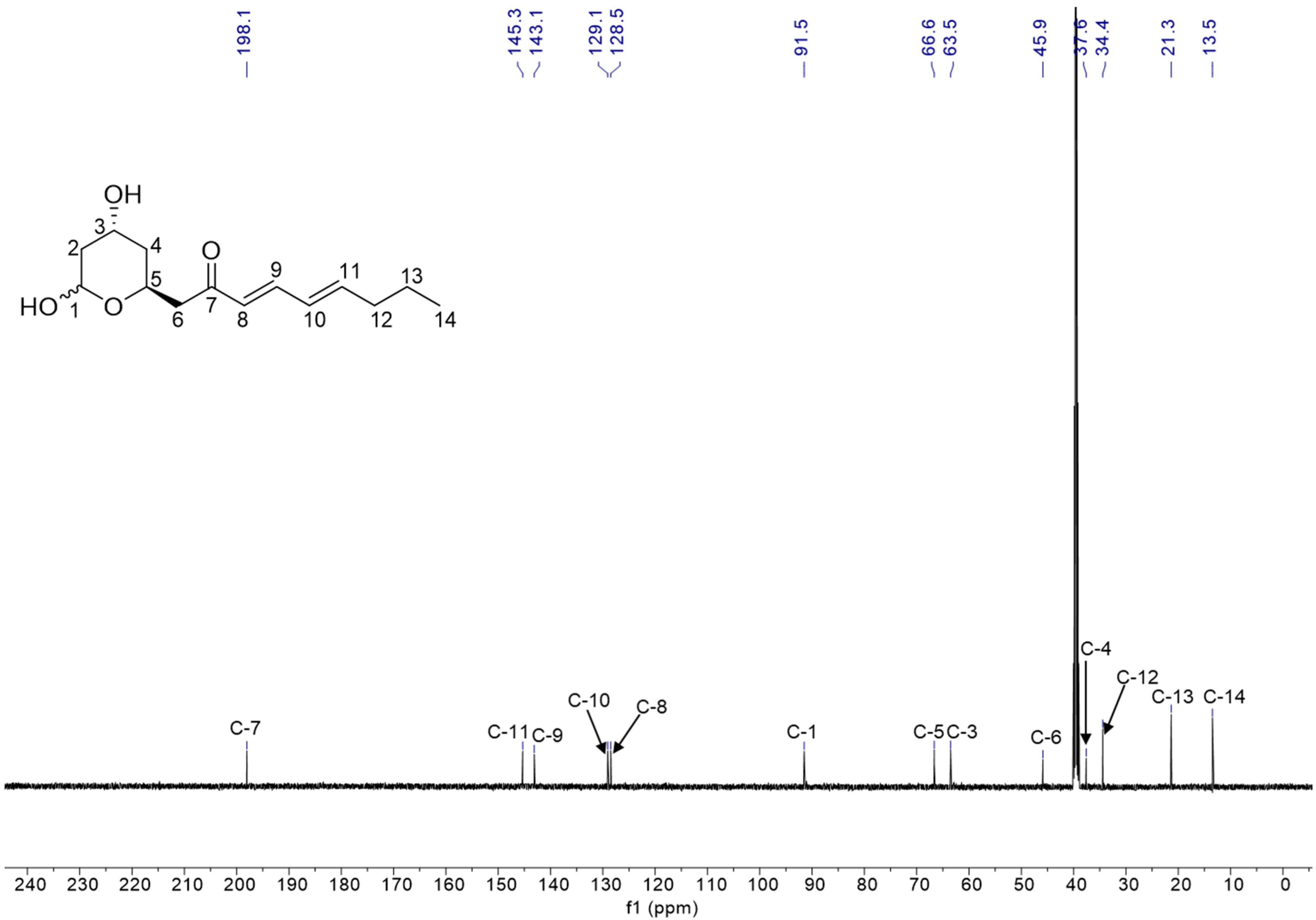

Figure S44. ^{13}C NMR spectrum of palitanol A (**6**) in DMSO-d_6 (125 MHz).

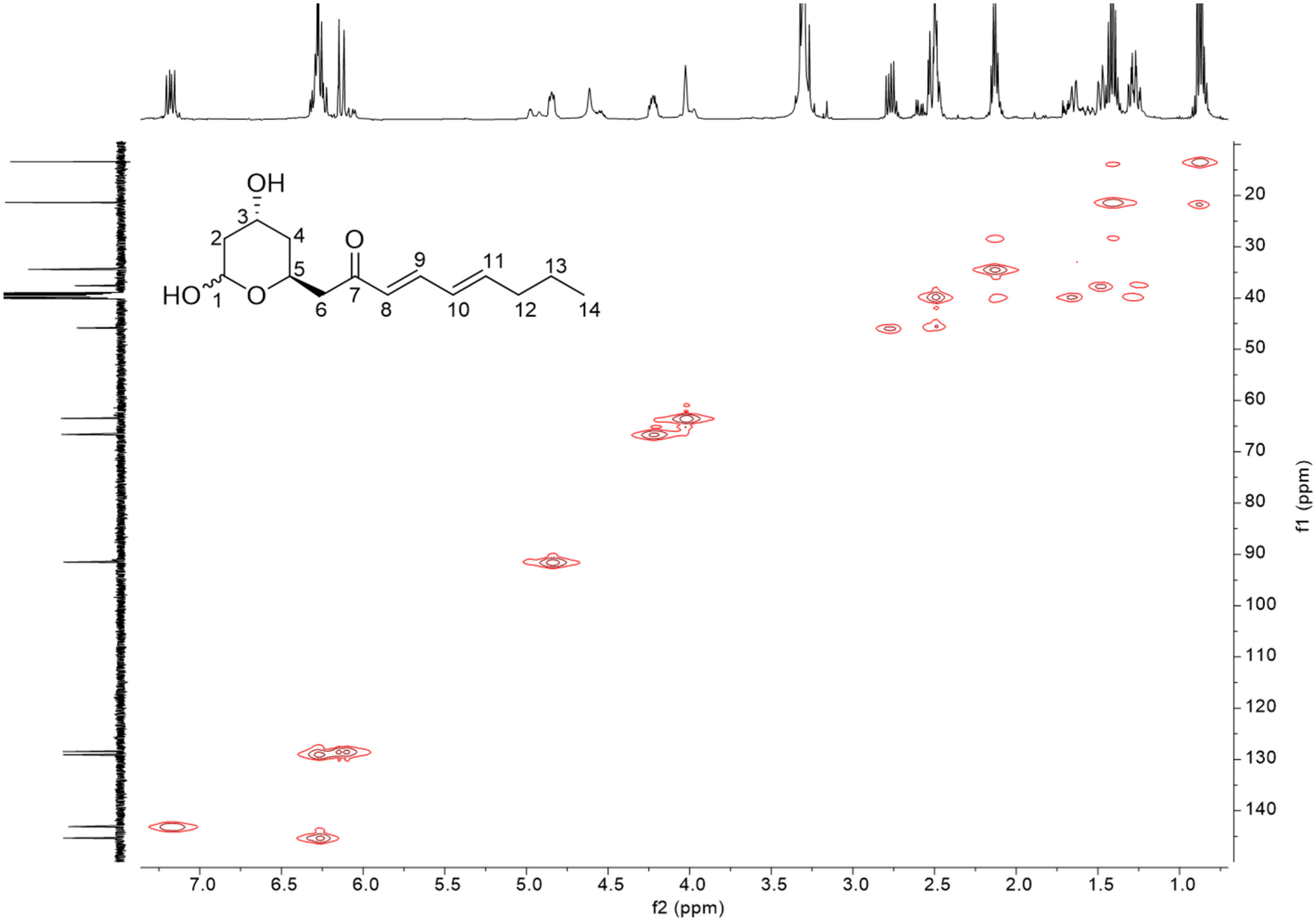

Figure S45. HSQC spectrum of palitanol A (**6**) in DMSO-*d*6.

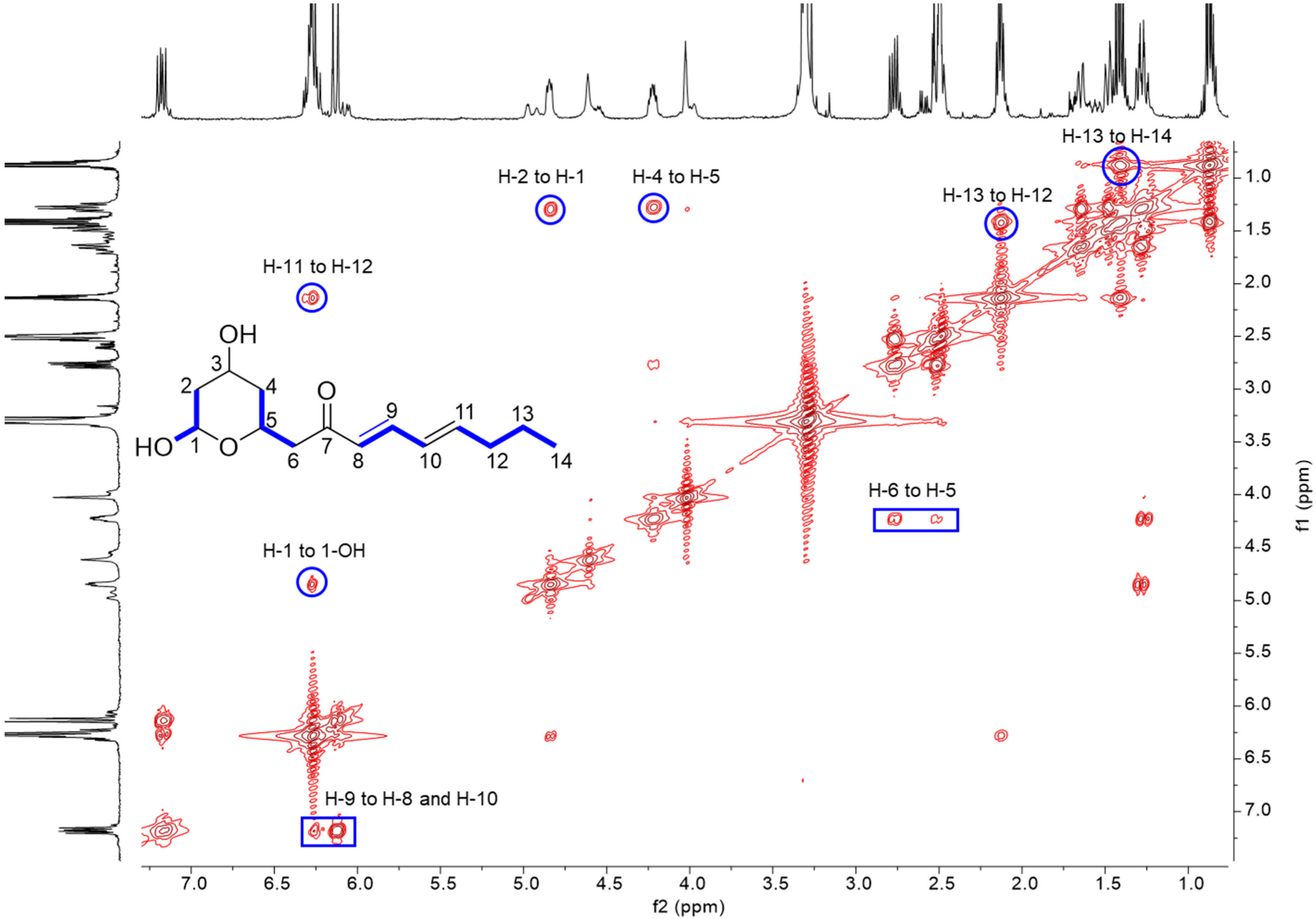

Figure S46. ${}^{1}H$-${}^{1}H$ COSY spectrum of palitanol A (**6**) in DMSO-d_6.

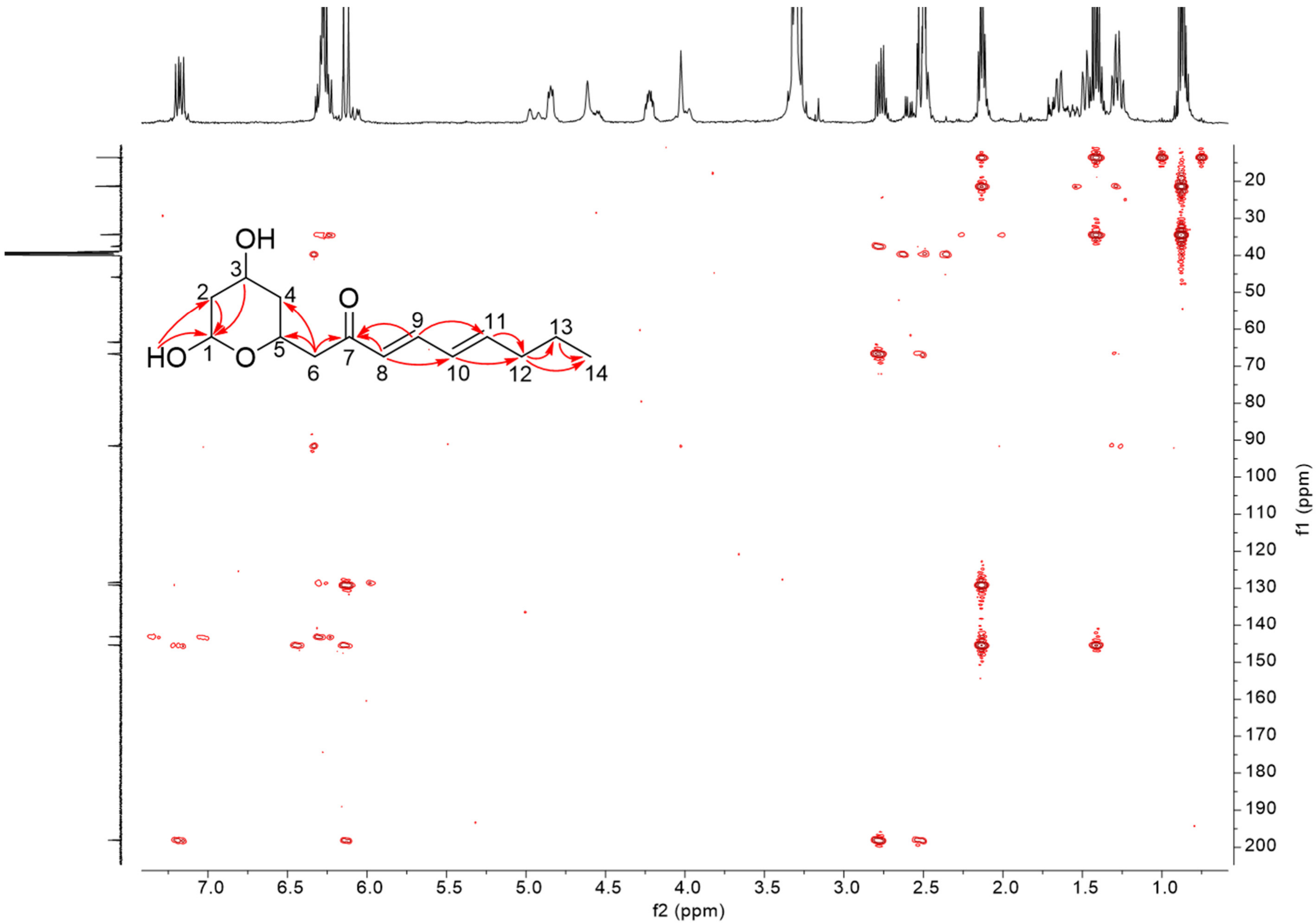

Figure S47. HMBC spectrum of palitanol A (**6**) in DMSO-d_6.

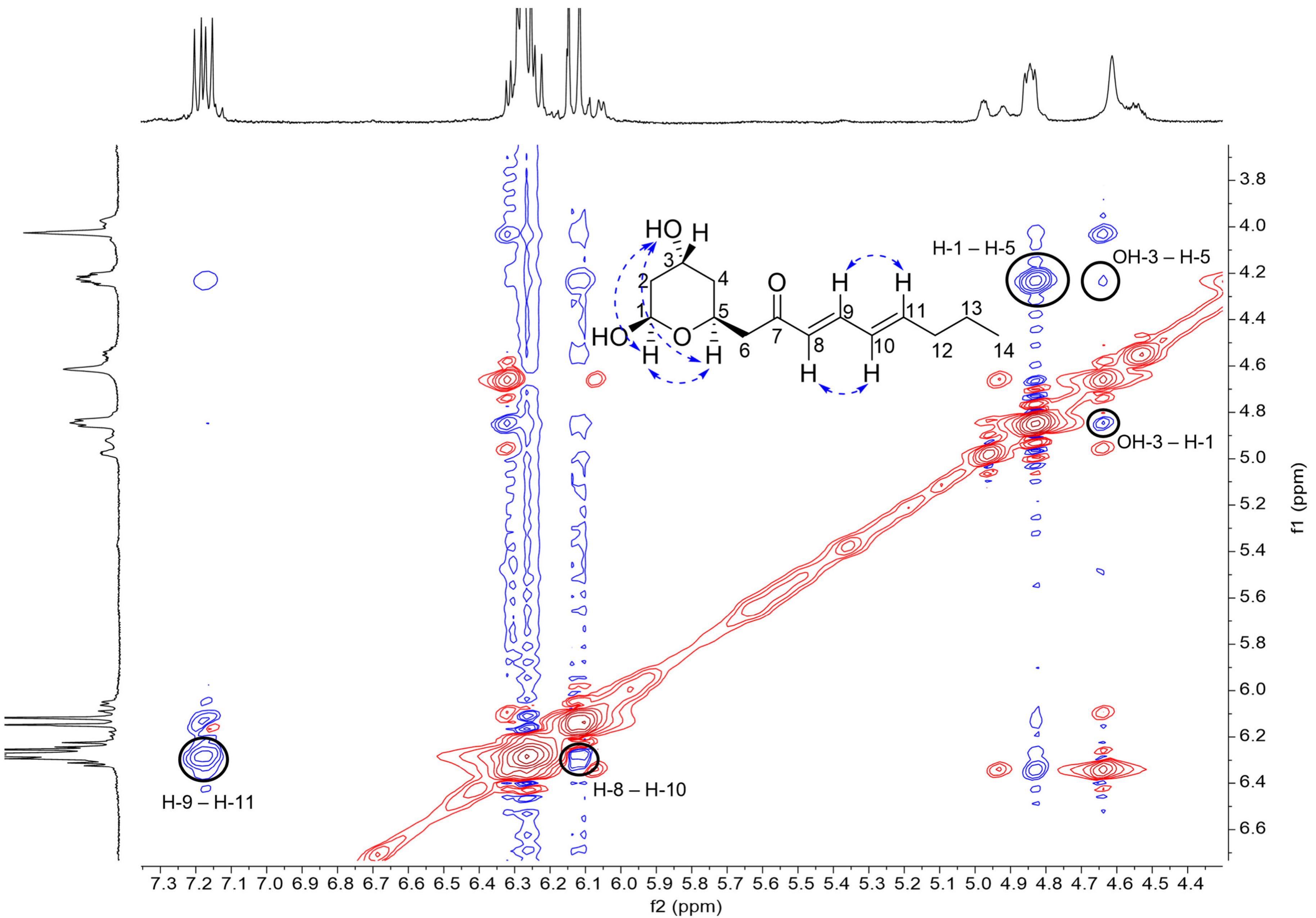

Figure S48. NOESY spectrum of palitanol A (**6**) in DMSO-d_6.

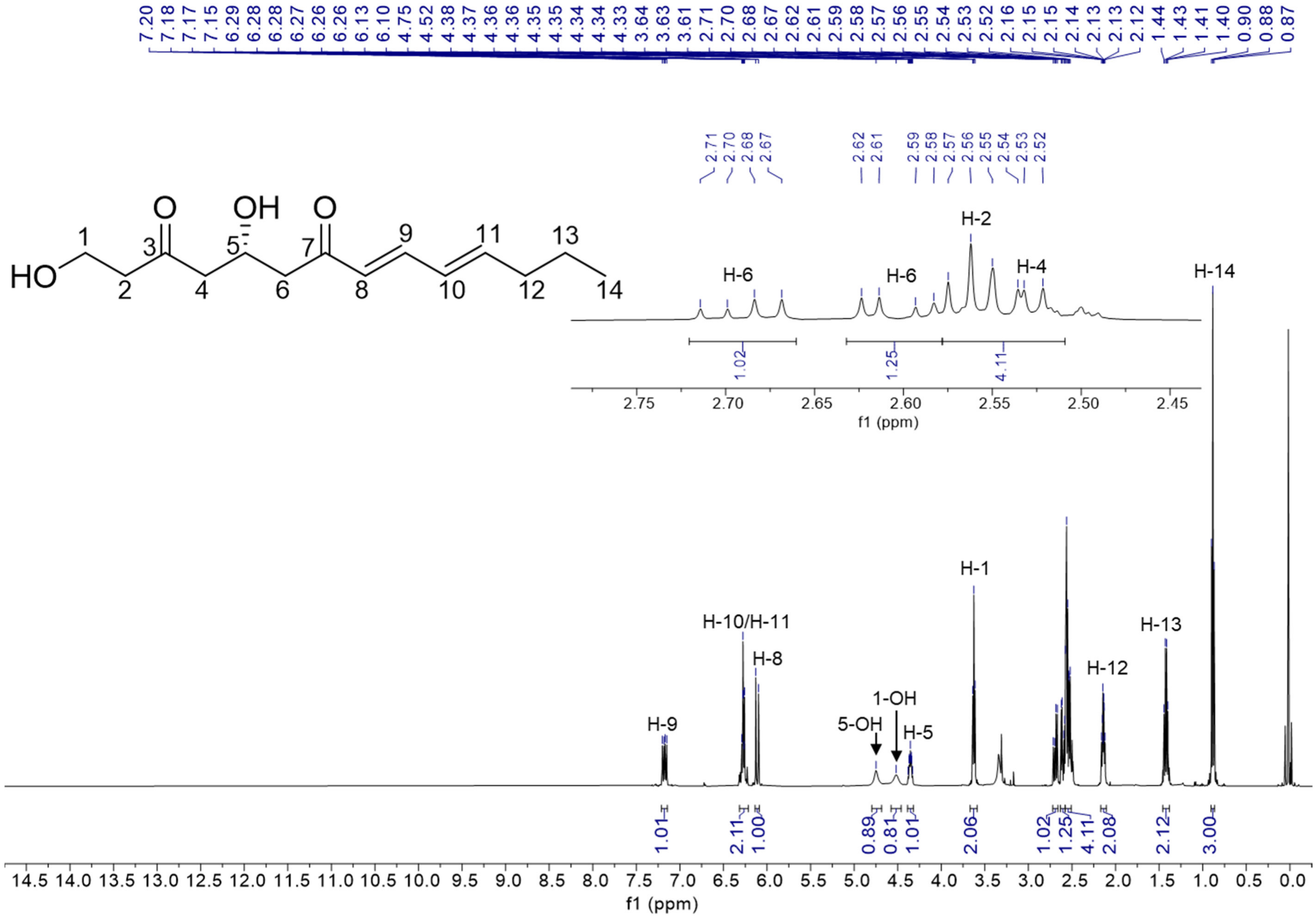

Figure S49. ^{1}H NMR spectrum of palitanol B (**7**) in DMSO-d_6 (500 MHz).

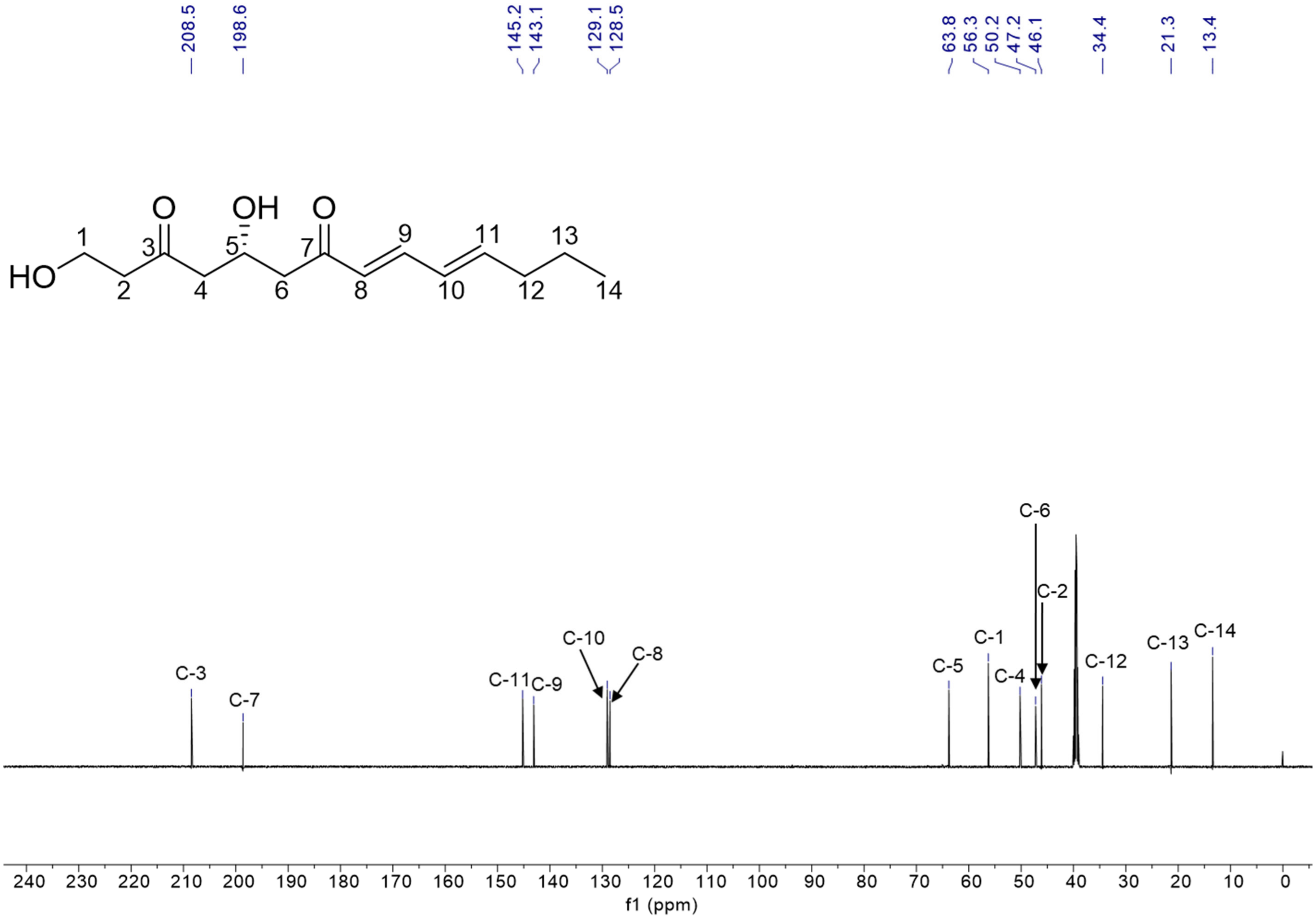

Figure S50. ^{13}C NMR spectrum of palitanol B (**7**) in DMSO-d_6 (125 MHz).

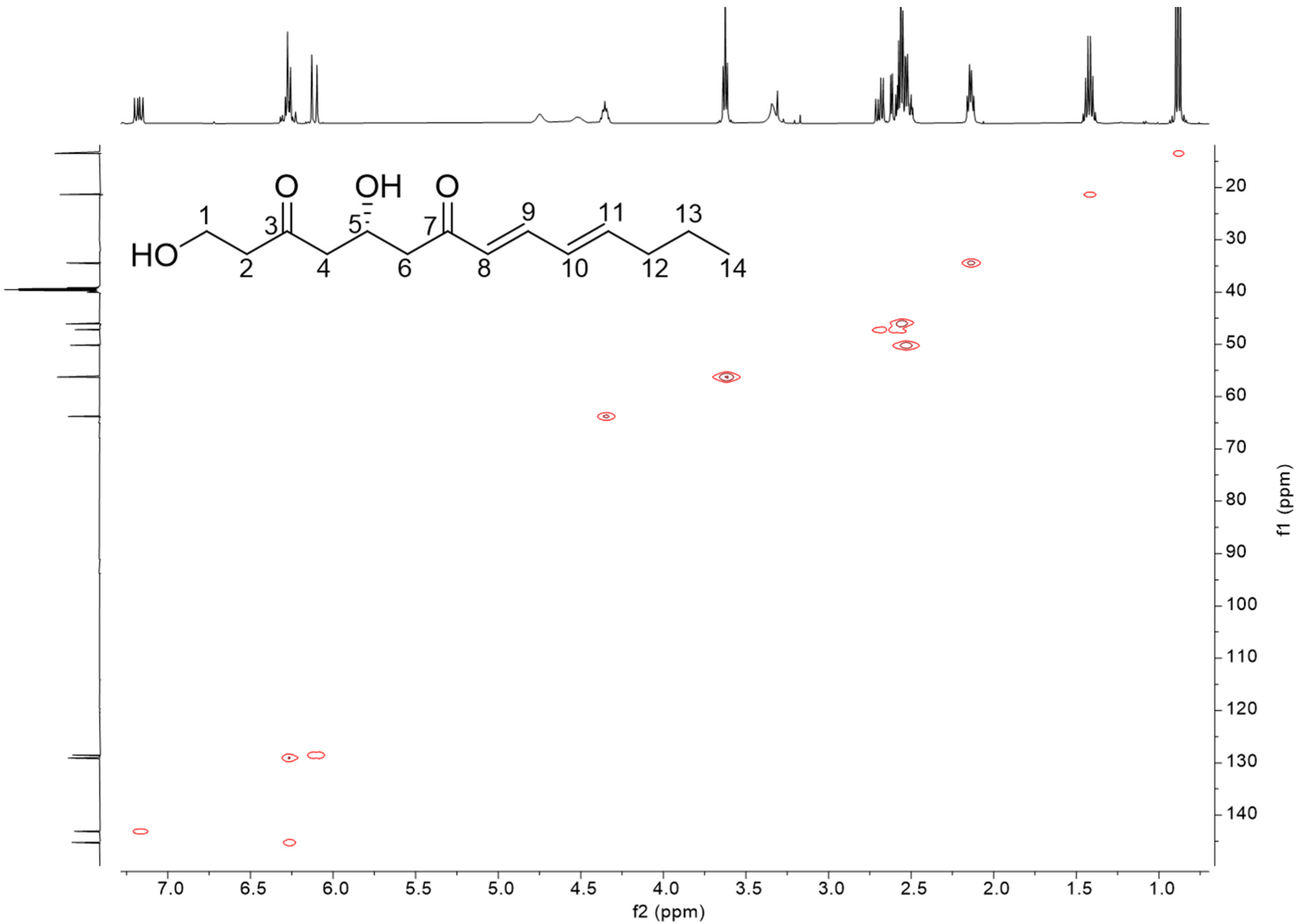

Figure S51. HSQC spectrum of palitanol B (**7**) in DMSO-*d*6.

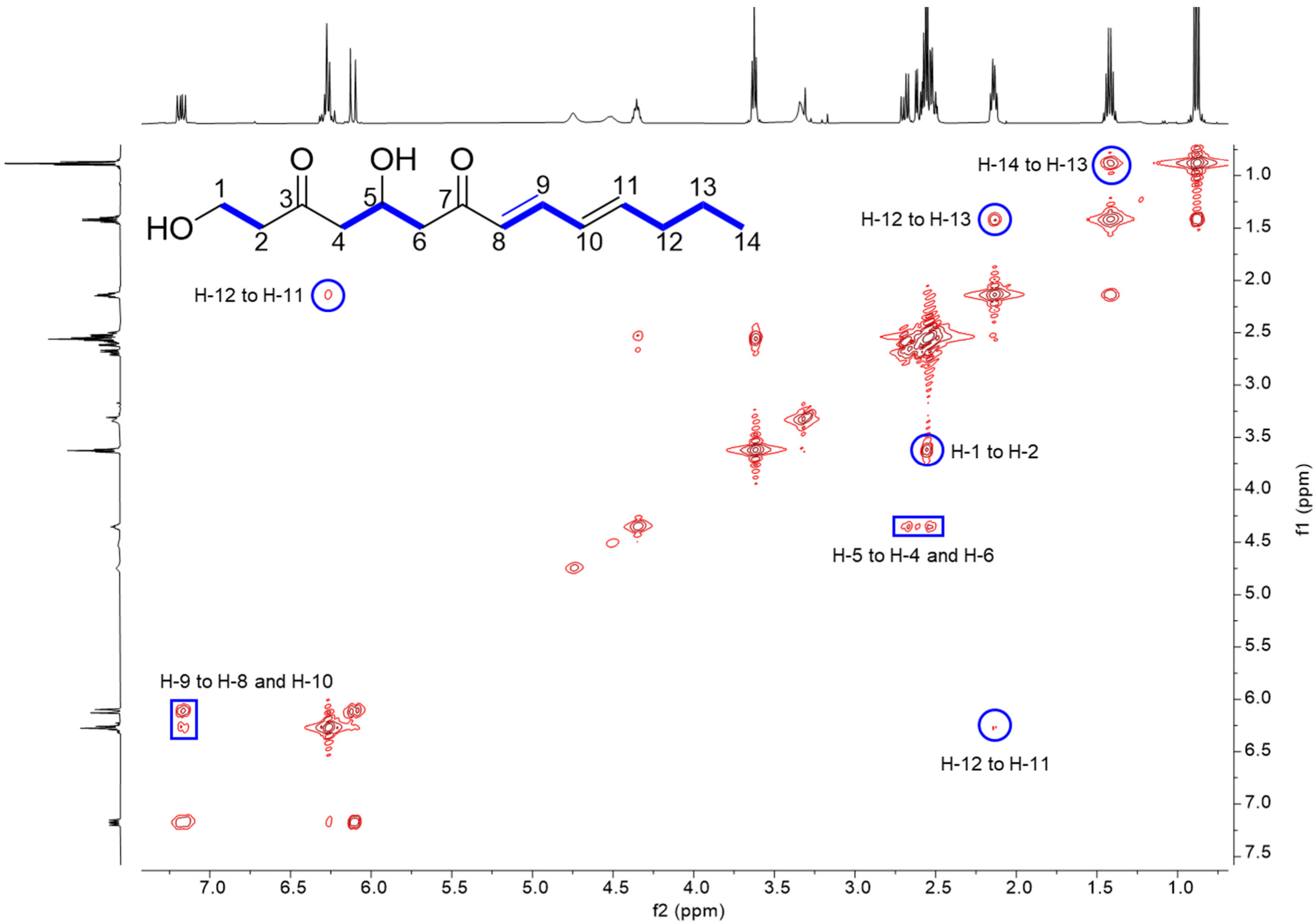

Figure S52. ¹H-¹H COSY spectrum of palitanol B (**7**) in DMSO-d_6.

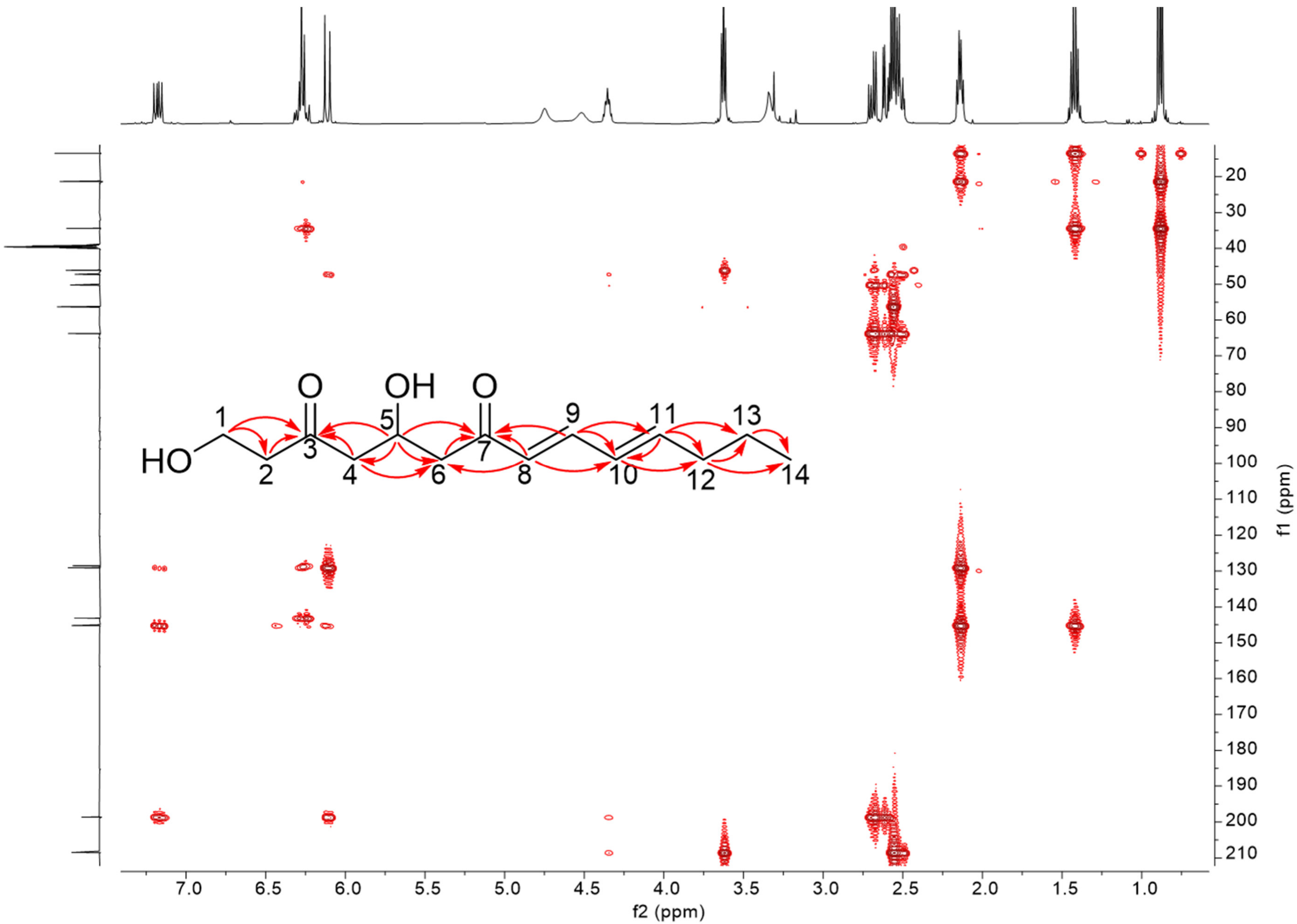

Figure S53. HMBC spectrum of palitanol B (**7**) in DMSO-d_6.

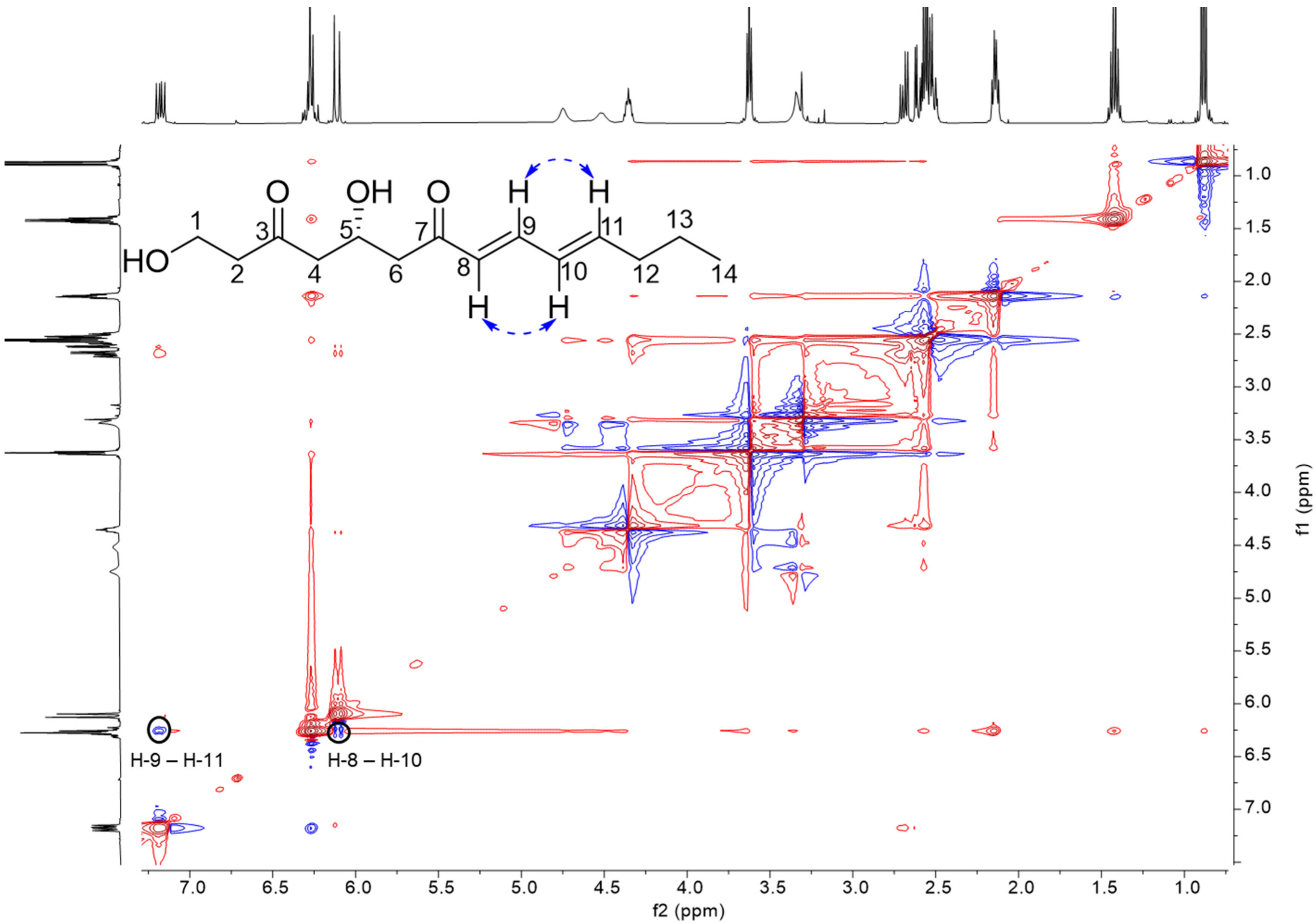

Figure S54. NOESY spectrum of palitanol B (**7**) in DMSO-d_6.

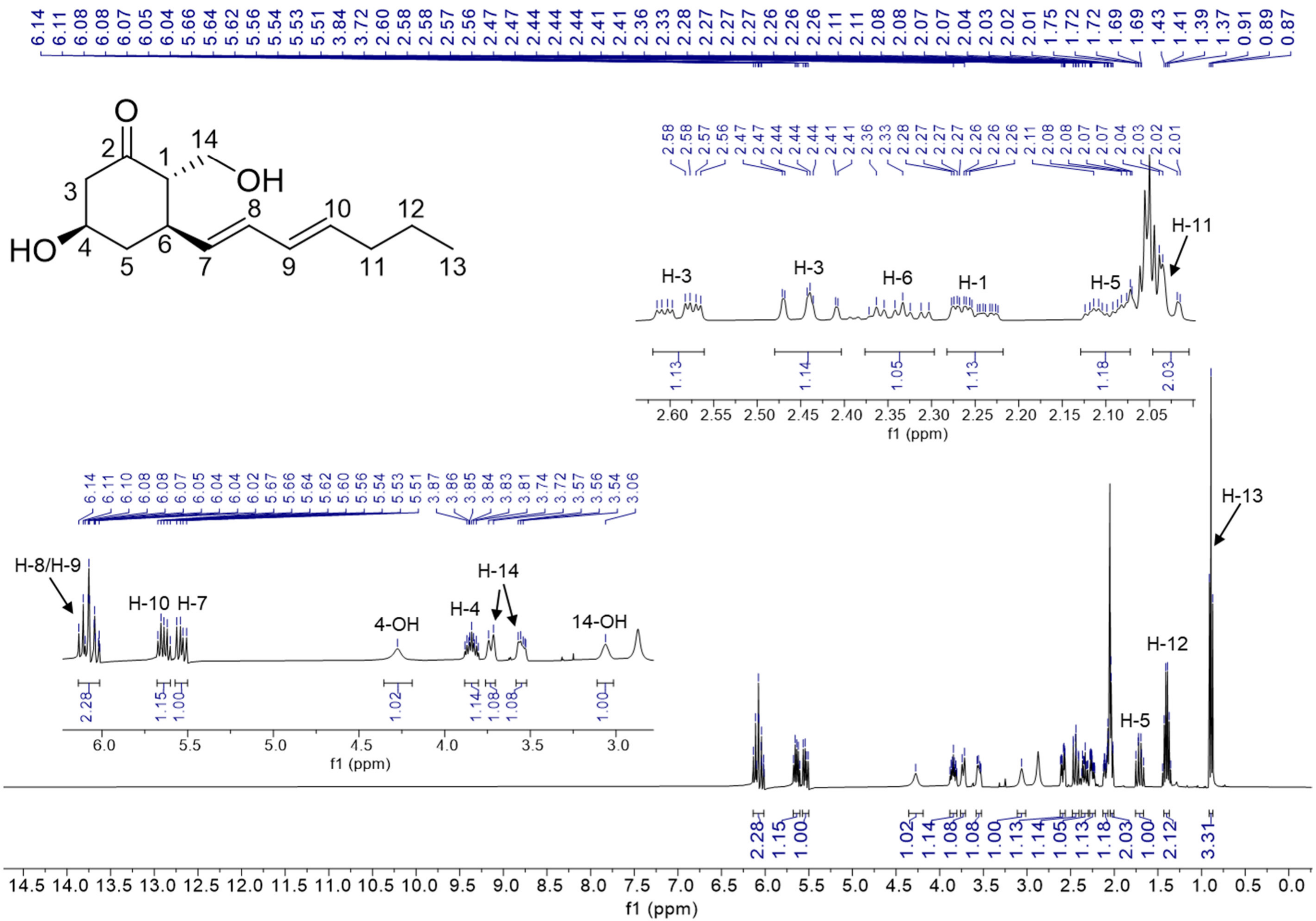

Figure S55. ^{1}H NMR spectrum of penihydrone C (**8**) in acetone-d_6 (400 MHz).

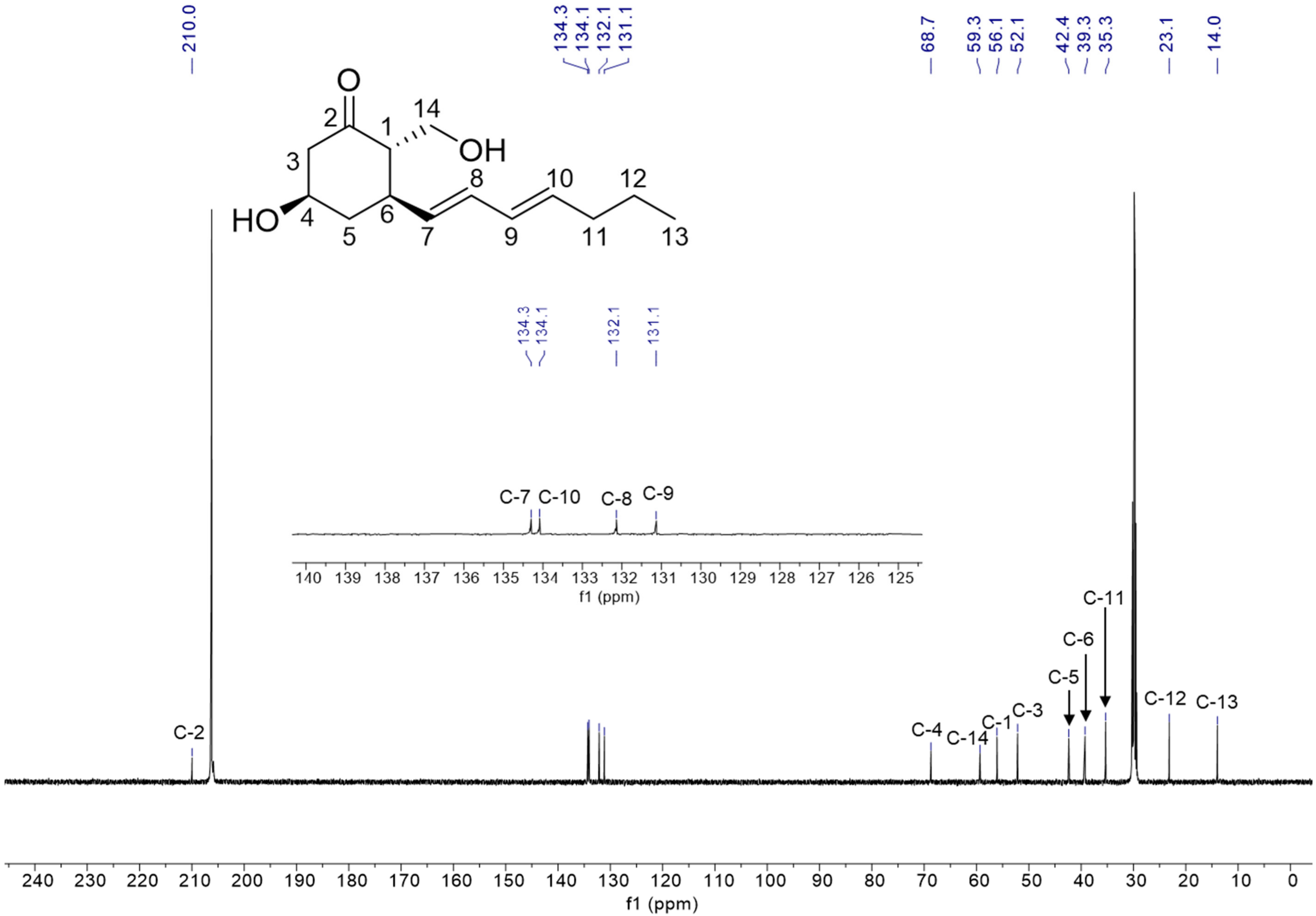

Figure S56. ^{13}C NMR spectrum of penihydrone C (**8**) in acetone-d_6 (125 MHz).

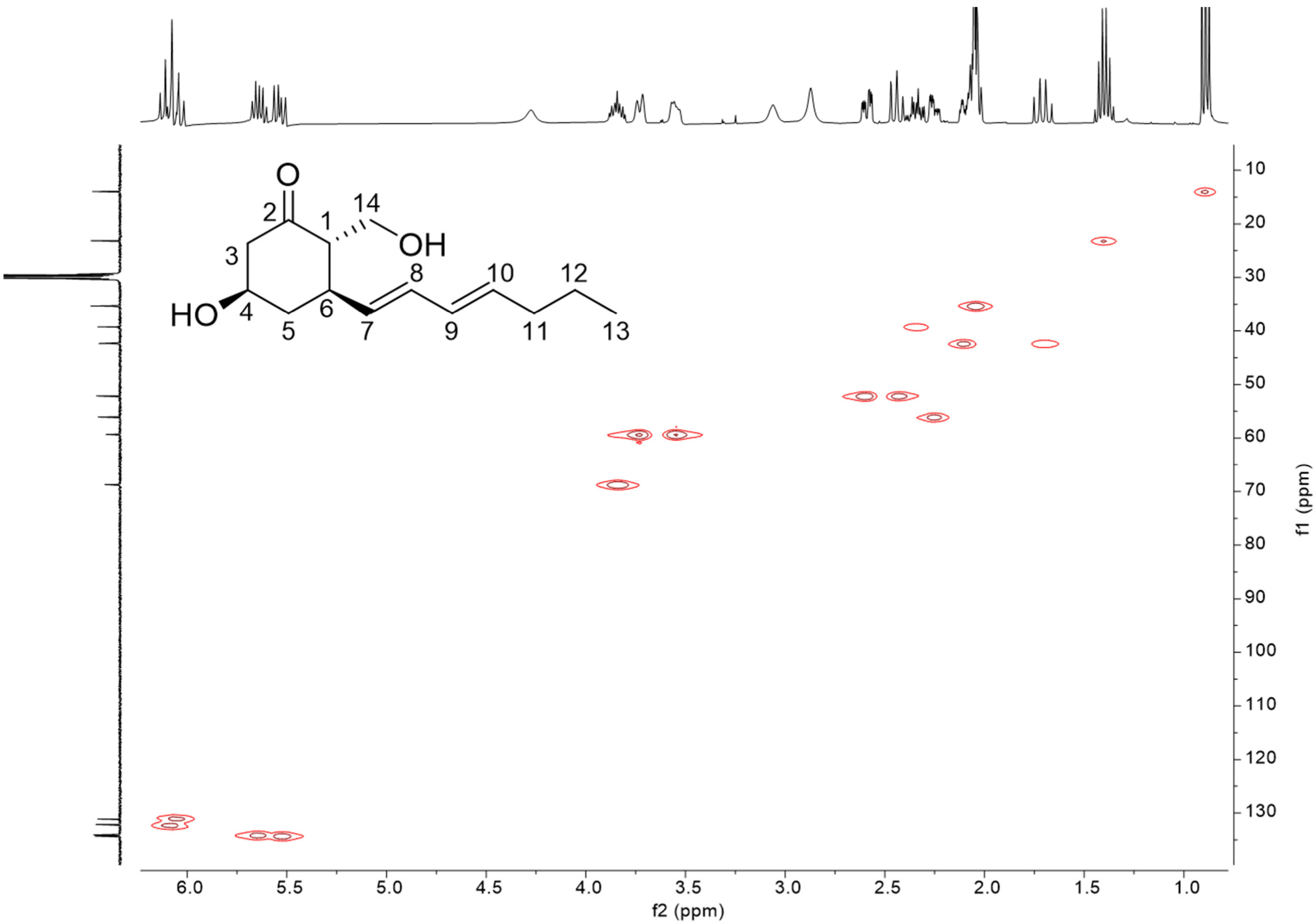

Figure S57. HSQC spectrum of penihydrone C (**8**) in acetone-d_6.

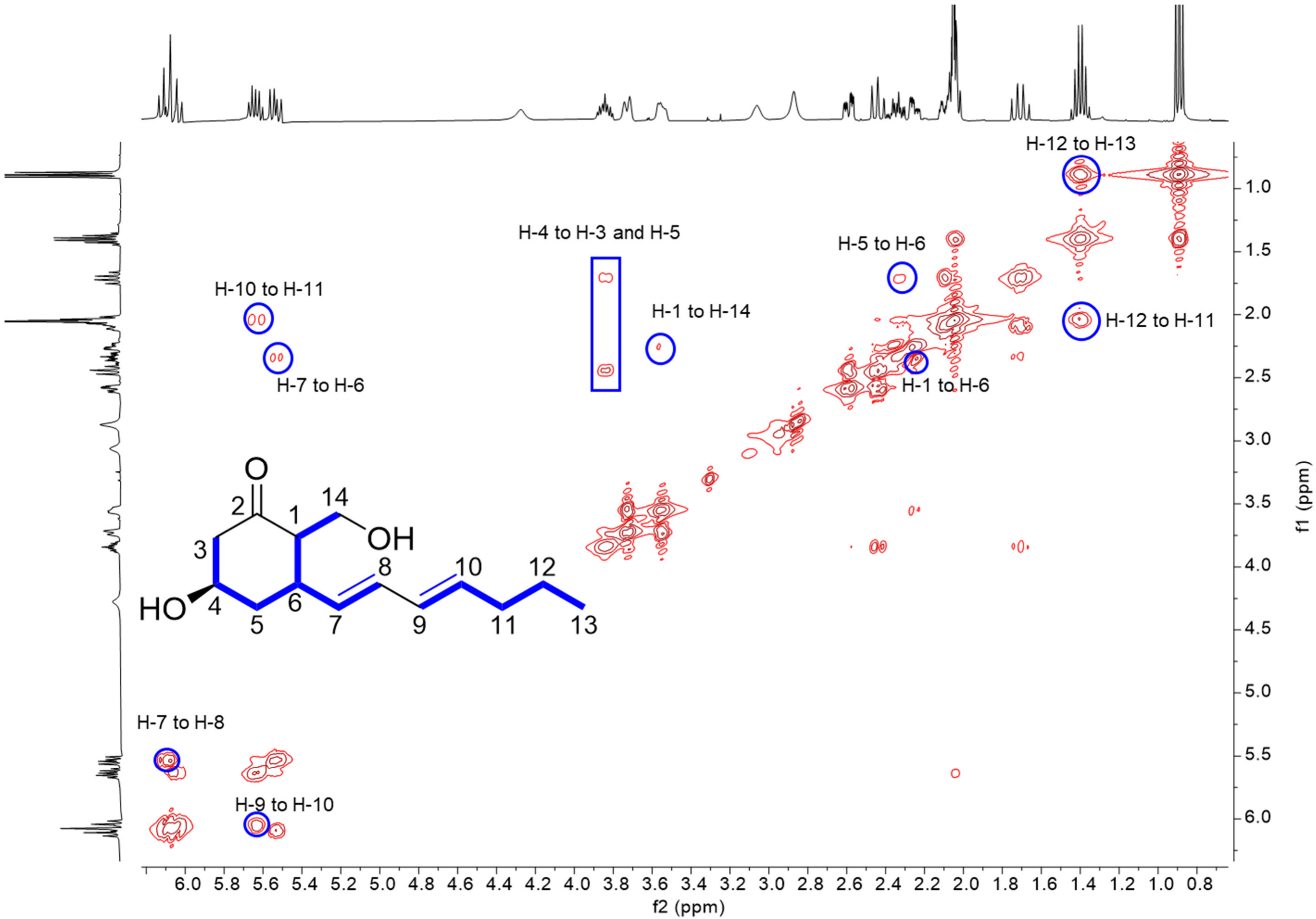

Figure S58. ^{1}H-^{1}H COSY spectrum of penihydrone C (**8**) in acetone-d_6.

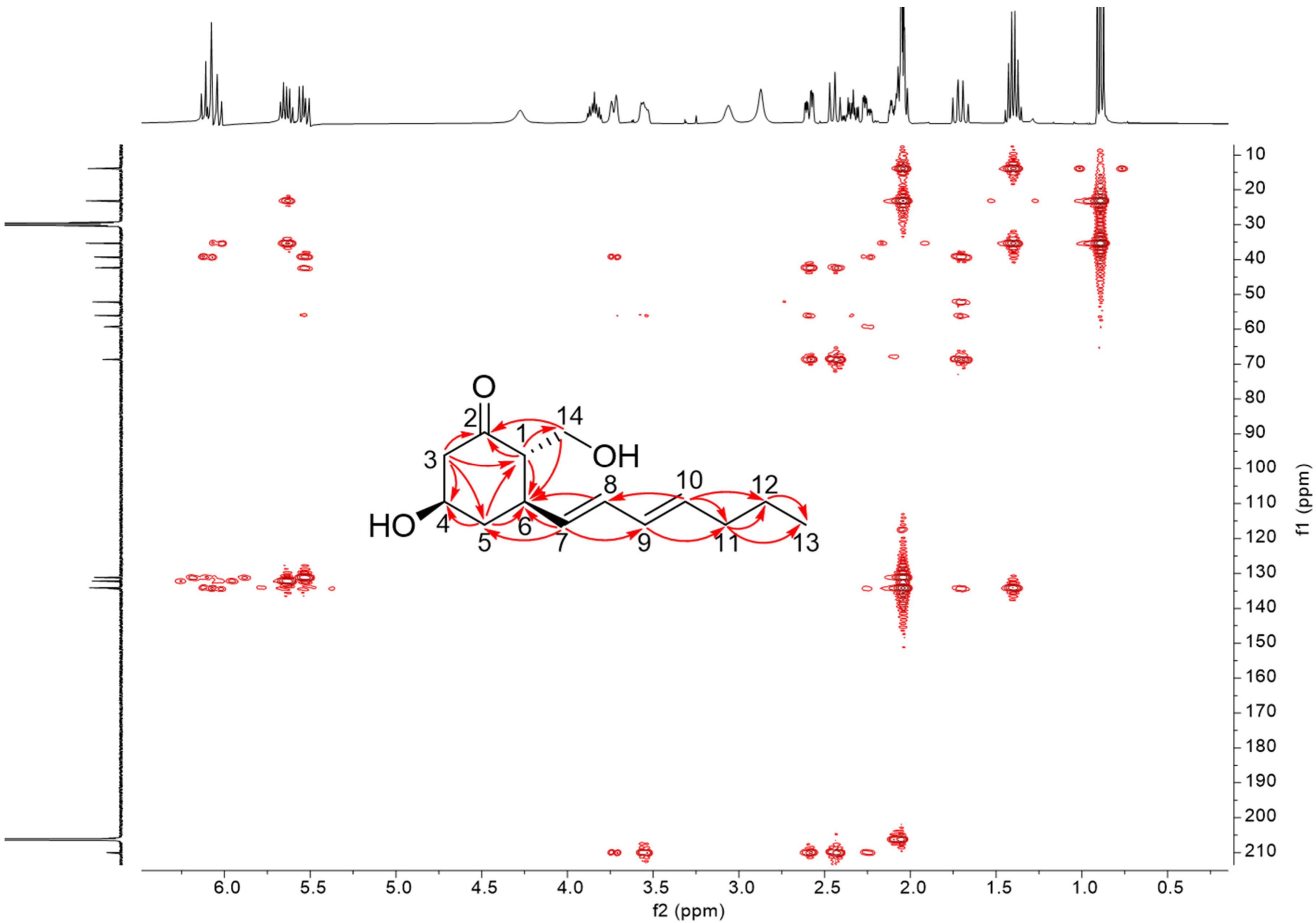

Figure S59. HMBC spectrum of penihydrone C (**8**) in acetone-d_6.

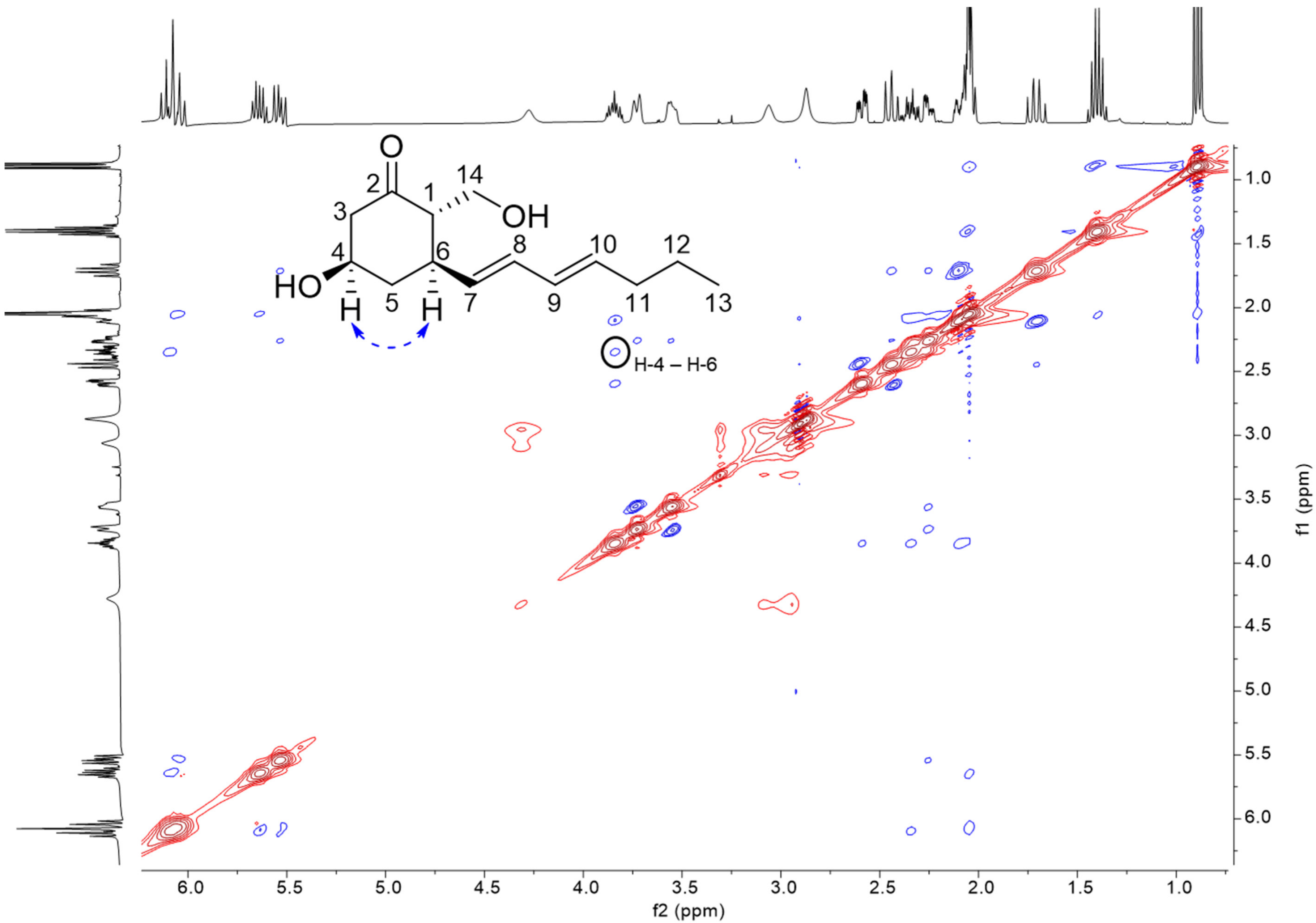

Figure S60. NOESY spectrum of penihydrone C (**8**) in acetone-d_6.

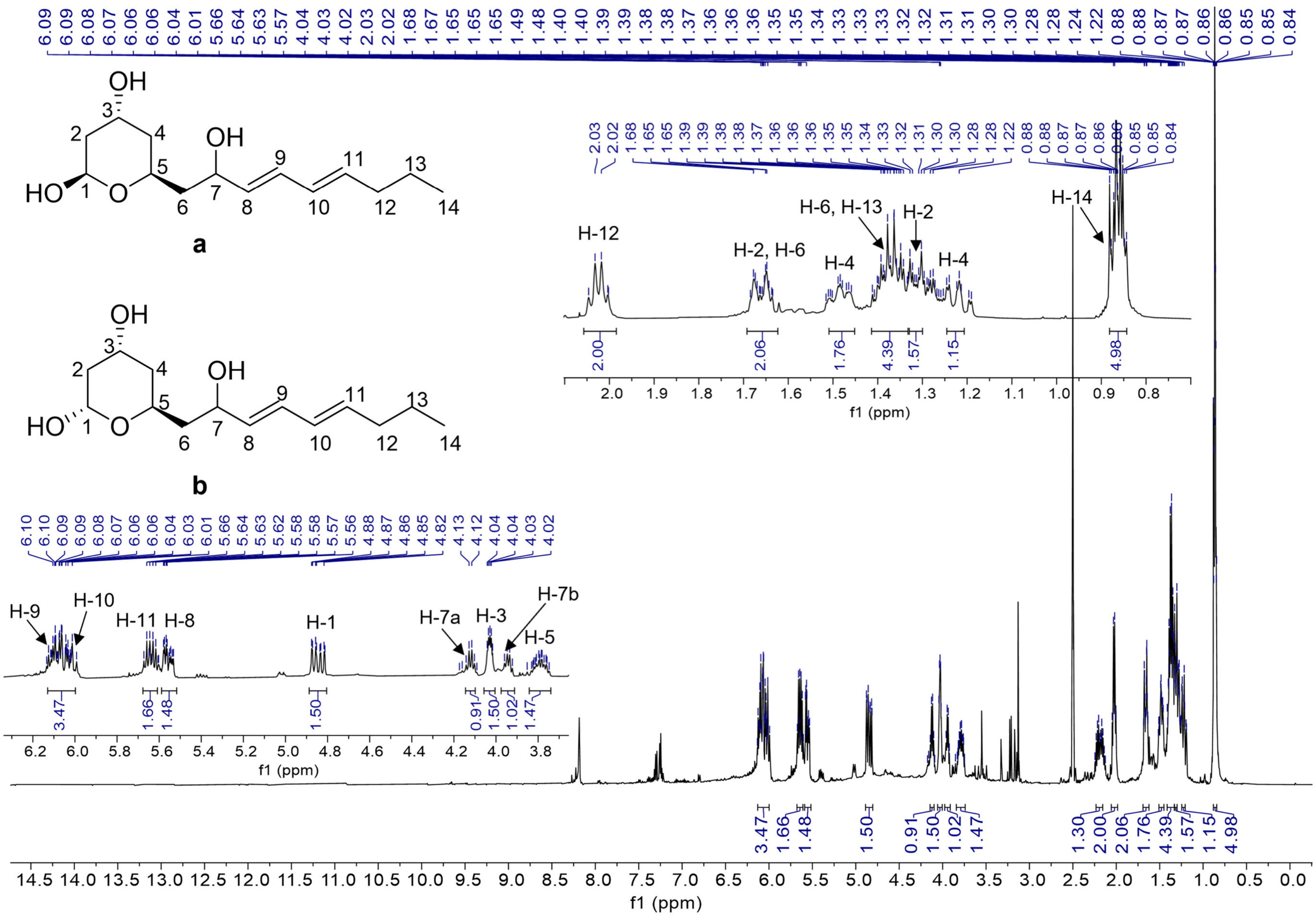

Figure S61. ^{1}H NMR spectrum of palitanol C (**9**) in DMSO-d_6 (500 MHz). Labelling without a or b means signals for both structures.

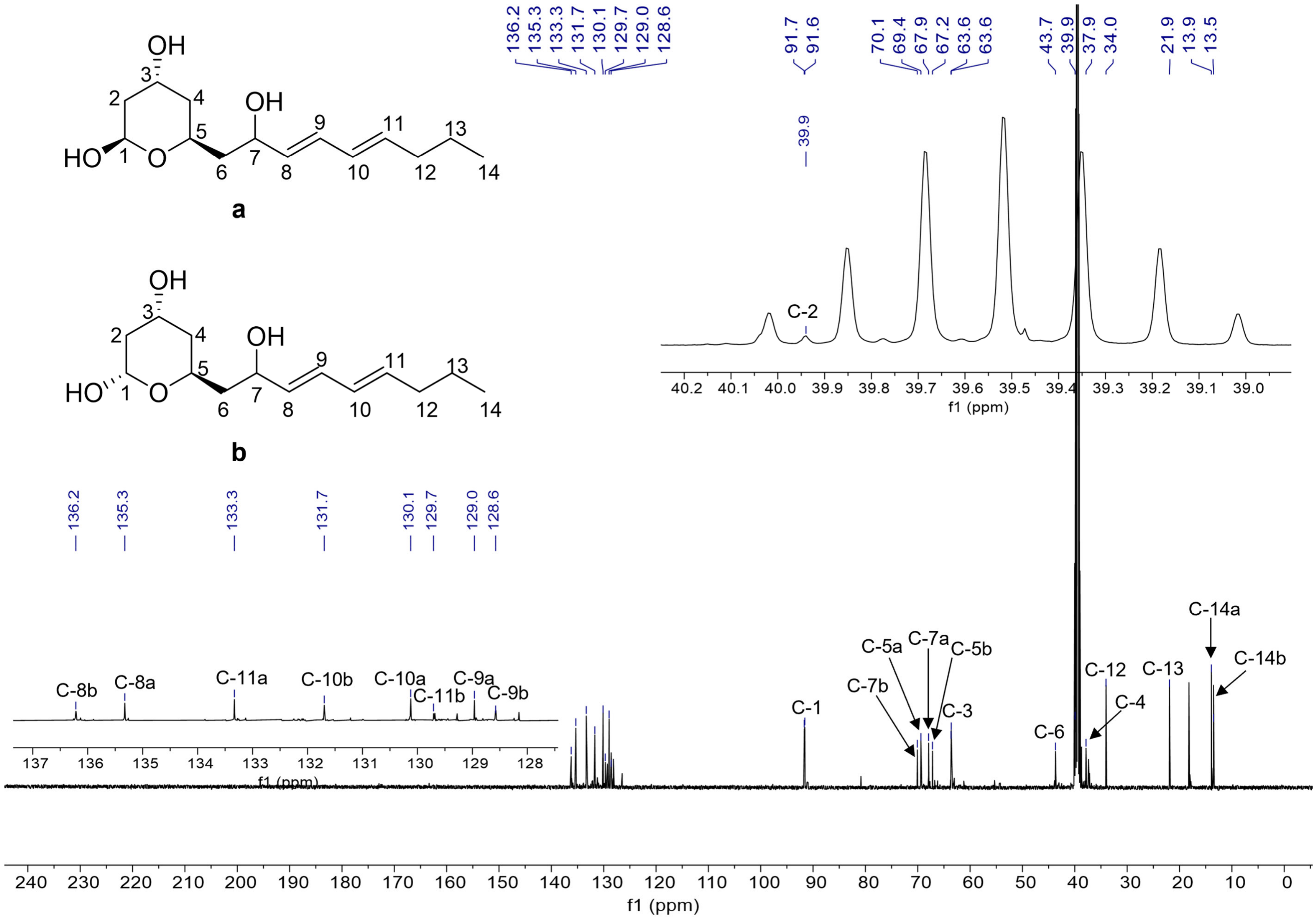

Figure S62. ^{13}C NMR spectrum of palitanol C (**9**) in DMSO-d_6 (125 MHz). Labelling without a or b means signals for both structures.

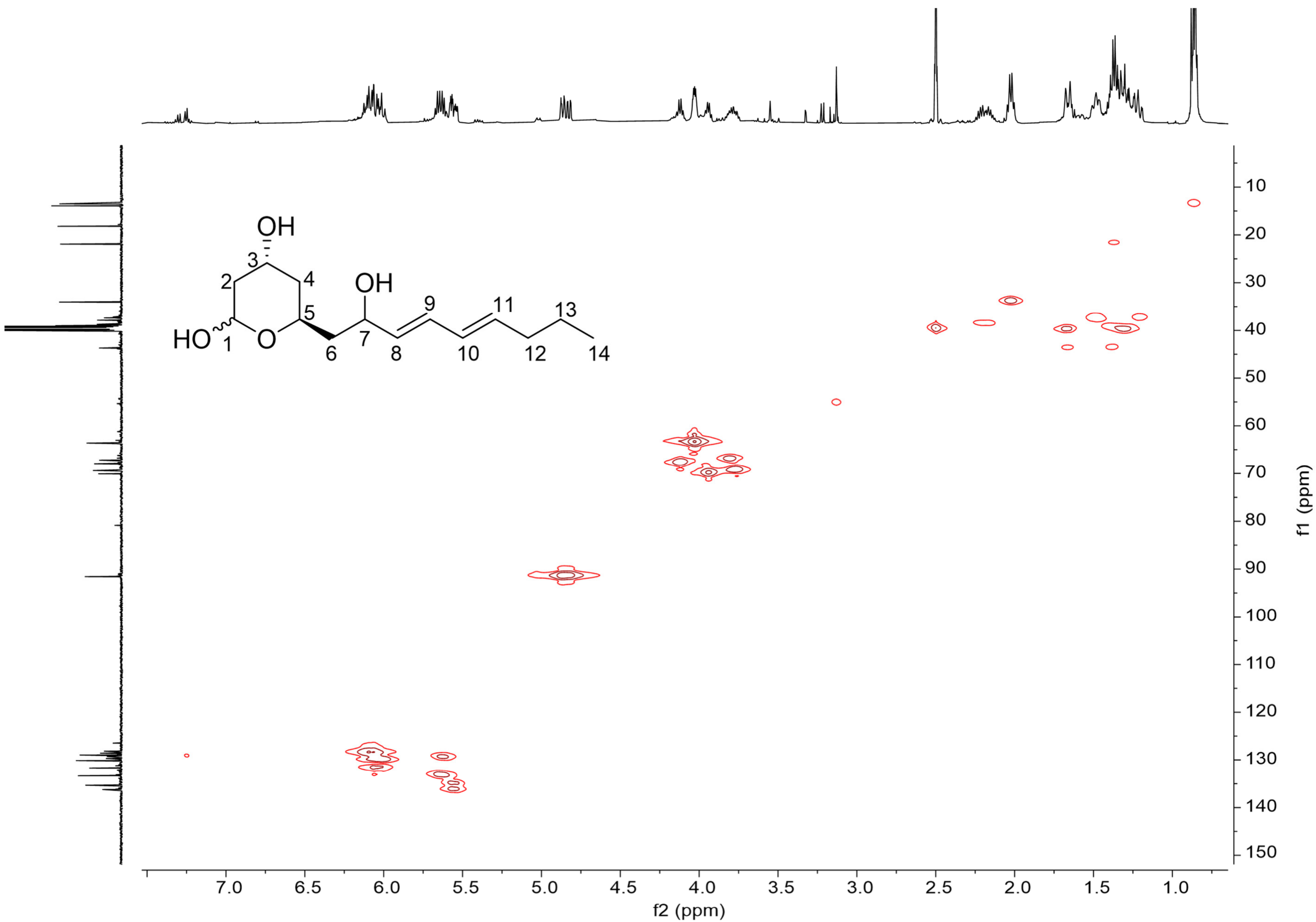

Figure S63. HSQC spectrum of palitanol C (**9**) in DMSO-*d*6.

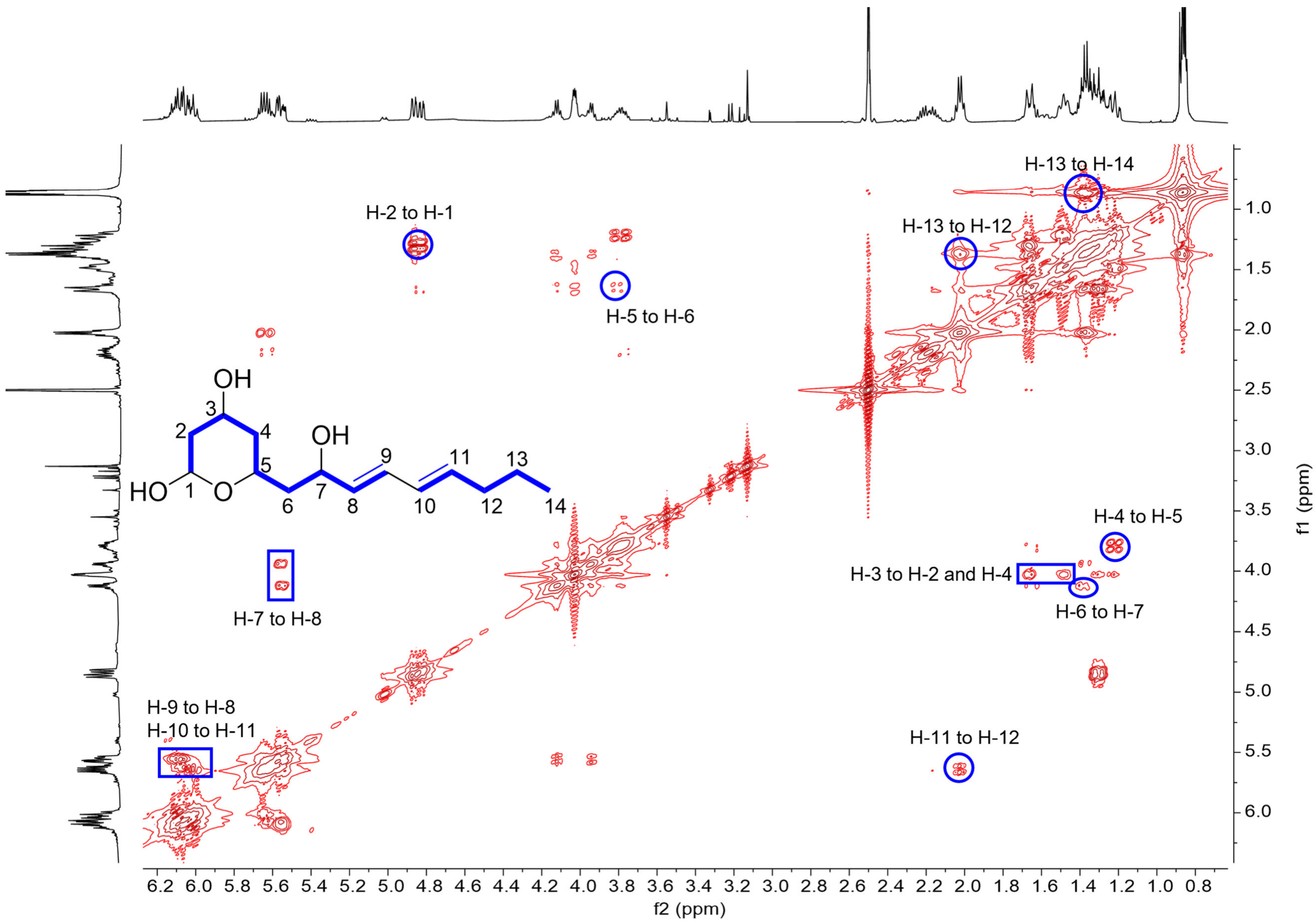

Figure S64. ^1H-^1H COSY spectrum of palitanol C (**9**) in DMSO-d_6.

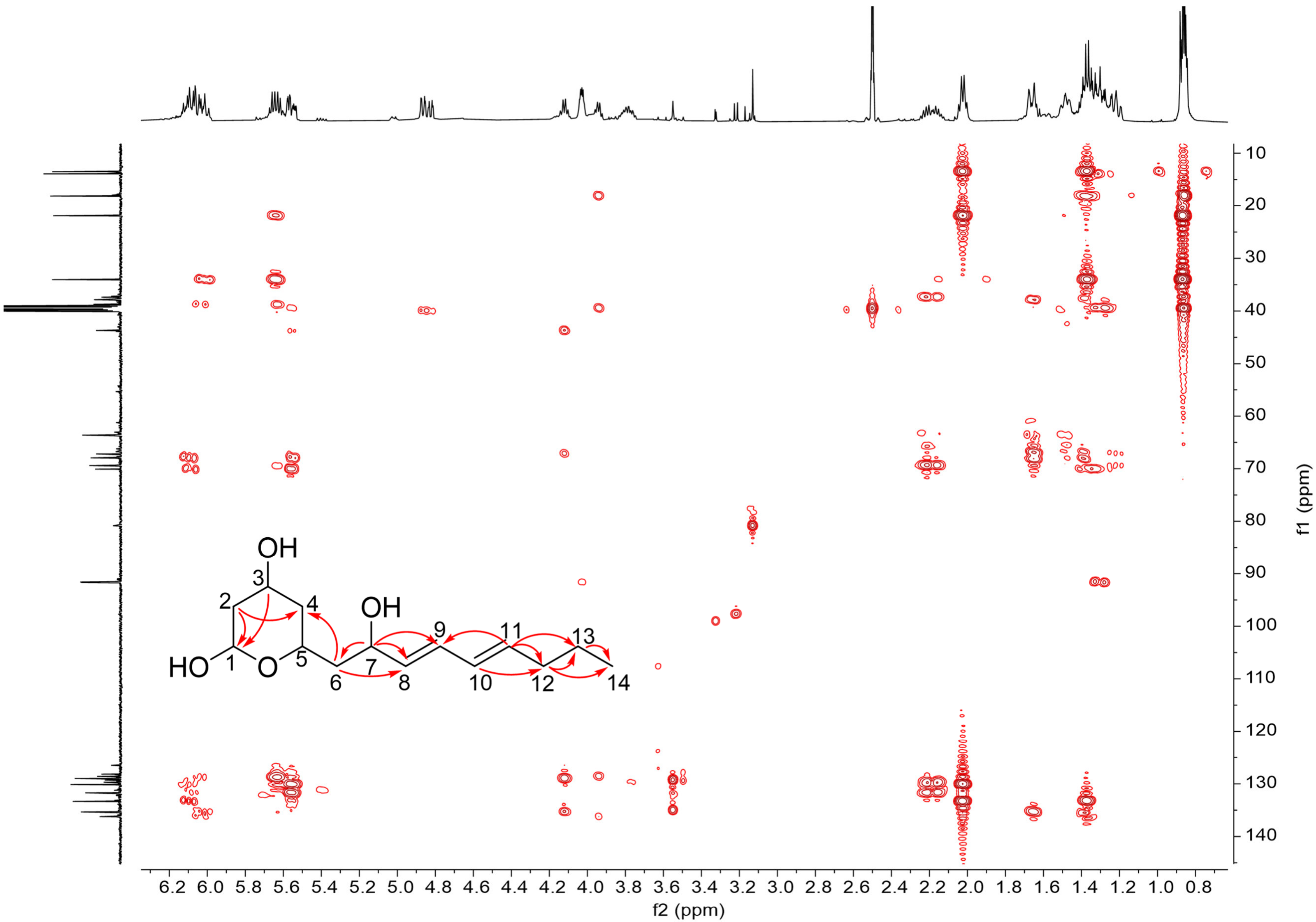

Figure S65. HMBC spectrum of palitanol C (**9**) in DMSO-d_6.

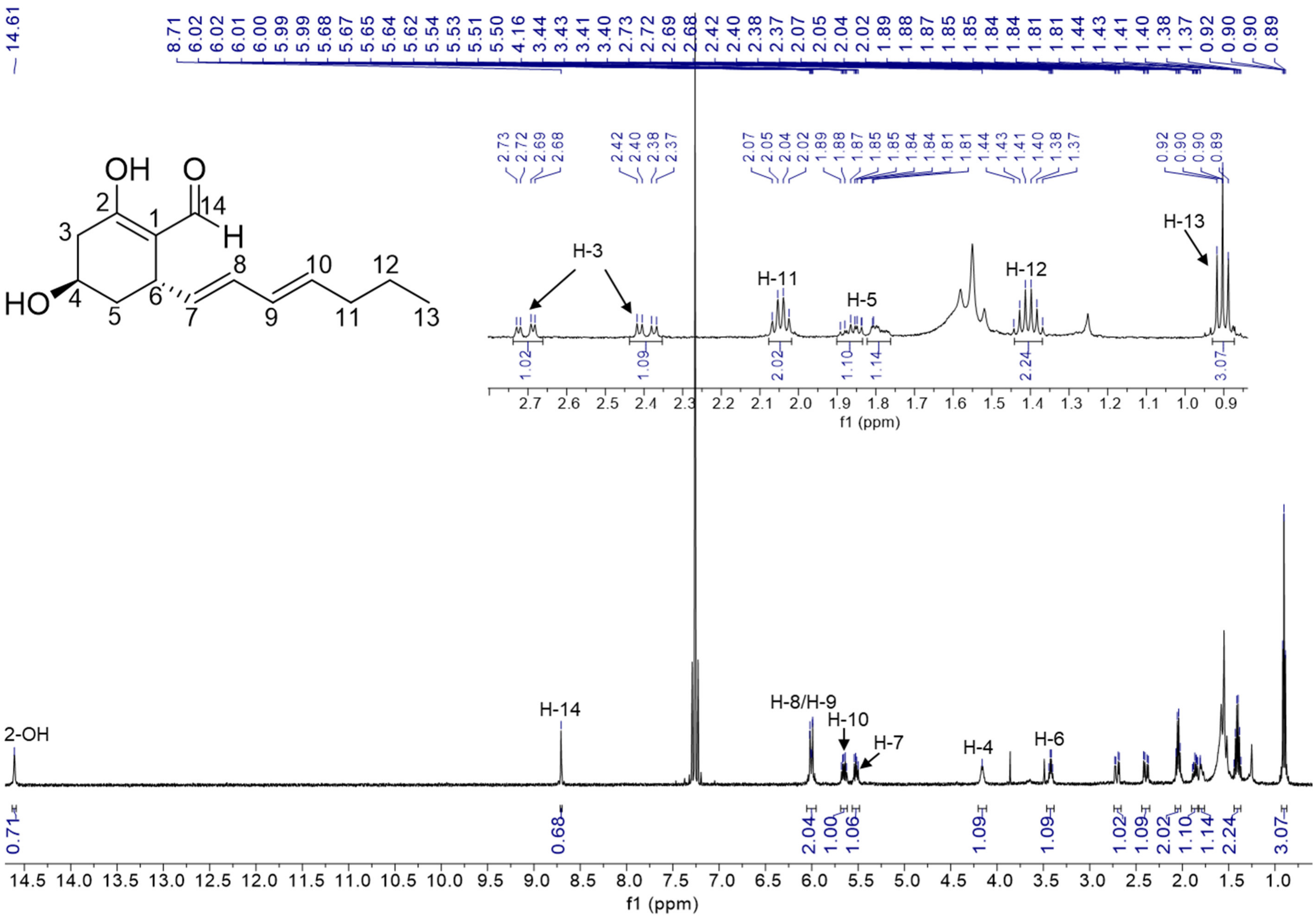

Figure S66. ^{1}H NMR spectrum of penihydronal (**12**) in $CDCl_3$ (500 MHz).

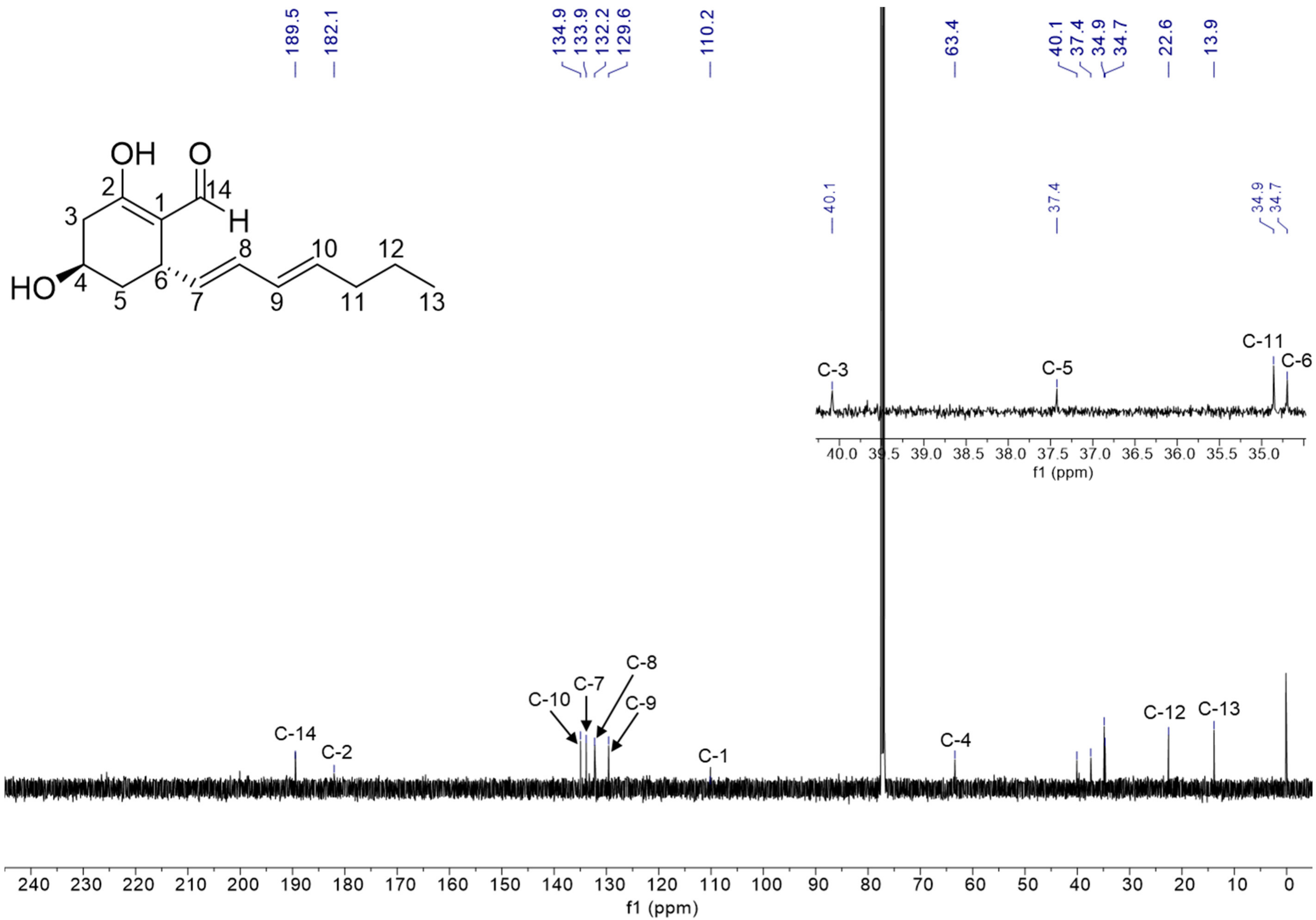

Figure S67. ^{13}C NMR spectrum of penihydronal (**12**) in $CDCl_3$ (125 MHz).

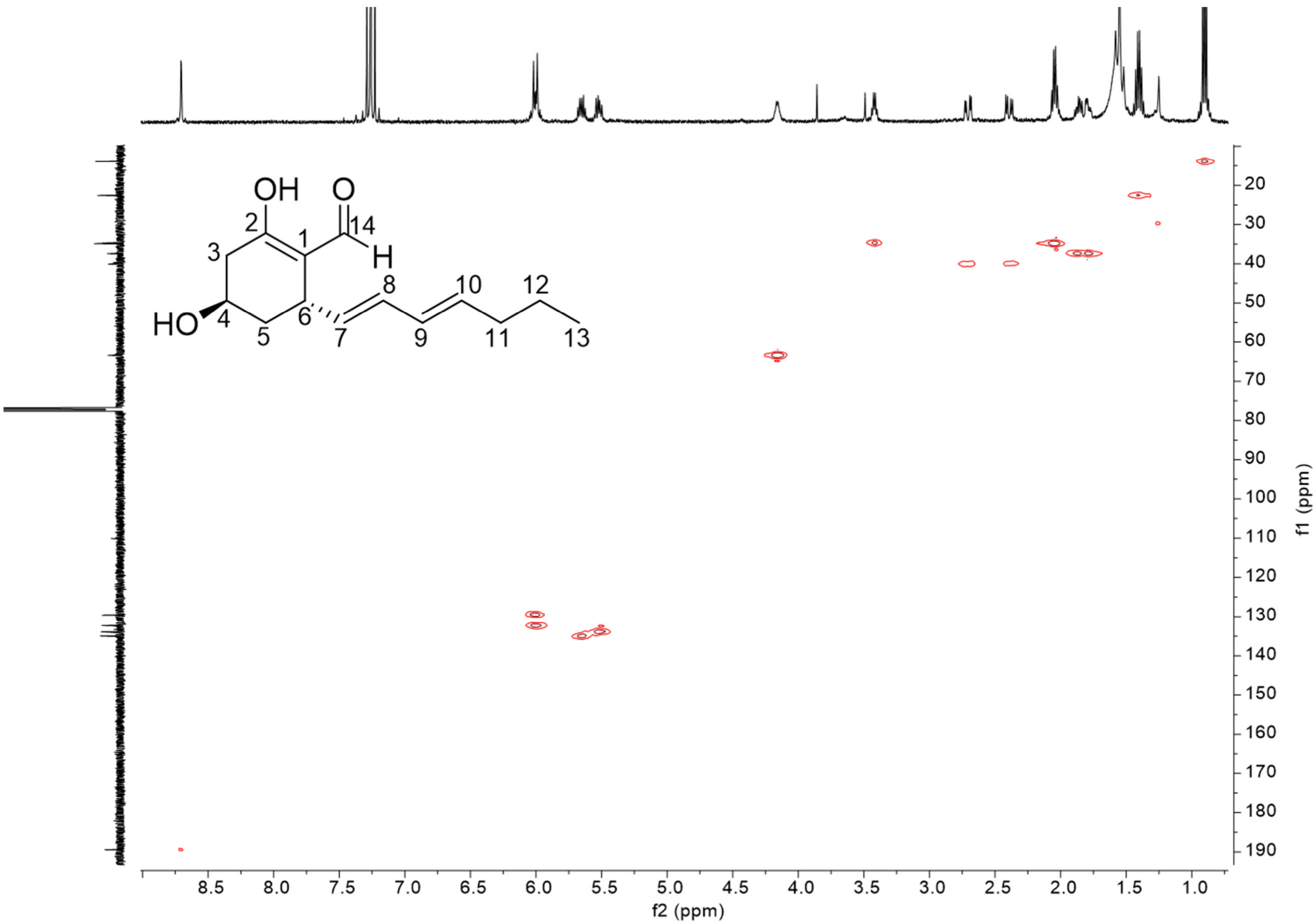

Figure S68. HSQC spectrum of penihydronal (**12**) in $CDCl_3$.

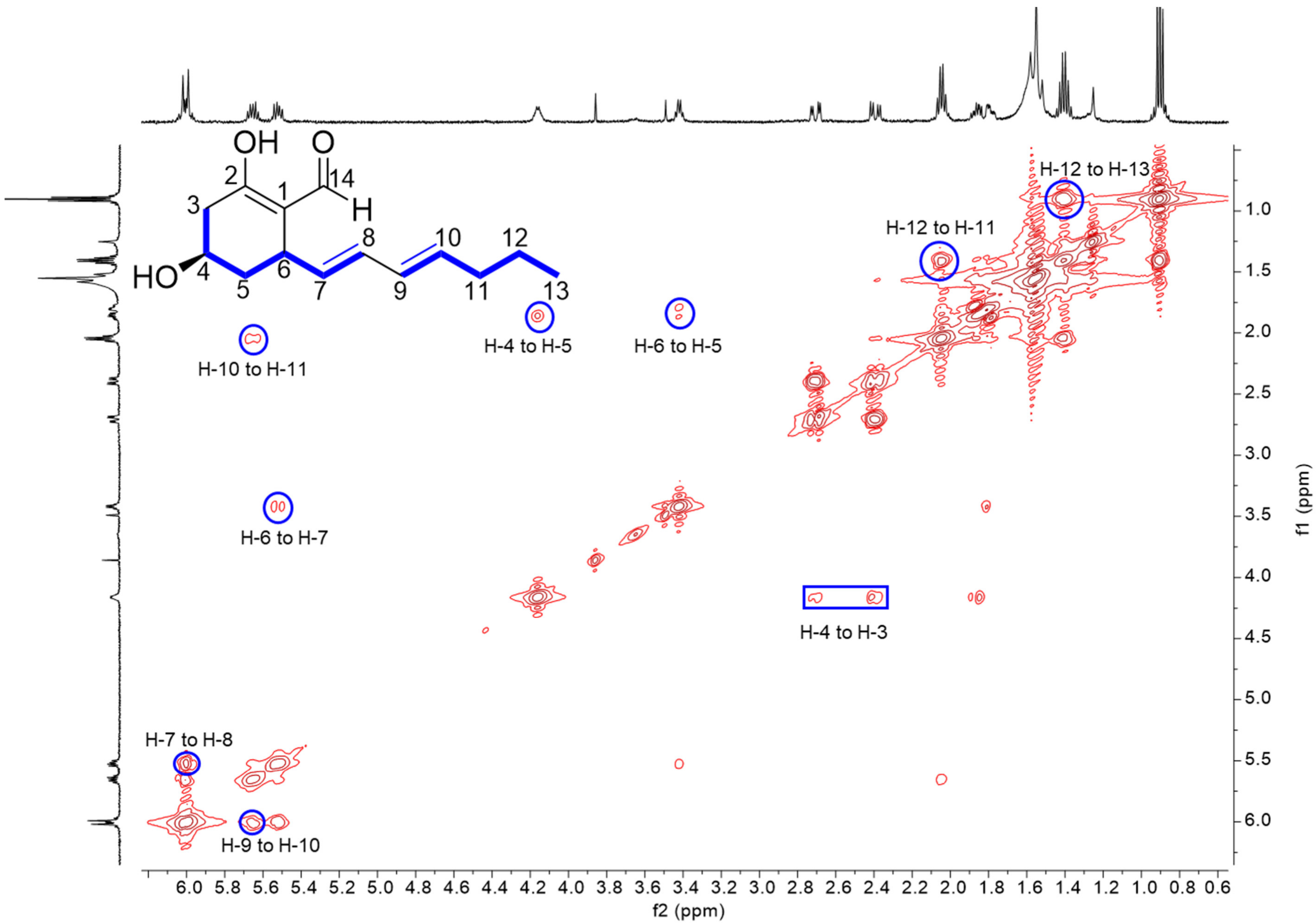

Figure S69. ^{1}H-^{1}H COSY spectrum of penihydronal (**12**) in $CDCl_3$.

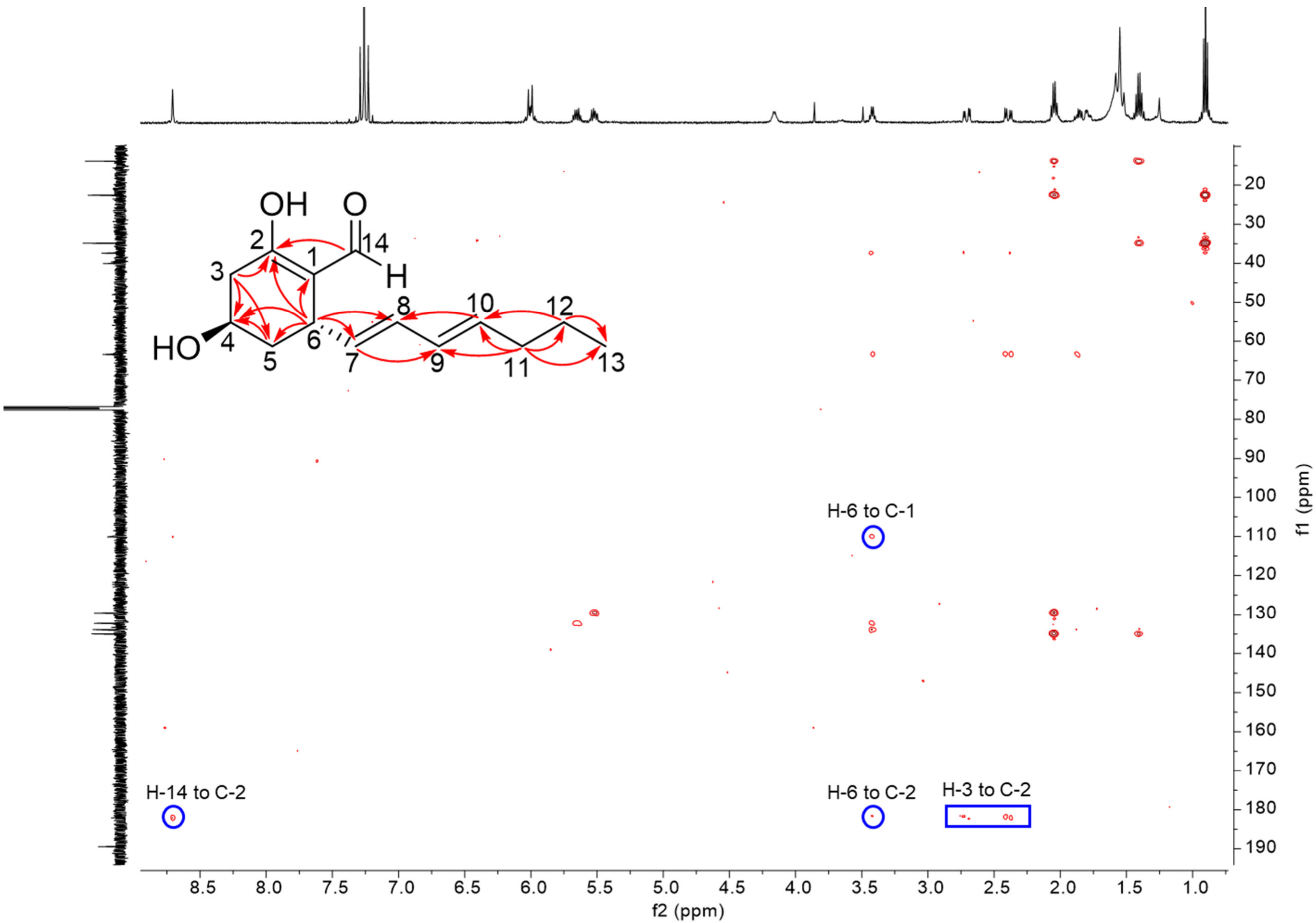

Figure S70. HMBC spectrum of penihydronal (**12**) in $CDCl_3$.

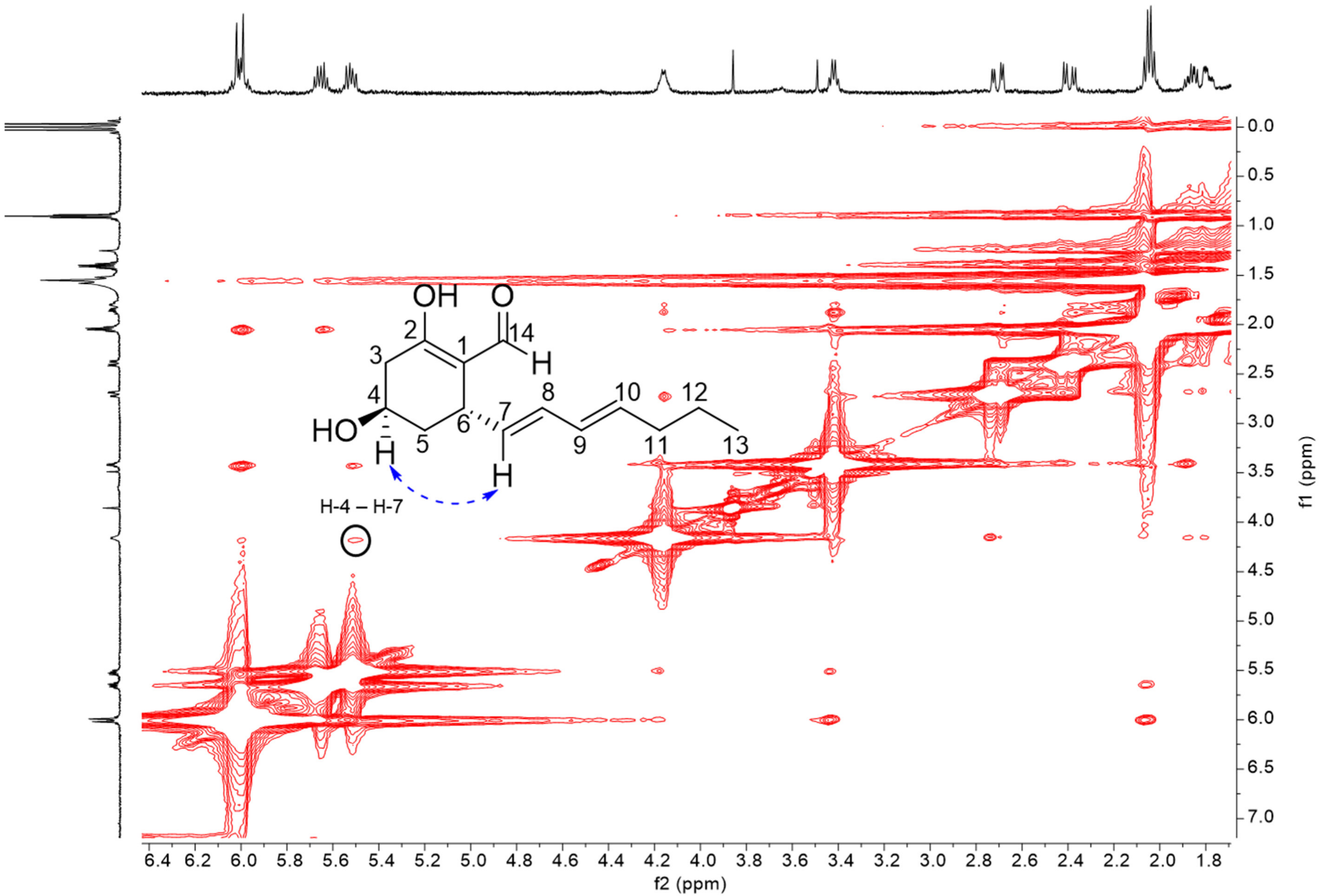

Figure S71. NOESY spectrum of penihydronal (**12**) in $CDCl_3$.

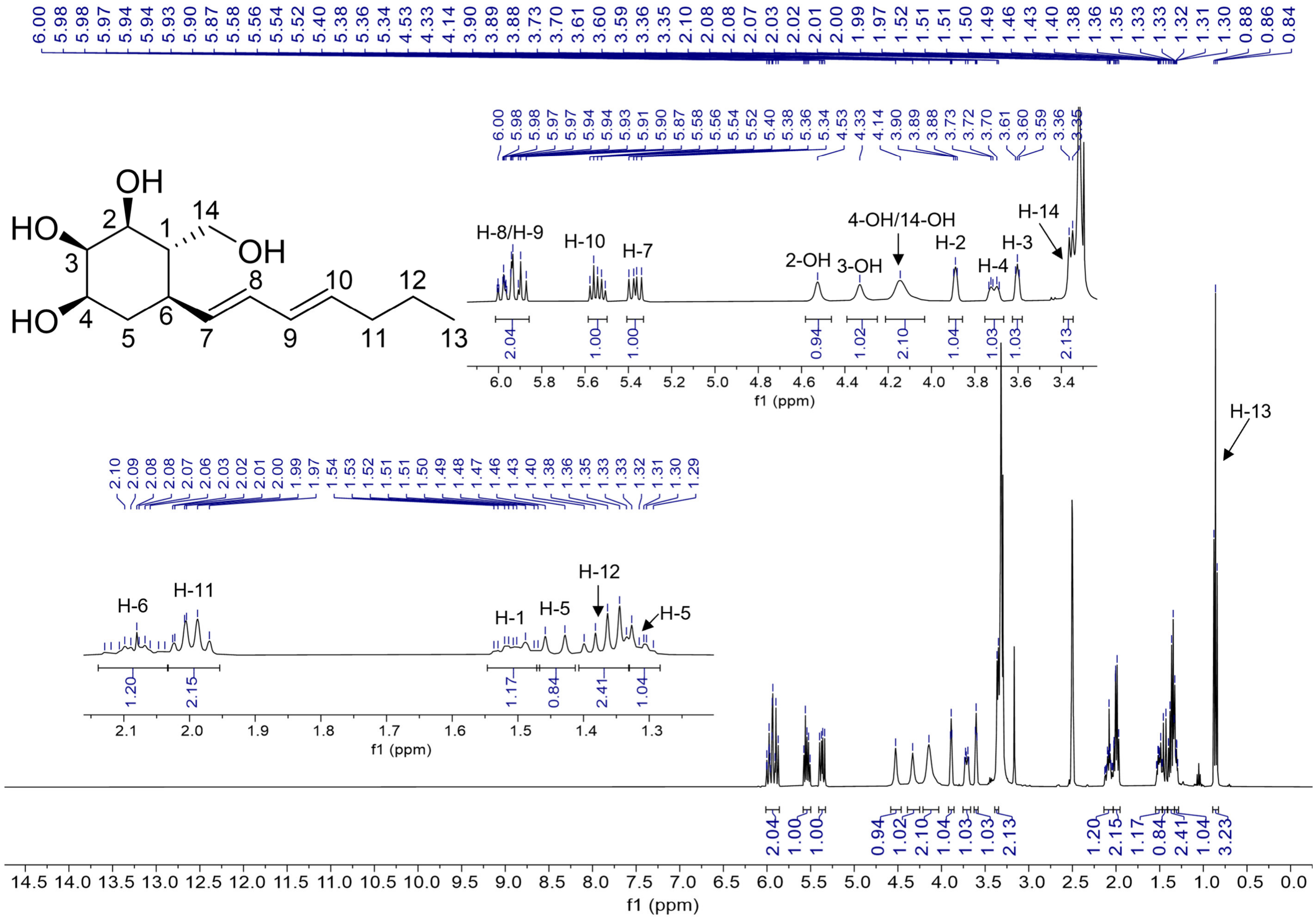

Figure S72. ^{1}H NMR spectrum of dia-palitantol (**16**) in DMSO-d_6 (400 MHz).

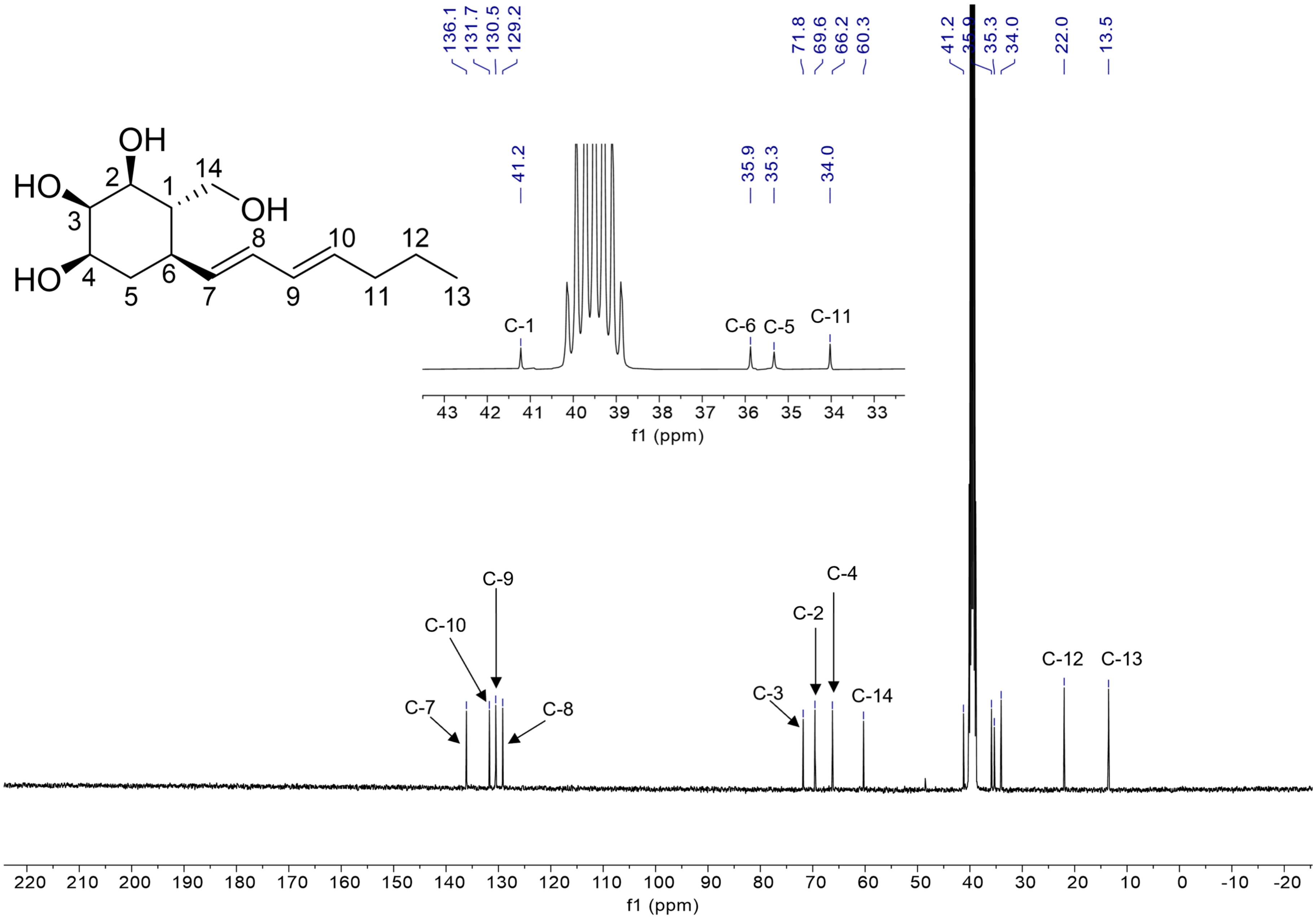

Figure S73. ^{13}C NMR spectrum of dia-palitantol (**16**) in DMSO-d_6 (100 MHz).

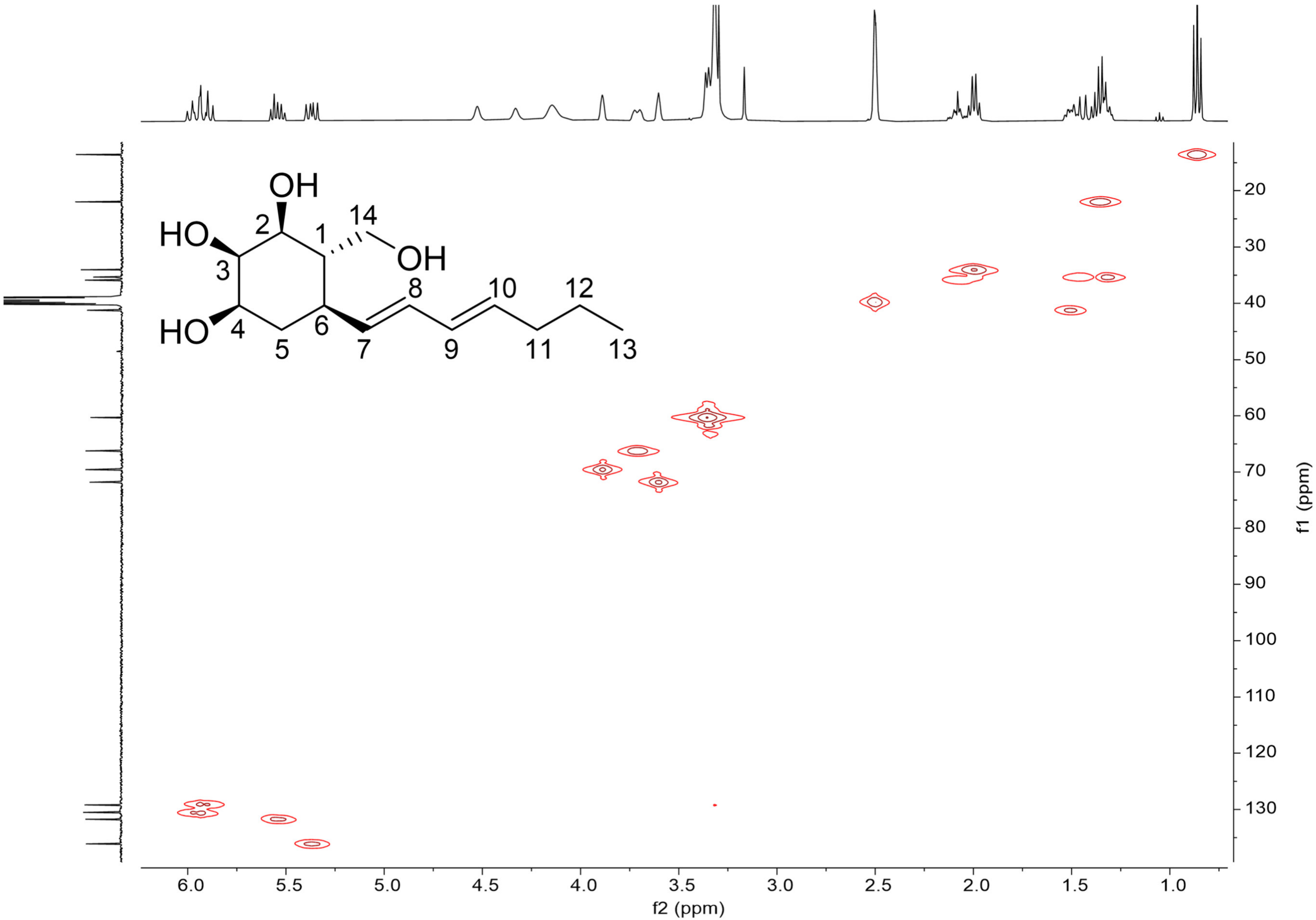

Figure S74. HSQC spectrum of dia-palitantol (**16**) in DMSO-d_6.

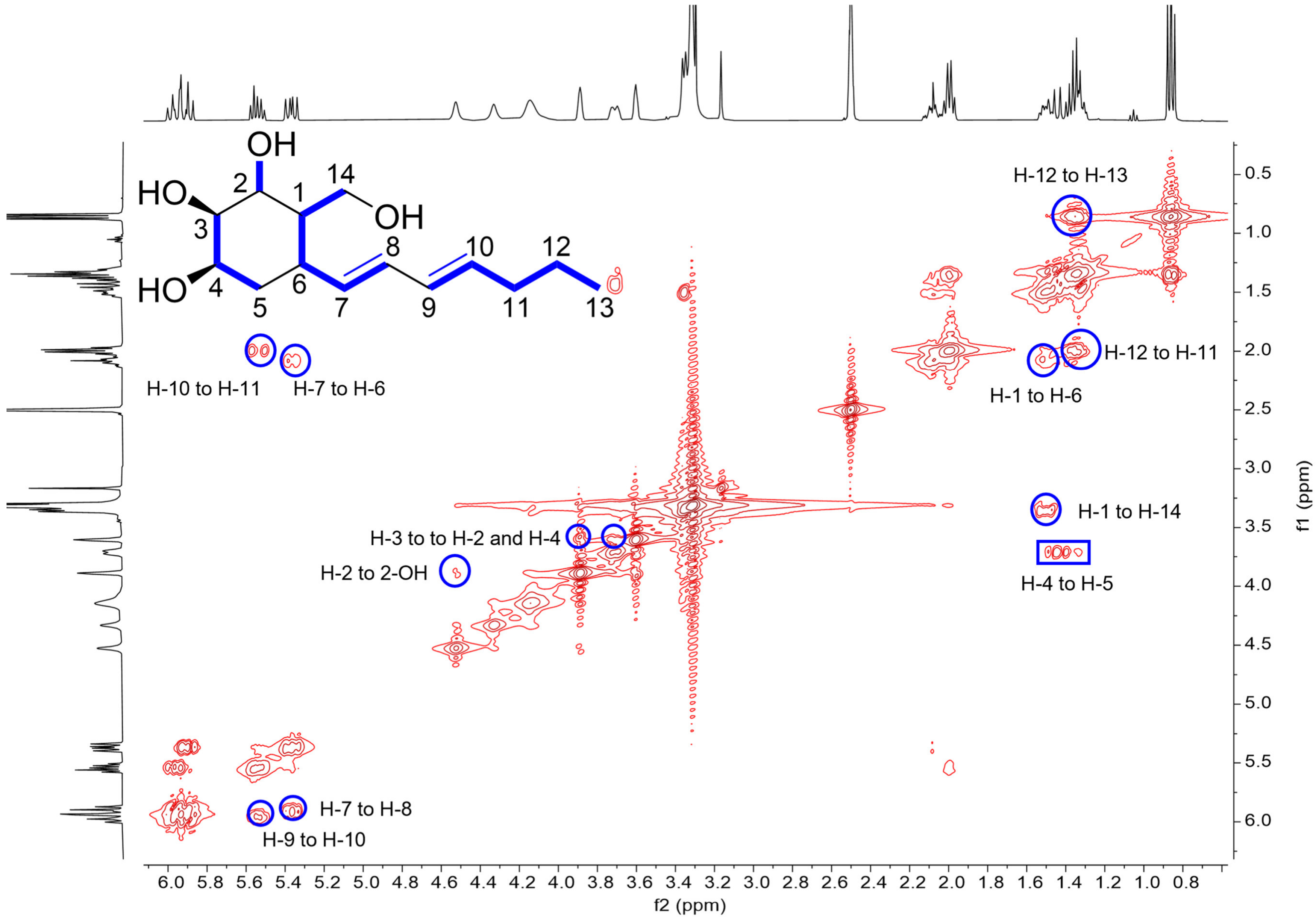

Figure S75. ^1H-^1H COSY spectrum of dia-palitantol (**16**) in DMSO-d_6.

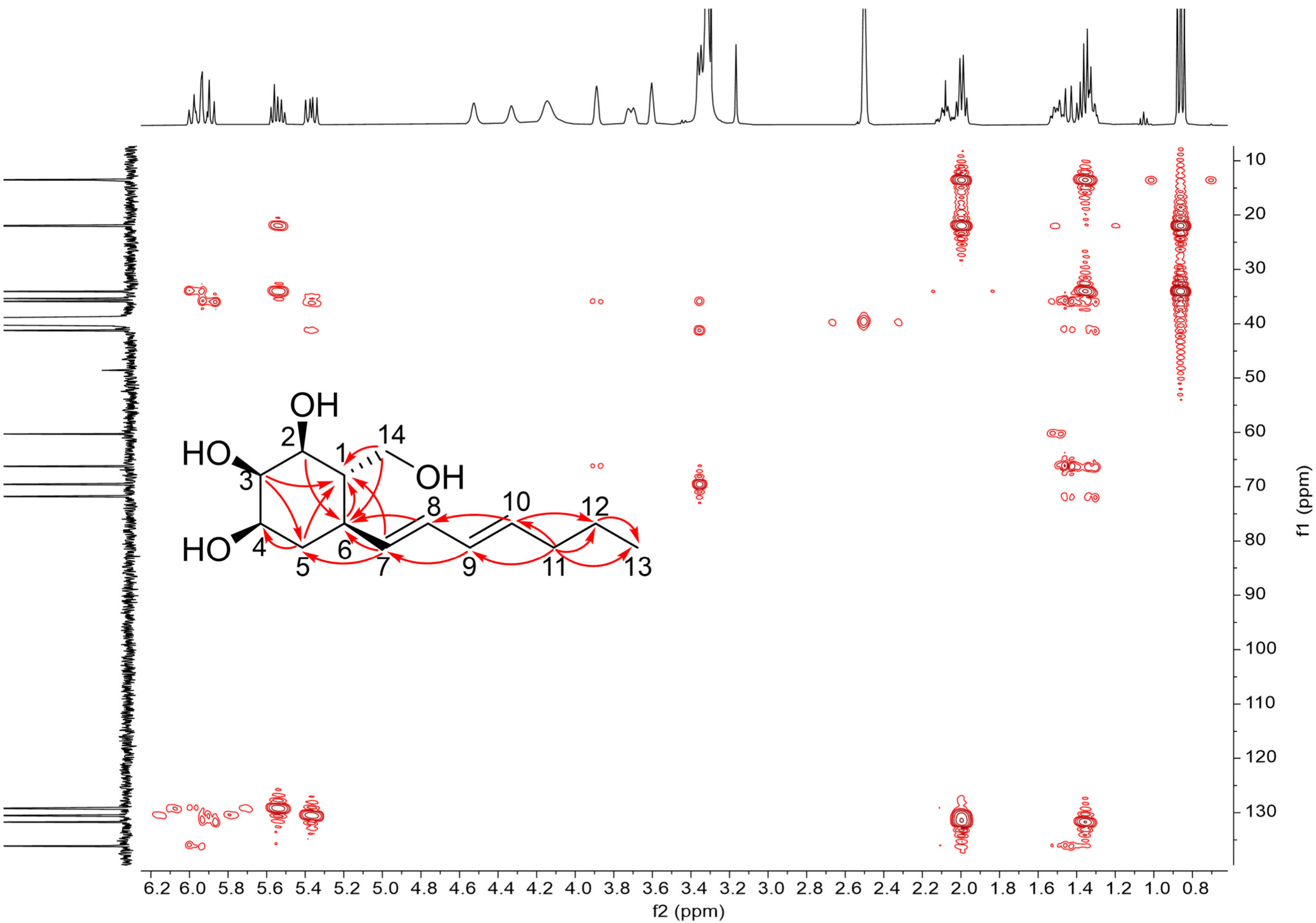

Figure S76. HMBC spectrum of dia-palitantol (**16**) in DMSO-d_6.

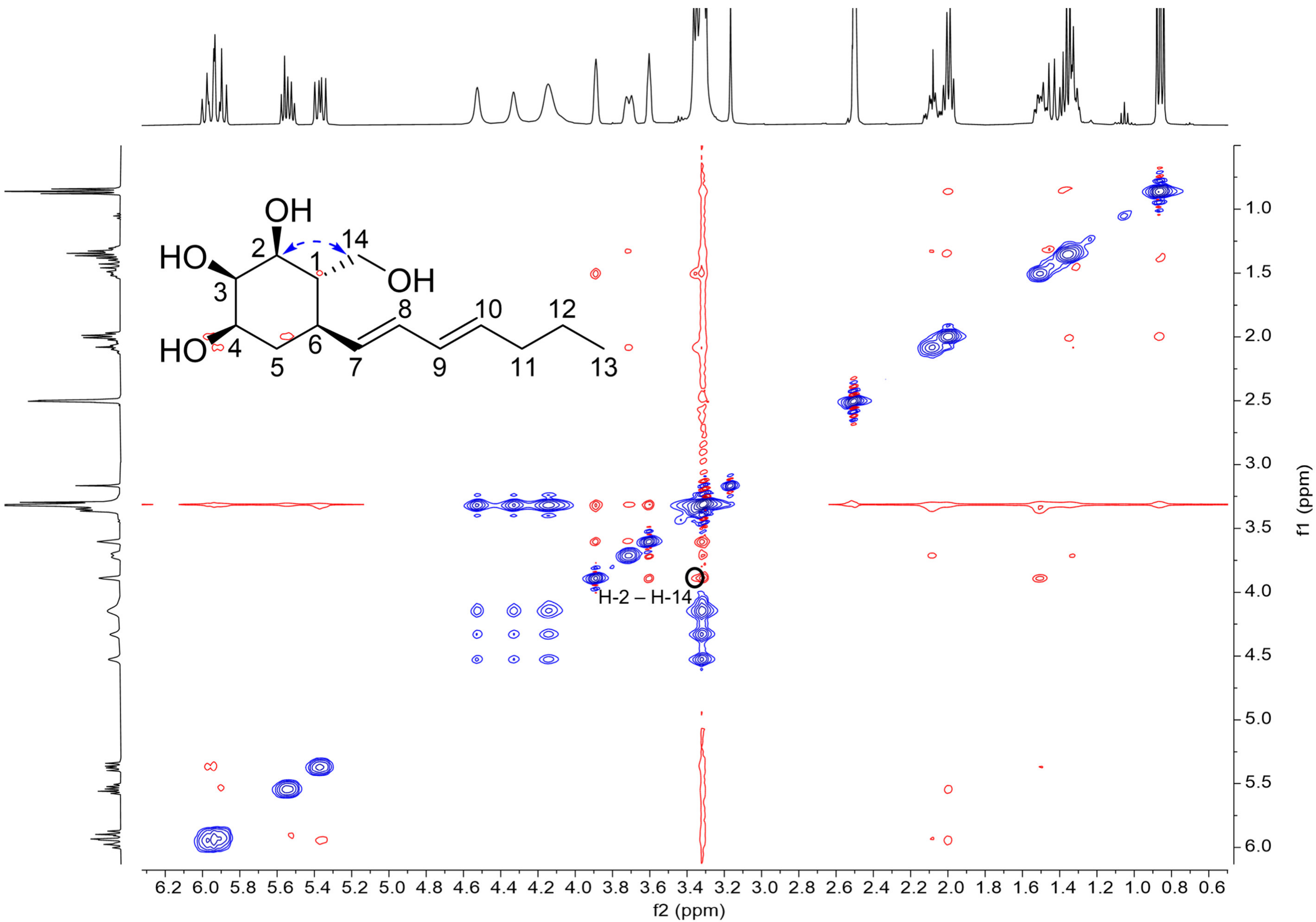

Figure S77. NOESY spectrum of dia-palitantol (**16**) in DMSO-d_6.

Supplementary References

1 Z.-X. Zhang, Z.-H. Li and S.-M. Li, *J. Nat. Prod.*, 2023, **86**, 1053–1060.

2 M. R. Green and J. Sambrook, *Molecular cloning. A laboratory manual*, Cold Spring Harbor Laboratory Press, Cold Spring Harbor N.Y., 4th edn., 2012.

3 F. W. Studier and B. A. Moffatt, *J. Mol. Biol.*, 1986, **189**, 113–130.

4 L. Zheng, H. Wang, A. Fan and S.-M. Li, *Nat. Commun.*, 2020, **11**, 4914.

5 Z.-X. Zhang, Z.-H. Li, W.-B. Yin and S.-M. Li, *Org. Lett.*, 2022, **24**, 262–267.

6 a) K. Blin, S. Shaw, A. M. Kloosterman, Z. Charlop-Powers, G. P. van Wezel, M. H. Medema and T. Weber, *Nucleic Acids Res.*, 2021, **49**, W29–W35; b) S. F. Altschul, W. Gish, W. Miller, E. W. Myers and D. J. Lipman, *J. Mol. Biol.*, 1990, **215**, 403–410;

7 A. P. Jacobus and J. Gross, *PLoS One*, 2015, **10**, e0119221.

8 J. Fan, G. Liao, F. Kindinger, L. Ludwig-Radtke, W.-B. Yin and S.-M. Li, *J. Am. Chem. Soc.*, 2019, **141**, 4225–4229.

9 W. B. Yin, Y. H. Chooi, A. R. Smith, R. A. Cacho, Y. Hu, T. C. White and Y. Tang, *ACS Synth. Biol.*, 2013, **2**, 629–634.

10 Z.-H. Li, Y. Dai, J. Zhou, L. Yang and S.-M. Li, *Org. Lett.*, 2025, **27**, 2433–2437.

11 W. Li, Y. Dai, H.-L. Wei, J. Zhou, J. Fan, Z.-H. Li, X. Xie and S.-M. Li, *Org. Lett.*, 2024, **26**, 10464–10469.

12 a) X.-Y. Chen, Q. Yang and Y. Zou, *Angew. Chem., Int. Ed.*, 2025, e202516403; b) Y.-C. Chen, J.-J. Wu, M.-H. Chen and S.-M. Li, *Chemistry – A European Journal*, 2026, **32**, e03453;

13 J. H. Birkinshaw and H. Raistrick, *Biochem. J.*, 1936, **30**, 801–808.

14 P. M. Santucci, C. R. de Oliveira, A. F. Monteiro, G. R. Mendes, M. R. de Amorim, G. H. Rosa, L. D. Sette, R. C. Marchi, V. M. Deflon, P. H. dos Santos, G. G. Carvalho, M. Brocchi, R. Guido, A. G. Ferreira, K. T. de Oliveira and R. G. Berlinck, *Fitoterapia*, 2025, **180**, 106310.

15 Y. Kimura, T. Mizuno and A. Shimada, *Tetrahedron Lett.*, 1997, **38**, 469–472.

16 G. P.-J. Hareau, M. Koiwa, S. Hikichi and F. Sato, *J. Am. Chem. Soc.*, 1999, **121**, 3640–3650.

17 L. Liu, M. C. Tang and Y. Tang, *J. Am. Chem. Soc.*, 2019, **141**, 19538–19541.

18 Y. M. Chiang, M. Ahuja, C. E. Oakley, R. Entwistle, A. Asokan, C. Zutz, C. C. Wang and B. R. Oakley, *Angew. Chem., Int. Ed.*, 2016, **55**, 1662–1665.

19 M. Peter, Y. Yang and S.-M. Li, *RSC Adv.*, 2022, **12**, 28171–28177.
